# Synthesis and Antiprotozoal Profile of 3,4,5‐Trisubstituted Isoxazoles

**DOI:** 10.1002/open.202100141

**Published:** 2021-07-30

**Authors:** Fernanda Andreia Rosa, Samara Mendes de Souza Melo, Karlos Eduardo Pianoski, Julia Poletto, Mariellen Guilherme dos Santos, Michael Jackson Vieira da Silva, Danielle Lazarin‐Bidóia, Hélito Volpato, Sidnei Moura, Celso Vataru Nakamura

**Affiliations:** ^1^ Departamento de Química Universidade Estadual de Maringá (UEM) Maringá Brazil; ^2^ Departamento de Ciências Básicas da Saúde Universidade Estadual de Maringá (UEM) Maringá Brazil; ^3^ Instituto de Biotecnologia Universidade de Caxias do Sul (UCS) Caxias do Sul Brazil

**Keywords:** antileishmanial drugs, antiprotozoal agents, cyclocondensation reactions, isoxazoles, *N-*acylhydrazone derivatives

## Abstract

A series of 60 4‐aminomethyl 5‐aryl‐3‐substituted isoxazoles were synthesized by an efficient method and evaluated in vitro against *Leishmania amazonensis* and *Trypanosoma cruzi*, protozoa that cause the neglected tropical diseases leishmaniasis and Chagas disease, respectively. Thirteen compounds exhibited a selective index greater than 10. The series of 3‐*N*‐acylhydrazone isoxazole derivatives bearing the bithiophene core exhibited the best antiparasitic effects.

## Graphical abstract

**Sixty new 3,4,5‐trisubstituted isoxazole** derivatives were synthesized using a straightforward method, and their antiprotozoal profile against promastigote form of *L. amazonensis* and epimastigote form of *T. cruzi* were evaluated. The series of 3‐*N*‐acylhydrazone isoxazoles bearing the bithiophene core exhibited better antiparasitic effects than 3‐carboxyethyl or 3‐carbohydrazide isoxazole analogues.

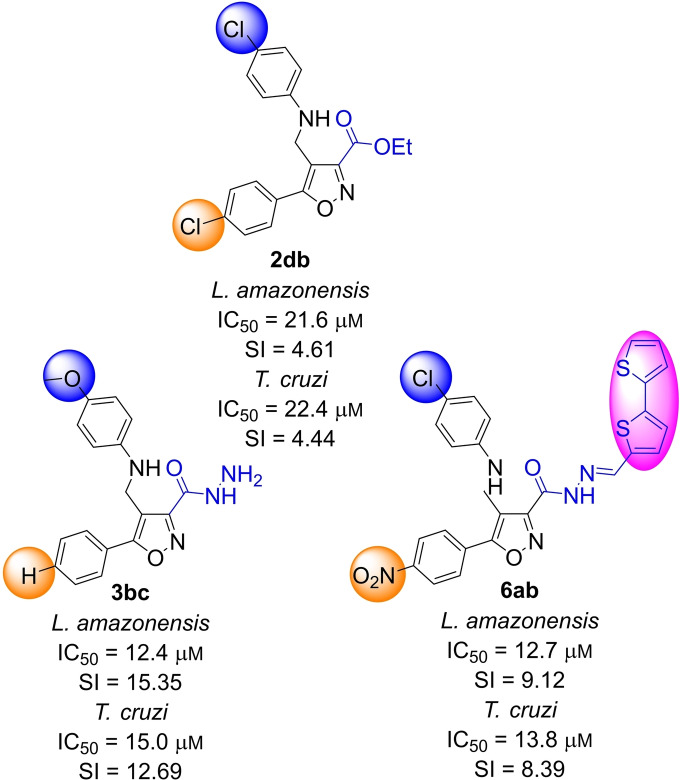

## 1 Introduction

Neglected tropical diseases (NTDs) are a health problem that primarily affects poor and marginalized people in developing countries. These diseases are caused by parasitic organisms that affect millions of people around the world. Among them, it is important to highlight leishmaniasis and Chagas disease, both with a greater occurrence in tropical and sub‐tropical countries, especially in Latin America.^[1]^

Leishmaniasis has been reported in 98 countries, with over a million people annually infected. This chronic disease is transmitted to mammals by the bite of infected sand flies with flagellated protozoa of the genus *Leishmania*. The clinical manifestations depend on the *Leishmania* species. Visceral leishmaniasis (VL) is the most serious clinical form, and cutaneous leishmaniasis (CL) is the most frequent.[^2^, ^3a^]

In addition, Chagas disease, caused by the vector‐borne flagellate protozoan parasite *Trypanosoma cruzi*, is another NTD. This disease has infected over 20 million people in Central and South America and is responsible for around 20000 deaths per year.[^3b^, ^4^]

The current treatments for leishmaniasis and Chagas disease are based on outdated drugs with serious side effects, high cost, prolonged treatment period, and parasite resistance.[^2^, ^3^, ^4^] Thus, the development of new and safe drugs for NTDs is extremely important and urgent. Nevertheless, few governments and pharmaceutical companies have researched drug discovery for NTDs, making the progress even more difficult. Furthermore, the lack of a complete understanding of the parasites’ biology has been a significant limitation on target‐based drug discovery.^[5]^ It is noteworthy that the crucial role of the trypanothione metabolism for parasite infectivity survival and absence in humans offers an attractive pathway to drive the drug discovery. So, efforts have been devoted to the development of key enzyme inhibitors of trypanothione metabolism. However, due to the ability of the parasite to survive with reduced levels of trypanothione, this strategy might fail to discover effective antiprotozoal drugs.^[6]^ In this way, many studies have screened for growth inhibitors against parasite forms. From this approach, it is possible to identify promising inhibitors that might be optimized further to discover new chemical entities for potential application in the treatment of neglected diseases.

**Scheme 1:**
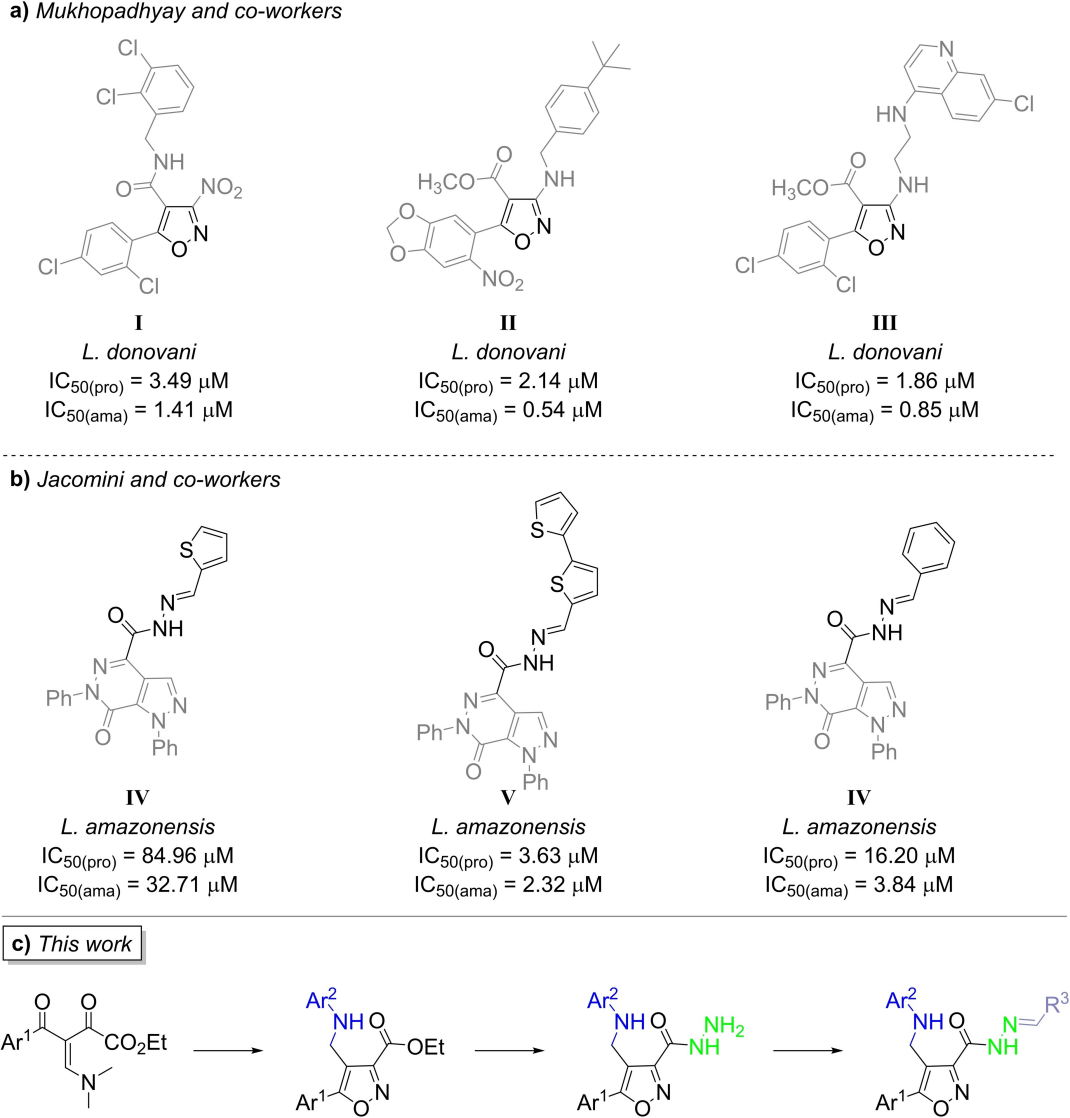
Antiprotozoal activities of isoxazole core (**a**) and pyrazolo[3,4‐*d*]pyridazin‐7‐one‐*N*‐acylhydrazone (**b**). Compounds synthesized and evaluated in this work (**c**).

In medicinal chemistry, the heterocyclic core displays a well‐known and wide range of pharmacology properties. In particular, the isoxazole ring is a privileged heterocycle that exhibits a broad spectrum of biological activities,[^7^, ^8^, ^9^] including antileishmanial[^10^, ^11^, ^12^, ^13^, ^14^, ^15^, ^16^, ^17^] and trypanocidal.[^14^, ^15^, ^16^, ^17^, ^18^, ^19^] Furthermore, most of these studies have reported the antileishmanial and trypanocidal activities of 3,5‐disubstituted isoxazoles, whereas the 3,4,5‐trisubstituted isoxazoles have not been as extensively studied. This fact could be related to the approaches reported for the synthesis of functionalized isoxazoles.^[9]^ Although 3,5‐disubstituted isoxazoles can be synthesized by conventional methods using pre‐functionalized building blocks, the methodologies to access 3,4,5‐trisubstituted isoxazoles are more limited. Recently, Mukhopadhyay and co‐workers^[13]^ published the synthesis of 4,5‐disubstituted‐3‐nitro/amino‐isoxazoles from a one‐pot transformation of Morita‐Baylis‐Hillman acetates and evaluated the antileishmanial activity against promastigote and amastigote forms of *L. donovani* (Scheme 1a).

Our research group has been working on the development of methodologies for the regioselective synthesis of heterocycles with potential pharmacological properties in special antileishmanial activity.[^20^, ^21^, ^22^, ^23^] One of these methodologies allowed for the highly regioselective synthesis of 3,4,5‐trisubstituted isoxazoles from the cyclocondensation of β‐enamino diketones with hydroxylamine.^[24]^ Furthermore, we have observed the importance of the *N*‐acylhydrazone (NAH) moiety on aza‐heterocyclic rings against antileishmanial activity.[^20^, ^21^, ^23^] For instance, 1,4,6‐trisubstituted pyrazolo[3,4‐*d*]pyridazin‐7‐one‐*N*‐acylhydrazone hybrids **IV**, **V**, and **VI** exhibited good activity values against both forms of *L. amazonensis* (Scheme 1b), whereas its precursor carbohydrazide was not active.[^20^, ^21^]

Thus, with the purpose of developing potent antiprotozoan compounds that are less toxic and more selective, in this work, we report the synthesis of a new series of 4,5‐disubstituted isoxazole 3‐*N*‐acylhydrazone hybrids (Scheme 1c) as well as the evaluation of their antiprotozoan activity against the promastigote form of *Leishmania amazonensis* and the epimastigote form of *Trypanosoma cruzi*.

## 2 Results and Discussion

### 2.1 Chemistry

**Scheme 2:**
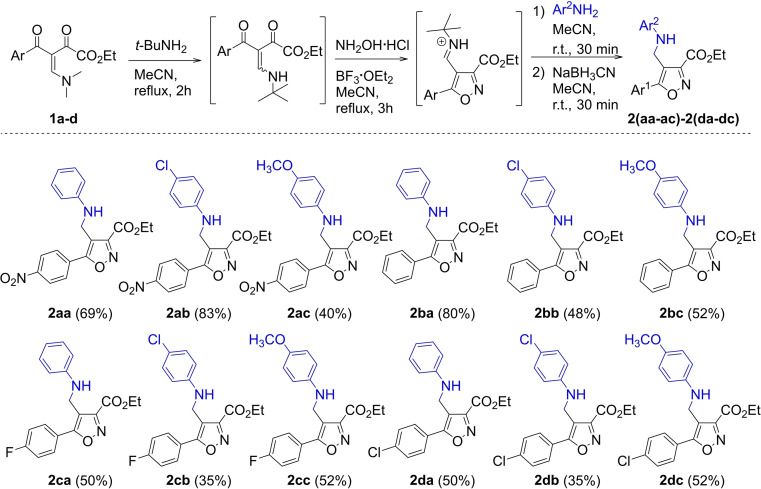
One‐pot synthesis of 3‐carboxyethyl‐4‐[(aryl)aminomethyl]‐5‐arylisoxazoles 2. [a] Reaction conditions: β‐enamino diketone **1 a**–**d** (1.0 mmol), *t*‐Bu‐amine (1.05 mmol), MeCN (4.0 mL), hydroxylamine hydrochloride (1.2 mmol), BF_3_OEt_2_ (2.0 mmol), arylamine (3.0 mmol), NaBH_3_CN (1.2 mmol). [b] Isolated yield after recrystallization in ethanol or purification by column chromatography.

We started our investigation by a one‐pot synthesis of 3‐carboxyethyl‐4‐[(aryl)aminomethyl]‐5‐arylisoxazoles **2**(**aa**–**ac**)‐**2**(**da**–**dc**) from β‐enamino diketones **1 a**–**d**. To this, we followed the recently reported methodologies by our research group (Scheme 2).[^24^, ^25^, ^26^] Gratifyingly, this reaction provided 12 new 4‐aminomethyl isoxazoles **2** with high regioselectivity. All substrates **1 a**–**d** lead to the desired product with moderate to good yields (35–83 %) (Scheme 2).

**Scheme 3:**
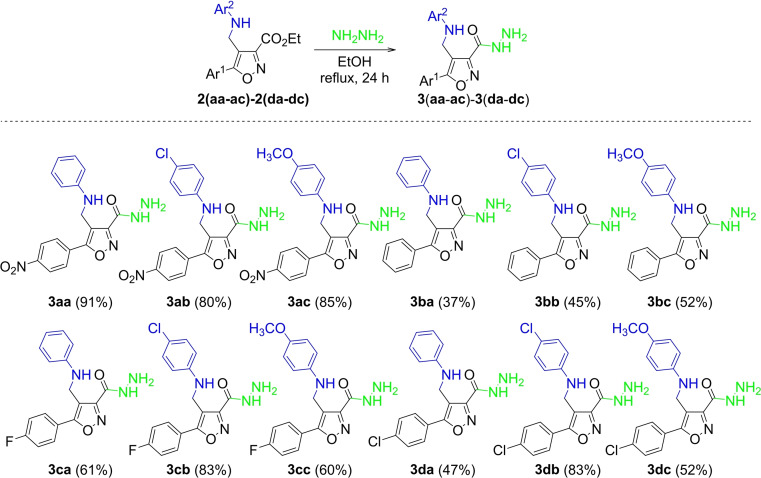
Synthesis of 3‐carbohydrazide derivatives **3**. [a] Reaction conditions: Isoxazole **2** (1.0 mmol, 1.0 equiv.), hydrazine monohydrate (20.0 mmol, 20.0 equiv.), EtOH (4.0 mL). [b] Isolated yield after filtration and washed with cold water.

Next, the 3‐carboxyethyl derivatives **2** were transformed into corresponding 3‐carbohydrazide derivatives **3** by hydrazinolysis^[20]^ (Scheme 3).

**Scheme 4:**
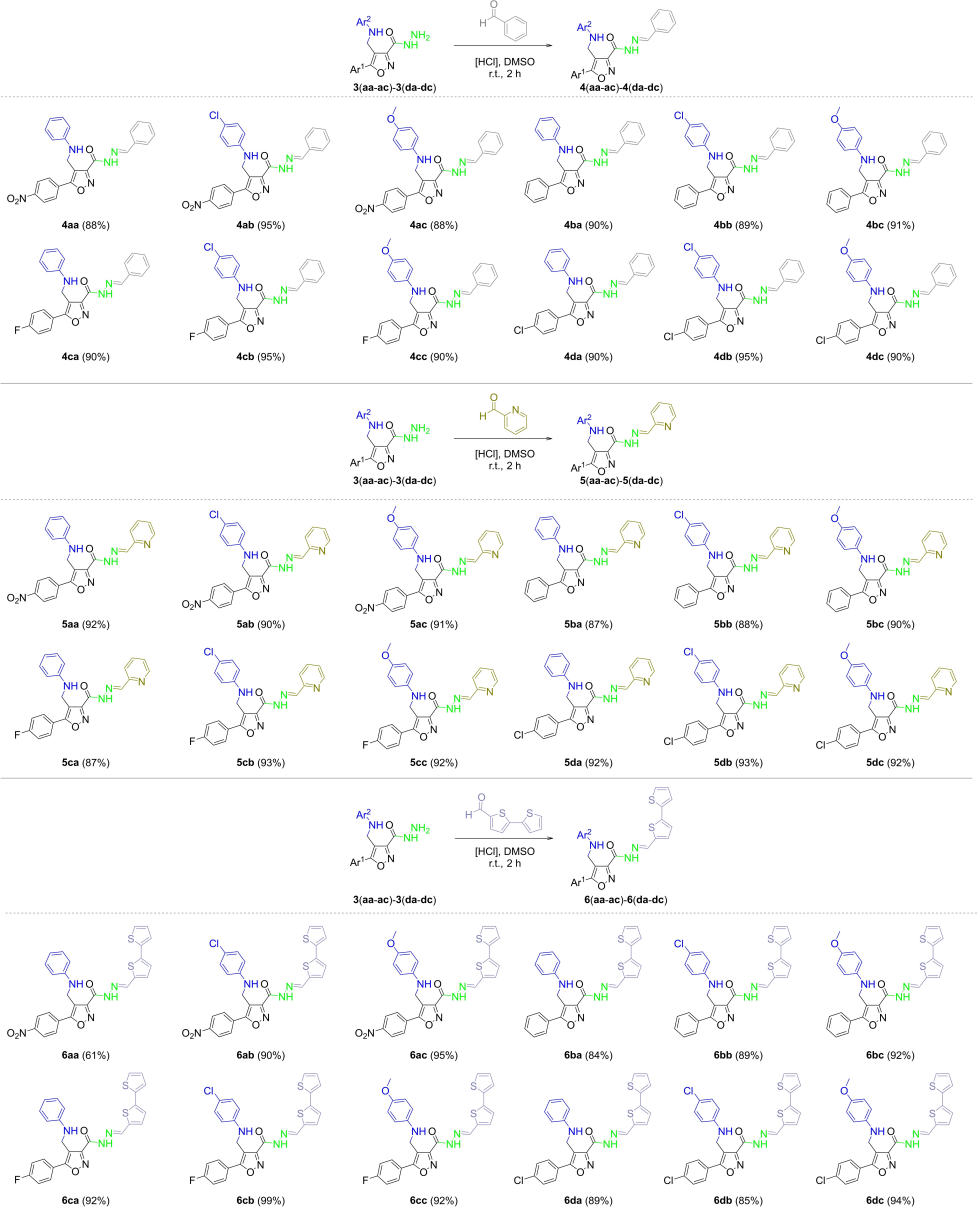
Synthesis of 3‐*N*‐acylhydrazone derivatives **4**, **5**, and **6**. [a] Reaction conditions: Isoxazole **3** (1.0 mmol), benzaldehyde, 2‐formylpyridine or 2,2'‐bithiophene‐5‐carboxaldehyde (1.0 mmol), DMSO (2.0 mL), Two drops of hydrochloric acid (37 %). [b] Isolated yield after filtration and washed with cold water.

Due to the great influence of the *N‐*acylhydrazone moiety on the activity, we performed the condensation reaction of the 3‐carbohydrazide derivatives **3** with benzaldehyde, 2‐formylpyridine, and 2,2'‐bithiophene‐5‐carboxaldehyde using acid conditions. 3‐*N*‐acylhydrazone isoxazole derivatives **4**, **5**, and **6** were obtained in good to excellent yields (61–99 %) (Scheme 4).

The NAH derivatives (**4**, **5**, and **6**) can exist as four possible isomers due to the relative configuration of the imino double bond. However, in the ^1^H NMR spectra of NAH **4**, **5**, and **6**, only a set of signals was observed, which indicates the presence of only one isomeric form in the solution. On the basis of previous reported literature[^27^, ^28^] and spectroscopic data, we attributed the relative configuration of NAH derivatives to (*E*)‐diastereomer. All compounds were characterized using spectroscopic and spectrometric data (see Supplementary information for more details).

### 2.2 Antiproliferative and Cytotoxic Assays

All the novel isoxazole derivatives (**2**, **3**, **4**, **5**, and **6**) were evaluated against promastigote forms of *L. amazonensis* and epimastigote forms of *T. cruzi*, and the results were expressed as half maximal inhibitory concentration (IC_50_). Additionally, the toxicity of the compounds was evaluated against two different cell lines: epithelial cell LLCMK2 and macrophages J774 A1. The antiproliferative activity, cytotoxicity, and the calculated selectivity index (SI) data are summarized in Table 1. Compounds with IC_50_ >200 μm were considered inactive. Almost all compounds displayed antiproliferative activity, with many of them exhibiting IC_50_ less than 50 μm. Isoxazole 3‐*N*‐acylhydrazone‐(bi)thiophene derivatives **6** showed better antiproliferative activity than the other isoxazole derivatives (**2**, **3**, **4**, or **5**).

**Table 1:** In vitro antiproliferative activity in *L. amazonensis*, *T. cruzi* and cytotoxicity in mammalian cells treated with the compounds.

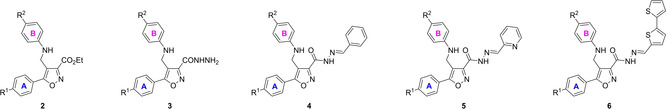										
Compounds	*R*^*1*^ Ring A	*R* ^*2*^ *Ring B*	*L. amazonensis* IC_50 [_μm] ^*[a]*^	*T. cruzi* IC_50 [_μm] ^*[b]*^	Fibroblast CC_50 [_μm] ^*[c]*^	Macrophages CC_50_ [μm] ^*[d]*^	SI_L‐F_^*[e]*^	SI_L‐M_^*[f]*^	SI_T‐F_^*[g]*^	SI_T‐M_^*[h]*^
**2 aa**	NO_2_	H	34.8±1.7	54.2±3.0	269.5±8.9	202.5±6.5	7.74	5.82	4.97	3.74
**2 ab**		Cl	53.7±0.9	59.1±8.7	204.8±1.3	194.6±4.2	3.81	3.62	3.46	3.29
**2 ac**		MeO	28.0±1.2	21.6±2.0	170.4±6.2	144.1±1.4	6.09	5.04	7.89	6.67
**2 ba**	H	H	25.4±3.1	28.5±1.3	203.5±3.0	200.6±7.7	8.01	7.90	7.14	7.04
**2 bb**		Cl	70.6±8.5	81.3±5.3	483.7±10.2	408.3±7.9	6.85	5.78	5.94	5.02
**2 bc**		MeO	45.1±5.8	32.8±2.9	369.2±4.9	297.5±2.9	8.18	6.59	11.25	9.07
**2 ca**	F	H	25.9±2.6	32.4±3.1	245.1±9.2	202.7±5.2	9.46	7.82	7.56	6.25
**2 cb**		Cl	42.1±5.2	39.6±2.3	214.6±4.7	174.0±5.8	5.10	4.13	5.42	4.40
**2 cc**		MeO	39.0±1.5	37.9±4.4	315.7±8.2	288.0±3.6	8.09	7.38	8.32	7.59
**2 da**	Cl	H	29.9±2.1	32.9±3.7	157.8±7.7	154.7±3.0	5.28	5.17	4.80	4.70
**2 db**		Cl	21.6±2.2	22.4±2.6	99.5±5.7	87.9±4.6	4.61	4.07	4.44	3.92
**2 dc**		MeO	28.5±4.1	37.5±4.0	285.7±9.5	205.4±7.6	10.0	7.21	7.62	5.48
**3 aa**	NO_2_	H	42.1±1.3	39.8±3.6	198.3±9.5	142.8±0.8	4.71	3.39	4.98	3.59
**3 ab**		Cl	**15.7±0.4**	**12.1±0.9**	98.3±4.1	91.7±3.8	6.26	5.84	8.12	7.58
**3 ac**		MeO	76.7±0.6	82.9±3.4	499.0±2.7	402.6±11.5	6.51	5.25	6.02	4.86
**3 ba**	H	H	34.9±2.9	39.5±5.6	188.7±9.0	110.3±5.6	5.41	3.16	4.78	2.79
**3 bb**		Cl	35.7±6.0	39.0±7.1	288.1±9.9	231.4±8.1	8.07	6.48	7.38	5.93
**3 bc**		MeO	**12.4±1.7**	**15.0±0.8**	190.4±3.7	185.7±6.1	**15.35**	**14.97**	**12.69**	**12.38**
**3 ca**	F	H	20.3±0.9	22.8±1.7	197.5±5.0	174.0±8.1	11.51	8.57	10.25	7.63
**3 cb**		Cl	171.8±10.4	>200	801.6±11.4	694.2±10.7	4.67	4.04	3.86	3.69
**3 cc**		MeO	59.2±5.7	65.3±3.6	165.7±9.6	149.5±4.4	2.80	2.53	2.54	2.29
**3 da**	Cl	H	23.1±1.5	27.9±2.3	305.2±11.4	256.9±6.8	13.21	11.12	10.93	9.20
**3 db**		Cl	59.1±4.6	55.7±10.2	381.0±4.2	336.0±5.1	6.44	5.68	6.84	6.03
**3 dc**		MeO	55.8±3.9	49.5±2.9	309.6±8.9	209.7±3.5	5.55	3.76	6.25	4.24
**4 aa**	NO_2_	H	40.7±3.2	35.1±4.6	118.0±7.3	100.5±6.0	2.90	2.47	1.07	2.86
**4 ab**		Cl	132.6±8.8	146.8±10.7	398.5±6.9	287.4±2.3	3.01	2.18	2.71	1.96
**4 ac**		MeO	**14.5±2.2**	**18.7±1.4**	**241.2±3.8**	**209.3±8.5**	**16.63**	**14.43**	**12.89**	**11.19**
**4 ba**	H	H	76.8±8.7	69.6±3.9	408.6±7.8	385.6±3.2	5.32	5.02	5.87	5.54
**4 bb**		Cl	27.8±0.7	27.0±1.1	321.8±9.7	305.3±7.4	11.57	10.98	11.91	11.30
**4 bc**		MeO	21.5±0.8	26.7±3.9	209.6±4.7	176.4±6.6	9.75	8.20	7.85	6.61
**4 ca**	F	H	134.7±11.4	148.6±10.3	813.8±13.5	784.4±10.6	6.04	5.83	5.47	5.29
**4 cb**		Cl	87.3±6.4	98.7±10.1	381.2±6.9	365.1±8.7	4.37	4.18	3.86	3.69
**4 cc**		MeO	56.3±8.5	78.6±7.4	307.5±9.8	299.8±8.9	5.46	5.32	3.91	3.81
**4 da**	Cl	H	48.6±2.8	49.7±2.2	265.5±5.9	235.7±9.6	5.46	4.85	5.34	4.74
**4 db**		Cl	67.4±3.9	71.0±4.7	404.7±4.0	395.1±9.9	6.00	5.86	5.70	5.56
**4 dc**		MeO	54.3±2.7	65.9±6.1	295.8±9.0	244.6±7.9	5.45	4.50	4.49	3.71
**5 aa**	NO_2_	H	125.8±2.1	122.9±10.9	407.3±3.9	388.5±7.9	3.24	3.09	3.31	3.16
**5 ab**		Cl	26.0±2.1	24.7±2.9	100.9±4.4	94.2±3.9	3.88	3.55	4.08	3.81
**5 ac**		MeO	>200	>200	800.9±10.4	723.6±3.0	–	–	–	–
**5 ba**	H	H	35.8±4.8	33.6±4.4	329.5±5.6	303.2±9.5	9.20	8.47	9.80	9.02
**5 bb**		Cl	27.8±4.1	26.9±0.9	338.2±8.6	300.8±5.9	12.38	11.01	12.57	11.18
**5 bc**		MeO	**17.9±3.7**	**19.4±1.1**	184.3±5.1	176.8±9.8	**10.3**	**9.88**	**9.50**	**9.11**
**5 ca**	F	H	74.8±2.9	69.5±3.7	402.7±8.6	374.4±5.8	5.38	5.01	5.79	5.38
**5 cb**		Cl	22.6±5.0	39.4±3.1	154.0±7.8	112.0±6.8	6.81	4.96	3.91	2.84
**5 cc**		MeO	**18.0±2.5**	**19.7±0.9**	290.9±4.7	255.4±8.1	**16.16**	**14.18**	**14.76**	**12.96**
**5 da**	Cl	H	69.0±8.7	72.6±3.8	298.0±5.7	289.4±9.5	4.32	4.19	4.10	3.98
**5 db**		Cl	87.9±2.0	97.3±3.6	202.6±3.1	198.5±4.6	2.30	2.26	2.08	2.04
**5 dc**		MeO	**17.4±2.0**	**17.9±0.7**	107.4±2.2	98.6±2.3	**6.17**	**5.67**	**6.00**	**5.51**
**6 aa**	NO_2_	H	32.9±0.9	38.4±2.1	287.4±7.3	209.6±9.6	8.74	6.37	7.48	5.46
**6 ab**		Cl	**12.7±1.1**	**13.8±1.5**	115.8±5.5	95.3±5.3	**9.12**	**7.50**	**8.39**	**6.91**
**6 ac**		MeO	48.9±7.1	52.5±2.9	230.6±5.9	183.6±3.0	4.72	3.75	4.40	3.50
**6 ba**	H	H	46.9±3.0	52.4±2.9	208.6±2.9	196.5±4.4	4.45	4.19	3.98	3.75
**6 bb**		Cl	**18.4±1.2**	**19.0±0.7**	226.5±8.7	188.2±7.4	**12.31**	**10.22**	**11.92**	**9.90**
**6 bc**		MeO	**17.2±3.1**	**18.1±2.4**	197.8±5.9	170.5±3.3	**11.5**	**9.91**	**10.9**	**9.42**
**6 ca**	F	H	34.6±2.4	42.7±5.6	387.5±4.7	308.5±3,0	11.2	8.92	9.07	7.22
**6 cb**		Cl	**19.7±2.6**	**20.4±3.6**	214.7±8.9	209.5±3.1	**10.9**	**10.6**	**10.5**	**10.3**
**6 cc**		MeO	**20.6±1.7**	**22.8±3.0**	315.6±10.8	267.2±7.9	**15.32**	**12.97**	**13.84**	**11.71**
**6 da**	Cl	H	28.0±1.3	27.2±2.5	347.1±8.4	312.1±4.3	12.39	11.14	12.76	11.47
**6 db**		Cl	38.5±4.6	33.6±4.8	309.5±3.1	300.5±4.2	8.04	7.81	9.21	8.94
**6 dc**		MeO	32.7±3.0	30.5±1.6	397.5±7.8	308.9±8.0	12.16	9.45	13.0	10.1

[a] IC_50_ values against promastigote form of *Leishmania amazonensis*. [b] IC_50_ values of epimastigote form of *Trypanosoma cruzi*. [c] Cytotoxic concentration corresponding to 50 % inhibition of fibroblast growth. [d] Cytotoxic concentration corresponding to 50 % inhibition of macrophages growth. [e] Selectivity index (Fibroblast CC_50_/*L. amazonensis* IC_50_). [f] Selectivity index (Macrophages CC_50_/*L. amazonensis* IC_50_). [g] Selectivity index (Fibroblast CC_50_/*T. cruzi* IC_50_). [h] Selectivity index (Macrophages CC_50_/*T. cruzi* IC_50_).

The series of compounds **2** exhibited IC_50_ values in the range 21.6 to 70.6 μm and 21.6 to 81.3 μM against promastigote and epimastigote forms of *L. amazonensis* and *T. cruzi*, respectively. It was found that compounds containing a chlorine atom at the *para*‐position of the phenyl ring A and a chlorine atom or methoxy group at the *para*‐position of the ring B (**2 db** and **2 dc**) exhibited the best results compared to their analogs. However, the presence of a chlorine atom at the ring B led to the compounds with the lowest SI (**2 ab**, **2 bb**, **2 cb**, and **2 db**) compared to those with a methoxy group or a hydrogen atom at the ring B.

Carbohydrazide derivatives **3** showed a similar activity profile against promastigote and epimastigote forms, with IC_50_ values in the range 12.4 to 171.8 μm and 12.1 to >200 μm, respectively. However, the carbohydrazide derivative **3 db** was 2.7‐fold less active than their precursor carboxyethyl **2 db**. Nevertheless, three compounds had a significant increase in activity and selectivity index when the carboxyethyl group was replaced by the carbohydrazide group (**3 ab**, **3 bb**, **3 bc**). For instance, the compound **3 bc** (IC_50(pro)_=12.4 μm; SI 15.35 and 14.97) was 3.6‐fold more active than its analog **2 bc** (IC_50(pro)_=45.1 μm; SI 8.18_(f)_ and 6.59_(m)_) and exhibited a selective index greater than 14.

To series of NAH derivatives **4**, the compounds exhibited IC_50_ values in the range 14.5 to 134.7 μm and 18.7 to 148.6 μm against *L. amazonensis* and *T. cruzi*, respectively, demonstrating an antiprotozoal profile. Derivatives with R^2^=OMe on ring B (**4 ac**, **4 bc**, and **4 cc**) were found more active than the compounds containing R^2^=H or Cl on ring B, with the exception of **4 dc**. The compound containing a methoxy group on ring B and a nitro group on ring A (**4 ac**) was the most active and selective of the series. Furthermore, the transformation of the carbohydrazide **3 ac** (IC_50(pro)_=76.7 μm and IC_50(pro)_=82.9 μm; SI 4.86–6.51) into its NAH derivative **4 ac** (IC_50(pro)_=14.5 μm and IC_50(epi)_=18.7 μm; SI 11.19–16.63) led to an approximately 4‐fold and 5‐fold increase in anti‐leishmanial and anti‐*Trypanosoma cruzi* activities, respectively, with a better selectivity index.

Among series of NAH derivatives **5**, the compounds with R^2^=OMe on ring B (**5 bc**, **5 cc**, and **5 dc**) were more actives against *L. amazonensis* and *T. cruzi* compared to the compounds containing R^2^=H or Cl on ring B, except **5 ac**, which was inactive (IC_50_ >200 μm). Among the most active compounds **5 bc**, **5 cc**, and **5 dc**, the NAH with a fluorine atom on ring A (**5 cc**) presented a better selective index (SI=12.96–16.16), whereas the NAH with a chlorine atom on ring A (**5 dc**) had the worst selective index (SI=5.51–6.17).

The series of NAH derivatives **6** presented a greater amount of active compounds among all of the evaluated series of compounds, exhibiting IC_50_ values in the range 12.7 to 48.9 μm and 13.8 to 52.5 μm against *L. amazonensis* and *T. Cruzi*, respectively. NAHs containing a chlorine atom or methoxy group on ring B were more active than the hydrogen atom on ring B, except **6 da**. Among the most active NAHs with R^2^=Cl (**6 ab**, **6 bb**, **6 cb**), the derivative **6 bb** containing a hydrogen atom on ring A had a better SI. The results obtained from NAH **6** compared to their carbohydrazide precursors **3** corroborate the importance of the NAH group containing a bithiophene ring for anti‐leishmanial activity.

The comparison between the NAH derivatives **4**, **5**, and **6** has shown that the compounds containing a fluorine or hydrogen atom at ring A and a methoxy group or chlorine atom at ring B had the better selectivity indexes (>10). These results indicate that ring A does not seem to tolerate substituents or tolerate light bulky groups, such as a fluorine atom. In contrast, ring B seems to tolerate bulky groups, such as a chlorine atom and methoxy group.

## 3 Conclusion

60 new 3,4,5‐trisubstituted isoxazoles have been prepared using simple and efficient methodologies that allowed the construction of an isoxazole core and structural variations on the 3‐, 4‐, and 5‐positions of that heterocyclic ring. The antiproliferative activity of the isoxazole derivatives was tested in vitro against the promastigote form of *L. amazonensis* and epimastigote form of *T. cruzi*. The most active series against both protozoa was isoxazole 3‐*N*‐acylhydrazone derivatives containing a bithiophene core (compounds **6**). These results contributed to describing the importance of the isoxazole *N*‐acylhydrazone hybrids to the development of potential antiparasitic agents. From the results obtained in this study, the compounds **3 bc**, **4 ac**, and **6 ab** could be considered as lead structures for further studies in the optimization of potent and selective antiprotozoal agents.

## Experimental Section

### General

The reagents used were obtained by the commercial supplier without previous purification. Solvents were dried and purified according to recommended procedures.^[29]^ All the reactions were monitored by thin‐layer chromatography with Merck TLC silica gel plates and analyzed with UV light. All melting points were measured using a MQAPF‐307 Microquímica apparatus using benzoic acid as an internal standard. ^1^H NMR, ^13^C NMR, HSQC, and HMBC experiments were run on a Bruker Avance III HD apparatus operating at ^1^H 300 and 500 MHz and ^13^C 75 and 125 MHz. Chemical shifts are reported in ppm using TMS as the internal standard for CDCl_3_ in ^1^H and ^13^C. ESI(+)‐MS and tandem ESI(+)‐MS/MS were acquired using a hybrid high‐resolution and high accuracy microTof (Q‐TOF) mass spectrometer (Bruker). For ESI(+)‐MS, the energy for the collision‐induced dissociations (CDI) was optimized for each component. For data acquisition and processing, the Q‐TOF‐control data analysis software (Bruker Scientific) was used.

### General Synthetic Procedure and Spectral Data

#### Synthesis of 3‐Carboxyethyl‐4‐[(aryl)aminomethyl]‐5‐arylisoxazoles 2(aa–ac)‐2(da–dc)

To a solution of β‐enamino diketone **1** ^[30]^ (**1 a**: 0.320 g; **1 b**: 0.275 g; **1 c**: 0.293 g; **1 d**: 0.307 g, 1.0 mmol, 1.0 equiv.) in MeCN (4 mL) was added *tert*‐butyl amine (0.0384 g, 1.05 equiv.), and the mixture was stirred under reflux for 2 h. Next, hydroxylamine hydrochloride (0.083 g, 1.2 mmol, 1.2 equiv.) and boron trifluoride diethyl etherate solution 46.5 % (0.530 mL, 2.0 mmol, 2.0 equiv.) were added, and the mixture was stirred under reflux for 3 h. Then, the reaction mixture was cooled to room temperature, substituted arylamine (3.0 mmol, 3.0 equiv.) was added, and the reaction mixture was stirred for 30 min. Next, sodium cyanoborohydride (0.037 g, 1.2 equiv.) was added, and the reaction was stirred for another 30 min. Then, the solvent was evaporated under a vacuum, and the obtained residue was washed with a solution of NaCl (25 mL), extracted with dichloromethane (3×20 mL), and dried over anhydrous sodium sulfate. The solvent was evaporated under reduced pressure, and the obtained residue was purified by recrystallization in ethanol or isolated on a silica gel chromatography column using a 70 : 30 mixture of hexane: ethyl acetate as the eluent.

Experimental data for all the compounds are reported in the Supporting Information. An example for each series of compounds is described as follows.

**3‐Carboxyethyl‐4‐(phenyl)aminomethyl‐5‐(4‐nitrophenyl)isoxazole (2 aa)**: Orange solid; 69 % yield; mp 152.4–153.7 °C; ^1^H NMR (300.06 MHz, CDCl_3_) δ (ppm) 1.42 (*t*, 3H, OCH_2_CH_3_, *J=*7.1 Hz), 4.46 (*s*, 2H, NHCH_2_), 4.49 (*q*, 2H, OCH_2_CH_3_, *J*=7.1 Hz), 6.60 (*dd*, 2H, C_6_H_5_, *J*=8.6; 1.0 Hz), 6.79 (*dd*, 1H, C_6_H_5_, *J*=7.4; 7.4 Hz), 7.17 (*dd*, 2H, C_6_H_5_, *J*=8.6; 7.4 Hz), 7.97 (*d*, 2H, 4‐NO_2_−C_6_H_4_, *J*=9.0 Hz), 8.37 (*d*, 2H, 4‐NO_2_−C_6_H_4_, *J*=9.0 Hz); ^13^C NMR (75.45 MHz, CDCl_3_) δ (ppm) 14.2 (OCH_2_ CH_3_), 37.1 (NHCH_2_), 62.8 (OCH_2_CH_3_), 113.9 (C_6_H_5_), 115.3 (C4), 119.1 (C_6_H_5_), 124.6, 128.8 (4‐NO_2_−C_6_H_4_), 129.7 (C_6_H_5_), 132.6 (4‐NO_2_−C_6_H_4_), 147.4, (C_6_H_5_), 149.1 (4‐NO_2_−C_6_H_4_), 156.0 (C3), 160.6 (C=O), 167.5 (C5); HRMS (ESI+): calcd for C_19_H_18_N_3_O_5_^+^, [M+H]^+^: 368.1241, found 368.1260.

#### Synthesis of 3‐Carbohydrazide‐4‐[(aryl)aminomethyl]‐5‐arylisoxazoles 3(aa–ac)‐3(da–dc)

The isoxazole **2** (1.0 mmol, 1.0 equiv.) was solubilized in EtOH (4 mL), and monohydrate of hydrazine (0.350 g, 20.0 mmol, 20.0 equiv.) was added. The mixture was stirred under reflux for 24 h. Then, the solvent was evaporated under a vacuum, and the residue was filtered and washed with cold water. The solid was dried under a vacuum.

**3‐Carbohydrazide‐4‐(phenyl)aminomethyl‐5‐(4‐nitrophenyl)isoxazole (3 aa)**: Dark yellow solid; 91 % yield; mp 189.0–190.4 °C; ^1^H NMR (300.06 MHz, DMSO‐*d_6_*) δ (ppm) 4.39 (*d*, 2H, NHCH_2_, *J=*4.8 Hz), 4.72 (*ls*, 2H, NHNH_2_), 5.87 (*ls*, 1H, NHCH_2_), 6.56–6.61 (*m*, 3H, C_6_H_5_), 7.03–7.09 (*m*, 2H, C_6_H_5_), 8.07 (*d*, 2H, 4‐NO_2_−C_6_H_4_, *J*=9.0 Hz), 8.39 (*d*, 2H, 4‐NO_2_−C_6_H_4_, *J*=9.0 Hz), 10.22 (*sl*, 1H, NHNH_2_); ^13^C NMR (75.45 MHz, DMSO‐*d_6_*) δ (ppm) 35.6 (NHCH_2_), 112.7 (C_6_H_5_), 114.8 (C4), 116.8 (C_6_H_5_), 124.4, 128.8 (4‐NO_2_−C_6_H_4_), 129.9 (C_6_H_5_), 132.6, 148.2 (4‐NO_2_−C_6_H_4_), 148.3 (C_6_H_5_), 157.9 (C=O), 158.2 (C3), 165.3 (C5); HRMS (ESI+): calcd for C_17_H_16_N_5_O_4_^+^, [M+H]^+^: 354.1197, found 354.1196.

#### Synthesis of 3‐[(2E)‐N'‐(Benzylidene)hydrazinecarbonyl]‐4‐[(aryl)aminomethyl]‐5‐arylisoxazoles 4(aa–ac)‐4(da–dc)

Compound **3** (1.0 mmol, 1.0 equiv.) was solubilized in DMSO (2 mL), and benzaldehyde (0.106 g, 1.0 mmol, 1.0 equiv.) and two drops of hydrochloric acid (37 %) were added. The mixture was stirred at room temperature for 2 h. Then, cold distilled water (100.0 mL) was added, and the product was filtered under vacuum and washed with cold distilled water. The solid was dried under a vacuum.

**3‐[(2*E*)‐*N*'‐(Benzylidene)hydrazinecarbonyl]‐4‐(phenyl)aminomethyl‐5‐(4‐nitrophenyl)isoxazole (4 aa)**: Yellow solid; 88 % yield; mp 92.6–94.4 °C; ^1^H NMR (300.06 MHz, DMSO‐*d_6_*) δ (ppm) 4.46 (*s*, 2H, NHCH_2_), 5.95 (*ls*, 1H, NHCH_2_), 6.58–6.61 (*m*, 3H, C_6_H_5_ – A), 7.43–7.09 (*m*, 2H, C_6_H_5_ – A), 7.45 (*ddd*, 1H, C_6_H_5_ – B, *J*=7.3, 4.9, 1.3 Hz), 7.89 (*ddd*, 1H, C_6_H_5_ – B, *J*=7.7, 7.7, 1.5 Hz), 7.96–7.99 (*m*, 1H, C_6_H_5_ – B), 8.11 (*d*, 2H, 4‐NO_2_−C_6_H_4_, *J*=9.0 Hz), 8.42 (*d*, 2H, 4‐NO_2_−C_6_H_4_, *J*=9.0 Hz), 8.54 (*s*, 1H, NCH), 8.63 (*d*, 1H, C_6_H_5_ – B), 12.68 (*s*, 1H, NHN); ^13^C NMR (75.45 MHz, DMSO‐*d_6_*) δ (ppm) 35.6 (NHCH_2_), 112.7 (C_6_H_5_), 115.4 (C4), 116.7 (C_6_H_5_), 124.4 (4‐NO_2_−C_6_H_4_), 127.3, 128.6, 128.9 (C_6_H_5_ – A and B), 128.9 (4‐NO_2_−C_6_H_4_), 130.5 (C_6_H_5_ – A or B), 132.1 (4‐NO_2_−C_6_H_4_), 133.9, 148.2 (C_6_H_5_ – A and B), 148.4 (4‐NO_2_−C_6_H_4_), 149.8 (C=N), 155.3 (C3), 157.4 (C=O), 165.8 (C5); HRMS (ESI+): calcd for C_24_H_20_N_5_O_4_^+^, [M+H]^+^: 442.1510, found 442.1515.

#### Synthesis of 3‐[(2E)‐N'‐(2‐pyridinylmethylene)hydrazinecarbonyl]‐4‐[(aryl)aminomethyl]‐5‐arylisoxazoles 5(aa–ac)‐5(da–dc)

Compound **3** (1.0 mmol, 1.0 equiv.) was solubilized in DMSO (2 mL), and 2‐formylpyridine (0.107 g, 1.0 mmol, 1.0 equiv.) and two drops of hydrochloric acid (37 %) were added. The mixture was stirred at room temperature for 2 h. Then, cold distilled water (100.0 mL) was added, and the product was filtered under vacuum and washed with cold distilled water. The solid was dried under a vacuum.

**3‐[(2*E*)‐*N'*‐(2‐pyridinylmethylene)hydrazinecarbonyl]‐4‐(phenyl)aminomethyl‐5‐(4‐nitrophenyl)isoxazole (5 aa)**: Orange solid; 92 % yield; mp 223.8–225.5 °C; ^1^H NMR (300.06 MHz, DMSO‐*d_6_*) δ (ppm) 4.46 (*s*, 2H, NHCH_2_), 5.95 (*ls*, 1H, NHCH_2_), 6.58–6.61 (*m*, 3H, C_6_H_5_), 7.04–7.09 (*m*, 2H, C_6_H_5_), 7.45 (*ddd*, 1H, 2‐C_5_H_4_N, *J*=7.3, 4.9, 1.3 Hz), 7.89 (*ddd*, 1H, 2‐C_5_H_4_N, *J*=7.7, 7.7, 1.5 Hz), 7.96–7.99 (*m*, 1H, 2‐C_5_H_4_N), 8.11 (*d*, 2H, 4‐NO_2_−C_6_H_4_, *J*=9.0 Hz), 8.42 (*d*, 2H, 4‐NO_2_−C_6_H_4_, *J*=9.0 Hz), 8.54 (*s*, 1H, NCH), 8.63 (*d*, 1H, 2‐C_5_H_4_N, *J*=4.4 Hz), 12.68 (*s*, 1H, NHN); ^13^C NMR (75.45 MHz, DMSO‐*d_6_*) δ (ppm) 35.6 (NHCH_2_), 112.7 (C_6_H_5_), 115.6 (C4), 116.8 (C_6_H_5_), 120.1 (2‐C_5_H_4_N), 124.5(4‐NO_2_−C_6_H_4_), 124.8 (2‐C_5_H_4_N), 128.7 (4‐NO_2_−C_6_H_4_), 128.9 (C_6_H_5_), 132.0 (4‐NO_2_−C_6_H_4_), 137.0 (2‐C_5_H_4_N), 148.2 (4‐NO_2_−C_6_H_4_), 148.4 (C_6_H_5_), 149.6 (C=N), 150.0, 152.9 (2‐C_5_H_4_N), 155.8 (C3), 157.2 (C=O), 165.9 (C5); HRMS (ESI+): calcd for C_23_H_19_N_6_O_4_^+^, [M+H]^+^: 443.1462, found 443.1493.

#### Synthesis of 3‐[(2E)‐N'‐(2,2'‐bithienyl‐5‐methylene)hydrazinecarbonyl]‐4‐[(aryl)aminomethyl]‐5‐arylisoxazoles 6(aa–ac)‐6(da–dc)

Compound **3** (1.0 mmol, 1.0 equiv.) was solubilized in DMSO (2 mL), and 2,2′‐Bithiophene‐5‐carboxaldehyde (0.194 g, 1.0 mmol, 1.0 equiv.) and two drops of hydrochloric acid (37 %) were added. The mixture was stirred at room temperature for 2 h. Then, cold distilled water (100.0 mL) was added, and the product was filtered under vacuum and washed with cold distilled water. The solid was dried under a vacuum.

**3‐[(2*E*)‐*N'*‐(2,2'‐bithienyl‐5‐methylene)hydrazinecarbonyl]‐4‐(phenyl)aminomethyl‐5‐(4‐nitrophenyl)isoxazole (6 aa)**: Yellow solid; 61 % yield; mp 232.5–233.9 °C; ^1^H NMR (300.06 MHz, DMSO‐*d_6_*) δ (ppm) 4.45 (*s*, 2H, NHCH_2_), 5.95 (*ls*, 1H, NHCH_2_), 6.57–6.60 (*m*, 3H, C_6_H_5_), 7.05–7.08 (*m*, 2H, C_6_H_5_), 7.13 (*dd*, 1H, C_8_H_5_S_2_, *J*=5.1, 3.6 Hz), 7.33 (*d*, 1H, C_8_H_5_S_2_, *J*=3.8 Hz), 7.44–7.46 (*m*, 2H, C_8_H_5_S_2_), 7.60 (*dd*, 1H, C_8_H_5_S_2_, *J*=5.1, 1.1 Hz), 8.11 (*d*, 2H, 4‐NO_2_−C_6_H_4_, *J*=8.9 Hz), 8.42 (*d*, 2H, 4‐NO_2_−C_6_H_4_, *J*=8.9 Hz), 8.63 (*s*, 1H, NCH), 12.47 (*s*, 1H, NHN); ^13^C NMR (75.45 MHz, DMSO‐*d_6_*) δ (ppm) 35.6 (NHCH_2_), 112.7 (C_6_H_5_), 115.5 (C4), 116.8 (C_6_H_5_), 124.4 (4‐NO_2_−C_6_H_4_), 124.4, 125.3, 126.7, 128.6 (C_8_H_5_S_2_), 128.6 (4‐NO_2_−C_6_H_4_), 128.9 (C_6_H_5_), 132.1 (4‐NO_2_−C_6_H_4_), 132.9, 135.9, 137.2, 139.4 (C_8_H_5_S_2_), 144.2 (C=N), 148.2 (4‐NO_2_−C_6_H_4_), 148.4 (C_6_H_5_), 155.2 (C3), 157.3 (C=O), 165.8 (C5); HRMS (ESI+): calcd for C_26_H_20_N_5_O_4_S_2_^+^, [M+H]^+^: 530.0951, found 530.0973.

#### Parasite and Cell culture

The antiproliferative activity was determined in promastigote forms of *Leishmania amazonensis* (WHOM/BR/75/JOSEFA strain) and epimastigote forms of *Trypanosoma cruzi* (Y strain). The promastigote forms of *L. amazonensis* were cultured in Warren medium (brain heart infusion, hemin, and folic acid; pH 7.4) supplemented with 10 % fetal bovine serum (FBS) at 25 °C. The epimastigote forms of *T.cruzi* were cultured in LIT medium (liver infusion tryptose; hemin, and folic acid; pH 7.4) supplemented with 10 % FBS at 28 °C. Cytotoxicity in mammalian cells was determined in fibroblast line (L929) and macrophages (J774 A1). Fibroblasts were cultured in DMEM medium (pH 7.2) supplemented with 10 % FBS at 37 °C in a 5 % CO_2_ atmosphere. Macrophages were cultured in RPMI‐1640 (pH 7.2) medium supplemented with 10 % FBS at 37 °C in a 5 % CO_2_ atmosphere.

#### Dilution of Compounds

Stock solutions of the compounds were prepared in DMSO and then diluted in the respective medium. The groups (controls and treated) were tested with DMSO concentrations below 1 % that do not affect the viability of the protozoa and mammalian cells.

#### Antiproliferative Essay

Promastigote forms (1×10^6^ parasites.mL^−1^) were cultured in 96‐well plates in the presence and absence of different concentrations of compounds diluted in Warren medium supplemented with 10 % FBS and incubated for 72 h. The epimastigote forms (1×10^6^ parasites.mL^−1^) were cultured in 96‐well plates in the presence and absence of different concentrations of compounds diluted in LIT medium supplemented with 10 % FBS and incubated for 96 h. After treatment, the parasites were incubated with a solution of 2,3‐bis(2‐methoxy‐4‐nitro‐5‐sulfophenyl)‐2H‐tetrazolium‐5‐carboxanilide (XTT; 0.5 mg.mL^−1^) and phenazine methanesulfonate activator (PMS; 0.06 mg.mL^−1^) in PBS for 4 h. Then, the absorbance was read in a microplate reader (Bio Tek‐Power Wave XS) at 450 nm.^[20]^ The percentage of viable parasites was calculated in relation to the control in order to determine the concentration that inhibits 50 % of the protozoa (IC_50_). The control groups of each experiment received the same experimental conditions as the treated groups (cell concentration used, temperature, and incubation time).

#### Cytotoxicity Assay in Mammalian Cells

Fibroblast cell (2.5×10^5^ cells.mL^−1^) suspensions were prepared in DMEM medium supplemented with 10 % FBS and added to 96‐well plates. Then, the plates were incubated at 37 °C in a CO_2_ atmosphere for 24 h to obtain confluent cell growth. After incubation, cells were treated with different concentrations of compounds diluted in DMEM for 72 h or left untreated. Macrophage (5×10^5^ cells.mL^−1^) suspensions were prepared in RPMI‐1640 medium supplemented with 10 % FBS and added to 96‐well plates. Then, the plates were incubated at 37 °C in a CO_2_ atmosphere for 24 h to obtain confluent cell growth. After incubation, cells were treated with different concentrations of compounds diluted in RPMI‐1640 for 48 h or left untreated. After treatment, the medium was removed, and cells were incubated with MTT (2 mg.mL^−1^) for 4 h. Then, DMSO was added for solubilization of the formazan and analyzed with a reading microplate reader (BIO‐TEK PowerWave XS spectrophotometer) at 392 nm.^[20]^ The percentage of viable cells was calculated in relation to the untreated control group to determine the cytotoxic concentration in 50 % of the cells (CC_50_). The control groups of each experiment received the same experimental conditions as the treated groups (cell concentration used, temperature, CO_2_, and incubation time).

## Conflict of interest

The authors declare no conflict of interest.

## Supporting information

As a service to our authors and readers, this journal provides supporting information supplied by the authors. Such materials are peer reviewed and may be re‐organized for online delivery, but are not copy‐edited or typeset. Technical support issues arising from supporting information (other than missing files) should be addressed to the authors.

Supporting Information
