# Supplementary material for: Synthesis and Antiprotozoal Profile of 3,4,5‐Trisubstituted Isoxazoles
Source: ChemistryOpen. 2021 Jul 30;10(10):931–8. doi: 10.1002/open.202100141 (PMC8485799; doi:10.1002/open.202100141)
Supplement: Supplementary file 1 — Supporting Information [file OPEN-10-931-s001.pdf]

ChemistryOpen

Supporting Information

Synthesis and Antiprotozoal Profile of 3,4,5-Trisubstituted Isoxazoles

Fernanda Andreia Rosa,* Samara Mendes de Souza Melo, Karlos Eduardo Pianoski, Julia Poletto, Mariellen Guilherme dos Santos, Michael Jackson Vieira da Silva, Danielle Lazarin-Bidóia, Hélito Volpato, Sidnei Moura, and Celso Vataru Nakamura

SUPORTING INFORMATION

Table of contents

1. Synthetic procedure and spectral data S2-S13

2. ^{1}H and ^{13}C spectra for **2(aa-ac)-2(da-dc)** S14-S37

3. ^{1}H and ^{13}C spectra for **3(aa-ac)-3(da-dc)** S38-S61

4. ^{1}H and ^{13}C spectra for **4(aa-ac)-4(da-dc)** S62-S85

5. ^{1}H and ^{13}C spectra for **5(aa-ac)-5(da-dc)** S86-S109

6. ^{1}H and ^{13}C spectra for **6(aa-ac)-6(da-dc)** S110-S133

7. References S134

General Synthetic Procedure and Spectral Data.

Synthesis of 3-Carboxyethyl-4-[(aryl)aminomethyl]-5-arylisoxazoles 2(aa-ac)-2(da-dc)

General method. To a solution of β-enamino diketone **1**[2] (**1a**: 0.320 g; **1b**: 0.275 g; **1c**: 0.293 g; **1d**: 0.307 g, 1.0 mmol, 1.0 equiv) in MeCN (4 mL) was added *tert*-butyl amine (0.0384 g, 1.05 equiv.), and the mixture was stirred under reflux for 2 h. Next, hydroxylamine hydrochloride (0.083 g, 1.2 mmol, 1.2 equiv) and boron trifluoride diethyl etherate solution 46.5% (0.530 mL, 2.0 mmol, 2.0 equiv) were added, and the mixture was stirred under reflux for 3 h. Then, the reaction mixture was cooled to room temperature, substituted arylamine (3.0 mmol, 3.0 equiv) was added, and the reaction mixture was stirred for 30 min. Next, sodium cyanoborohydride (0.037 g, 1.2 equiv.) was added, and the reaction was stirred for another 30 min. Then, the solvent was evaporated under a vacuum, and the obtained residue was washed with a solution of NaCl (25 mL), extracted with dichloromethane (3x20 mL), and dried over anhydrous sodium sulfate. The solvent was evaporated under reduced pressure, and the obtained residue was purified by recrystallization in ethanol (**2aa-2ac** and **2db**) or isolated on a silica gel chromatography column using a 70:30 mixture of hexane: ethyl acetate as the eluent (**2ba-2bc**; **2ca-2cb**; **2da** and **2dc**). The pure product was dried under a vacuum.

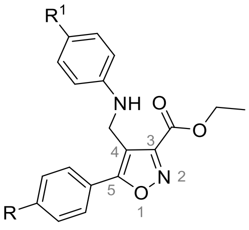

2(aa-ac)-2(da-dc)

3-Carboxyethyl-4-(phenyl)aminomethyl-5-(4-nitrophenyl)isoxazole (**2aa**): Orange solid; 69% yield; mp 152.4-153.7 °C; **^{1}H NMR** (300.06 MHz, $CDCl_3$) δ (ppm) 1.42 (*t*, 3H, $OCH_2C\underline{H_3}$, *J* = 7.1 Hz), 4.46 (*s*, 2H, $NHC\underline{H_2}$), 4.49 (*q*, 2H, $OC\underline{H_2}CH_3$, *J* = 7.1 Hz), 6.60 (*dd*, 2H, C_6H_5, *J* = 8.6; 1.0 Hz), 6.79 (*dd*, 1H, C_6H_5, *J* = 7.4; 7.4 Hz), 7.17 (*dd*, 2H, C_6H_5, *J* = 8.6; 7.4 Hz), 7.97 (*d*, 2H, 4-NO_2-C_6H_4, *J* = 9.0 Hz), 8.37 (*d*, 2H, 4-NO_2-C_6H_4, *J* = 9.0 Hz); **^{13}C NMR** (75.45 MHz, $CDCl_3$) δ (ppm) 14.2 ($OCH_2\underline{C}H_3$), 37.1 ($NH\underline{C}H_2$), 62.8 ($O\underline{C}H_2CH_3$), 113.9 (C_6H_5), 115.3 (C4), 119.1 (C_6H_5), 124.6, 128.8 (4-NO_2-C_6H_4), 129.7 (C_6H_5), 132.6 (4-NO_2-C_6H_4), 147.4, (C_6H_5), 149.1 (4-NO_2-C_6H_4), 156.0 (C3), 160.6 (C=O), 167.5 (C5); **HRMS** (ESI+): calcd for $C_{19}H_{18}N_3O_5^+$, $[M+H]^+$: 368.1241, found 368.1260.

3-Carboxyethyl-4-[(4-chlorophenyl)aminomethyl]-5-(4-nitrophenyl)isoxazole (**2ab**): Orange solid; 71% yield; mp 175.7-176.7 °C; **^{1}H NMR** (300.06 MHz, $CDCl_3$) δ (ppm) 1.43 (*t*, 3H, $OCH_2C\underline{H_3}$, *J* = 7.1 Hz), 4.43 (*s*, 2H, $NHC\underline{H_2}$), 4.49 (*q*, 2H, $OC\underline{H_2}CH_3$, *J* = 7.1 Hz), 6.51 (*d*, 2H, 4-Cl-C_6H_4, *J* = 8.9 Hz), 7.11 (*d*, 2H, 4-Cl-C_6H_4, *J* = 8.9 Hz), 7.94 (*d*, 2H, 4-NO_2-C_6H_4, *J* = 9,0 Hz), 8.39 (*d*, 2H, 4-NO_2-C_6H_4, *J* = 9,0 Hz); **^{13}C NMR** (75.45 MHz, $CDCl_3$) δ (ppm) 14.3 ($OCH_2\underline{C}H_3$), 37.2 ($NH\underline{C}H_2$), 62.9 ($O\underline{C}H_2CH_3$), 115,0 (4-Cl-C_6H_4), 115.2 (C4), 123.8 (4-Cl-C_6H_4), 124.6, 128,7 (4-NO_2-C_6H_4), 129.4 (4-Cl-C_6H_4), 132.4 (4-NO_2-C_6H_4), 145.8 (4-Cl-C_6H_4), 149.1 (4-NO_2-C_6H_4), 155.9 (C3), 160,4 (C=O), 167.4 (C5); **HRMS** (ESI+): calcd for $C_{19}H_{17}ClN_3O_5^+$, $[M+H]^+$: 402.0851, found 402.0869.

3-Carboxyethyl-4-[(4-methoxyphenyl)aminomethyl]-5-(4-nitrophenyl)isoxazole (**2ac**): Brown solid; 40% yield; mp 142.2-144.7 °C; **^{1}H NMR** (300.06 MHz, $CDCl_3$) δ (ppm) 1.43 (*t*, 3H, $OCH_2C\underline{H_3}$, *J* = 7.1 Hz), 3.75 (*s*, 3H, 4-$OC\underline{H_3}$-C_6H_4), 4.40 (*ls*, 2H, $NHC\underline{H_2}$), 4.49 (*q*, 2H, $OC\underline{H_2}CH_3$, *J* = 7.1 Hz), 6.59 (*d*, 2H, 4-OCH_3-C_6H_4, *J* = 8.9 Hz), 6.76 (*d*, 2H, 4-OCH_3-C_6H_4, *J* = 8.9 Hz), 7.97 (*d*, 2H, 4-NO_2-C_6H_4, *J* = 9.0 Hz), 8.37 (*d*, 2H, 4-NO_2-C_6H_4, *J* = 9.0 Hz); **^{13}C NMR** (75.45 MHz, $CDCl_3$) δ (ppm) 14.3 ($OCH_2\underline{C}H_3$), 38.2 ($NH\underline{C}H_2$), 55.7 (4-$O\underline{C}H_3$-C_6H_4), 62.8 ($O\underline{C}H_2CH_3$), 114.9, 115.6 (4-OCH_3-C_6H_4), 115.7 (C4), 124.5, 128.7, 132.5 (4-NO_2-C_6H_4), 141.3 (4-OCH_3-C_6H_4), 148.9 (4-NO_2-C_6H_4), 153.3 (C3), 155.9 (4-OCH_3-C_6H_4), 160.6 (C=O), 167.3 (C5); **HRMS** (ESI+): calcd for $C_{20}H_{20}N_3O_6^+$, $[M+H]^+$: 398.1347, found 398.1360.

3-Carboxyethyl-4-(phenyl)aminomethyl-5-(phenyl)isoxazole (**2ba**): Light Brown solid; 80% yield; mp 95.7-97.0 °C; **^{1}H NMR** (300.06 MHz, $CDCl_3$) δ (ppm) 1.40 (*t*, 3H, $OCH_2C\underline{H_3}$, *J* = 7.1 Hz), 4.39 (*s*, 2H, $NHC\underline{H_2}$), 4.46 (*q*, 2H, $OC\underline{H_2}CH_3$, *J* = 7.1 Hz), 6.57 (*dd*, 2H, C_6H_5 - B, *J* = 8.6; 1.0 Hz), 6.73 (*dd*, 1H, C_6H_5 - B, *J* = 7.4; 7.4 Hz), 7.13 (*dd*, 3H, C_6H_5 - B, *J* = 8.6; 7.4 Hz), 7.51-7.53 (*m*, 3H, C_6H_5 - A), 7.73-7.74 (*m*, 2H, C_6H_5 - A); **^{13}C NMR** (75.45 MHz,

$CDCl_3$) δ (ppm) 14.2 ($OCH_2\underline{C}H_3$), 36.9 ($NH\underline{C}H_2$), 62.5 ($O\underline{C}H_2CH_3$), 113.4 (C4), 113.7, 118.4, 126.9, 127.8, 129.3, 129.4, 130.9, 147.3, (C_6H_5), 155.6 (C3), 160.8 (C=O), 169.9 (C5); **HRMS** (ESI+): calcd for $C_{19}H_{19}N_2O_3^+$, $[M+H]^+$: 323.1390, found 323.1374.

3-Carboxyethyl-4-[(4-chlorophenyl)aminomethyl]-5-(phenyl)isoxazole (**2bb**): Brown solid; 48% yield; mp 138.2-139.1 °C; **^{1}H NMR** (300.06 MHz, $CDCl_3$) δ (ppm) 1.42 (*t*, 3H, $OCH_2C\underline{H_3}$, *J* = 7.1 Hz), 4.39 (*ls*, 2H, $NHC\underline{H_2}$), 4.47 (*q*, 2H, $OC\underline{H_2}CH_3$, *J* = 7.1 Hz), 6.45 (*d*, 2H, 4-Cl-C_6H_4, *J* = 9.0 Hz), 7.05 (*d*, 2H, 4-Cl-C_6H_4, *J* = 9.0 Hz), 7.51-7.53 (*m*, 3H, C_6H_5), 7.70-7.74 (*m*, 2H, C_6H_5); **^{13}C NMR** (75.45 MHz, $CDCl_3$) δ (ppm) 14.2 ($OCH_2\underline{C}H_3$), 37.0 ($NH\underline{C}H_2$), 62.6 ($O\underline{C}H_2CH_3$), 113.1 (C4), 114,8, 123.1 (4-Cl-C_6H_4), 126.8, 127.8 (C_6H_5), 129.2 (4-Cl-C_6H_4), 129.4, 131.1 (C_6H_5), 146.1 (4-Cl-C_6H_4), 155.5 (C3), 160.8 (C=O), 170.0 (C5); **HRMS** (ESI+): calcd for $C_{19}H_{18}ClN_2O_3^+$, $[M+H]^+$: 357.1000, found 357.1017.

3-Carboxyethyl-4-[(4-methoxyphenyl)aminomethyl]-5-(phenyl)isoxazole (**2bc**): Brown solid; 52% yield; mp 131.0-131.5 °C; **^{1}H NMR** (300.06 MHz, $CDCl_3$) δ (ppm) 1.41 (*t*, 3H, $OCH_2C\underline{H_3}$, *J* = 7.1 Hz), 3.70 (*s*, 3H, 4-$OC\underline{H_3}$-C_6H_4), 4.38 (*s*, 2H, $NHC\underline{H_2}$), 4.47 (*q*, 2H, $OC\underline{H_2}CH_3$, *J* = 7.1 Hz), 6.55 (*d*, 2H, 4-OCH_3-C_6H_4, *J* = 9.0 Hz), 6.73 (*d*, 2H, 4-OCH_3-C_6H_4, *J* = 9.0 Hz), 7.51-7.53 (*m*, 3H, C_6H_5), 7.72-7.75 (*m*, 2H, C_6H_5); **^{13}C NMR** (75.45 MHz, $CDCl_3$) δ (ppm) 14.3 ($OCH_2\underline{C}H_3$), 38.1 ($NH\underline{C}H_2$), 55.9 (4-$O\underline{C}H_3$-C_6H_4), 62.5 ($O\underline{C}H_2CH_3$), 113.6 (C4), 114.9, 115.4 (4-OCH_3-C_6H_4), 127.0, 127.8, 129.3, 130.9 (C_6H_5), 141.7, 152.9 (4-OCH_3-C_6H_4), 155.6 (C3), 160.6 (C=O), 169.9 (C5); **HRMS** (ESI+): calcd for $C_{20}H_{21}N_2O_4^+$, $[M+H]^+$: 353.1496, found 353.1497.

3-Carboxyethyl-4-(phenyl)aminomethyl-5-(4-fluorophenyl)isoxazole (**2ca**): White solid; 50% yield; mp 170.4-174.3 °C; **^{1}H NMR** (300.06 MHz, $CDCl_3$) δ (ppm) 1.39 (*t*, 3H, $OCH_2C\underline{H_3}$, *J* = 7.1 Hz), 4.22 (*s*, 1H, $N\underline{H}CH_2$), 4.39 (*s*, 2H, $NHC\underline{H_2}$), 4.46 (*q*, 2H, $OC\underline{H_2}CH_3$, *J* = 7.1 Hz), 6.56-6.77 (*m*, 2H, C_6H_5), 6.72-6.77 (*m*, 1H, C_6H_5), 7.12-7.25 (*m*, 4H, 4-F-C_6H_4 and C_6H_5), 7.72-7.76 (*m*, 2H, 4-F-C_6H_4); **^{13}C NMR** (75.45 MHz, $CDCl_3$) δ (ppm) 14.2 ($OCH_2\underline{C}H_3$), 37.0 ($NH\underline{C}H_2$), 62.5 ($O\underline{C}H_2CH_3$), 113.1 (C4), 113.7 (C_6H_5), 116.6 (*d*, 4-F-C_6H_4, $^2J_{C\text{-}F}$ = 22.1 Hz), 118.6 (C_6H_5), 123.1 (*d*, 4-F-C_6H_4, $^4J_{C\text{-}F}$ = 3.4 Hz), 129.4 (C_6H_5), 130.0 (*d*, 4-F-C_6H_4, $^3J_{C\text{-}F}$ = 8.7 Hz), 147.5 (C_6H_5), 155.6 (C3), 160.6 ($\underline{C}$=O), 164.2 (*d*, 4-F-C_6H_4, $^1J_{C\text{-}F}$ = 252.5 Hz), 169.0 (C5); **HRMS** (ESI+): calcd for $C_{19}H_{18}FN_2O_3^+$, $[M+H]^+$: 341.1296, found 341.1295.

3-Carboxyethyl-4-[(4-chlorophenyl)aminomethyl]-5-(4-fluorophenyl)isoxazole (**2cb**): White solid; 35% yield; mp 110.4-111.8 °C; **^{1}H NMR** (300.06 MHz, $CDCl_3$) δ (ppm) 1.41 (*t*, 3H, $OCH_2C\underline{H_3}$, *J* = 7.1 Hz), 4.37 (*s*, 2H, $NHC\underline{H_2}$), 4.47 (*q*, 2H, $OC\underline{H_2}CH_3$, *J* = 7.1 Hz), 6.47 (*d*, 2H, 4-Cl-C_6H_4, *J* = 9.0), 7.08 (*d*, 2H, 4-Cl-C_6H_4, *J* = 9.0), 7.20-7.26 (*m*, 2H, 4-F-C_6H_4), 7.69-7.74 (*m*, 2H, 4-F-C_6H_4); **^{13}C NMR** (75.45 MHz, $CDCl_3$) δ (ppm) 14.2 ($OCH_2\underline{C}H_3$), 37.1 ($NH\underline{C}H_2$), 62.6 ($O\underline{C}H_2CH_3$), 112.9 (C4), 114.8 (4-Cl-C_6H_4), 116.7 (*d*, 4-F-C_6H_4, $^2J_{C\text{-}F}$ = 22.1 Hz), 123.0 (*d*, 4-F-C_6H_4, $^4J_{C\text{-}F}$ = 3.2 Hz), 129.9 (*d*, 4-F-C_6H_4, $^3J_{C\text{-}F}$ = 9.0 Hz), 130.0, 146.1 (4-Cl-C_6H_4), 155.5 (C3), 160.7 ($\underline{C}$=O), 163.3 (*d*, 4-F-C_6H_4, $^1J_{C\text{-}F}$ = 249.5 Hz), 165.9 (4-Cl-C_6H_4), 169.1 (C5); **HRMS** (ESI+): calcd for $C_{19}H_{17}ClFN_2O_3^+$, $[M+H]^+$: 375.0906, found 375.0916.

3-Carboxyethyl-4-[(4-methoxyphenyl)aminomethyl]-5-(4-fluorophenyl)isoxazole (**2cc**): Yellow solid; 52% yield; mp 81.9-82.8 °C; **^{1}H NMR** (300.06 MHz, $CDCl_3$) δ (ppm) 1.41 (*t*, 3H, $OCH_2C\underline{H_3}$, *J* = 7.1 Hz), 3.74 (*s*, 3H, 4-$OC\underline{H_3}$-C_6H_4), 4.00 (*ls*, 1H, $N\underline{H}CH_2$), 4.34 (*s*, 2H, $NHC\underline{H_2}$), 4.47 (*q*, 2H, $OC\underline{H_2}CH_3$, *J* = 7.1 Hz), 6.56 (*d*, 2H, 4-OCH_3-C_6H_4, *J* = 9.0), 6.75 (*d*, 2H, 4-OCH_3-C_6H_4, *J* = 9.0), 7.18-7.24 (*m*, 2H, 4-F-C_6H_4), 7.73-7.77 (*m*, 2H, 4-F-C_6H_4); **^{13}C NMR** (75.45 MHz, $CDCl_3$) δ (ppm) 14.2 ($OCH_2\underline{C}H_3$), 37.1 ($NH\underline{C}H_2$), 55.9 (4-$O\underline{C}H_3$-C_6H_4), 62.5 ($O\underline{C}H_2CH_3$), 113.4 (C4), 115.0 (4-$O\underline{C}H_3$-C_6H_4), 116.6 (*d*, 4-F-C_6H_4, $^2J_{C\text{-}F}$ = 22.1 Hz), 123.2 (*d*, 4-F-C_6H_4, $^4J_{C\text{-}F}$ = 3.4 Hz), 129.9 (*d*, 4-F-C_6H_4, $^3J_{C\text{-}F}$ = 9.0 Hz), 141.7, 153.1 (4-$O\underline{C}H_3$-C_6H_4), 155.7 (C3), 160.7 ($\underline{C}$=O), 164.2 (*d*, 4-F-C_6H_4, $^1J_{C\text{-}F}$ = 249.5 Hz), 165.9 (4-$O\underline{C}H_3$-C_6H_4), 169.0 (C5); **HRMS** (ESI+): calcd for $C_{20}H_{20}N_2O_4^+$, $[M+H]^+$: 371.1402, found 371.1401.

3-Carboxyethyl-4-(4-(phenyl)aminomethyl-5-(4-chlorophenyl)isoxazole (**2da**): Light yellow solid; 50% yield; mp 172.9-173.4 °C; **^{1}H NMR** (500.13 MHz, $CDCl_3$) δ (ppm) 1.41 (*t*, 3H, $OCH_2C\underline{H_3}$, *J* = 7.1 Hz), 4.41 (*s*, 2H, $NHC\underline{H_2}$), 4.47 (*q*, 2H, $OC\underline{H_2}CH_3$, *J* = 7.1 Hz), 6.58 (*dd*, 2H, C_6H_5, *J* = 8.6, 1.0 Hz), 6.76 (*dd*, 1H, C_6H_5, *J* = 7.4, 7.4 Hz), 7.15 (*dd*, 2H, C_6H_5, *J* = 8.6, 7.4 Hz), 7.50 (*d*, 2H, 4-Cl-C_6H_4, *J* = 9.0 Hz), 7.69 (*d*, 2H, 4-Cl-C_6H_4, *J* = 9.0 Hz); **^{13}C NMR** (125.76 MHz, $CDCl_3$) δ (ppm) 14.2 ($OCH_2\underline{C}H_3$), 37.1 ($NH\underline{C}H_2$), 62.8 ($O\underline{C}H_2CH_3$), 113.6 (C4), 113.8, 118.7 (C_6H_5), 125.3, 129.0 (4-Cl-C_6H_4), 129.4 (C_6H_5), 129.7, 137.3 (4-Cl-C_6H_4), 147.5 (C_6H_5), 155.7 (C3), 160.6 (C=O), 168.8 (C5); **HRMS** (ESI+): calcd for $C_{19}H_{18}ClN_2O_3^+$, $[M+H]^+$: 357.1000, found 357.1017.

3-Carboxyethyl-4-[4-(chlorophenyl)aminomethyl]-5-(4-chlorophenyl)isoxazole (**2db**): Light Yellow solid; 35% yield; mp 173.3-175.4 °C; **^{1}H NMR** (500.13 MHz, $CDCl_3$) δ (ppm) 1.41 (*t*, 3H, $OCH_2C\underline{H_3}$, *J* = 7.1 Hz), 4.38 (*s*, 2H, $NHC\underline{H_2}$), 4.47 (*q*, 2H, $OC\underline{H_2}CH_3$, *J* = 7.1 Hz), 6.48 (*d*, 2H, 4-Cl-C_6H_4 - B, *J* = 9.0 Hz), 7.08 (*d*, 2H, 4-Cl-C_6H_4 - B,

J = 9.0 Hz), 7.51 (*d*, 2H, 4-Cl-C_6H_4 - A, *J* = 9.0 Hz), 7.66 (*d*, 2H, 4-Cl-C_6H_4 - A, *J* = 9.0 Hz); **^{13}C NMR** (125.76 MHz, $CDCl_3$) δ (ppm) 14.2 (OCH_2CH_3), 37.1 ($NHCH_2$), 62.7 (OCH_2CH_3), 113.4 (C4), 114.8, 123.3, 125.2, 129.0 129.3, 129.9, 137.4, 146.0 (4-Cl-C_6H_4 – A and B), 155.6 (C3), 160.7 (C=O), 168.9 (C5); **HRMS** (ESI+): calcd for $C_{19}H_{17}Cl_2N_2O_3^+$, $[M+H]^+$: 391.0611, found 391.0620.

3-Carboxyethyl-4-[(4-methoxyphenyl)aminomethyl]-5-(4-chlorophenyl)isoxazole (**2dc**): Brown solid; 52% yield; mp 144.8-145.9 ^{o}C; **^{1}H NMR** (300.06 MHz, $CDCl_3$) δ (ppm) 1.41 (*t*, 3H, OCH_2CH_3, *J* = 7.1 Hz), 3.74 (*s*, 3H, 4-OCH_3-C_6H_4), 4.35 (*s*, 2H, $NHCH_2$), 4.47 (*q*, 2H, OCH_2CH_3, *J* = 7.1 Hz), 6.56 (*d*, 2H, 4-OCH_3-C_6H_4, *J* = 9.0), 6.75 (*d*, 2H, 4-OCH_3-C_6H_4, *J* = 9.0), 7.49 (*d*, 2H, 4-Cl-C_6H_4, *J* = 9.0), 7.69 (*d*, 2H, 4-Cl-C_6H_4, *J* = 9.0); **^{13}C NMR** (75.45 MHz, $CDCl_3$) δ (ppm) 14.2 (OCH_2CH_3), 38.1 ($NHCH_2$), 55.8 (4-OCH_3-C_6H_4), 62.5 (OCH_2CH_3), 113.8 (C4), 115.0, 115.5 (4-OCH_3-C_6H_4), 125.4, 129.0, 129.6, 137.2 (4-Cl-C_6H_4), 141.6, 153.1 (4-OCH_3-C_6H_4), 155.7 (C3), 160.6 (C=O), 168.8 (C5); **HRMS** (ESI+): calcd for $C_{20}H_{20}ClN_2O_4^+$, $[M+H]^+$: 387.1106, found 387.1097.

Synthesis of 3-Carbohydrazide-4-[(aryl)aminomethyl]-5-arylisoxazoles 3(aa-ac)-3(da-dc)

General method. The isoxazole **2** (1.0 mmol, 1.0 equiv) was solubilized in EtOH (4 mL), and monohydrate of hydrazine (1.0 g, 20.0 mmol, 20.0 equiv) was added. The mixture was stirred under reflux for 24 h. Then, the solvent was evaporated under a vacuum, and the residue was filtered and washed with cold water. The solid was dried under a vacuum.

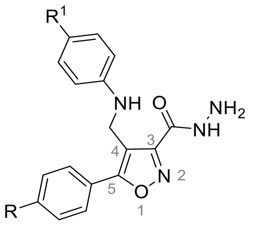

3(aa-ac)-3(da-dc)

3-Carbohydrazide-4-(phenyl)aminomethyl-5-(4-nitrophenyl)isoxazole (**3aa**): Dark yellow solid; 91% yield; mp 189.0-190.4 ^{o}C; **^{1}H NMR** (300.06 MHz, DMSO-*d*$_6$) δ (ppm) 4.39 (*d*, 2H, $NHCH_2$, *J* = 4.8 Hz), 4.72 (*ls*, 2H, $NHNH_2$), 5.87 (*ls*, 1H, $NHCH_2$), 6.56-6.61 (*m*, 3H, C_6H_5), 7.03-7.09 (*m*, 2H, C_6H_5), 8.07 (*d*, 2H, 4-NO_2-C_6H_4, *J* = 9.0 Hz), 8.39 (*d*, 2H, 4-NO_2-C_6H_4, *J* = 9.0 Hz), 10.22 (*sl*, 1H, $NHNH_2$); **^{13}C NMR** (75.45 MHz, DMSO-*d*$_6$) δ (ppm) 35.6 ($NHCH_2$), 112.7 (C_6H_5), 114.8 (C4), 116.8 (C_6H_5), 124.4, 128.8 (4-NO_2-C_6H_4), 129.9 (C_6H_5), 132.6, 148.2 (4-NO_2-C_6H_4), 148.3 (C_6H_5), 157.9 (C=O), 158.2 (C3), 165.3 (C5); **HRMS** (ESI+): calcd for $C_{17}H_{16}N_5O_4^+$, $[M+H]^+$: 354.1197, found 354.1196.

3-Carbohydrazide-4-[(4-chlorophenyl)aminomethyl]-5-(4-nitrophenyl)isoxazole (**3ab**): Yellow solid; 80% yield; mp 174.1-176.8 ^{o}C; **^{1}H NMR** (300.06 MHz, DMSO-*d*$_6$) δ (ppm) 4.39 (*d*, 2H, $NHCH_2$, *J* = 4.83), 4.72 (*ls*, 2H, $NHNH_2$), 5.82 (*ls*, 1H, $NHCH_2$), 6.56-6.60 (*m*, 3H, 4-Cl-C_6H_4), 7.02-7.08 (*m*, 2H, 4-Cl-C_6H_4), 8.07 (*d*, 2H, 4-NO_2-C_6H_4, *J* = 9,0 Hz), 8.39 (*d*, 2H, 4-NO_2-C_6H_4, *J* = 9,0 Hz), 10.21 (*ls*, 1H, $NHNH_2$); **^{13}C NMR** (75.45 MHz, DMSO-*d*$_6$) δ (ppm) 35.6 ($NHCH_2$), 112.7 (4-Cl-C_6H_4), 114.8 (C4), 116.7 (4-Cl-C_6H_4), 124.3, 128.5 (4-NO_2-C_6H_4), 128.8 (4-Cl-C_6H_4), 132.2 (4-NO_2-C_6H_4), 148.1 (4-Cl-C_6H_4), 148.2 (4-NO_2-C_6H_4), 157.8 (C3), 158.1 (C=O), 165.2 (C5); **HRMS** (ESI+): calcd for $C_{17}H_{15}ClN_5O_4^+$, $[M+H-Cl]^+$: 354.1197, found 354.1199.

3-Carbohydrazide-4-[(4-methoxyphenyl)aminomethyl]-5-(4-nitrophenyl)isoxazole (**3ac**): Brown solid; 85% yield; mp 144.2-146.8 ^{o}C; **^{1}H NMR** (300.06 MHz, $CDCl_3$) δ (ppm) 3.73 (*s*, 3H, 4-OCH_3-C_6H_4), 4.42 (*s*, 2H, $NHCH_2$), 6.55 (*d*, 2H, 4-OCH_3-C_6H_4, *J* = 8.9 Hz), 6.72 (*d*, 2H, 4-OCH_3-C_6H_4, *J* = 8.9 Hz), 7.92 (*d*, 2H, 4-NO_2-C_6H_4, *J* = 8.9 Hz), 8.24 (*s*, 1H, $NHNH_2$), 8.38 (*d*, 2H, 4-NO_2-C_6H_4, *J* = 8.9 Hz); **^{13}C NMR** (75.45 MHz, $CDCl_3$) δ (ppm) 38.2 ($NHCH_2$), 55.8 (4-OCH_3-C_6H_4), 115.0 (4-OCH_3-C_6H_4), 115.6 (C4), 116.0 (4-OCH_3-C_6H_4), 124.5, 128.8, 132.6 (4-NO_2-C_6H_4), 141.3 (4-OCH_3-C_6H_4), 149.0 (4-NO_2-C_6H_4), 153.4 (C3), 156.4 (4-OCH_3-C_6H_4), 160.3 (C=O), 166.9 (C5); **HRMS** (ESI+): calcd for $C_{18}H_{18}N_5O_5^+$, $[M+H]^+$: 384.1302, found 384.1317.

3-Carbohydrazide-4-(phenyl)aminomethyl-5-(phenyl)isoxazole (**3ba**): White solid; 37% yield; mp 151.3-152.4 ^{o}C; **^{1}H NMR** (300.06 MHz, $CDCl_3$) δ (ppm) 4.10 (*ls*, 2H, $NHNH_2$), 4.47 (*s*, 2H, $NHCH_2$), 4.70 (*ls*, 1H, $NHCH_2$), 6.52 (*dd*, 2H, C_6H_5 - B, *J* = 8.6; 1.0 Hz), 6.67-6.72 (*m*, 1H, C_6H_5 - B), 7.09 (*dd*, 3H, C_6H_5 - B, *J* = 8.6; 7.4 Hz), 7.53-

7.55 (*m*, 3H, C_6H_5 - A), 7.68-7.72 (*m*, 2H, C_6H_5 - A) 8.16 (*sl,* 1H, N$\underline{H}$NH_2); **^{13}C NMR** (75.45 MHz, $CDCl_3$) δ (ppm) 36.8 (NH$\underline{C}H_2$), 113.3 (C4), 114.0, 118.4, 127.0, 127.9, 129.3, 129.4, 131.0, 147.6 (C_6H_5), 156.1 (C3), 160.8 (C=O), 169.5 (C5); **HRMS** (ESI+): calcd for $C_{17}H_{17}N_4O_2^+$, $[M+H]^+$: 309.1346, found 323.1351.

3-Carbohydrazide-4-[(4-chlorophenyl)aminomethyl]-5-(phenyl)isoxazole (**3bb**): White solid; 45% yield; mp 190.6-191.6 °C; **^{1}H NMR** (300.06 MHz, DMSO-*d₆*) δ (ppm) 4.30 (*d*, 2H, NHC$\underline{H_2}$, *J* = 4.9 Hz), 4.65 (*s*, 2H, NHN$\underline{H_2}$), 6.07 (*t,* 1H, N$\underline{H}$CH_2, *J* = 4.83 Hz), 6.59 (*d*, 2H, 4-Cl-C_6H_4, *J* = 8.8 Hz), 7.08 (*d*, 2H, 4-Cl-C_6H_4, *J* = 8.8 Hz), 7.56-7.58 (*m*, 3H, C_6H_5), 7.76-7.80 (*m*, 2H, C_6H_5), 10.10 (*s*, 1H, N$\underline{H}$NH_2); **^{13}C NMR** (75.45 MHz, DMSO-*d₆*) δ (ppm) 35.7 (NH$\underline{C}H_2$), 112.0 (C4), 113.9, 119.8 (4-Cl-C_6H_4), 126.5, 127.81(C_6H_5), 128.6 (4-Cl-C_6H_4), 129.3, 130.8 (C_6H_5), 147.2 (4-Cl-C_6H_4), 157.6 (C3), 158.4 (C=O), 167.6 (C5); **HRMS** (ESI+): calcd for $C_{17}H_{16}ClN_4O_2^+$, $[M+H]^+$: 343.0956, found 343.0975.

3-Carbohydrazide-4-[(4-methoxyphenyl)aminomethyl]-5-(phenyl)isoxazole (**3bc**): White solid; 52% yield; mp 132.2-135.4 °C; **^{1}H NMR** (300.06 MHz, $CDCl_3$) δ (ppm) 3.72 (*s*, 3H, 4-OC$\underline{H_3}$-C_6H_4), 4.11 (*s*, 2H, NHC$\underline{H_2}$), 4.41 (*s*, 2H, NHN$\underline{H_2}$), 6.52 (*d*, 2H, 4-OCH_3-C_6H_4, *J* = 9.0 Hz), 6.69 (*d*, 2H, 4-OCH_3-C_6H_4, *J* = 9.0 Hz), 7.52-7.54 (*m*, 3H, C_6H_5), 7.67-7.71 (*m*, 2H, C_6H_5), 8.21 (*s*, 1H, N$\underline{H}$NH_2); **^{13}C NMR** (75.45 MHz, $CDCl_3$) δ (ppm) 38.0 (NH$\underline{C}H_2$), 55.8 (4-O$\underline{C}H_3$-C_6H_4), 113.3 (C4), 114.9, 115.7 (4-OCH_3-C_6H_4), 127.0, 127.9, 129.3, 130.9 (C_6H_5), 141.6, 152.9 (4-OCH_3-C_6H_4), 156.2 (C3), 160.8 (C=O), 169.5 (C5); **HRMS** (ESI+): calcd for $C_{20}H_{21}N_2O_4^+$, $[M+H]^+$: 339.1452, found 339.1464.

3-Carbohydrazide-4-(phenyl)aminomethyl-5-(4-fluorophenyl)isoxazole (**3ca**): White solid; 61% yield; mp 174.0-175.0 °C; **^{1}H NMR** (300.06 MHz, $CDCl_3$) δ (ppm) 4.10 (*s*, 2H, NHN$\underline{H_2}$), 4.44 (*s*, 2H, NHC$\underline{H_2}$), 4.64 (*ls*, 1H, N$\underline{H}$NH_2), 6.54 (*dd*, 2H, C_6H_5, *J* = 8.6, 1.0 Hz), 6.70-6.75 (*m*, 1H, C_6H_5), 7.11 (*dd*, 2H, C_6H_5, *J* = 8.6, 7.4 Hz), 7.20-7.23 (*m*, 2H, 4-F-C_6H_4), 7.69-7.73 (*m*, 2H, 4-F-C_6H_4), 8.11 (*s*, 1H, N$\underline{H}$NH_2); **^{13}C NMR** (75.45 MHz, $CDCl_3$) δ (ppm) 36.8 (NH$\underline{C}H_2$), 113.2 (C4), 114.0 (C_6H_5), 116.7 (*d*, 4-F-C_6H_4, $^2J_{C\text{-}F}$ = 22.1 Hz), 118.6 (C_6H_5), 123.2 (*d,* 4-F-C_6H_4, $^4J_{C\text{-}F}$ = 3.4 Hz), 129.4 (C_6H_5), 130.1 (*d*, 4-F-C_6H_4, $^3J_{C\text{-}F}$ = 8.7 Hz), 147.6 (C_6H_5), 156.1 (C3), 160.7 ($\underline{C}$=O), 164.2 (*d,* 4-F-C_6H_4, $^1J_{C\text{-}F}$ = 252.6 Hz), 168.7 (C5); **HRMS** (ESI+): calcd for $C_{17}H_{16}FN_4O_2^+$, $[M+H]^+$: 327.1252, found 327.1236.

3-Carbohydrazide-4-[(4-chlorophenyl)aminomethyl]-5-(4-fluorophenyl)isoxazole (**3cb**): White solid; 83% yield; mp 178.1-181.2 °C; **^{1}H NMR** (300.06 MHz, DMSO-*d₆*) δ (ppm) 4.26 (*s*, 2H, NHC$\underline{H_2}$), 4.67 (*ls*, 2H, NHN$\underline{H_2}$), 5.78 (*s*, 1H, N$\underline{H}$CH_2), 6.57-6.60 (*m*, 2H, 4-Cl-C_6H_4), 6.89-6.93 (*m*, 2H, 4-Cl-C_6H_4), 7.41-7.45 (*m*, 2H, 4-F-C_6H_4), 7.85-7.87 (*m*, 2H, 4-F-C_6H_4), 10.13 (*ls*, 1H, N$\underline{H}$NH_2); **^{13}C NMR** (75.45 MHz, DMSO-*d₆*) δ (ppm) 35.6 (NH$\underline{C}H_2$), 111.9 (C4), 113.9 (4-Cl-C_6H_4), 116.5 (*d,* 4-F-C_6H_4, $^2J_{C\text{-}F}$ = 22.1 Hz), 119.8 (4-Cl-C_6H_4), 123.2 (*d,* 4-F-C_6H_4, $^4J_{C\text{-}F}$ = 3.1 Hz), 128.5 (4-Cl-C_6H_4), 129.7 (*d*, 4-F-C_6H_4, $^3J_{C\text{-}F}$ = 8.9 Hz), 147.2 (4-Cl-C_6H_4), 157.5 (C3), 160.7 ($\underline{C}$=O), 163.3 (*d,* 4-F-C_6H_4, $^1J_{C\text{-}F}$ = 249.3 Hz), 166.8 (C5); **HRMS** (ESI+): calcd for $C_{17}H_{15}ClFN_4O_2^+$, $[M+H]^+$: 361.0962, found 375.0876.

3-Carbohydrazide-4-[(4-methoxyphenyl)aminomethyl]-5-(4-fluorophenyl)isoxazole (**3cc**): Yellow solid; 60% yield; mp 171.1-173.6 °C; **^{1}H NMR** (300.06 MHz, $CDCl_3$) δ (ppm) 3.72 (*s*, 3H, 4-OC$\underline{H_3}$-C_6H_4), 4.11 (*s*, 2H, NHN$\underline{H_2}$), 4.41 (*s*, 2H, NHC$\underline{H_2}$), 6.52 (*d*, 2H, 4-OCH_3-C_6H_4, *J* = 9.0), 6.69 (*d*, 2H, 4-OCH_3-C_6H_4, *J* = 9.0), 7.52-7.54 (*m*, 2H, 4-F-C_6H_4), 7.67-7.71 (*m*, 2H, 4-F-C_6H_4), 8.21 (*s*, 1H, N$\underline{H}$NH_2); **^{13}C NMR** (75.45 MHz, $CDCl_3$) δ (ppm) 38.1 (NH$\underline{C}H_2$), 55.9 (O$\underline{C}H_3$), 113.2 (C4), 114.9, 115.8 (4-O$\underline{C}H_3$-C_6H_4), 116.7 (*d,* 4-F-C_6H_4, $^2J_{C\text{-}F}$ = 22.1 Hz), 123.2 (*d,* 4-F-C_6H_4, $^4J_{C\text{-}F}$ = 3.4 Hz), 130.1 (*d*, 4-F-C_6H_4, $^3J_{C\text{-}F}$ = 8.7 Hz), 141.6, 153.1 (4-O$\underline{C}H_3$-C_6H_4), 156.2 (C3), 160.7 ($\underline{C}$=O), 164.2 (*d,* 4-F-C_6H_4, $^1J_{C\text{-}F}$ = 252.5 Hz), 168.7 (C5); **HRMS** (ESI+): calcd for $C_{18}H_{18}FN_4O_3^+$, $[M+H]^+$: 357.1357, found 357.1367.

3-Carbohydrazide-4-(4-(phenyl)aminomethyl-5-(4-chlorophenyl)isoxazole (**3da**): White solid; 47% yield; mp 202.8-205.6 °C; **^{1}H NMR** (300.06 MHz, DMSO-*d₆*) δ (ppm) 4.30 (*s*, 2H, NHC$\underline{H_2}$), 5.82 (*s*, 1H, N$\underline{H}$CH_2), 6.53-6.60 (*m*, 3H, C_6H_5), 7.06 (*dd*, 2H, C_6H_5, *J* = 7.8, 7.8 Hz), 7.65 (*d*, 2H, 4-Cl-C_6H_4, *J* = 8.7 Hz), 7.82 (*d*, 2H, 4-Cl-C_6H_4, *J* = 8.7 Hz), 10.12 (*s*, 1H, N$\underline{H}$NH_2); **^{13}C NMR** (75.45 MHz, DMSO-*d₆*) δ (ppm) 35.6 (NH$\underline{C}H_2$), 112.7 (C_6H_5), 112.8 (C4), 116.6 (C_6H_5), 125.4, 128.9, 129.0 (4-Cl-C_6H_4), 129.5 (C_6H_5), 135.6 (4-Cl-C_6H_4), 148.3 (C_6H_5), 157.7 (C3), 166.4 (C=O), 166.4 (C5); **HRMS** (ESI+): calcd for $C_{17}H_{16}ClN_4O_2^+$, $[M+H]^+$: 343.0956, found 343.0961.

3-Carbohydrazide-4-[4-(chlorophenyl)aminomethyl]-5-(4-chlorophenyl)isoxazole (**3db**): Gray solid; 83% yield; mp 175.9-177.8 °C; **^{1}H NMR** (500.13 MHz, DMSO-*d₆*) δ (ppm) 4.30 (*d*, 2H, NHC$\underline{H_2}$, *J* = 5.2 Hz), 4.65 (*ls*, 2H, NHN$\underline{H_2}$), 6.07 (*t*, 1H, N$\underline{H}$CH_2, *J* = 4.95 Hz), 6.59 (*d*, 2H, 4-Cl-C_6H_4 - B, *J* = 8.8 Hz), 7.09 (*d*, 2H, 4-Cl-C_6H_4 - B, *J* = 8.8 Hz), 7.65 (*d*, 2H, 4-Cl-C_6H_4 - A, *J* = 8.6 Hz), 7.80 (*d*, 2H, 4-Cl-C_6H_4 - A, *J* = 8.6 Hz), 10.12 (*s*, 1H, N$\underline{H}$NH_2); **^{13}C NMR** (75.45 MHz, , DMSO-*d₆*) δ (ppm) 35.6 (NH$\underline{C}H_2$), 112.5 (C4), 113.9, 119.8, 125.3, 128.6 129.9, 129.5,

135.6, 147.2 (4-Cl-C_6H_4 – A and B), 157.6 (C3), 158.3 (C=O), 166.5 (C5); **HRMS** (ESI+): calcd for $C_{17}H_{15}Cl_2N_4O_2^+$, $[M+H]^+$: 377.0567, found 377.0563.

3-Carbohydrazide-4-[(4-methoxyphenyl)aminomethyl]-5-(4-chlorophenyl)isoxazole (**3dc**): Yellow solid; 52% yield; mp 157.0-158.8 ^oC; **^{1}H NMR** (300.06 MHz, DMSO-d_6) δ (ppm) 3.63 (*s*, 3H, 4-$OC\underline{H}_3$-C_6H_4), 4.25 (*s*, 2H, $NHC\underline{H}_2$), 4.25 (*ls*, 2H, $NHN\underline{H}_2$), 6.55 (*d*, 2H, 4-OCH_3-C_6H_4, *J* = 8.4), 6.70 (*d*, 2H, 4-OCH_3-C_6H_4, *J* = 8.4), 7.65 (*d*, 2H, 4-Cl-C_6H_4, *J* = 8.5), 7.82 (*d*, 2H, 4-Cl-C_6H_4, *J* = 8.5); **^{13}C NMR** (75.45 MHz, DMSO-d_6) δ (ppm) 36.5 ($NH\underline{C}H_2$), 55.3 (4-$O\underline{C}H_3$-C_6H_4), 113.0 (C4), 114.0, 114.5 (4-$O\underline{C}H_3$-C_6H_4), 125.4, 129.0, 129.4, 135.5 (4-Cl-C_6H_4), 142.3, 151.5 (4-$O\underline{C}H_3$-C_6H_4), 158.1 (C3), 161.1 ($\underline{C}$=O), 166.4 (C5); **HRMS** (ESI+): calcd for $C_{18}H_{18}ClN_4O_3^+$, $[M+H]^+$: 373.1062, found 373.1073.

Synthesis of 3-[(2*E*)-N´-(Benzylidene)hydrazinecarbonyl]-4-[(aryl)aminomethyl]-5-arylisoxazoles 4(aa-ac)-4(da-dc)

General method. Compound **3** (1.0 mmol, 1.0 equiv) was solubilized in DMSO (2 mL), and benzaldehyde (0.106 g, 1.0 mmol, 1.0 equiv) and two drops of hydrochloric acid (37%) were added. The mixture was stirred at room temperature for 2 h. Then, cold distilled water (50 mL) was added, and the product was filtered under a vacuum and washed with cold distilled water. The solid was dried under a vacuum.

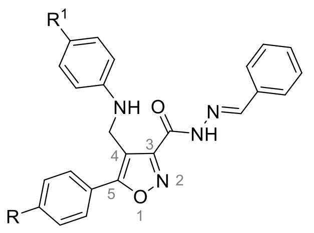

4(aa-ac)-4(da-dc)

3-[(2*E*)-*N´*-(Benzylidene)hydrazinecarbonyl]-4-(phenyl)aminomethyl-5-(4-nitrophenyl)isoxazole (**4aa**): Yellow solid; 88% yield; mp 92.6-94.4 ^oC; **^{1}H NMR** (300.06 MHz, DMSO-d_6) δ (ppm) 4.46 (*s*, 2H, $NHC\underline{H}_2$), 5.95 (*ls*, 1H, $N\underline{H}CH_2$), 6.58-6.61 (*m*, 3H, C_6H_5 - A), 7.43-7.09 (*m*, 2H, C_6H_5 - A), 7.45 (*ddd*, 1H, C_6H_5 – B, *J* = 7.3, 4.9, 1.3 Hz), 7.89 (*ddd*, 1H, C_6H_5 – B, *J* = 7.7, 7.7, 1.5 Hz), 7.96-7.99 (*m*, 1H, C_6H_5 – B), 8.11 (*d*, 2H, 4-NO_2-C_6H_4, *J* = 9.0 Hz), 8.42 (*d*, 2H, 4-NO_2-C_6H_4, *J* = 9.0 Hz), 8.54 (*s*, 1H, $NC\underline{H}$), 8.63 (*d*, 1H, C_6H_5 – B), 12.68 (*s*, 1H, $N\underline{H}N$); **^{13}C NMR** (75.45 MHz, DMSO-d_6) δ (ppm) 35.6 ($NH\underline{C}H_2$), 112.7 (C_6H_5), 115.4 (C4), 116.7 (C_6H_5), 124.4 (4-NO_2-C_6H_4), 127.3, 128.6, 128.9 (C_6H_5 – A and B), 128.9 (4-NO_2-C_6H_4), 130.5 (C_6H_5 – A or B), 132.1 (4-NO_2-C_6H_4), 133.9, 148.2 (C_6H_5 – A and B), 148.4 (4-NO_2-C_6H_4), 149.8 (C=N), 155.3 (C3), 157.4 (C=O), 165.8 (C5); **HRMS** (ESI+): calcd for $C_{24}H_{20}N_5O_4^+$, $[M+H]^+$: 442.1510, found 442.1515.

3-[(2*E*)-*N´*-(Benzylidene)hydrazinecarbonyl]-4-[(4-chlorophenyl)aminomethyl]-5-(4-nitrophenyl)isoxazole (**4ab**): Orange solid; 95% yield; mp 177.6-179.0 ^oC; **^{1}H NMR** (300.06 MHz, DMSO-d_6) δ (ppm) 4.44 (*s*, 2H, $NHC\underline{H}_2$), 6.20 (*ls*, 1H, $N\underline{H}CH_2$), 6.59 (*d*, 2H, 4-Cl-C_6H_4, *J* = 8.9 Hz), 7.09 (*d*, 2H, 4-Cl-C_6H_4, *J* = 8.9 Hz), 7.46-7.48 (*m*, 3H, C_6H_5), 7.72-7.74 (*m*, 2H, C_6H_5), 8.10 (*d*, 2H, 4-NO_2-C_6H_4, *J* = 8.9 Hz), 8.42 (*d*, 2H, 4-NO_2-C_6H_4, *J* = 8.9 Hz), 8.50 (*s*, 1H, $NC\underline{H}$), 12.44 (*s*, 1H, $N\underline{H}N$); **^{13}C NMR** (75.45 MHz, DMSO-d_6) δ (ppm) 36.5 ($NH\underline{C}H_2$), 112.7 (4-Cl-C_6H_4), 115.4 (C4), 116.7 (4-Cl-C_6H_4), 124.3 (4-NO_2-C_6H_4), 127.3 (C_6H_5), 128.6 (4-Cl-C_6H_4), 128.8 (C_6H_5), 128.9 (4-NO_2-C_6H_4), 130.5 (C_6H_5), 132.1 (4-NO_2-C_6H_4), 133.9 (C_6H_5), 148.2 (4-Cl-C_6H_4), 148.4 (4-NO_2-C_6H_4), 149.8 (C=N), 155.5 (C3), 157,4 (C=O), 165.8 (C5); **HRMS** (ESI+): calcd for $C_{24}H_{19}ClN_5O_4^+$, $[M+H-Cl]^+$: 476.1120, found 476.1117.

3-[(2*E*)-*N´*-(Benzylidene)hydrazinecarbonyl]-4-[(4-methoxyphenyl)aminomethyl]-5-(4-nitrophenyl)isoxazole (**4ac**): Beige solid; 88% yield; mp 90.2-93.5 ^oC; **^{1}H NMR** (300.06 MHz, DMSO-d_6) δ (ppm) 3.63 (*s*, 3H, 4-$OC\underline{H}_3$-C_6H_4), 4.43 (*s*, 2H, $NHC\underline{H}_2$), 5.72 (*ls*, 1H, $N\underline{H}CH_2$), 6.58-6.62 (*m*, 2H, 4-OCH_3-C_6H_4), 6.71 (*d*, 2H, 4-OCH_3-C_6H_4, *J* = 8.9 Hz), 7.46-7.48 (*m*, 3H, C_6H_5), 7.71-7.74 (*m*, 2H, C_6H_5), 8.12 (*d*, 2H, 4-NO_2-C_6H_4, *J* = 8.9 Hz), 8.41 (*d*, 2H, 4-NO_2-C_6H_4, *J* = 8.9 Hz), 8.49 (*s*, 1H, $NC\underline{H}$), 12.46 (*s*, 1H, $N\underline{H}N$); **^{13}C NMR** (75.45 MHz, DMSO-d_6) δ (ppm) 36.8 ($NH\underline{C}H_2$), 55.3 (4-$O\underline{C}H_3$-C_6H_4), 114.5 (4-OCH_3-C_6H_4), 114.6 (C4), 114.9 (4-OCH_3-C_6H_4), 124.4 (4-NO_2-C_6H_4), 127.3, 128.7 (C_6H_5), 128.8 (4-NO_2-C_6H_4), 129.0, 130.6 (C_6H_5), 132.1 (4-NO_2-C_6H_4),

134.6 (4-OCH_3-C_6H_4), 148.4 (4-NO_2-C_6H_4), 149.9 (C=N), 155.6 (C3), 157.5 (4-OCH_3-C_6H_4), 158.4 (C=O), 165.9 (C5); **HRMS** (ESI+): calcd for $C_{25}H_{22}N_5O_5^+$, $[M+H]^+$: 472.1615, found 472.1616.

3-[(2*E*)-*N′*-(Benzylidene)hydrazinecarbonyl]-4-(phenyl)aminomethyl-5-(phenyl)isoxazole (**4ba**): White solid; 90% yield; mp 169.9-172.0 ^oC; **^1H NMR** (300.06 MHz DMSO-d_6) δ (ppm) 4.36 (*d*, 2H, NHC$\underline{H_2}$, *J* = 4.5 Hz), 5.94 (*s,* 1H, N$\underline{H}$$CH_2$), 6.55-6.60 (*m*, 2H, C_6H_5 - B), 7.03-7.08 (*m*, 2H, C_6H_5 - B), 7.45-7.47 (*m*, 3H, C_6H_5 - A), 7.59-7.61 (*m*, 3H, C_6H_5 - C), 7.71-7.73 (*m*, 2H, C_6H_5 - A), 7.82-7.85 (*m*, 2H, C_6H_5 - C), 8.48 (*s*, 1H, NC$\underline{H}$), 12.41 (*s,* 1H, N$\underline{H}$N); **^{13}C NMR** (75.45 MHz, DMSO-d_6) δ (ppm) 35.6 (NH$\underline{C}H_2$), 112.6 (C_6H_5), 112.9 (C4), 116.5, 126.4, 127.3, 127.3, 128.8, 129.9, 129.4, 130.5, 130.9, 133.9, 148.3 (C_6H_5), 149.6 (C=N), 155.8 (C3), 157.2 (C=O), 168.1 (C5); **HRMS** (ESI+): calcd for $C_{24}H_{21}N_4O_2^+$, $[M+H]^+$: 397.1659, found 397.1665.

3-[(2*E*)-*N′*-(Benzylidene)hydrazinecarbonyl]-4-[(4-chlorophenyl)aminomethyl]-5-(phenyl)isoxazole (**4bb**): White solid; 89% yield; mp 188.4-190.1 ^oC; **^1H NMR** (300.06 MHz, DMSO-d_6) δ (ppm) 4.35 (*d*, 2H, NHC$\underline{H_2}$, *J* = 4.9 Hz), 6.18 (*t,* 1H, N$\underline{H}$$CH_2$, J = 4.94 Hz), 6.60 (*d*, 2H, 4-Cl-C_6H_4, *J* = 8.8 Hz), 7.08 (*d*, 2H, 4-Cl-C_6H_4, *J* = 8.8 Hz), 7.46-7.48 (*m*, 3H, C_6H_5 - A), 7.59-7.61 (*m*, 3H, C_6H_5 - B), 7.71-7.74 (*m*, 2H, C_6H_5 - A), 7.80-7.83 (*m*, 2H, C_6H_5 - B), 8.50 (*s*, 1H, NC$\underline{H}$), 12.42 (*s,* 1H, N$\underline{H}$N); **^{13}C NMR** (75.45 MHz, DMSO-d_6) δ (ppm) 35.7 (NH$\underline{C}H_2$), 112.7 (C4), 113.9, 119.8 (4-Cl-C_6H_4), 126.4, 127.4 (C_6H_5), 128.6 (4-Cl-C_6H_4), 129.0, 129.4, 130.6, 131.0, 134.0, 148.3 (C_6H_5), 147.3 (4-Cl-C_6H_4), 149.6 (C=N), 155.8 (C3), 157.2 (C=O), 168.2 (C5); **HRMS** (ESI+): calcd for $C_{24}H_{20}ClN_4O_2^+$, $[M+H]^+$: 431.1269, found 431.1262.

3-[(2*E*)-*N′*-(Benzylidene)hydrazinecarbonyl]-4-[(4-methoxyphenyl)aminomethyl]-5-(phenyl)isoxazole (**4bc**): White solid; 91% yield; mp 197.6-199.6 ^oC; **^1H NMR** (300.06 MHz, DMSO-d_6) δ (ppm) 3.63 (*s*, 3H, 4-OC$\underline{H_3}$-C_6H_4), 4.31 (*d*, 2H, NHC$\underline{H_2}$, *J* = 5.5 Hz), 5.53 (*t,* 1H, N$\underline{H}$$CH_2$, J = 5.5 Hz), 6.57 (*d*, 2H, 4-OCH_3-C_6H_4, *J* = 8.9 Hz), 6.70 (*d*, 2H, 4-Cl-C_6H_4, *J* = 8.8 Hz), 7.46-7.48 (*m*, 3H, C_6H_5 - A), 7.59-7.61 (*m*, 3H, C_6H_5 - B), 7.71-7.74 (*m*, 2H, C_6H_5 - A), 7.80-7.83 (*m*, 2H, C_6H_5 - B), 8.46 (*s*, 1H, NC$\underline{H}$), 12.41 (*s,* 1H, N$\underline{H}$N); **^{13}C NMR** (75.45 MHz, DMSO-d_6) δ (ppm) 35.5 (NH$\underline{C}H_2$), 55.3 (4-O$\underline{C}H_3$-C_6H_4), 113.2 (C4), 114.0, 114.6 (4-OCH_3-C_6H_4), 126.6, 127.4, 129.0, 129.4, 130.6, 130.9 (C_6H_5 – A and B), 134.0 (4-O$\underline{C}H_3$-C_6H_4), 149.6 (C=N), 151.5 (4-OCH_3-C_6H_4), 155.9 (C3), 157.4 (C=O), 168.1 (C5); **HRMS** (ESI+): calcd for $C_{25}H_{23}N_4O_3^+$, $[M+H]^+$: 427.1765, found 427.1787.

3-[(2*E*)-*N′*-(Benzylidene)hydrazinecarbonyl]-4-(phenyl)aminomethyl-5-(4-fluorophenyl)isoxazole (**4ca**): Yellow solid; 90% yield; mp 157.7-159.6 ^oC; **^1H NMR** (300.06 MHz, DMSO-d_6) δ (ppm) 4.29-4.36 (*m*, 2H, NHC$\underline{H_2}$), 5.82-5.90 (*m*, 1H, N$\underline{H}$$CH_2$), 6.56-6.61 (*m*, 2H, C_6H_5 - A), 7.04-7.09 (*m*, 2H, C_6H_5 - A), 7.43-7.49 (*m*, 5H, C_6H_5 - B), 7.70-7.73 (*m*, 2H, 4-F-C_6H_4), 7.87-7.92 (*m*, 2H, 4-F-C_6H_4), 8.49 (*s*, 1H, NC$\underline{H}$), 12.46 (*s,* 1H, N$\underline{H}$N); **^{13}C NMR** (75.45 MHz, DMSO-d_6) δ (ppm) 35.6 (NH$\underline{C}H_2$), 112.7 (C_6H_5 – A), 112.9 (C4), 116.6 (*d*, 4-F-C_6H_4, $^2J_{C-F}$ = 22.1 Hz), 116.6 (C_6H_5 - A), 123.1 (*d,* 4-F-C_6H_4, $^4J_{C-F}$ = 3.3 Hz), 127.3, 128.9, 128.9 (C_6H_5 – A and B), 129.9 (*d*, 4-F-C_6H_4, $^3J_{C-F}$ = 9.0 Hz), 130.5, 133.9, 148.3 (C_6H_5 – A and B), 149.6 (C=N), 155.8 (C3), 157.3 ($\underline{C}$=O), 163.4 (*d,* 4-F-C_6H_4, $^1J_{C-F}$ = 249.5 Hz), 167.3 (C5); **HRMS** (ESI+): calcd for $C_{24}H_{20}FN_4O_2^+$, $[M+H]^+$: 415.1565, found 415.1579.

3-[(2*E*)-*N′*-(Benzylidene)hydrazinecarbonyl]-4-[(4-chlorophenyl)aminomethyl]-5-(4-fluorophenyl)isoxazole (**4cb**): White solid; 95% yield; mp 167.9-170.5 ^oC; **^1H NMR** (300.06 MHz, DMSO-d_6) δ (ppm) 4.35 (*s*, 2H, NHC$\underline{H_2}$), 6.14 (*ls*, 1H, N$\underline{H}$$CH_2$), 6.60 (*d*, 2H, 4-Cl-$C_6H_4$, *J* = 8.9 Hz), 7.08 (*m*, 2H, 4-Cl-C_6H_4, *J* = 8.9 Hz), 7.43-7.49 (*m*, 5H, C_6H_5), 7.71-7.74 (*m*, 2H, 4-F-C_6H_4), 7.84-7.89 (*m*, 2H, 4-F-C_6H_4), 8.50 (*s*, 1H, NC$\underline{H}$), 12.38 (*s,* 1H, N$\underline{H}$N); **^{13}C NMR** (75.45 MHz, DMSO-d_6) δ (ppm) 35.7 (NH$\underline{C}H_2$), 112.6 (C4), 113.9 (4-Cl-C_6H_4), 116.6 (*d,* 4-F-C_6H_4, $^2J_{C-F}$ = 22.2 Hz), 119.9 (4-Cl-C_6H_4), 123.0 (*d,* 4-F-C_6H_4, $^4J_{C-F}$ = 3.3 Hz), 127.3(C_6H_5), 128.6 (4-Cl-C_6H_4), 128.9 (C_6H_5), 129.9 (*d*, 4-F-C_6H_4, $^3J_{C-F}$ = 9.0 Hz), 130.5, 133.9 (C_6H_5), 147.2 (4-Cl-C_6H_4), 149.7 (C=N), 155.7 (C3), 157.1 ($\underline{C}$=O), 163.4 (*d,* 4-F-C_6H_4, $^1J_{C-F}$ = 249.7 Hz), 167.3 (C5); **HRMS** (ESI+): calcd for $C_{24}H_{19}ClFN_4O_2^+$, $[M+H]^+$: 449.1175, found 449.1179.

3-[(2*E*)-*N′*-(Benzylidene)hydrazinecarbonyl]-4-[(4-methoxyphenyl)aminomethyl]-5-(4-fluorophenyl)isoxazole (**4cc**): Beige solid; 90% yield; mp 181.7-185.5 ^oC; **^1H NMR** (300.06 MHz, DMSO-d_6) δ (ppm) 3.60 (*s*, 3H, 4-OC$\underline{H_3}$-C_6H_4), 4.30 (*d*, 2H, NHC$\underline{H_2}$, *J* = 5.5 Hz), 5.50 (*t*, 1H, N$\underline{H}$$CH_2$, *J* = 5.5 Hz), 6.57 (*d*, 2H, 4-OCH_3-C_6H_4, *J* = 9.0), 6.71 (*d*, 2H, 4-OCH_3-C_6H_4, *J* = 9.0), 7.43-7.49 (*m*, 5H, C_6H_5), 7.70-7.73 (*m*, 2H, 4-F-C_6H_4), 7.88-7.92 (*m*, 2H, 4-F-C_6H_4), 8.47 (*s*, 1H, NC$\underline{H}$), 12.38 (*s,* 1H, N$\underline{H}$N); **^{13}C NMR** (75.45 MHz, DMSO-d_6) δ (ppm) 36.5 (NH$\underline{C}H_2$), 55.3 (O$\underline{C}H_3$), 113.1 (C4), 114.0, 114.5 (4-O$\underline{C}H_3$-C_6H_4), 116.6 (*d,* 4-F-C_6H_4, $^2J_{C-F}$ = 22.1 Hz), 123.1 (*d,* 4-F-C_6H_4, $^4J_{C-F}$ = 3.1 Hz), 127.3, 128.9 (C_6H_5), 129.9 (*d*, 4-F-C_6H_4, $^3J_{C-F}$ = 8.7 Hz), 130.5, 133.9 (C_6H_5), 142.4 (4-O$\underline{C}H_3$-C_6H_4), 149.6 (C=N), 151.5 (4-O$\underline{C}H_3$-C_6H_4), 155.8 (C3), 157.4 ($\underline{C}$=O), 163.4 (*d,* 4-F-C_6H_4, $^1J_{C-F}$ = 249.4 Hz), 167.2 (C5); **HRMS** (ESI+): calcd for $C_{25}H_{22}FN_4O_3^+$, $[M+H]^+$: 445.1670, found 445.1680.

3-[(2*E*)-*N*′-(Benzylidene)hydrazinecarbonyl]-4-(4-(phenyl)aminomethyl-5-(4-chlorophenyl)isoxazole (**4da**): White solid; 90% yield; mp 189.3-191.8 °C; **^{1}H NMR** (300.06 MHz, DMSO-*d_6*) δ (ppm) 4.37 (*s*, 2H, $NHC\underline{H_2}$), 5.88 (*s*, 1H, $N\underline{H}CH_2$), 6.55-6.60 (*m*, 3H, C_6H_5 - A), 7.03-7.08 (*m*, 2H, C_6H_5 - A), 7.46 (*ls*, 3H, 4-Cl-C_6H_4 and C_6H_5 - B), 7.66-7.69 (*m*, 4H, 4-Cl-C_6H_4 and C_6H_5 - B), 7.84 (*d*, 2H, 4-Cl-C_6H_4, J = 8.3 Hz), 8.47 (*s*, 1H, $NC\underline{H}$), 12.38 (*s,* 1H, $N\underline{H}N$); **^{13}C NMR** (75.45 MHz, DMSO-*d_6*) δ (ppm) 35.4 ($NH\underline{C}H_2$), 112.5 (C_6H_5 - A), 113.3 (C4), 116.5 (C_6H_5 - A), 125.1, 128.7, 128.9 (4-Cl-C_6H_4), 129.4, 130.4, 133.8 (C_6H_5 - B), 135.6 (4-Cl-C_6H_4), 135.6, 148.1 (C_6H_5 – A and B), 149.5 (C=N), 155.5 (C3), 157.2 (C=O), 166.8 (C5); **HRMS** (ESI+): calcd for $C_{24}H_{20}ClN_4O_2^+$, $[M+H]^+$: 431.1269, found 431.1277.

3-[(2*E*)-*N*′-(Benzylidene)hydrazinecarbonyl]-4-[4-(chlorophenyl)aminomethyl]-5-(4-chlorophenyl)isoxazole (**4db**): Gray solid; 95% yield; mp 167.5-169.3 °C; **^{1}H NMR** (300.06 MHz, DMSO-*d_6*) δ (ppm) 4.28-4.37 (*m*, 2H, $NHC\underline{H_2}$), 6.09-6.17 (*m*, 1H, $N\underline{H}CH_2$), 6.57-6.61 (*m*, 2H, 4-Cl-C_6H_4 - B), 7.09 (*d*, 2H, 4-Cl-C_6H_4 - B, *J* = 8.8 Hz), 7.46-7.48 (*m*, 3H, 4-Cl-C_6H_4 - A), 7.64-7.73 (*m*, 4H, 4-Cl-C_6H_4 and C_6H_5), 7.78-7.84 (*m*, 2H, 4-Cl-C_6H_4 and C_6H_5), 8.49 (*s*, 1H, $NC\underline{H}$), 12.42 (*s,* 1H, $N\underline{H}N$); **^{13}C NMR** (75.45 MHz, , DMSO-*d_6*) δ (ppm) 35.6 ($NH\underline{C}H_2$), 113.2 (C4), 113.9, 119.9, 125.2 (4-Cl-C_6H_4 – A and B), 127.4 (C_6H_5), 128.6 (4-Cl-C_6H_4 – A or B), 128.9 (C_6H_5), 129.1, 129.5 (4-Cl-C_6H_4 – A and B), 130.6, 133.9 (C_6H_5), 135.8, 147.2 (4-Cl-C_6H_4 – A and B), 149.5 (C=N), 155.7 (C3), 157.2 (C=O), 167.1 (C5); **HRMS** (ESI+): calcd for $C_{24}H_{19}Cl_2N_4O_2^+$, $[M+H]^+$: 465.0880, found 465.0898.

3-[(2*E*)-*N*′-(Benzylidene)hydrazinecarbonyl]-4-[(4-methoxyphenyl)aminomethyl]-5-(4-chlorophenyl)isoxazole (**4dc**): Light Yellow solid; 90% yield; mp 176.3-177.1 °C; **^{1}H NMR** (300.06 MHz, DMSO-*d_6*) δ (ppm) 3.63 (*s*, 3H, 4-$OC\underline{H_3}$-C_6H_4), 4.32 (*s*, 2H, $NHC\underline{H_2}$), 5.50 (*ls*, 1H, $N\underline{H}CH_2$), 6.57 (*d*, 2H, 4-OCH_3-C_6H_4, *J* = 9.0 Hz), 6.71 (*d*, 2H, 4-OCH_3-C_6H_4, *J* = 9.0 Hz), 7.45-7.48 (*m*, 3H, 4-Cl-C_6H_4 and C_6H_5), 7.66-7.73 (*m*, 4H, 4-Cl-C_6H_4 and C_6H_5), 7.86 (*d*, 2H, 4-Cl-C_6H_4, *J* = 8.7), 8.47 (*s*, 1H, $NC\underline{H}$), 12.39 (*s,* 1H, $N\underline{H}N$); **^{13}C NMR** (75.45 MHz, DMSO-*d_6*) δ (ppm) 36.5 ($NH\underline{C}H_2$), 55.3 (4-$O\underline{C}H_3$-C_6H_4), 113.7 (C4), 114.1, 114.5 (4-$O\underline{C}H_3$-C_6H_4), 125.3 (4-Cl-C_6H_4), 127.3, 128.9 (C_6H_5), 129.1, 129.5 (4-Cl-C_6H_4), 130.5, 133.9 (C_6H_5), 135.7 (4-Cl-C_6H_4), 142.4 (4-$O\underline{C}H_3$-C_6H_4), 149.5 (C=N), 151.5 (4-$O\underline{C}H_3$-C_6H_4), 155.7 (C3), 157.4 ($\underline{C}$=O), 166.9 (C5); **HRMS** (ESI+): calcd for $C_{25}H_{22}ClN_4O_3^+$, $[M+H]^+$: 461.1375, found 461.1381.

Synthesis of 3-[(2*E*)-*N*′-(2-pyridinylmethylene)hydrazinecarbonyl]-4-[(aryl)aminomethyl]-5-arylisoxazoles 5(aa-ac)-5(da-dc)

General method. The compound **3** (1.0 mmol, 1.0 equiv) was solubilized in DMSO (2 mL), and 2-formylpyridine (0.107 g, 1.0 mmol, 1.0 equiv) and two drops of hydrochloric acid (37%) were added. The mixture was stirred at room temperature for 2 h. Then, cold distilled water (50 mL) was added, and the product was filtered and dried under a vacuum.

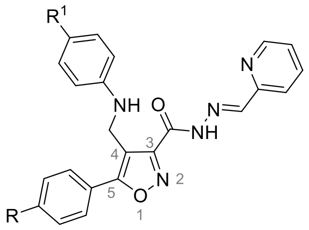

5(aa-ac)-5(da-dc)

3-[(2*E*)-*N*′-(2-pyridinylmethylene)hydrazinecarbonyl]-4-(phenyl)aminomethyl-5-(4-nitrophenyl)isoxazole (**5aa**): Orange solid; 92% yield; mp 223.8-225.5 °C; **^{1}H NMR** (300.06 MHz, DMSO-*d_6*) δ (ppm) 4.46 (*s*, 2H, $NHC\underline{H_2}$), 5.95 (*ls,* 1H, $N\underline{H}CH_2$), 6.58-6.61 (*m*, 3H, C_6H_5), 7.04-7.09 (*m*, 2H, C_6H_5), 7.45 (*ddd*, 1H, 2-C_5H_4N, *J* = 7.3, 4.9, 1.3 Hz), 7.89 (*ddd*, 1H, 2-C_5H_4N, *J* = 7.7, 7.7, 1.5 Hz), 7.96-7.99 (*m,* 1H, 2-C_5H_4N), 8.11 (*d*, 2H, 4-NO_2-C_6H_4, *J* = 9.0 Hz), 8.42 (*d*, 2H, 4-NO_2-C_6H_4, *J* = 9.0 Hz), 8.54 (*s*, 1H, $NC\underline{H}$), 8.63 (*d*, 1H, 2-C_5H_4N, *J* = 4.4 Hz), 12.68 (*s,* 1H, $N\underline{H}N$); **^{13}C NMR** (75.45 MHz, DMSO-*d_6*) δ (ppm) 35.6 ($NH\underline{C}H_2$), 112.7 (C_6H_5), 115.6 (C4), 116.8 (C_6H_5), 120.1 (2-C_5H_4N), 124.5(4-NO_2-C_6H_4), 124.8 (2-C_5H_4N), 128.7 (4-NO_2-C_6H_4), 128.9 (C_6H_5), 132.0 (4-NO_2-C_6H_4), 137.0 (2-C_5H_4N), 148.2 (4-NO_2-C_6H_4), 148.4 (C_6H_5), 149.6 (C=N), 150.0, 152.9 (2-C_5H_4N), 155.8 (C3), 157.2 (C=O), 165.9 (C5); **HRMS** (ESI+): calcd for $C_{23}H_{19}N_6O_4^+$, $[M+H]^+$: 443.1462, found 443.1493.

3-[(2*E*)-*N*´-(2-pyridinylmethylene)hydrazinecarbonyl]-4-[(4-chlorophenyl)aminomethyl]-5-(4-nitrophenyl)isoxazole (5ab): Orange solid; 90% yield; mp 214.2-218.3 °C; **^{1}H NMR** (300.06 MHz, DMSO-d_6) δ (ppm) 4.29-4.46 (*m*, 2H, NHC<u>H</u>$_2$), 5.95 (*ls,* 1H, N<u>H</u>CH$_2$), 6.56-6.61 (*m*, 2H, 4-Cl-C_6H_4), 7.04-7.09 (*m*, 2H, 4-Cl-C_6H_4), 7.45 (*ddd*, 1H, 2-C_5H_4N, *J* = 7.4, 4.8, 1.4 Hz), 7.89 (*ddd*, 1H, 2-C_5H_4N, *J* = 7.7, 7.7, 1.7 Hz), 7.96-7.99 (*m,* 1H, 2-C_5H_4N), 8.11 (*d*, 2H, 4-NO_2-C_6H_4, *J* = 8.9 Hz), 8.42 (*d*, 2H, 4-NO_2-C_6H_4, *J* = 8.9 Hz), 8.54 (*s*, 1H, NC<u>H</u>), 8.63 (*ddd*, 1H, 2-C_5H_4N, *J* = 4.7, 1.4, 1.4 Hz), 12.68 (*s,* 1H, N<u>H</u>N); **^{13}C NMR** (75.45 MHz, DMSO-d_6) δ (ppm) 35.6 (NH<u>C</u>H$_2$), 112.7 (4-Cl-C_6H_4), 115.6 (C4), 116.8 (4-Cl-C_6H_4), 120.1 (2-C_5H_4N), 124.5 (4-NO_2-C_6H_4), 124.8 (2-C_5H_4N), 128.7 (4-Cl-C_6H_4), 128.9, 132.0 (4-NO_2-C_6H_4), 137.0 (2-C_5H_4N), 148.2 (4-Cl-C_6H_4), 148.4 (4-NO_2-C_6H_4), 149.6 (C=N), 150.0, 152.9 (2-C_5H_4N), 155.8 (C3), 157,2 (C=O), 165.9 (C5); **HRMS** (ESI+): calcd for $C_{23}H_{18}ClN_6O_4^+$, $[M+H-Cl]^+$: 477.1073, found 477.1074.

3-[(2*E*)-*N*´-(2-pyridinylmethylene)hydrazinecarbonyl]-4-[(4-methoxyphenyl)aminomethyl]-5-(4-nitrophenyl)isoxazole (5ac): Brown solid; 91% yield; mp 148.1-150.3 °C; **^{1}H NMR** (300.06 MHz, DMSO-d_6) δ (ppm) 3.63 (*s*, 3H, 4-OC<u>H</u>$_3$-C_6H_4), 4.41 (*s*, 2H, NHC<u>H</u>$_2$), 5.54 (*ls,* 1H, N<u>H</u>CH$_2$), 6.56 (*d*, 2H, 4-OCH_3-C_6H_4, *J* = 8.9 Hz), 6.70 (*d*, 2H, 4-OCH_3-C_6H_4, *J* = 8.9 Hz), 7.45 (*ddd*, 1H, 2-C_5H_4N, *J* = 7.4, 4.8, 1.3 Hz), 7.90 (*ddd*, 1H, 2-C_5H_4N, *J* = 7.5, 7.5, 1.3 Hz), 7.98 (*d,* 1H, 2-C_5H_4N, J = 7.9 Hz), 8.12 (*d*, 2H, 4-NO_2-C_6H_4, *J* = 9.0 Hz), 8.42 (*d*, 2H, 4-NO_2-C_6H_4, *J* = 9.0 Hz), 8.52 (*s*, 1H, NC<u>H</u>), 8.63 (*d*, 1H, 2-C_5H_4N, *J* = 4.5 Hz), 12.68 (*s,* 1H, N<u>H</u>N); **^{13}C NMR** (75.45 MHz, DMSO-d_6) δ (ppm) 36.4 (NH<u>C</u>H$_2$), 55.3 (4-O<u>C</u>H$_3$-C_6H_4), 114.1, 114.5 (4-OCH_3-C_6H_4), 115.8 (C4), 120.1 (2-C_5H_4N), 124.4 (4-NO_2-C_6H_4), 124.8 (2-C_5H_4N), 128.7, 132.1 (4-NO_2-C_6H_4), 137.0 (2-C_5H_4N), 142.3 (4-OCH_3-C_6H_4), 148.4 (4-NO_2-C_6H_4), 149.6 (C=N), 150.0 (2-C_5H_4N), 151.5 (4-OCH_3-C_6H_4), 152.9 (2-C_5H_4N), 155.8 (C3), 157.3 (C=O), 165.8 (C5); **HRMS** (ESI+): calcd for $C_{24}H_{21}N_6O_5^+$, $[M+H]^+$: 473.1568, found 473.1569.

3-[(2*E*)-*N*´-(2-pyridinylmethylene)hydrazinecarbonyl]-4-(phenyl)aminomethyl-5-(phenyl)isoxazole (5ba): Light Yellow solid; 87% yield; mp 194.8-195.6 °C; **^{1}H NMR** (300.06 MHz, DMSO-d_6) δ (ppm) 4.37 (*d*, 2H, NHC<u>H</u>$_2$, *J* = 3.7 Hz), 5.94 (*s*, 1H, N<u>H</u>CH$_2$), 6.55-6.61 (*m*, 3H, C_6H_5 – B), 7.06 (*dd*, 2H, C_6H_5 – B, J = 8.7, 7.1 Hz), 7.45 (*ddd*, 1H, 2-C_5H_4N, *J* = 7.4, 4.9, 1.4 Hz), 7.59-7.62 (*m*, 3H, 2-C_5H_4N and C_6H_5), 7.82-7.98 (*m*, 4H, 2-C_5H_4N and C_6H_5), 8.53 (*s*, 1H, NC<u>H</u>), 8.63 (*ddd*, 1H, 2-C_5H_4N, *J* = 4.8, 1.7, 1.0 Hz), 12.66 (*s*, 1H, N<u>H</u>N); **^{13}C NMR** (75.45 MHz, DMSO-d_6) δ (ppm) 35.6 (NH<u>C</u>H$_2$), 112.6 (C_6H_5 – B), 113.1 (C4), 116.6 (C_6H_5 – B), 120.1 (2-C_5H_4N), 124.8 (2-C_5H_4N), 126.4, 127.3, 128.9, 129.4, 131.0 (C_6H_5 – A and B), 137.0 (2-C_5H_4N), 148.4 (C_6H_5 – B), 149.6 (C=N), 149.8, 152.9 (2-C_5H_4N), 156.1 (C3), 157.1 (C=O), 168.3 (C5); **HRMS** (ESI+): calcd for $C_{23}H_{20}N_5O_2^+$, $[M+H]^+$: 398.1612, found 398.1628.

3-[(2*E*)-*N*´-(2-pyridinylmethylene)hydrazinecarbonyl]-[(4-chlorophenyl)aminomethyl]-5-(phenyl)isoxazole (5bb): White solid; 88% yield; mp 209.3-211.7 °C; **^{1}H NMR** (300.06 MHz, DMSO-d_6) δ (ppm) 4.36 (*d*, 2H, NHC<u>H</u>$_2$, *J* = 4.9 Hz), 6.18 (*t*, 1H, N<u>H</u>CH$_2$, *J* = 4.9 Hz), 6.60 (*d*, 2H, 4-Cl-C_6H_4, *J* = 8.8 Hz), 7.09 (*d*, 2H, 4-Cl-C_6H_4, *J* = 8.7 Hz), 7.45 (*ddd*, 1H, 2-C_5H_4N, *J* = 7.3, 4.8, 1.4 Hz), 7.59-7.62 (*m*, 3H, C_6H_5), 7.80-7.83 (*m*, 2H, C_6H_5), 7.90 (*ddd,* 1H, 2-C_5H_4N, *J* = 7.8, 7.6, 1.7 Hz), 7.96-7.98 (*m,* 1H, 2-C_5H_4N), 8.53 (*s*, 1H, NC<u>H</u>), 8.63 (*d*, 1H, 2-C_5H_4N, *J* = 4.8 Hz), 12.66 (*s*, 1H, N<u>H</u>N); **^{13}C NMR** (75.45 MHz, DMSO-d_6) δ (ppm) 35.7 (NH<u>C</u>H$_2$), 112.8 (C4), 113.9 (C_6H_5), 119.8 (4-Cl-C_6H_4), 120.1, 124.8 (2-C_5H_4N), 126.3, 127.3 (4-Cl-C_6H_4), 128.6, 129.5, 131.0 (C_6H_5), 137.0 (2-C_5H_4N), 147.3 (4-Cl-C_6H_4), 149.7 (C=N), 149.8, 152.9 (2-C_5H_4N), 156.0 (C3), 157.0 (C=O), 168.4 (C5); **HRMS** (ESI+): calcd for $C_{23}H_{19}ClN_5O_2^+$, $[M+H]^+$: 432.1222, found 432.1235.

3-[(2*E*)-*N*´-(2-pyridinylmethylene)hydrazinecarbonyl]-[(4-methoxyphenyl)aminomethyl]-5-(phenyl)isoxazole (5bc): White solid; 90% yield; mp 175.4-180.5 °C; **^{1}H NMR** (300.06 MHz, DMSO-d_6) δ (ppm) 3.63 (*s*, 3H, 4-OC<u>H</u>$_3$-C_6H_4), 4.32 (*d*, 2H, NHC<u>H</u>$_2$, *J* = 5.3 Hz), 5.53 (*t*, 1H, N<u>H</u>CH$_2$, *J* = 5.5 Hz), 6.57 (*d*, 2H, 4-OCH_3-C_6H_4, *J* = 9.0 Hz), 6.70 (*d*, 2H, 4-OCH_3-C_6H_4, *J* = 9.0 Hz), 7.44 (*ddd*, 1H, 2-C_5H_4N, *J* = 7.3, 4.8, 1.4 Hz), 7.59-7.61 (*m*, 3H, C_6H_5), 7.82-7.92 (*m*, 3H, C_6H_5 and 2-C_5H_4N), 7.96-7.99 (*m,* 1H, 2-C_5H_4N), 8.51 (*s*, 1H, NC<u>H</u>), 8.63 (*ddd*, 1H, 2-C_5H_4N, *J* = 4.8, 1.7, 1.0 Hz), 12.65 (*s*, 1H, N<u>H</u>N); **^{13}C NMR** (75.45 MHz, DMSO-d_6) δ (ppm) 36.5 (NH<u>C</u>H$_2$), 55.3 (4-O<u>C</u>H$_3$-C_6H_4), 113.3 (C4), 113.9 (C_6H_5), 114.6 (4-OCH_3-C_6H_4), 120.1, 124.8 (2-C_5H_4N), 126.5 (4-OCH_3-C_6H_4), 127.4, 129.4, 131.0 (C_6H_5), 137.0 (2-C_5H_4N), 142.6 (4-OCH_3-C_6H_4), 149.7 (C=N), 149.8 (2-C_5H_4N), 151.4 (4-OCH_3-C_6H_4), 153.0 (2-C_5H_4N), 156.1 (C3), 157.2 (C=O), 168.2 (C5); **HRMS** (ESI+): calcd for $C_{24}H_{22}N_5O_3^+$, $[M+H]^+$: 428.1717, found 428.1734.

3-[(2*E*)-*N*´-(2-pyridinylmethylene)hydrazinecarbonyl]-4-(phenyl)aminomethyl-5-(4-fluorophenyl)isoxazole (5ca): Yellow solid; 87% yield; mp 157.7-159.6 °C; **^{1}H NMR** (300.06 MHz, DMSO-d_6) δ (ppm) 4.37 (*d*, 2H, NHC<u>H</u>$_2$, J = 4.3 Hz), 5.91 (*ls*, 1H, N<u>H</u>CH$_2$), 6.56-6.61 (*m*, 3H, C_6H_5), 7.07 (*dd*, 2H, C_6H_5), 7.43-7.49 (*m*, 3H, 4-F-

C_6H_4 and 2-C_5H_4N), 7.87-7.99 (*m*, 4H, 4-F-C_6H_4 and 2-C_5H_4N), 7.87-7.92 (*m*, 2H, 4-F-C_6H_4), 8.54 (*s*, 1H, NC<u>H</u>), 8.63 (*d*, 1H, 2-C_5H_4N, *J* = 4.6 Hz), 12.63 (*s*, 1H, N<u>H</u>N); **^{13}C NMR** (75.45 MHz, DMSO-*d_6*) δ (ppm) 35.6 (NH<u>C</u>H_2), 112.6 (C_6H_5), 113.0 (C4), 116.6 (C_6H_5), 116.7 (*d*, 4-F-C_6H_4, $^2J_{C\text{-}F}$ = 22.1 Hz), 120.1 (2-C_5H_4N), 123.0 (*d,* 4-F-C_6H_4, $^4J_{C\text{-}F}$ = 3.2 Hz), 124.8 (2-C_5H_4N), 128.9 (C_6H_5), 129.9 (*d*, 4-F-C_6H_4, $^3J_{C\text{-}F}$ = 8.9 Hz), 137.0 (2-C_5H_4N), 148.4 (C_6H_5), 149.6 (C=N), 149.8, 152.9 (2-C_5H_4N), 156.0 (C3), 161.8 (<u>C</u>=O), 163.4 (*d,* 4-F-C_6H_4, $^1J_{C\text{-}F}$ = 249.8 Hz), 167.4 (C5); **HRMS** (ESI+): calcd for $C_{23}H_{19}FN_5O_2^+$, $[M+H]^+$: 416.1517, found 416.1519.

3-[(2*E*)-*N´*-(2-pyridinylmethylene)hydrazinecarbonyl]-4-[(4-chlorophenyl)aminomethyl]-5-(4-fluorophenyl)isoxazole (**5cb**): Beige solid; 93% yield; mp 202.4-203.9 ºC; **^{1}H NMR** (300.06 MHz, DMSO-*d_6*) δ (ppm) 4.35 (*s*, 2H, NHC<u>H</u>$_2$), 6.15 (*ls*, 1H, N<u>H</u>CH_2), 6.60 (*d*, 2H, 4-Cl-C_6H_4, *J* = 8.9 Hz), 7.09 (*m*, 2H, 4-Cl-C_6H_4, *J* = 8.9 Hz), 7.42-7.49 (*m*, 3H, 4-F-C_6H_4 and 2-C_5H_4N), 7.85-7.98 (*m*, 4H, 4-F-C_6H_4 and 2-C_5H_4N), 8.54 (*s*, 1H, NC<u>H</u>), 8.63 (*d*, 1H, 2-C_5H_4N), 12.63 (*s,* 1H, N<u>H</u>N); **^{13}C NMR** (75.45 MHz, DMSO-*d_6*) δ (ppm) 35.6 (NH<u>C</u>H_2), 112.7 (C4), 113.9 (4-Cl-C_6H_4), 116.6 (*d*, 4-F-C_6H_4, $^2J_{C\text{-}F}$ = 22.2 Hz), 119.9 (4-Cl-C_6H_4), 120.1 (2-C_5H_4N), 122.9 (*d,* 4-F-C_6H_4, $^4J_{C\text{-}F}$ = 3.3 Hz), 124.8 (2-C_5H_4N), 128.6 (4-Cl-C_6H_4), 129.9 (*d*, 4-F-C_6H_4, $^3J_{C\text{-}F}$ = 8.9 Hz), 137.0 (2-C_5H_4N), 147.2 (4-Cl-C_6H_4), 149.6 (C=N), 149.8, 152.9 (2-C_5H_4N), 155.9 (C3), 156.9 (<u>C</u>=O), 163.5 (*d,* 4-F-C_6H_4, $^1J_{C\text{-}F}$ = 249.5 Hz), 167.5 (C5); **HRMS** (ESI+): calcd for $C_{23}H_{18}ClFN_5O_2^+$, $[M+H]^+$: 450.1128, found 450.1126.

3-[(2*E*)-*N´*-(2-pyridinylmethylene)hydrazinecarbonyl]-4-[(4-methoxyphenyl)aminomethyl]-5-(4-fluorophenyl)isoxazole (**5cc**): White solid; 92% yield; mp 188.7-189.5 ºC; **^{1}H NMR** (300.06 MHz, DMSO-*d_6*) δ (ppm) 3.63 (*s*, 3H, 4-OC<u>H</u>$_3$-C_6H_4), 4.31 (*d*, 2H, NHC<u>H</u>$_2$, *J* = 4.4 Hz), 5.50 (*s*, 1H, N<u>H</u>CH_2), 6.57 (*d*, 2H, 4-OCH_3-C_6H_4, *J* = 9.0), 6.71 (*d*, 2H, 4-OCH_3-C_6H_4, *J* = 9.0), 7.42-7.49 (*m*, 3H, 4-F-C_6H_4 and 2-C_5H_4N), 7.88-7.99 (*m*, 4H, 4-F-C_6H_4 and 2-C_5H_4N), 8.52 (*s*, 1H, NC<u>H</u>), 8.63 (*d*, 1H, 2-C_5H_4N, *J* = 4.5 Hz), 12.62 (*s*, 1H, N<u>H</u>N); **^{13}C NMR** (75.45 MHz, DMSO-*d_6*) δ (ppm) 36.4 (NH<u>C</u>H_2), 55.3 (O<u>C</u>H_3), 113.2 (C4), 114.0, 114.5 (4-O<u>C</u>H_3-C_6H_4), 116.6 (*d,* 4-F-C_6H_4, $^2J_{C\text{-}F}$ = 22.2 Hz), 120.1 (2-C_5H_4N), 123.1 (*d,* 4-F-C_6H_4, $^4J_{C\text{-}F}$ = 3.3 Hz), 124.8 (2-C_5H_4N), 129.9 (*d*, 4-F-C_6H_4, $^3J_{C\text{-}F}$ = 8.8 Hz), 124.8 (2-C_5H_4N), 142.5 (4-O<u>C</u>H_3-C_6H_4), 149.6 (C=N), 149.8 (2-C_5H_4N), 151.4 (4-O<u>C</u>H_3-C_6H_4), 152.9 (2-C_5H_4N), 156.0 (C3), 157.1 (<u>C</u>=O), 163.4 (*d,* 4-F-C_6H_4, $^1J_{C\text{-}F}$ = 249.4 Hz), 167.3 (C5); **HRMS** (ESI+): calcd for $C_{24}H_{21}FN_5O_3^+$, $[M+H]^+$: 446.1623, found 446.1639.

3-[(2*E*)-*N´*-(2-pyridinylmethylene)hydrazinecarbonyl]-4-(4-(phenyl)aminomethyl-5-(4-chlorophenyl)isoxazole (**5da**): White solid; 92% yield; mp 180.5-182.9 ºC; **^{1}H NMR** (300.06 MHz, DMSO-*d_6*) δ (ppm) 4.37 (*d*, 2H, NHC<u>H</u>$_2$, *J* = 5.0 Hz), 5.94 (*t*, 1H, N<u>H</u>CH_2, *J* = 5.0 Hz), 6.56-6.61 (*m*, 3H, C_6H_5), 7.04-7.09 (*m*, 2H, C_6H_5), 7.44 (*ddd*, 1H, 2-C_5H_4N, *J* = 7.4, 4.8, 1.3 Hz), 7.68 (*d*, 2H, 4-Cl-C_6H_4, *J* = 8.7 Hz), 7.85 (*d*, 2H, 4-Cl-C_6H_4, J = 8.7 Hz), 7.90 (*dd*, 1H, 2-C_5H_4N, J = 7.6, 1.6 Hz), 7.97 (*d*, 1H, 2-C_5H_4N, J = 7.6 Hz), 8.53 (*s*, 1H, NC<u>H</u>), 8.63 (*ddd*, 1H, 2-C_5H_4N, *J* = 4.8, 1.6, 1.0 Hz), 12.66 (*s*, 1H, N<u>H</u>N); **^{13}C NMR** (75.45 MHz, DMSO-*d_6*) δ (ppm) 35.6 (NH<u>C</u>H_2), 112.7 (C_6H_5), 113.6 (C4), 116.7 (C_6H_5), 120.1, 124.8 (2-C_5H_4N), 125.2, 128.9, 129.1 (4-Cl-C_6H_4), 129.6 (C_6H_5), 135.8 (4-Cl-C_6H_4), 137.0 (2-C_5H_4N), 148.4 (C_6H_5), 149.6 (C=N), 149.9, 152.9 (2-C_5H_4N), 155.9 (C3), 157.1 (C=O), 167.1 (C5); **HRMS** (ESI+): calcd for $C_{23}H_{19}ClN_5O_2^+$, $[M+H]^+$: 432.1222, found 432.1241.

3-[(2*E*)-*N´*-(2-pyridinylmethylene)hydrazinecarbonyl]-4-[4-(chlorophenyl)aminomethyl]-5-(4-chlorophenyl)isoxazole (**5db**): Gray solid; 93% yield; mp 175.7-176.4 ºC; **^{1}H NMR** (300.06 MHz, DMSO-*d_6*) δ (ppm) 4.29-4.36 (*m*, 2H, NHC<u>H</u>$_2$), 6.15 (*ls*, 1H, N<u>H</u>CH_2), 6.57-6.61 (*m*, 2H, 4-Cl-C_6H_4 - B), 7.09 (*d*, 2H, 4-Cl-C_6H_4 - B, *J* = 8.2 Hz), 7.45 (*ddd*, 1H, 2-C_5H_4N, *J* = 7.2, 4.8, 1.3 Hz), 7.64-7.70 (*m*, 2H, 4-Cl-C_6H_4 - A), 7.78-7.84 (*m*, 2H, 4-Cl-C_6H_4 - A), 7.91 (*dd,* 1H, 2-C_5H_4N, *J* = 7.4, 1.5 Hz), 7.97 (*d,* 1H, 2-C_5H_4N, *J* = 7.8 Hz), 8.53 (*s*, 1H, NC<u>H</u>), 8.63 (*d*, 1H, 2-C_5H_4N, *J* = 4.9 Hz), 12.67 (*s,* 1H, N<u>H</u>N); **^{13}C NMR** (75.45 MHz, , DMSO-*d_6*) δ (ppm) 35.7 (NH<u>C</u>H_2), 113.3 (C4), 114.0 (4-Cl-C_6H_4 – A and B), 120.2, 124.9 (2-C_5H_4N), 125.2, 125.3, 128.6, 129.1, 129.6, 135.9 (4-Cl-C_6H_4 – A and B), 137.1 (2-C_5H_4N), 147.2 (4-Cl-C_6H_4 – A or B), 149.7 (C=N), 149.9, 152.9 (2-C_5H_4N), 155.9 (C3), 157.0 (C=O), 167.3 (C5); **HRMS** (ESI+): calcd for $C_{23}H_{18}Cl_2N_5O_2^+$, $[M+H]^+$: 466.0832, found 466.0819.

3-[(2*E*)-*N´*-(2-pyridinylmethylene)hydrazinecarbonyl]-4-[(4-methoxyphenyl)aminomethyl]-5-(4-chlorophenyl)isoxazole (**5dc**): Gray solid; 92% yield; mp 210.4-212.4 ºC; **^{1}H NMR** (300.06 MHz, DMSO-*d_6*) δ (ppm) 3.63 (*s*, 3H, 4-OC<u>H</u>$_3$-C_6H_4), 4.33 (*s*, 2H, NHC<u>H</u>$_2$), 5.54 (*ls*, 1H, N<u>H</u>CH_2), 6.58 (*d*, 2H, 4-OCH_3-C_6H_4, *J* = 8.9 Hz), 6.71 (*d*, 2H, 4-OCH_3-C_6H_4, *J* = 8.9 Hz), 7.44 (*ddd*, 1H, 2-C_5H_4N, *J* = 7.3, 4.8, 1.3 Hz), 7.68 (*d*, 2H, 4-Cl-C_6H_4, *J* = 8.7), 7.84-7.99 (*m*, 4H, 4-Cl-C_6H_4 and 2-C_5H_4N), 8.52 (*s*, 1H, NC<u>H</u>), 8.63 (*d*, 1H, 2-C_6H_4N, *J* = 4.8, 1.5, 1.5 Hz), 12.64 (*s,* 1H, N<u>H</u>N); **^{13}C NMR** (75.45 MHz, DMSO-*d_6*) δ (ppm) 36.5 (NH<u>C</u>H_2), 55.3 (4-O<u>C</u>H_3-C_6H_4), 113.7 (C4), 114.2, 114.5 (4-O<u>C</u>H_3-C_6H_4), 120.2, 124.8 (2-C_5H_4N), 125.2 (4-Cl-C_6H_4), 129.1, 129.5 (4-Cl-C_6H_4), 135.7 (4-Cl-C_6H_4), 137.0 (2-C_5H_4N), 142.3 (4-O<u>C</u>H_3-C_6H_4), 149.6 (C=N), 149.8, 151.6 (2-C_5H_4N), 152.9 (4-O<u>C</u>H_3-C_6H_4), 156.0 (C3), 157.1 (<u>C</u>=O), 167.1 (C5); **HRMS** (ESI+): calcd for $C_{24}H_{21}ClN_5O_3^+$, $[M+H]^+$: 462.1327, found 462.1336.

Synthesis of 3-[(2*E*)-*N*´-(2,2´-bithienyl-5-methylene)hydrazinecarbonyl]-4-[(aryl)aminomethyl]-5-arylisoxazoles 6(aa-ac)-6(da-dc)

General method. The compound **3** (1.0 mmol, 1.0 equiv) was solubilized in DMSO (2 mL), and 2,2′-bithiophene-5-carboxaldehyde (0.194 g, 1.0 mmol, 1.0 equiv) and two drops of hydrochloric acid (37%) were added. The mixture was stirred at room temperature for 2 h. Then, cold distilled water (50 mL) was added, and the product was filtered and dried under a vacuum.

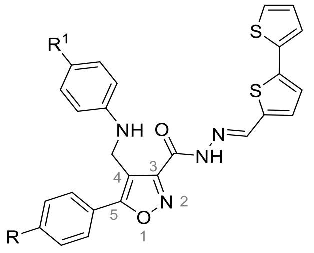

6(aa-ac)-6(da-dc)

3-[(2*E*)-*N*´-(2,2´-bithienyl-5-methylene)hydrazinecarbonyl]-4-(phenyl)aminomethyl-5-(4-nitrophenyl)isoxazole (**6aa**): Yellow solid; 61% yield; mp 232.5-233.9 °C; **^{1}H NMR** (300.06 MHz, DMSO-*d$_6$*) δ (ppm) 4.45 (*s*, 2H, NHC$\underline{H}_2$), 5.95 (*ls,* 1H, N$\underline{H}$CH$_2$), 6.57-6.60 (*m*, 3H, C_6H_5), 7.05-7.08 (*m*, 2H, C_6H_5), 7.13 (*dd*, 1H, $C_8H_5S_2$, *J* = 5.1, 3.6 Hz), 7.33 (*d*, 1H, $C_8H_5S_2$, *J* = 3.8 Hz), 7.44-7.46 (*m,* 2H, $C_8H_5S_2$), 7.60 (*dd*, 1H, $C_8H_5S_2$, *J* = 5.1, 1.1 Hz), 8.11 (*d*, 2H, 4-NO$_2$-C$_6$H$_4$, *J* = 8.9 Hz), 8.42 (*d*, 2H, 4-NO$_2$-C$_6$H$_4$, *J* = 8.9 Hz), 8.63 (*s*, 1H, NC$\underline{H}$), 12.47 (*s,* 1H, N$\underline{H}$N); **^{13}C NMR** (75.45 MHz, DMSO-*d$_6$*) δ (ppm) 35.6 (NH$\underline{C}$H$_2$), 112.7 (C_6H_5), 115.5 (C4), 116.8 (C_6H_5), 124.4 (4-NO$_2$-C$_6$H$_4$), 124.4, 125.3, 126.7, 128.6 ($C_8H_5S_2$), 128.6 (4-NO$_2$-C$_6$H$_4$), 128.9 (C_6H_5), 132.1 (4-NO$_2$-C$_6$H$_4$), 132.9, 135.9, 137.2, 139.4 ($C_8H_5S_2$), 144.2 (C=N), 148.2 (4-NO$_2$-C$_6$H$_4$), 148.4 (C_6H_5), 155.2 (C3), 157.3 (C=O), 165.8 (C5); **HRMS** (ESI+): calcd for $C_{26}H_{20}N_5O_4S_2^+$, [M+H]$^+$: 530.0951, found 530.0973.

3-[(2*E*)-*N*´-(2,2´-bithienyl-5-methylene)hydrazinecarbonyl]-4-[(4-chlorophenyl)aminomethyl]-5-(4-nitrophenyl)isoxazole (**6ab**): Dark yellow solid; 90% yield; mp 202.3-203.7 °C; **^{1}H NMR** (300.06 MHz, DMSO-*d$_6$*) δ (ppm) 4.44 (*s*, 2H, NHC$\underline{H}_2$), 6.17 (*s,* 1H, N$\underline{H}$CH$_2$), 6.58 (*d*, 2H, 4-Cl-C$_6$H$_4$, J = 8.9 Hz), 7.08 (*d*, 2H, 4-Cl-C$_6$H$_4$, J = 8.9 Hz), 7.13 (*dd*, 1H, $C_8H_5S_2$, *J* = 5.1, 3.6 Hz), 7.33 (*d*, 1H, $C_8H_5S_2$, *J* = 3.8 Hz), 7.43-7.45 (*m,* 2H, $C_8H_5S_2$), 7.59 (*dd*, 1H, $C_8H_5S_2$, *J* = 5.1, 1.1 Hz), 8.08 (*d*, 2H, 4-NO$_2$-C$_6$H$_4$, *J* = 8.9 Hz), 8.41 (*d*, 2H, 4-NO$_2$-C$_6$H$_4$, *J* = 8.9 Hz), 8.63 (*s*, 1H, NC$\underline{H}$), 12.47 (*s,* 1H, N$\underline{H}$N); **^{13}C NMR** (75.45 MHz, DMSO-*d$_6$*) δ (ppm) 35.6 (NH$\underline{C}$H$_2$), 114.0 (4-Cl-C$_6$H$_4$), 115.2 (C4), 120.0 (4-Cl-C$_6$H$_4$), 124.4 ($C_8H_5S_2$), 124.4 (4-NO$_2$-C$_6$H$_4$), 125.3, 126.7, 128.6 ($C_8H_5S_2$), 128.6 (4-Cl-C$_6$H$_4$), 128.7, 132.0 (4-NO$_2$-C$_6$H$_4$), 132.9, 135.9, 137.2, 139.4 ($C_8H_5S_2$), 144.3 (C=N), 147.1 (4-Cl-C$_6$H$_4$), 148.4 (4-NO$_2$-C$_6$H$_4$), 155.2 (C3), 157.2 (C=O), 165.9 (C5); **HRMS** (ESI+): calcd for $C_{26}H_{19}ClN_5O_4S_2^+$, [M+H]$^+$: 564.0562, found 564.0562.

3-[(2*E*)-*N*´-(2,2´-bithienyl-5-methylene)hydrazinecarbonyl]-4-[(4-methoxyphenyl)aminomethyl]-5-(4-nitrophenyl)isoxazole (**6ac**): Dark yellow solid; 95% yield; mp 193.5-194.9 °C; **^{1}H NMR** (300.06 MHz, DMSO-*d$_6$*) δ (ppm) 3.63 (*s*, 3H, 4-OC$\underline{H}_3$-C$_6$H$_4$), 4.38 (*s*, 2H, NHC$\underline{H}_2$), 5.56 (*ls,* 1H, N$\underline{H}$CH$_2$), 6.56 (*d*, 2H, 4-OCH$_3$-C$_6$H$_4$, *J* = 8.9 Hz), 6.70 (*d*, 2H, 4-OCH$_3$-C$_6$H$_4$, *J* = 8.9 Hz), 7.13 (*dd*, 1H, $C_8H_5S_2$, *J* = 5.1, 3.6 Hz), 7.33 (*d*, 1H, $C_8H_5S_2$, *J* = 3.8 Hz), 7.43-7.45 (*m,* 2H, $C_8H_5S_2$), 7.59 (*dd*, 1H, $C_8H_5S_2$, *J* = 5.1, 1.1 Hz), 8.12 (*d*, 2H, 4-NO$_2$-C$_6$H$_4$, *J* = 9.0 Hz), 8.42 (*d*, 2H, 4-NO$_2$-C$_6$H$_4$, *J* = 9.0 Hz), 8.61 (*s*, 1H, NC$\underline{H}$), 12.50 (*s,* 1H, N$\underline{H}$N); **^{13}C NMR** (75.45 MHz, DMSO-*d$_6$*) δ (ppm) 36.4 (NH$\underline{C}$H$_2$), 55.3 (4-O$\underline{C}$H$_3$-C$_6$H$_4$), 114.2, 114.5 (4-OCH$_3$-C$_6$H$_4$), 115.7 (C4), 124.4 ($C_8H_5S_2$), 124.4 (4-NO$_2$-C$_6$H$_4$), 125.4, 126.7, 128.7 ($C_8H_5S_2$), 128.7, 132.1 (4-NO$_2$-C$_6$H$_4$), 132.9, 136.0, 137.2, 139.4 ($C_8H_5S_2$), 142.3 (4-OCH$_3$-C$_6$H$_4$), 144.2 (C=N), 148.4 (4-NO$_2$-C$_6$H$_4$), 151.6 (4-OCH$_3$-C$_6$H$_4$), 155.3 (C3), 157.4 (C=O), 165.7 (C5); **HRMS** (ESI+): calcd for $C_{27}H_{22}N_5O_5S_2^+$, [M+H]$^+$: 560.1057, found 560.1088.

3-[(2*E*)-*N*´-(2,2´-bithienyl-5-methylene)hydrazinecarbonyl]-4-(phenyl)aminomethyl-5-(phenyl)isoxazole (**6ba**): Yellow solid; 84% yield; mp 199.9-201.3 °C; **^{1}H NMR** (300.06 MHz, DMSO-*d$_6$*) δ (ppm) 4.36 (*d*, 2H, NHC$\underline{H}_2$, J = 4.7 Hz), 5.93 (*t,* 1H, N$\underline{H}$CH$_2$, J = 5.3 Hz), 6.55-6.60 (*m*, 3H, C_6H_5 - A), 7.05-7.08 (*m*, 2H, C_6H_5 - A), 7.12-7.17 (*m*, 1H, $C_8H_5S_2$), 7.33 (*d*, 1H, $C_8H_5S_2$, *J* = 3.8 Hz), 7.41-7.50 (*m,* 1H, $C_8H_5S_2$), 7.56-7.65 (*m*, 4H, $C_8H_5S_2$ and C_6H_5 – B), 7.79-7.84 (*m*, 2H, C_6H_5 – B), 8.62 (*s*, 1H, NC$\underline{H}$), 8.81 (*m*, 1H, $C_8H_5S_2$), 12.45 (*s,* 1H, N$\underline{H}$N); **^{13}C NMR** (75.45 MHz, DMSO-*d$_6$*) δ (ppm) 35.6 (NH$\underline{C}$H$_2$), 112.6 (C_6H_5 – A), 113.0 (C4), 116.6 (C_6H_5 – A), 124.4, 125.3, 126.7 ($C_8H_5S_2$), 127.3 (C_6H_5 - B), 128.7 ($C_8H_5S_2$), 128.9, 129.3, 129.4, 130.9 (C_6H_5 – A and B), 132.8,

136.0, 137.3, 139.3 ($C_8H_5S_2$), 144.1 (C=N), 148.4 (C_6H_5 – B), 155.6 (C3), 157.2 (C=O), 168.1 (C5); **HRMS** (ESI+): calcd for $C_{26}H_{21}N_4O_2S_2^+$, $[M+H]^+$: 485.1100, found 485.1118.

3-[(2*E*)-*N*´-(2,2´-bithienyl-5-methylene)hydrazinecarbonyl]-4-[(4-chlorophenyl)aminomethyl]-5-(phenyl)isoxazole (6bb): Yellow solid; 89% yield; mp 211.1-213.9 °C; **^{1}H NMR** (300.06 MHz, DMSO-*d_6*) δ (ppm) 4.35 (*s*, 2H, $NHCH_2$), 6.14 (*ls*, 1H, $NHCH_2$), 6.59 (*d*, 2H, 4-Cl-C_6H_4, *J* = 8.9 Hz), 7.08 (*d*, 2H, 4-Cl-C_6H_4, *J* = 8.9 Hz), 7.13 (*dd*, 1H, $C_8H_5S_2$, *J* = 5.1, 3.6 Hz), 7.33 (*d*, 1H, $C_8H_5S_2$, *J* = 3.8 Hz), 7.43-7.45 (*m,* 2H, $C_8H_5S_2$), 7.59-7.61 (*m*, 4H, $C_8H_5S_2$ and C_6H_5), 7.79-7.82 (*m*, 2H, C_6H_5), 8.63 (*s*, 1H, NCH), 12.41 (*s,* 1H, NHN); **^{13}C NMR** (75.45 MHz, DMSO-*d_6*) δ (ppm) 35.7 ($NHCH_2$), 112.7 (C_6H_5), 113.9 (C4), 119.8 (4-Cl-C_6H_4), 124.4, 125.3, 126.4 ($C_8H_5S_2$), 126.7 (4-Cl-C_6H_4), 127.3 (C_6H_5), 128.6 (4-Cl-C_6H_4), 128.6, 129.4, 131.0 (C_6H_5), 132.8, 136.0, 137.3, 139.3 ($C_8H_5S_2$), 144.1 (C=N), 147.2 (4-Cl-C_6H_4), 155.5 (C3), 157.1 (C=O), 168.2 (C5); **HRMS** (ESI+): calcd for $C_{26}H_{20}ClN_4O_2S_2^+$, $[M+H]^+$: 519.0711, found 519.0701.

3-[(2*E*)-*N*´-(2,2´-bithienyl-5-methylene)hydrazinecarbonyl]-4-[(4-methoxyphenyl)aminomethyl]-5-(phenyl)isoxazole (6bc): Yellow solid; 92% yield; mp 165.5-167.0 °C; **^{1}H NMR** (300.06 MHz, DMSO-*d_6*) δ (ppm) 3.63 (*s*, 3H, 4-OCH_3-C_6H_4), 4.31 (*s*, 2H, $NHCH_2$), 5.52 (*ls,* 1H, $NHCH_2$), 6.56 (*d*, 2H, 4-OCH_3-C_6H_4, *J* = 8.9 Hz), 6.70 (*d*, 2H, 4-OCH_3-C_6H_4, *J* = 8.9 Hz), 7.13 (*dd*, 1H, $C_8H_5S_2$, *J* = 5.1, 3.6 Hz), 7.33 (*d*, 1H, $C_8H_5S_2$, *J* = 3.8 Hz), 7.45 (*dd,* 2H, $C_8H_5S_2$, J = 3.7, 1.3 Hz), 7.59-7.61 (*m*, 4H, $C_8H_5S_2$ and C_6H_5), 7.83 (*dd*, 2H, C_6H_5, *J* = 6.7, 2.9 Hz), 8.60 (*s*, 1H, NCH), 12.45 (*s,* 1H, NHN); **^{13}C NMR** (75.45 MHz, DMSO-*d_6*) δ (ppm) 36.5 ($NHCH_2$), 55.3 (4-OCH_3-C_6H_4), 113.2 (C4), 114.0, 114.5 (4-OCH_3-C_6H_4), 124.4, 125.4, 126.5 ($C_8H_5S_2$), 126.7, 127.3 (C_6H_5), 128.7 ($C_8H_5S_2$), 129.4, 130.9 (C_6H_5), 132.8, 136.0, 137.3, 139.3 ($C_8H_5S_2$), 142.4 (4-OCH_3-C_6H_4), 144.0 (C=N), 151.4 (4-OCH_3-C_6H_4), 155.6 (C3), 157.3 (C=O), 168.1 (C5); **HRMS** (ESI+): calcd for $C_{27}H_{23}N_4O_3S_2^+$, $[M+H]^+$: 515.1206, found 515.1229.

3-[(2*E*)-*N*´-(2,2´-bithienyl-5-methylene)hydrazinecarbonyl]-4-(phenyl)aminomethyl-5-(4-fluorophenyl)isoxazole (6ca): Beige solid; 92% yield; mp 207.6-209.5 °C; **^{1}H NMR** (300.06 MHz, DMSO-*d_6*) δ (ppm) 4.35 (*s*, 2H, $NHCH_2$, J = 4.3 Hz), 5.92 (*s*, 1H, $NHCH_2$), 6.56-6.60 (*m*, 3H, C_6H_5), 7.04-7.09 (*m*, 2H, C_6H_5), 7.13 (*dd*, 1H, $C_8H_5S_2$, *J* = 5.1, 3.6 Hz), 7.33 (*d*, 1H, $C_8H_5S_2$, *J* = 3.8 Hz), 7.44-7.50 (*m*, 3H, 4-F-C_6H_4 and $C_8H_5S_2$), 7.59 (*dd*, 1H, $C_8H_5S_2$, *J* = 5.1, 1.1 Hz), 7.87-7.91 (*m*, 2H, 4-F-C_6H_4), 8.62 (*s*, 1H, NCH), 12.45 (*s,* 1H, NHN); **^{13}C NMR** (75.45 MHz, DMSO-*d_6*) δ (ppm) 35.6 ($NHCH_2$), 112.7 (C_6H_5), 113.0 (C4), 116.6 (C_6H_5), 116.7 (*d*, 4-F-C_6H_4, $^2J_{C-F}$ = 22.1 Hz), 123.1 (*d,* 4-F-C_6H_4, $^4J_{C-F}$ = 3.1 Hz), 124.4, 125.4, 126.8, 128.7 ($C_8H_5S_2$), 128.9 (C_6H_5), 129.9 (*d*, 4-F-C_6H_4, $^3J_{C-F}$ = 8.8 Hz), 132.9, 136.0, 137.3, 139.3 ($C_8H_5S_2$), 144.1 (C=N), 148.4 (C_6H_5), 155.5 (C3), 157.2 (C=O), 163.4 (*d,* 4-F-C_6H_4, $^1J_{C-F}$ = 249.4 Hz), 167.3 (C5); **HRMS** (ESI+): calcd for $C_{26}H_{20}FN_4O_2S_2^+$, $[M+H]^+$: 503.1006, found 503.0995.

3-[(2*E*)-*N*´-(2,2´-bithienyl-5-methylene)hydrazinecarbonyl]-4-[(4-chlorophenyl)aminomethyl]-5-(4-fluorophenyl)isoxazole (6cb): Yellow solid; 99% yield; mp 199.5-201.2 °C; **^{1}H NMR** (300.06 MHz, DMSO-*d_6*) δ (ppm) 4.34 (*s*, 2H, $NHCH_2$), 6.14 (*ls*, 1H, $NHCH_2$), 6.59 (*d*, 2H, 4-Cl-C_6H_4, *J* = 8.8 Hz), 7.08 (*m*, 2H, 4-Cl-C_6H_4, *J* = 8.8 Hz), 7.13 (*dd*, 1H, $C_8H_5S_2$, *J* = 5.1, 3.6 Hz), 7.33 (*d*, 1H, $C_8H_5S_2$, *J* = 3.8 Hz), 7.43-7.49 (*m*, 4H, 4-Cl-C_6H_4 and $C_8H_5S_2$), 7.59 (*dd*, 1H, $C_8H_5S_2$, *J* = 5.1, 1.1 Hz), 7.84-7.89 (*m*, 2H, 4-F-C_6H_4), 8.63 (*s*, 1H, NCH), 12.42 (*s,* 1H, NHN); **^{13}C NMR** (75.45 MHz, DMSO-*d_6*) δ (ppm) 35.6 ($NHCH_2$), 112.6 (C4), 113.9 (4-Cl-C_6H_4), 116.6 (*d,* 4-F-C_6H_4, $^2J_{C-F}$ = 22.2 Hz), 119.8 (4-Cl-C_6H_4), 122.9 (*d,* 4-F-C_6H_4, $^4J_{C-F}$ = 3.1 Hz), 124.4, 125.3, 126.7, 128.5 ($C_8H_5S_2$), 128.6 (4-Cl-C_6H_4), 129.9 (*d*, 4-F-C_6H_4, $^3J_{C-F}$ = 8.9 Hz), 132.8, 136.0, 137.3, 139.3 ($C_8H_5S_2$), 144.1 (C=N), 147.2 (4-Cl-C_6H_4), 155.4 (C3), 157.0 (C=O), 163.4 (*d,* 4-F-C_6H_4, $^1J_{C-F}$ = 249.6 Hz), 167.3 (C5); **HRMS** (ESI+): calcd for $C_{26}H_{19}ClFN_4O_2S_2^+$, $[M+H]^+$: 537.0616, found 537.0617.

3-[(2*E*)-*N*´-(2,2´-bithienyl-5-methylene)hydrazinecarbonyl]-4-[(4-methoxyphenyl)aminomethyl]-5-(4-fluorophenyl)isoxazole (6cc): Brown solid; 92% yield; mp 179.8-181.4 °C; **^{1}H NMR** (300.06 MHz, DMSO-*d_6*) δ (ppm) 3.63 (*s*, 3H, 4-OCH_3-C_6H_4), 4.31 (*d*, 2H, $NHCH_2$, *J* = 4.4 Hz), 5.50 (*s*, 1H, $NHCH_2$), 6.57 (*d*, 2H, 4-OCH_3-C_6H_4, *J* = 9.0), 6.71 (*d*, 2H, 4-OCH_3-C_6H_4, *J* = 9.0), 7.13 (*dd*, 1H, $C_8H_5S_2$, *J* = 5.1, 3.6 Hz), 7.33 (*d*, 1H, $C_8H_5S_2$, *J* = 3.8 Hz), 7.42-7.48 (*m*, 4H, 4-F-C_6H_4 and $C_8H_5S_2$), 7.59 (*dd*, 1H, $C_8H_5S_2$, *J* = 5.1, 1.1 Hz), 7.86-7.91 (*m*, 2H, 4-F-C_6H_4), 8.60 (*s*, 1H, NCH), 12.41 (*s*, 1H, NHN); **^{13}C NMR** (75.45 MHz, DMSO-*d_6*) δ (ppm) 36.7 ($NHCH_2$), 55.3 (OCH_3), 113.1 (C4), 114.3, 114.6 (4-OCH_3-C_6H_4), 116.6 (*d*, 4-F-C_6H_4, $^2J_{C-F}$ = 22.1 Hz), 123.1 (*d,* 4-F-C_6H_4, $^4J_{C-F}$ = 3.2 Hz), 124.4, 125.4, 126.7, 128.7 ($C_8H_5S_2$), 130.0 (*d*, 4-F-C_6H_4, $^3J_{C-F}$ = 8.8 Hz), 132.8, 136.0, 137.3, 139.4 ($C_8H_5S_2$), 142.1 (4-OCH_3-C_6H_4), 144.2 (C=N), 151.7 (4-OCH_3-C_6H_4), 155.6 (C3), 157.3 (C=O), 163.4 (*d,* 4-F-C_6H_4, $^1J_{C-F}$ = 249.4 Hz), 167.3 (C5); **HRMS** (ESI+): calcd for $C_{27}H_{22}FN_4O_3S_2^+$, $[M+H]^+$: 533.1112, found 533.1116.

3-[(2*E*)-*N*´-(2,2´-bithienyl-5-methylene)hydrazinecarbonyl]-4-(4-(phenyl)aminomethyl-5-(4-chlorophenyl)isoxazole (6da): Yellow solid; 89% yield; mp 208.4-209.9 °C; **^{1}H NMR** (300.06 MHz, DMSO-*d_6*)

δ (ppm) 4.36 (*d*, 2H, NHC<u>H</u>$_2$, *J* = 5.2 Hz), 5.90 (*t*, 1H, N<u>H</u>CH$_2$, *J* = 5.2 Hz), 6.57-6.61 (*m*, 3H, C_6H_5), 7.04-7.09 (*m*, 2H, C_6H_5), 7.13 (*dd*, 1H, $C_8H_5S_2$, *J* = 5.1, 3.6 Hz), 7.33 (*d*, 1H, $C_8H_5S_2$, *J* = 3.8 Hz), 7.43-7.45 (*m,* 2H, $C_8H_5S_2$), 7.59 (*dd*, 1H, $C_8H_5S_2$, *J* = 5.1, 1.1 Hz), 7.68 (*d*, 2H, 4-Cl-C_6H_4, *J* = 8.7 Hz), 7.85 (*d*, 2H, 4-Cl-C_6H_4, *J* = 8.7 Hz), 8.62 (*s*, 1H, NC<u>H</u>), 12.42 (*s,* 1H, N<u>H</u>N); **^{13}C NMR** (75.45 MHz, DMSO-*d$_6$*) δ (ppm) 35.6 (NH<u>C</u>H$_2$), 112.7 (C_6H_5), 113.5 (C4), 116.6 (C_6H_5), 124.4, 125.3 ($C_8H_5S_2$), 125.3 (4-Cl-C_6H_4), 126.7, 128.6 ($C_8H_5S_2$), 128.9, 129.1 (4-Cl-C_6H_4), 129.5 (C_6H_5), 132.8 ($C_8H_5S_2$), 135.7 (4-Cl-C_6H_4), 136.0, 137.3, 139.3 ($C_8H_5S_2$), 144.1 (C=N), 148.3 (C_6H_5), 155.4 (C3), 157.2 (C=O), 167.0 (C5); **HRMS** (ESI+): calcd for $C_{26}H_{20}ClN_4O_2S_2^+$, $[M+H]^+$: 519.0711, found 519.0719.

3-[(2*E*)-*N*´-(2,2´-bithienyl-5-methylene)hydrazinecarbonyl]-4-[4-(chlorophenyl)aminomethyl]-5-(4-chlorophenyl)isoxazole (**6db**): Light yellow solid; 85% yield; mp 219.6-222.4 ^{o}C; **^{1}H NMR** (300.06 MHz, DMSO-*d$_6$*) δ (ppm) 4.29-4.35 (*m*, 2H, NHC<u>H$_2$</u>), 6.16 (*ls*, 1H, N<u>H</u>CH$_2$), 6.59 (*d*, 2H, 4-Cl-C_6H_4 – B, J = 8.6 Hz), 7.08-7.14 (*m*, 3H, 4-Cl-C_6H_4 - B and $C_8H_5S_2$), 7.33 (*d*, 1H, $C_8H_5S_2$, *J* = 3.8 Hz), 7.44-7.46 (*m,* 2H, $C_8H_5S_2$), 7.60 (*d*, 1H, $C_8H_5S_2$, *J* = 5.1 Hz), 7.68 (*d*, 2H, 4-Cl-C_6H_4 – A, J = 8.6 Hz), 7.82 (*d*, 2H, 4-Cl-C_6H_4 – A, J = 8.6 Hz), 8.62 (*s*, 1H, NC<u>H</u>), 12.46 (*s,* 1H, N<u>H</u>N); **^{13}C NMR** (75.45 MHz, , DMSO-*d$_6$*) δ (ppm) 35.7 (NH<u>C</u>H$_2$), 113.2 (C4), 114.0, 119.9 (4-Cl-C_6H_4 – A and B), 124.4 ($C_8H_5S_2$), 125.2, 125.3 (4-Cl-C_6H_4 – A and B), 125.3, 126.7, 128.6 ($C_8H_5S_2$), 128.6, 128.9, 129.1, 129.6 (4-Cl-C_6H_4 – A and B), 132.8 ($C_8H_5S_2$), 135.9 (4-Cl-C_6H_4 – A and B), 136.0, 137.3, 139.4 ($C_8H_5S_2$), 144.2 (C=N), 147.2 (4-Cl-C_6H_4 – A or B), 155.4 (C3), 157.1 (C=O), 167.1 (C5); **HRMS** (ESI+): calcd for $C_{26}H_{19}Cl_2N_4O_2S_2^+$, $[M+H]^+$: 553.0321, found 553.0325.

3-[(2*E*)-*N*´-(2,2´-bithienyl-5-methylene)hydrazinecarbonyl]-4-[(4-methoxyphenyl)aminomethyl]-5-(4-chlorophenyl)isoxazole (**6dc**): Yellow solid; 94% yield; mp 199.9-202.7 ^{o}C; **^{1}H NMR** (300.06 MHz, DMSO-*d$_6$*) δ (ppm) 3.63 (*s*, 3H, 4-OC<u>H$_3$</u>-C_6H_4), 4.31 (*s*, 2H, NHC<u>H$_2$</u>), 5.52 (*ls*, 1H, N<u>H</u>CH$_2$), 6.57 (*d*, 2H, 4-OCH$_3$-C_6H_4, *J* = 8.8 Hz), 6.71 (*d*, 2H, 4-OCH$_3$-C_6H_4, *J* = 8.8 Hz), 7.13 (*dd*, 1H, $C_8H_5S_2$, *J* = 5.1, 3.6 Hz), 7.33 (*d*, 1H, $C_8H_5S_2$, *J* = 3.8 Hz), 7.44 (*d,* 2H, $C_8H_5S_2$, J = 3.8 Hz), 7.59 (*dd*, 1H, $C_8H_5S_2$, *J* = 5.1, 1.1 Hz), 7.68 (*d*, 2H, 4-Cl-C_6H_4, *J* = 8.7), 7.85 (*d*, 2H, 4-Cl-C_6H_4, *J* = 8.7), 8.60 (*s*, 1H, NC<u>H</u>), 12.46 (*s,* 1H, N<u>H</u>N); **^{13}C NMR** (75.45 MHz, DMSO-*d$_6$*) δ (ppm) 36.5 (NH<u>C</u>H$_2$), 55.3 (4-O<u>C</u>H$_3$-C_6H_4), 113.7 (C4), 114.1, 114.5 (4-O<u>C</u>H$_3$-C_6H_4), 124.4 ($C_8H_5S_2$), 125.3 (4-Cl-C_6H_4), 125.3, 126.7, 128.6 ($C_8H_5S_2$), 129.1, 129.5 (4-Cl-C_6H_4), 132.8 ($C_8H_5S_2$), 135.7 (4-Cl-C_6H_4), 136.0, 137.3, 139.3 ($C_8H_5S_2$), 142.4 (4-O<u>C</u>H$_3$-C_6H_4), 144.1 (C=N), 151.5 (4-O<u>C</u>H$_3$-C_6H_4), 155.5 (C3), 157.3 (<u>C</u>=O), 166.9 (C5); **HRMS** (ESI+): calcd for $C_{27}H_{22}ClN_4O_3S_2^+$, $[M+H]^+$: 549.0816, found 549.0836.

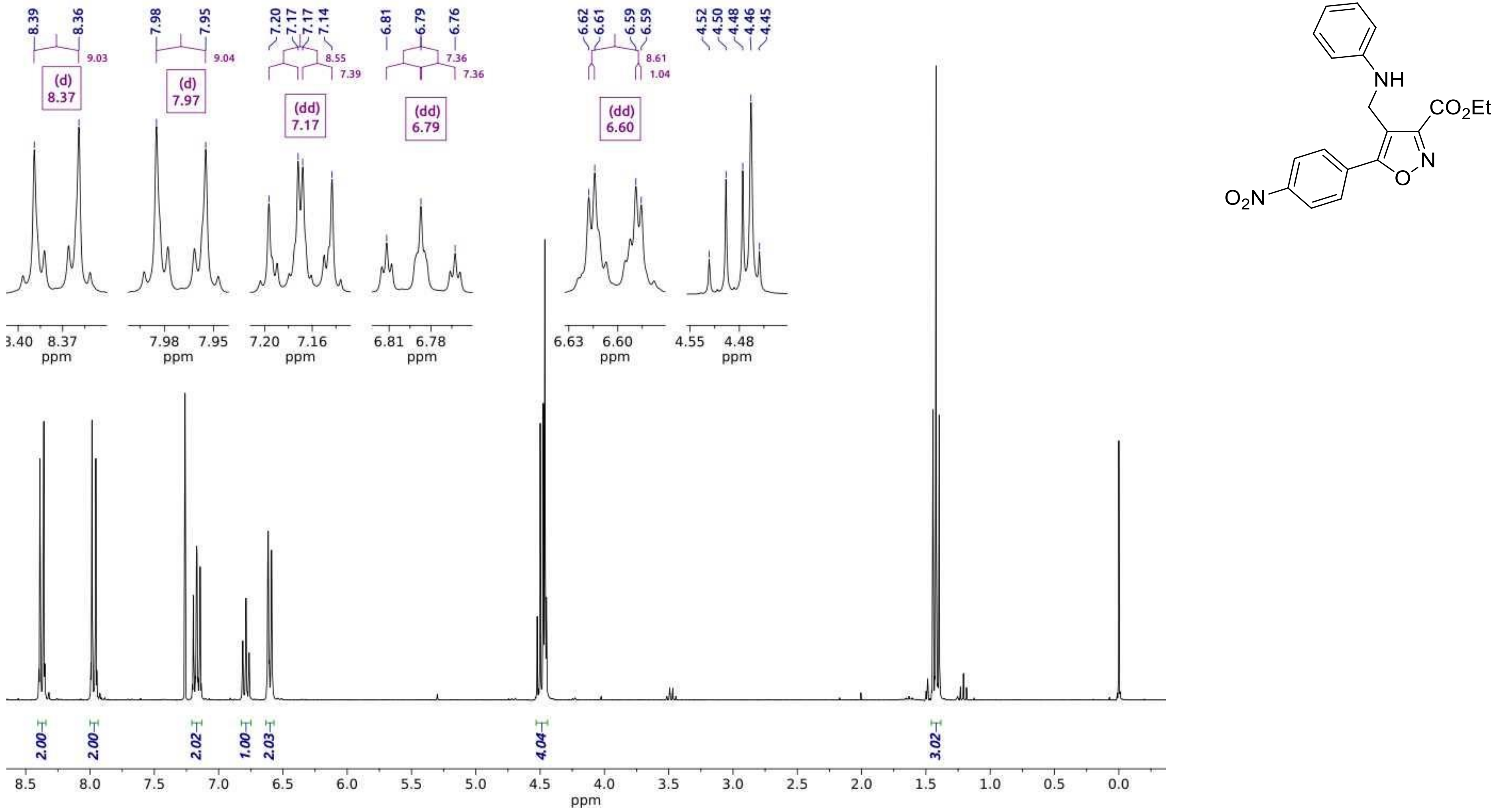

Figure S1 – ^{1}H NMR spectrum of compound **2aa** in $CDCl_3$ at 300.06 MHz.

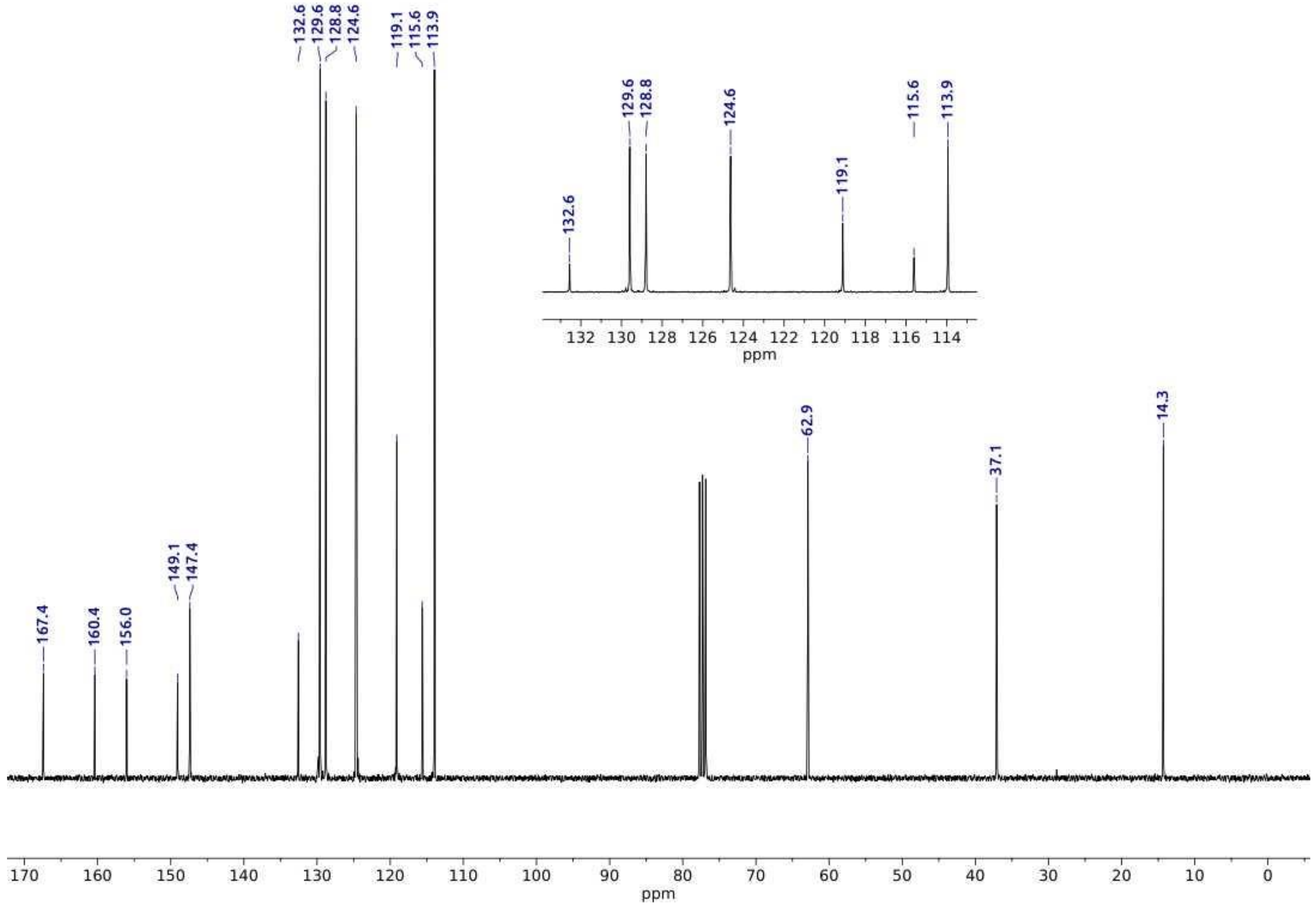

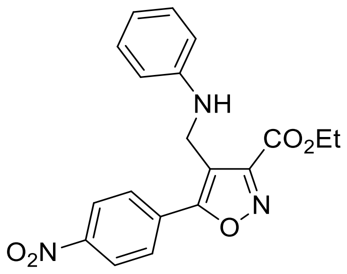

Figure S2 – ^{13}C NMR spectrum of compound **2aa** in $CDCl_3$ at 75.45 MHz.

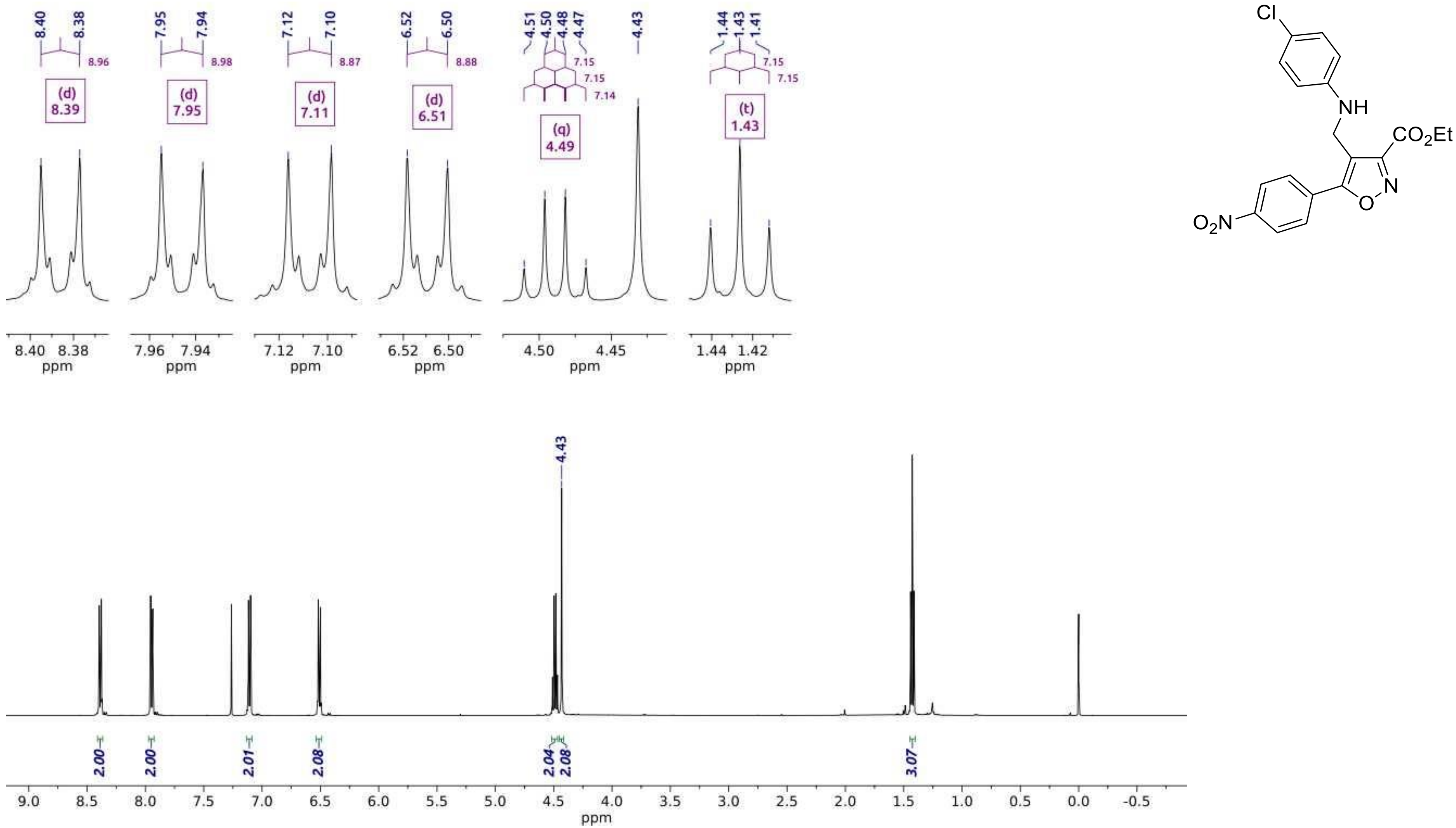

Figure S3 – ^{1}H NMR spectrum of compound **2ab** in $CDCl_3$ at 300.06 MHz.

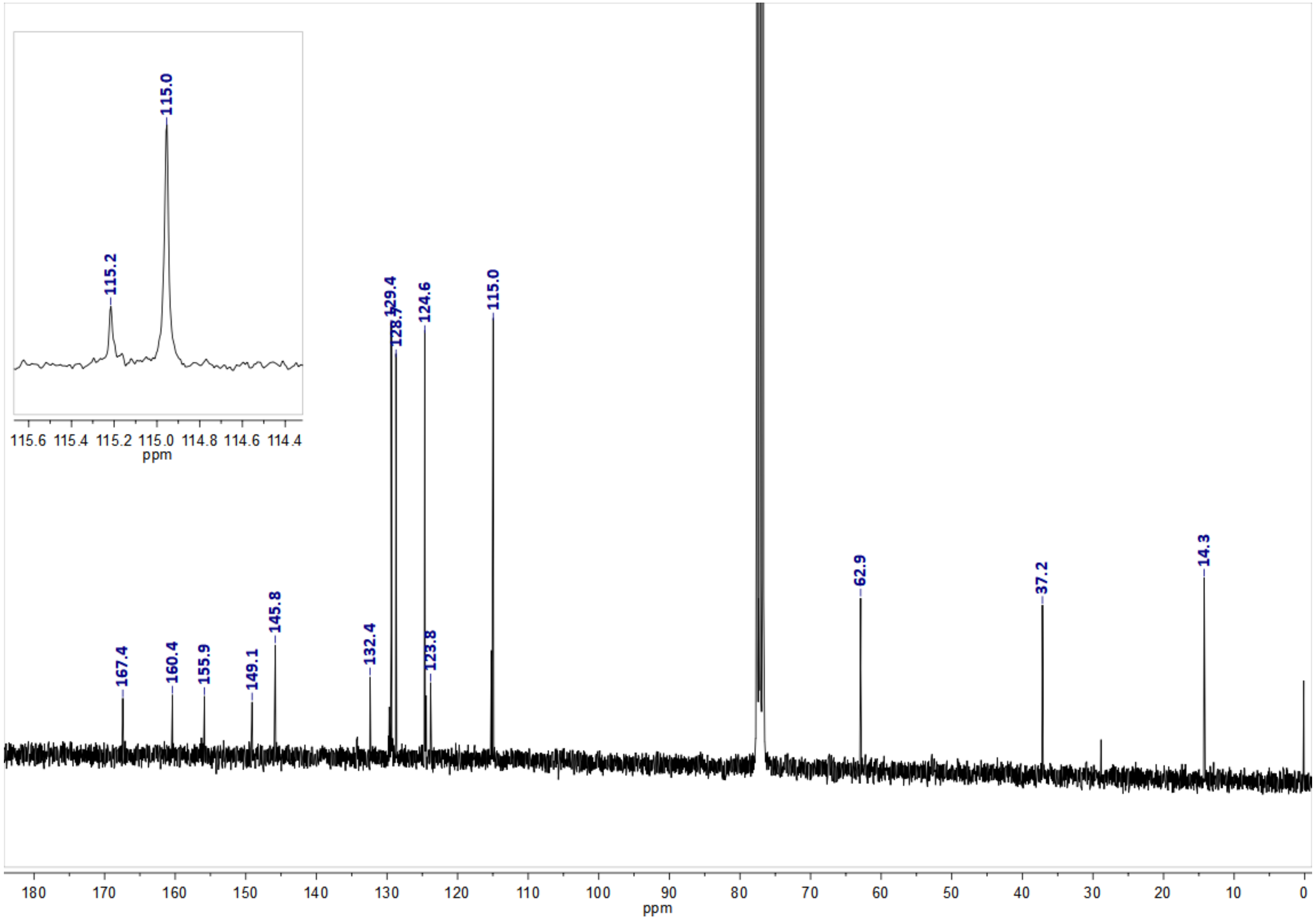

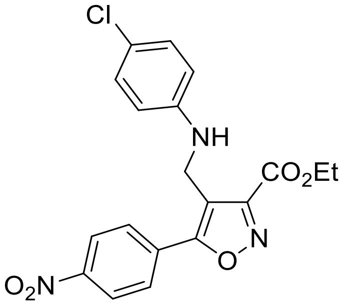

Figure S4 – ^{13}C NMR spectrum of compound **2ab** in $CDCl_3$ at 75.45 MHz.

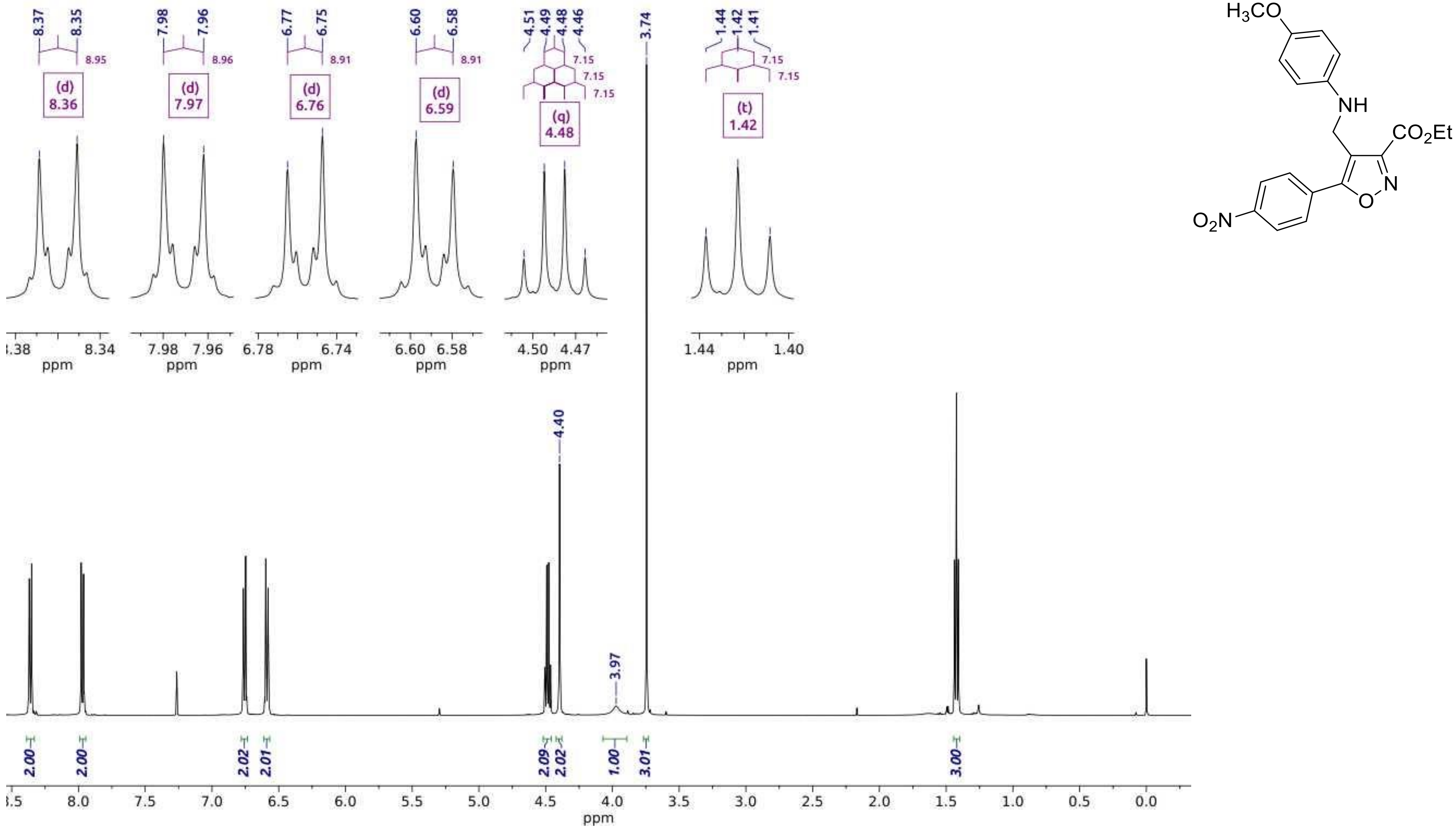

Figure S5 – ^{1}H NMR spectrum of compound **2ac** in $CDCl_3$ at 300.06 MHz.

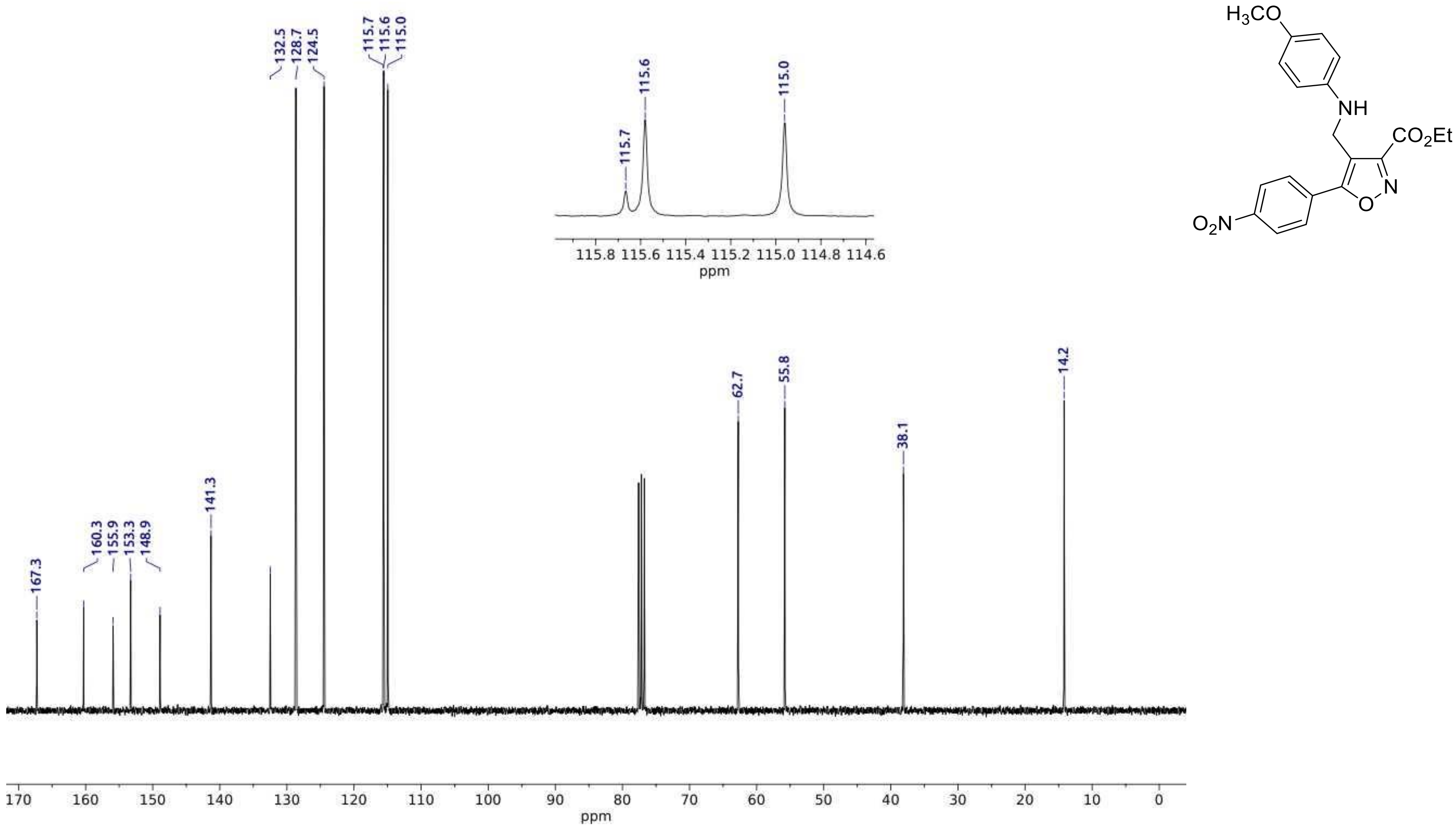

Figure S6 – ^{13}C NMR spectrum of compound **2ac** in $CDCl_3$ at 75.45 MHz.

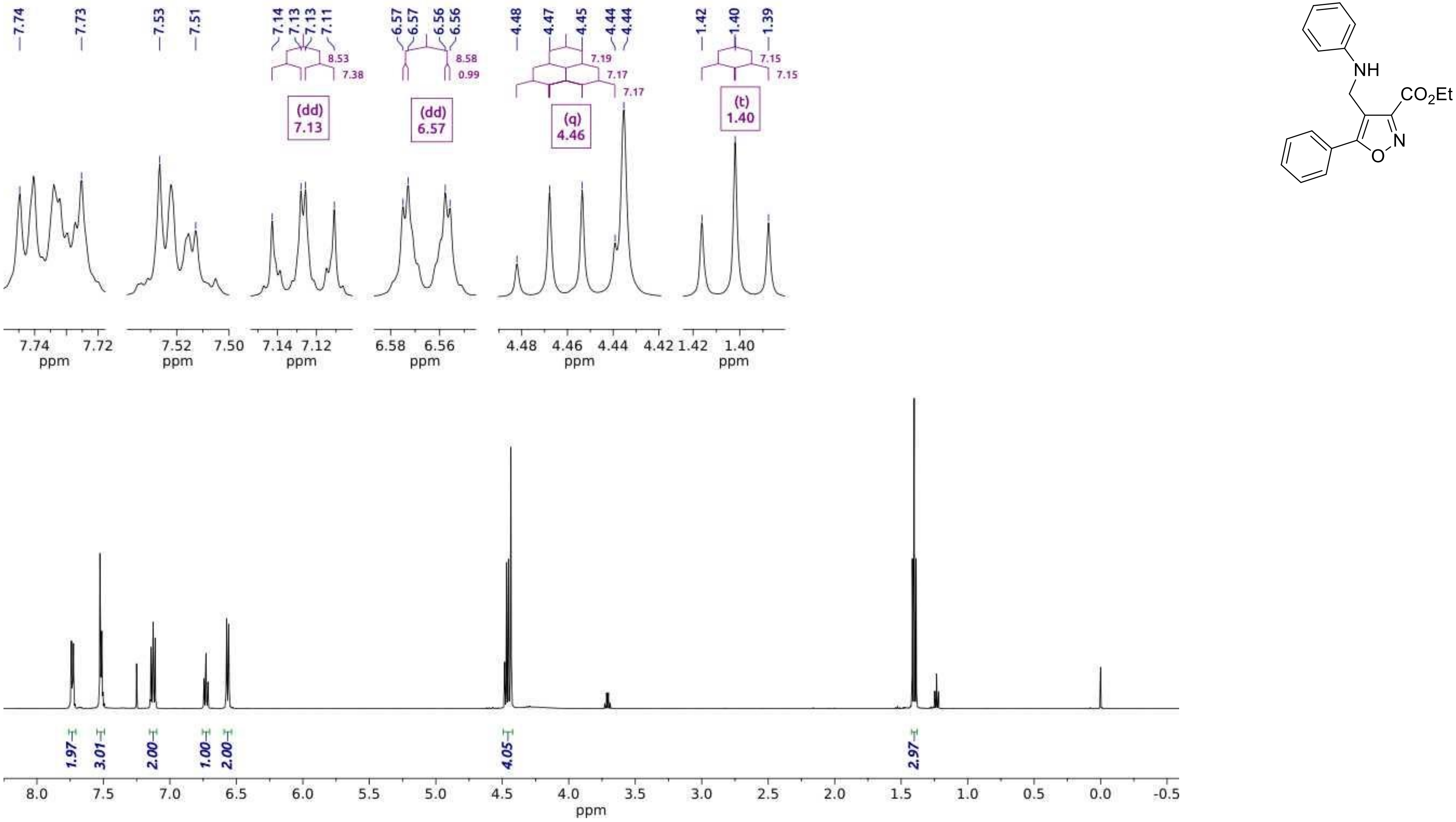

Figure S7 – ^{1}H NMR spectrum of compound **2ba** in $CDCl_3$ at 300.06 MHz.

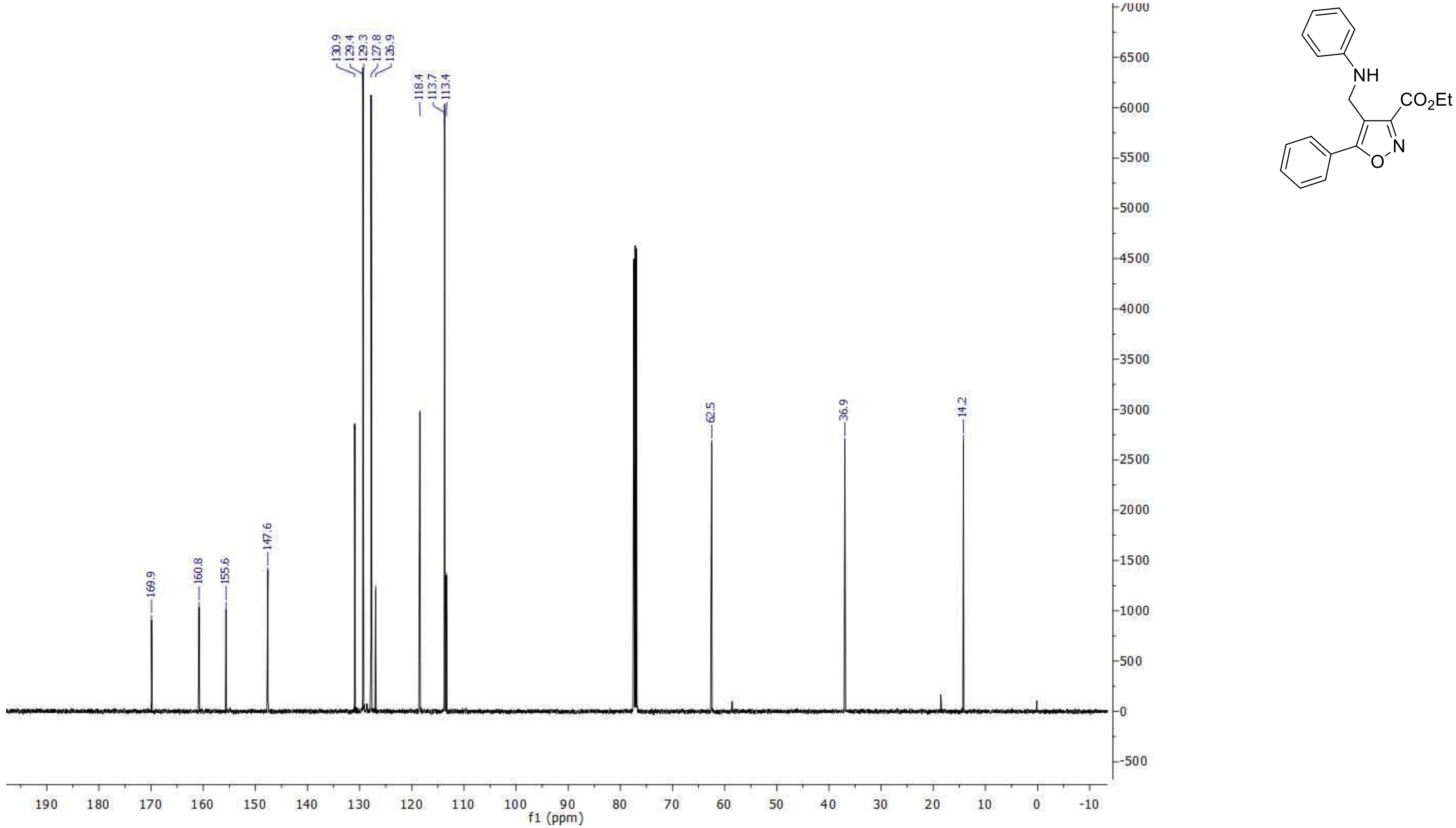

Figure S8 – ^{13}C NMR spectrum of compound **2ba** in $CDCl_3$ at 75.45 MHz.

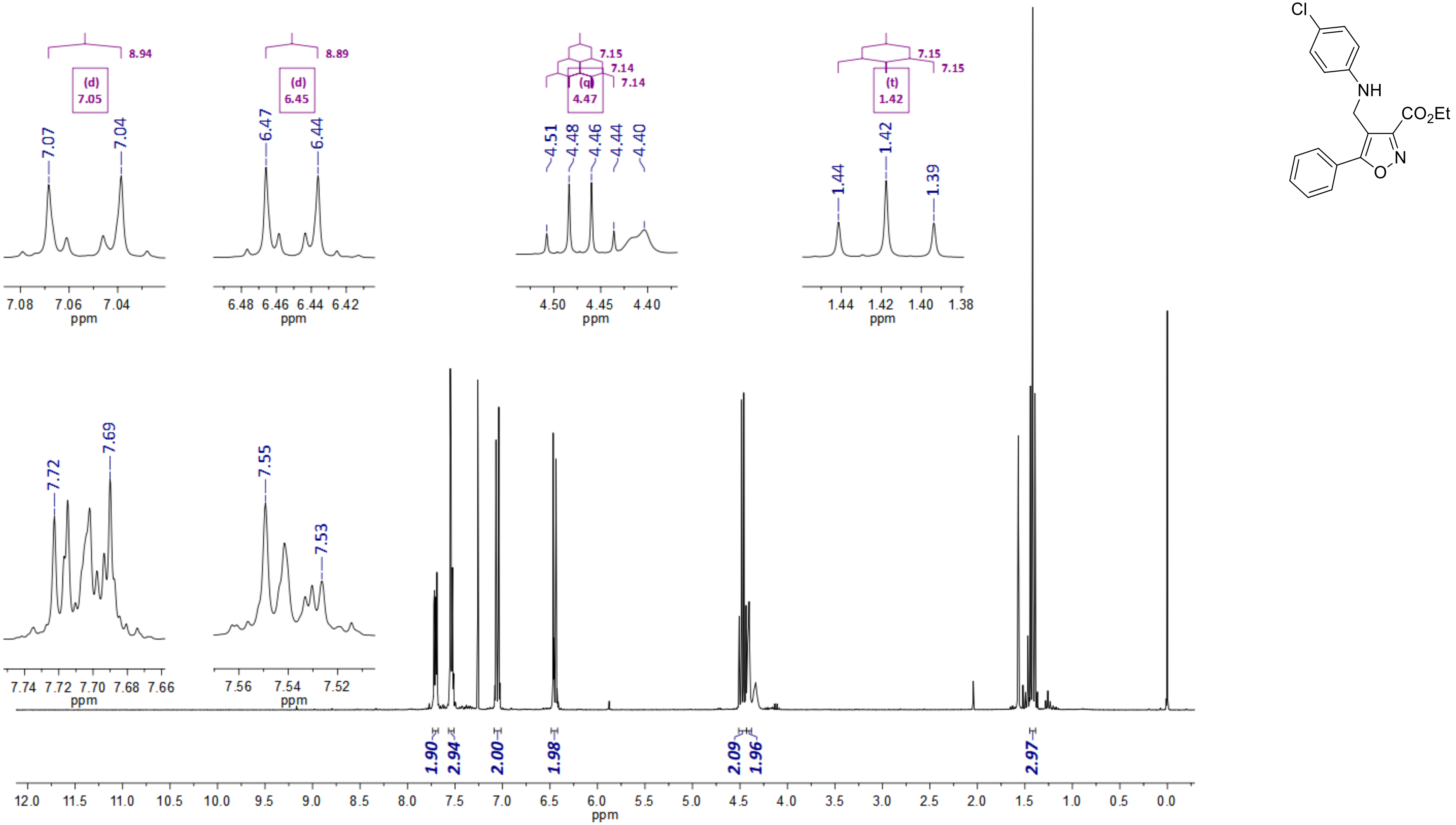

Figure S9 – ^{1}H NMR spectrum of compound **2bb** in $CDCl_3$ at 300.06 MHz.

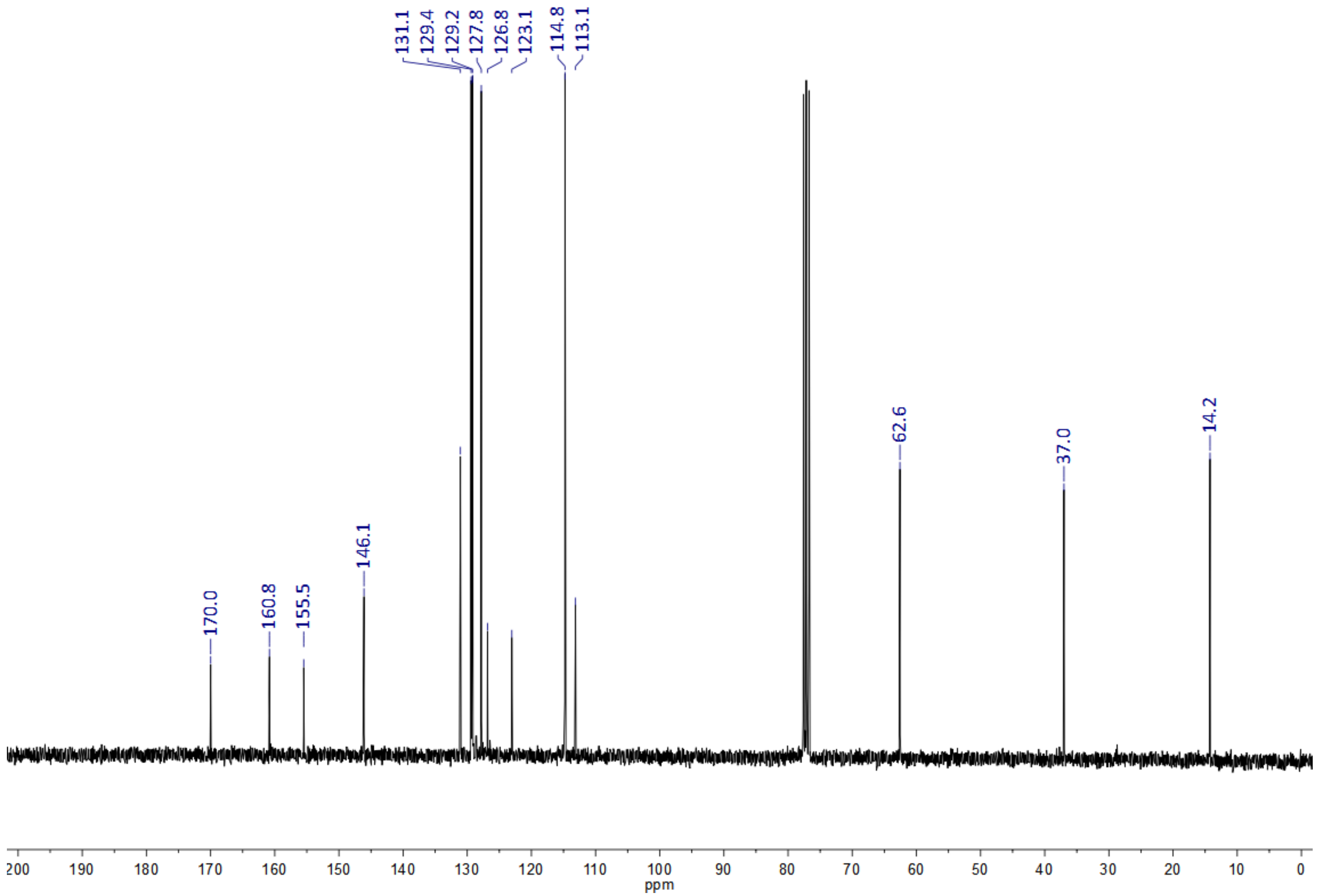

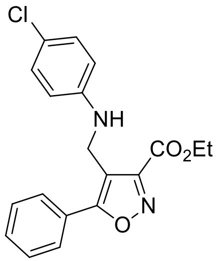

Figure S10 – ^{13}C NMR spectrum of compound **2bb** in $CDCl_3$ at 75.45 MHz.

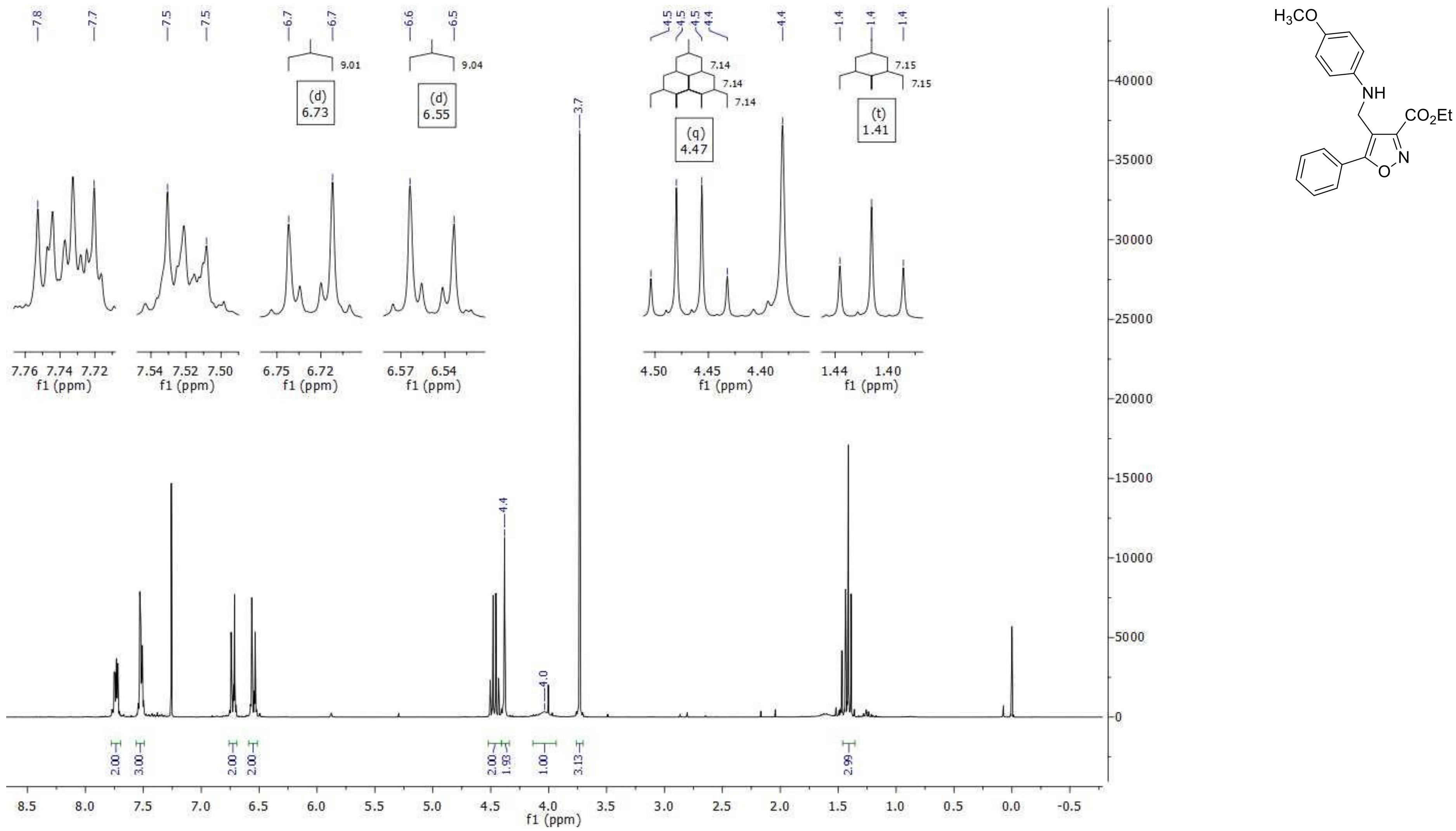

Figure S11 – ^{1}H NMR spectrum of compound **2bc** in $CDCl_3$ at 300.06 MHz.

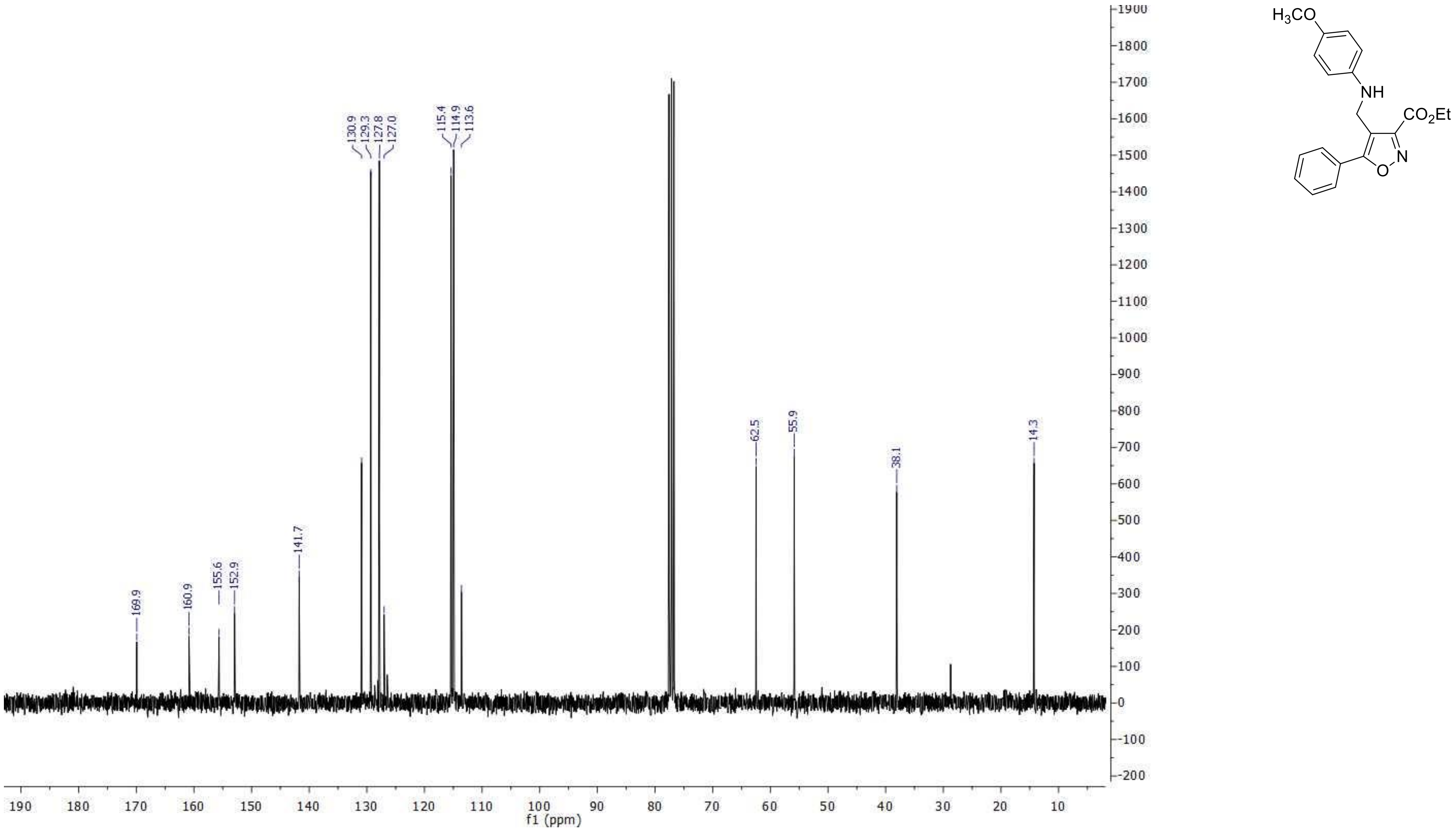

Figure S12 – ^{13}C NMR spectrum of compound **2bc** in $CDCl_3$ at 75.45 MHz.

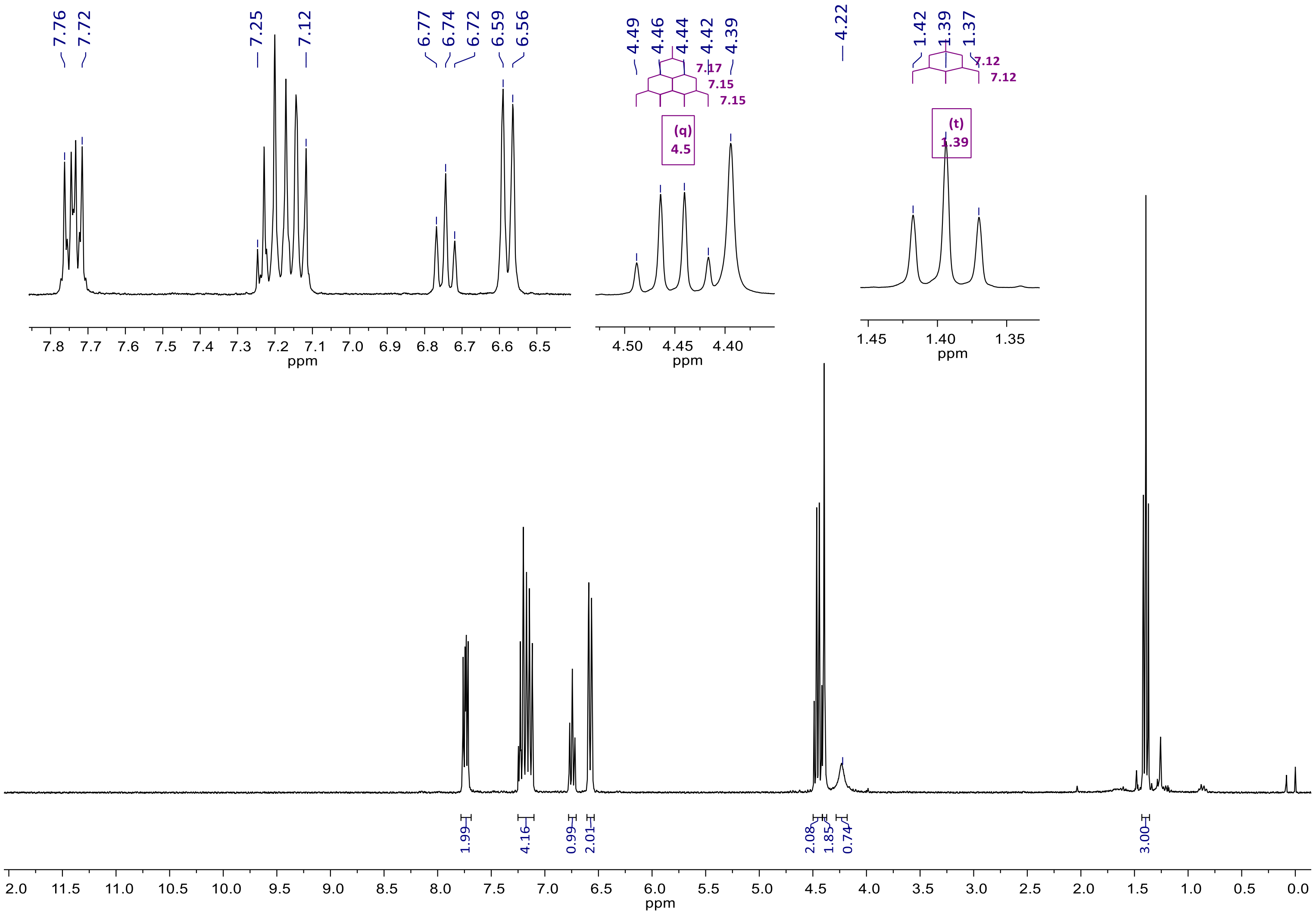

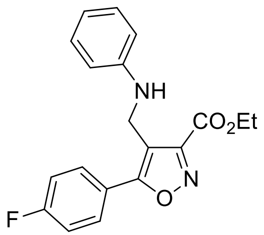

Figure S13 – ^{1}H NMR spectrum of compound **2ca** in $CDCl_3$ at 300.06 MHz.

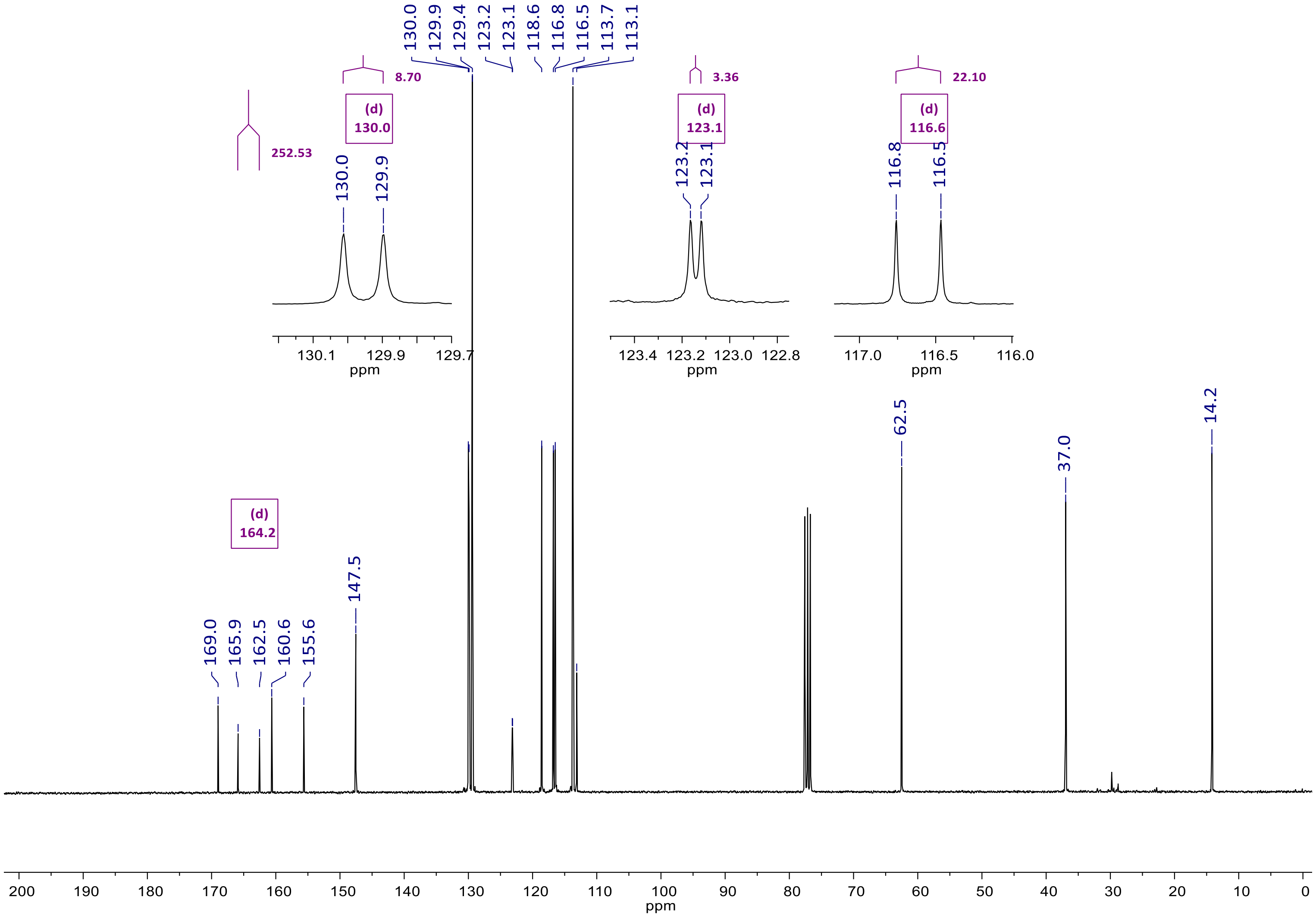

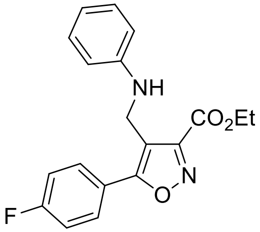

Figure S14 – ^{13}C NMR spectrum of compound **2ca** in $CDCl_3$ at 75.45 MHz.

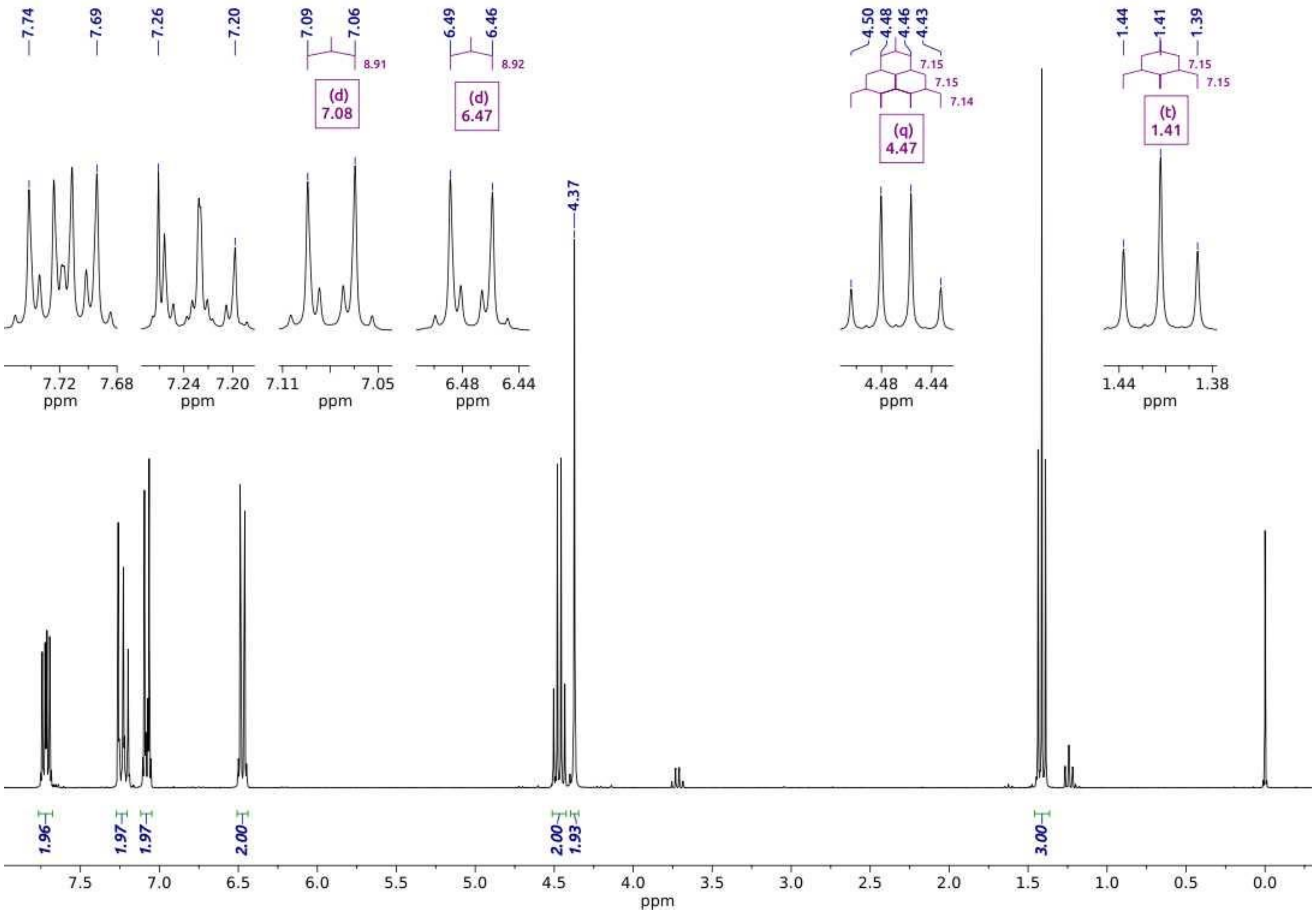

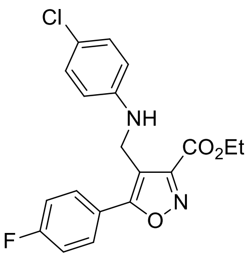

Figure S15 – ^{1}H NMR spectrum of compound **2cb** in $CDCl_3$ at 300.06 MHz.

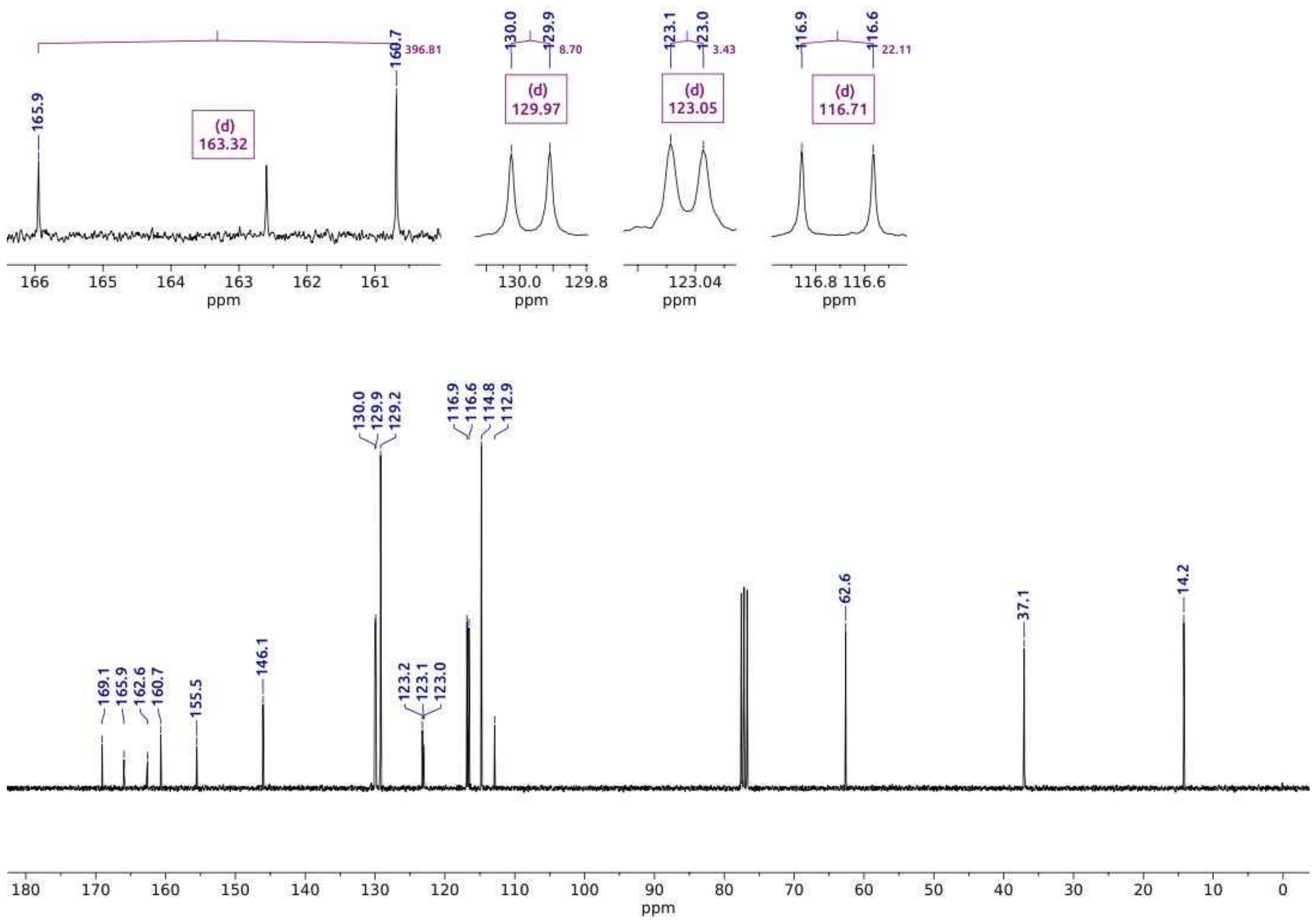

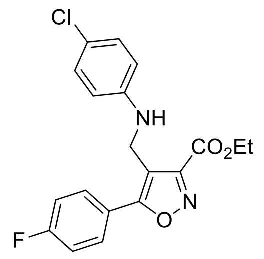

Figure S16 – ^{13}C NMR spectrum of compound **2cb** in $CDCl_3$ at 75.45 MHz.

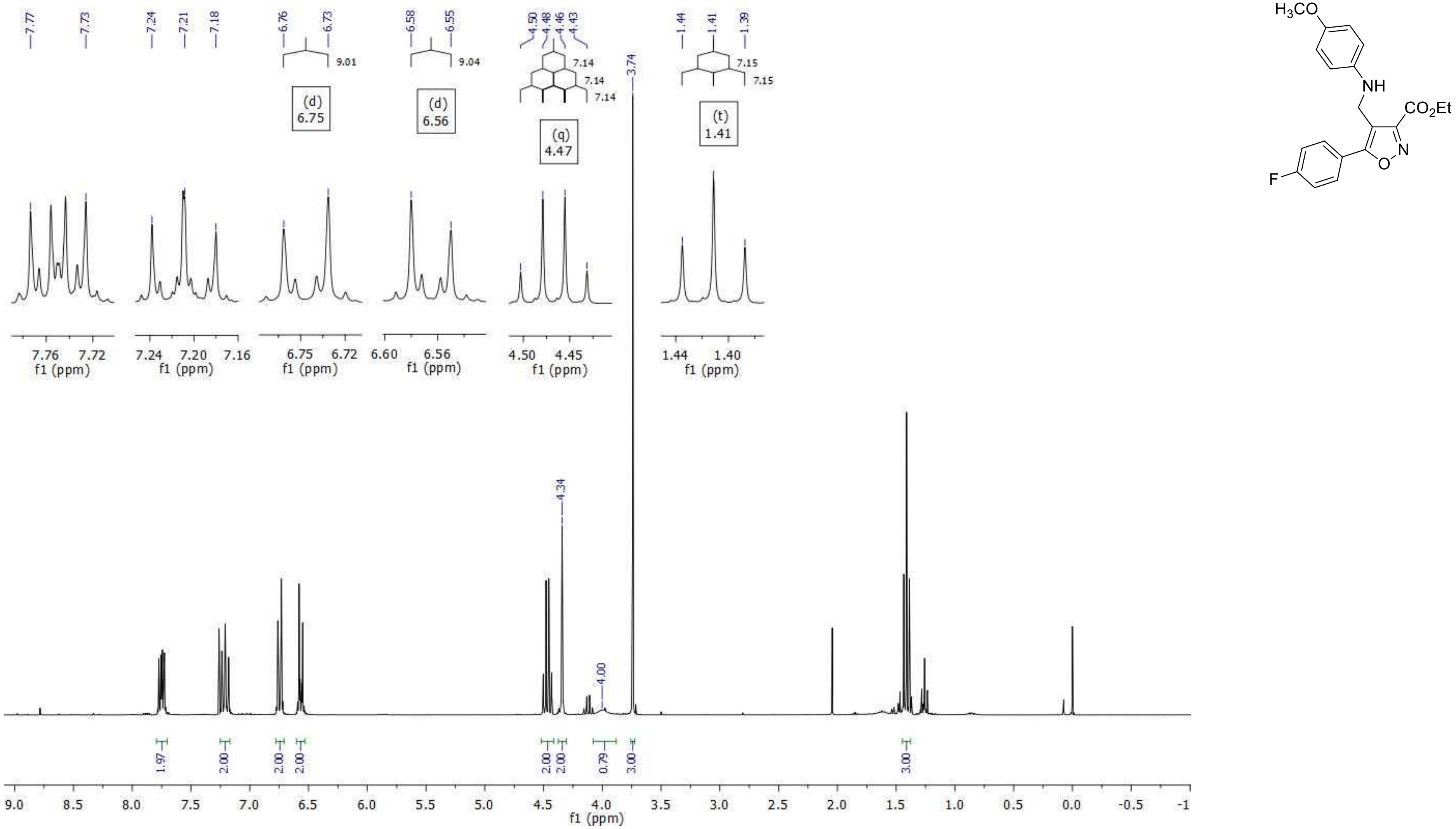

Figure S17 – ^{1}H NMR spectrum of compound **2cc** in $CDCl_3$ at 300.06 MHz.

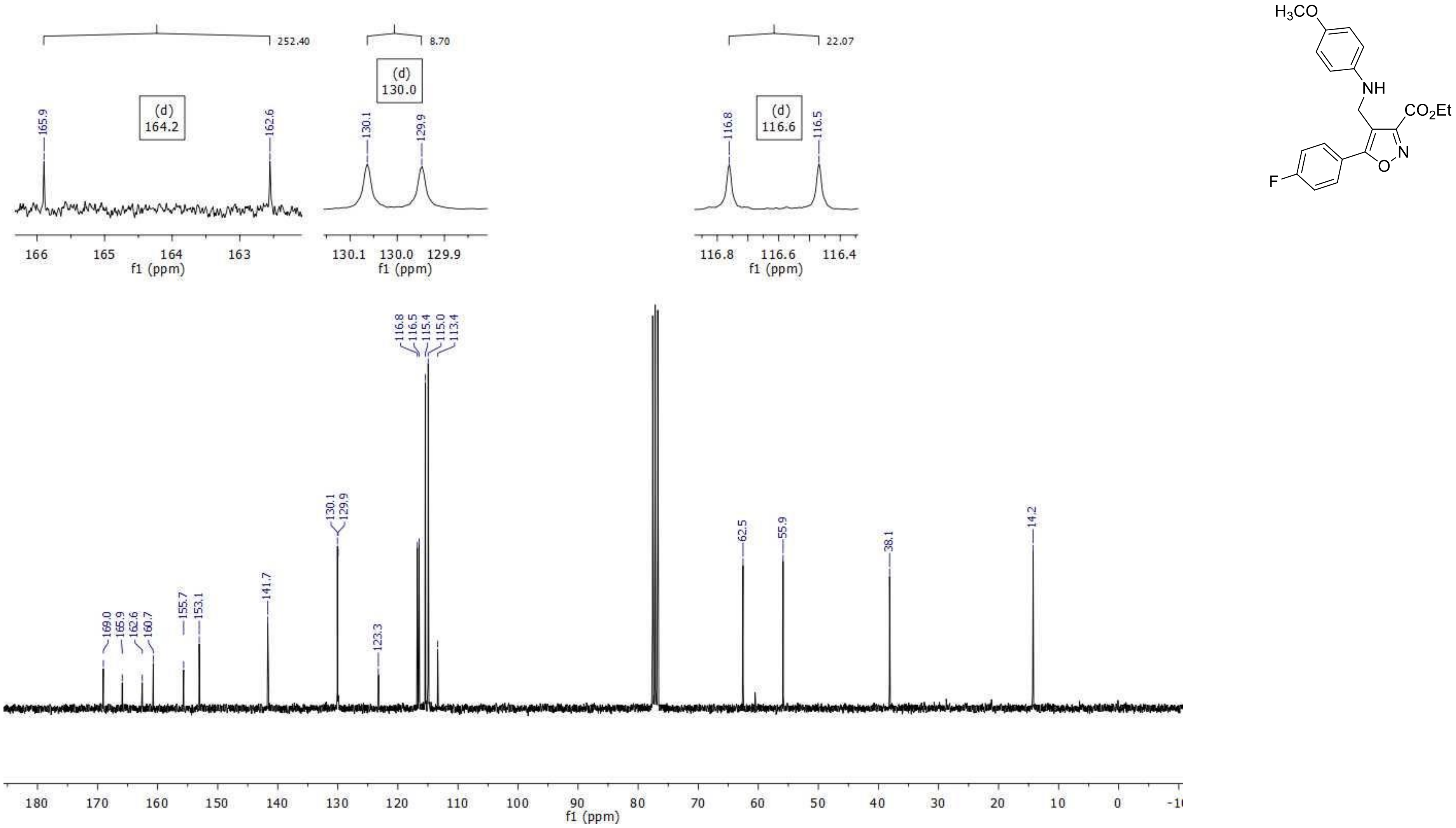

Figure S18 – ^{13}C NMR spectrum of compound **2cc** in $CDCl_3$ at 75.45 MHz.

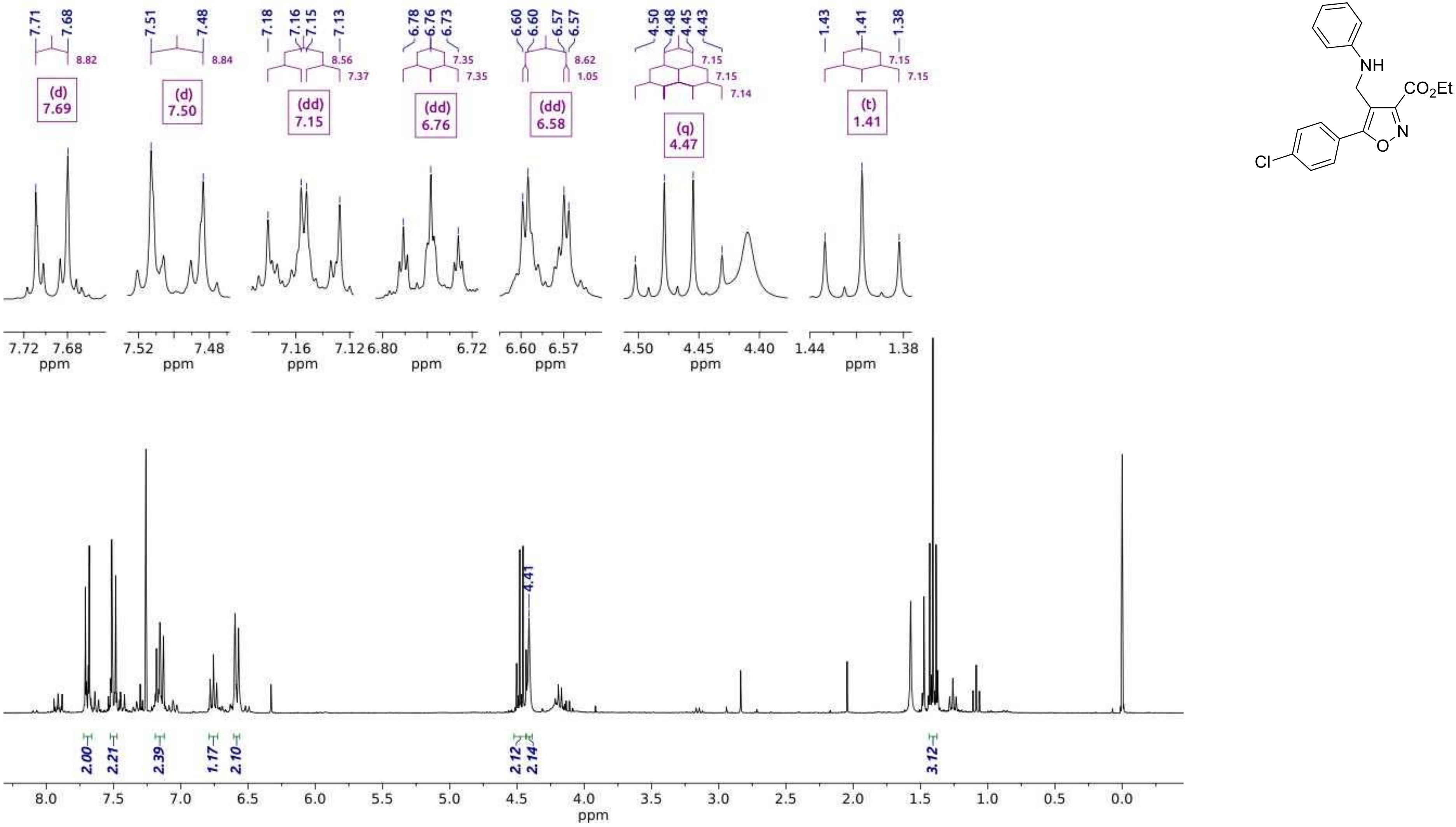

Figure S19 – ^{1}H NMR spectrum of compound **2da** in $CDCl_3$ at 500.13 MHz.

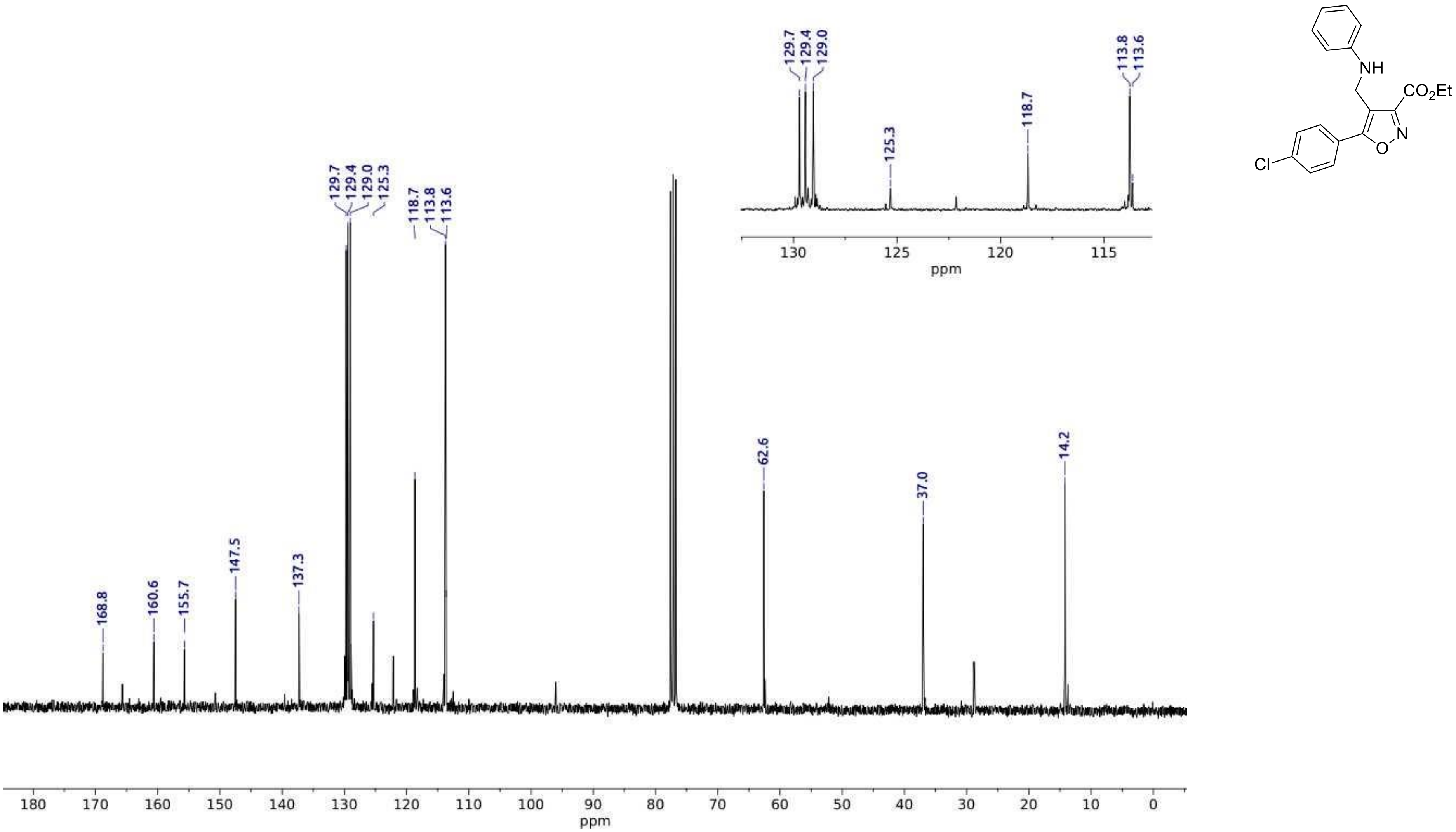

Figure S20 – ^{13}C NMR spectrum of compound **2da** in $CDCl_3$ at 125.76 MHz.

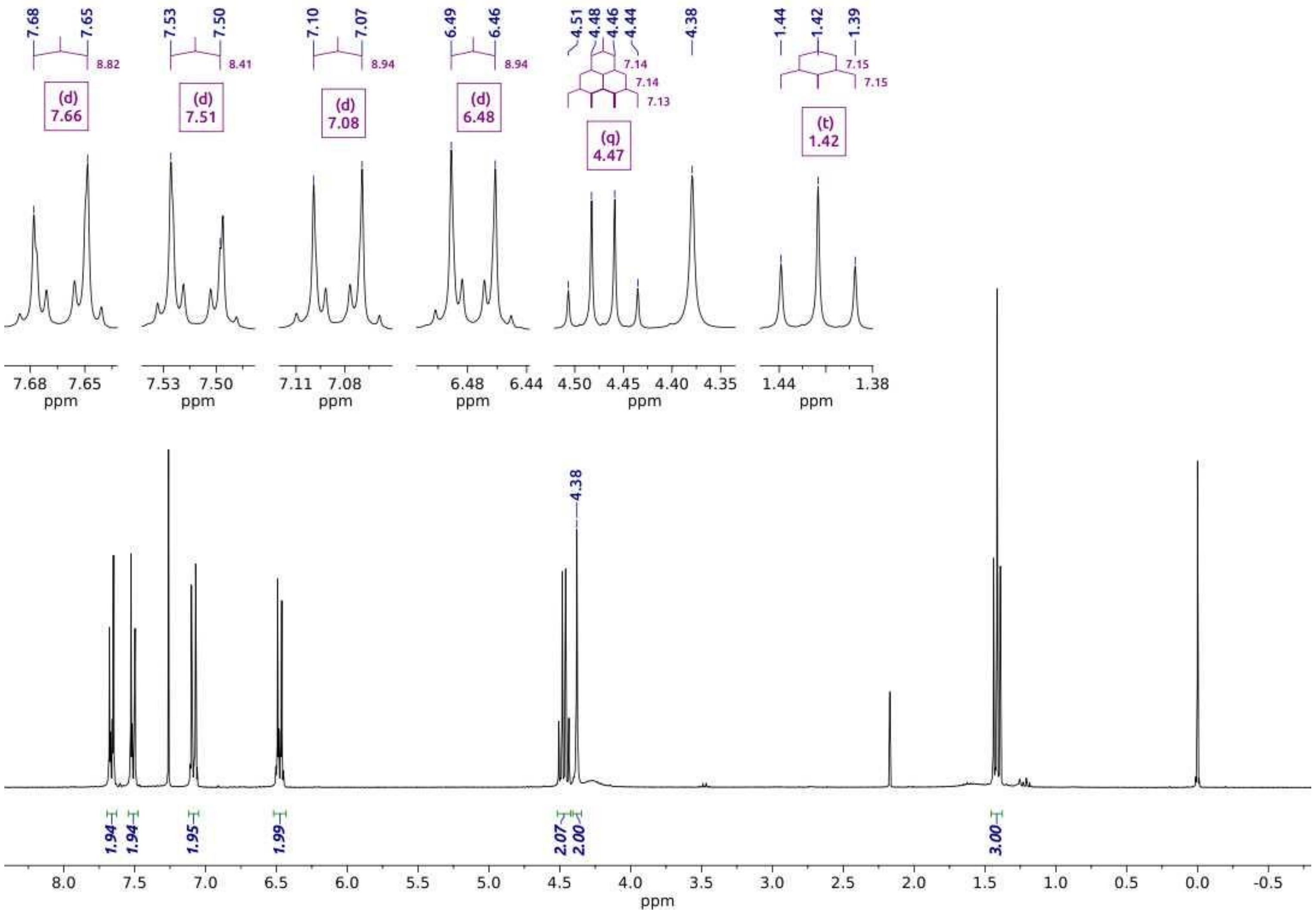

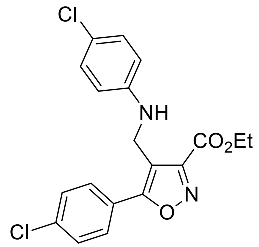

Figure S21 – ^{1}H NMR spectrum of compound **2db** in $CDCl_3$ at 500.13 MHz.

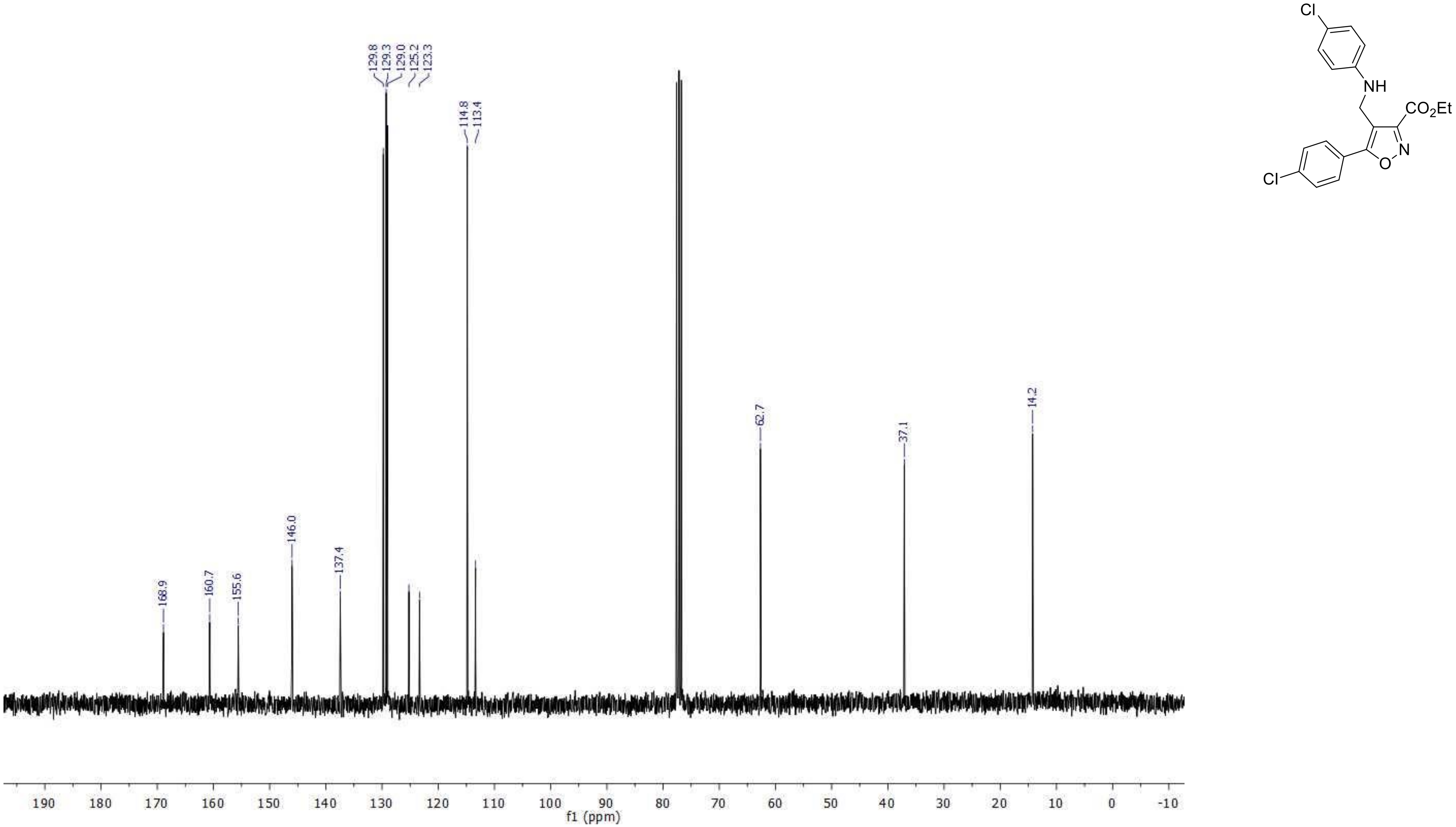

Figure S22 – ^{13}C NMR spectrum of compound **2db** in $CDCl_3$ at 125.76 MHz.

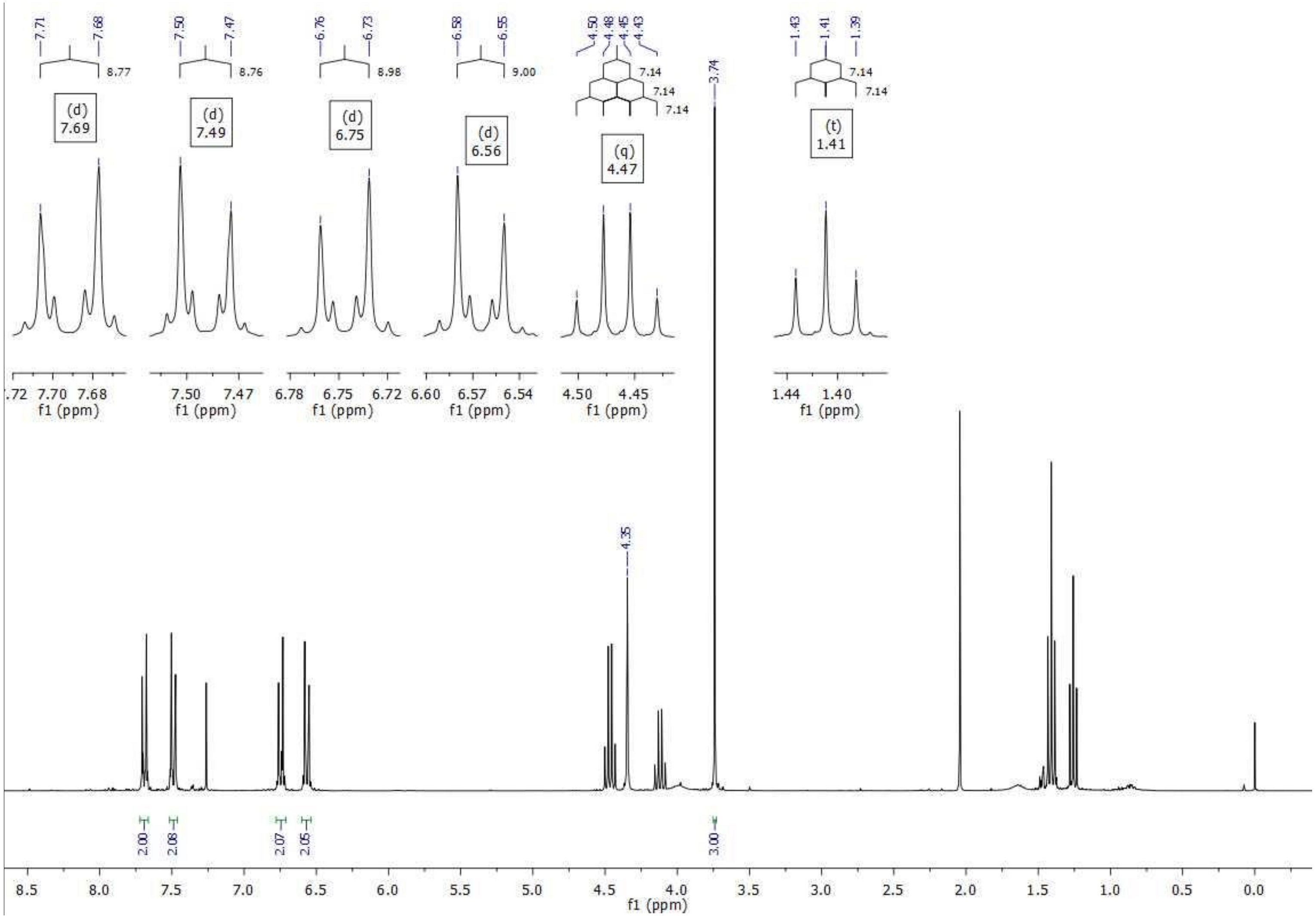

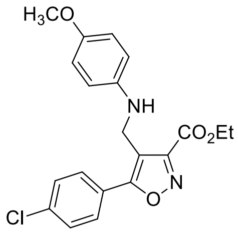

Figure S23 – ^{1}H NMR spectrum of compound **2dc** in $CDCl_3$ at 300.06 MHz.

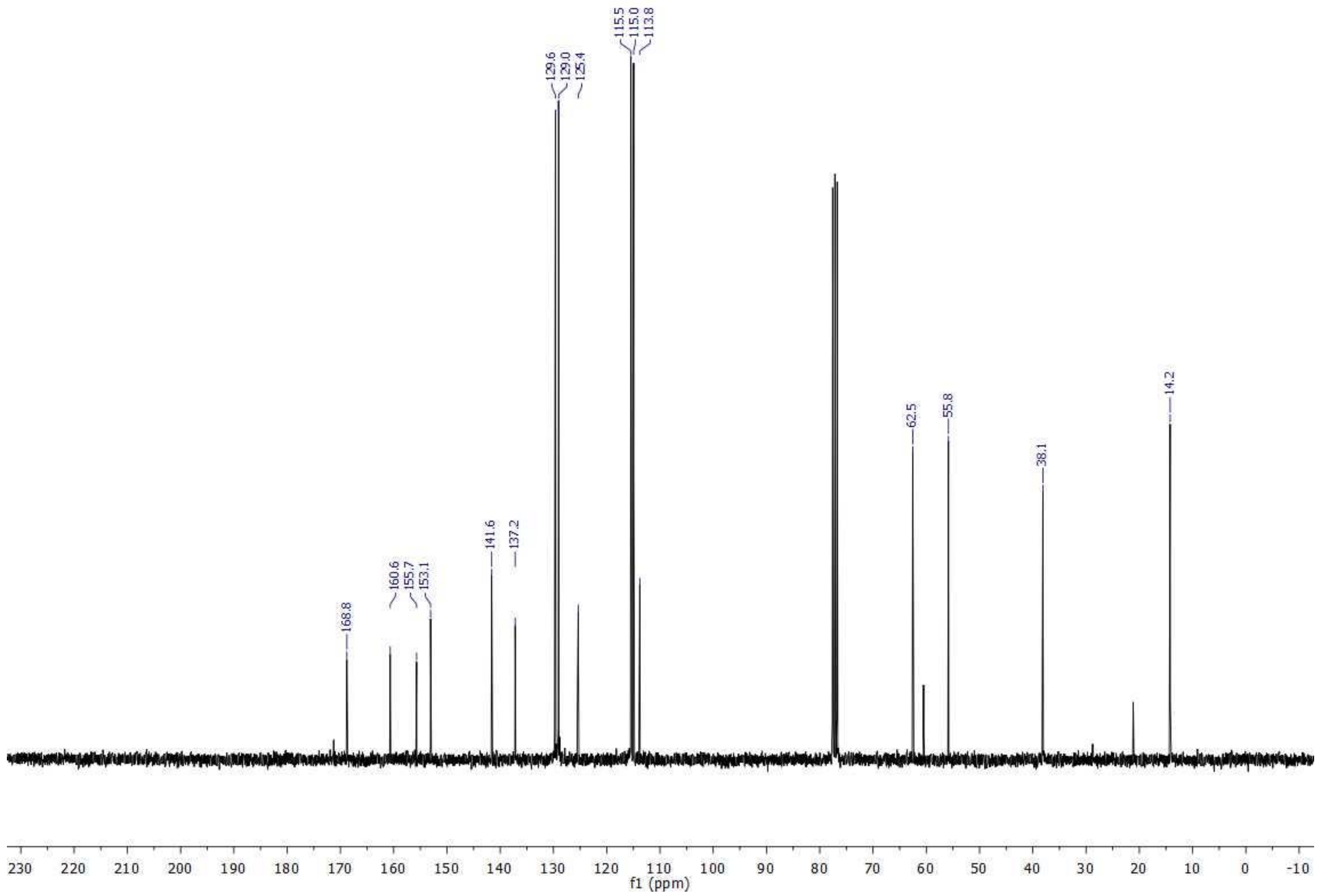

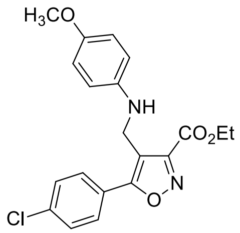

Figure S24 – ^{13}C NMR spectrum of compound **2dc** in $CDCl_3$ at 75.45 MHz.

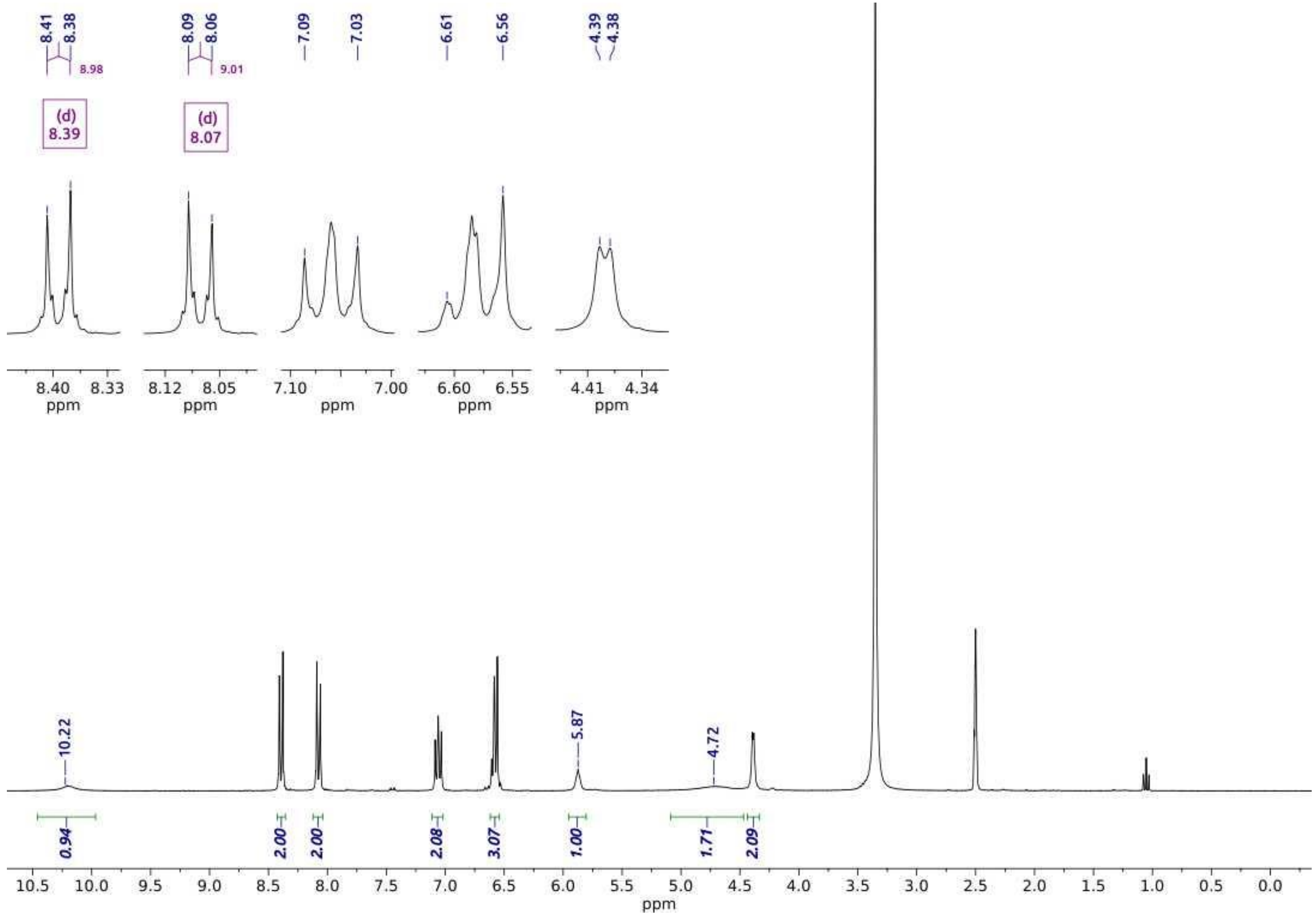

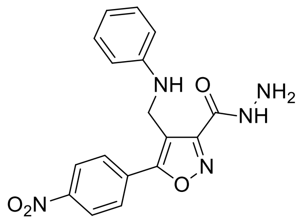

Figure S25 – ^{1}H NMR spectrum of compound **3aa** in DMSO-d_6 at 300.06 MHz.

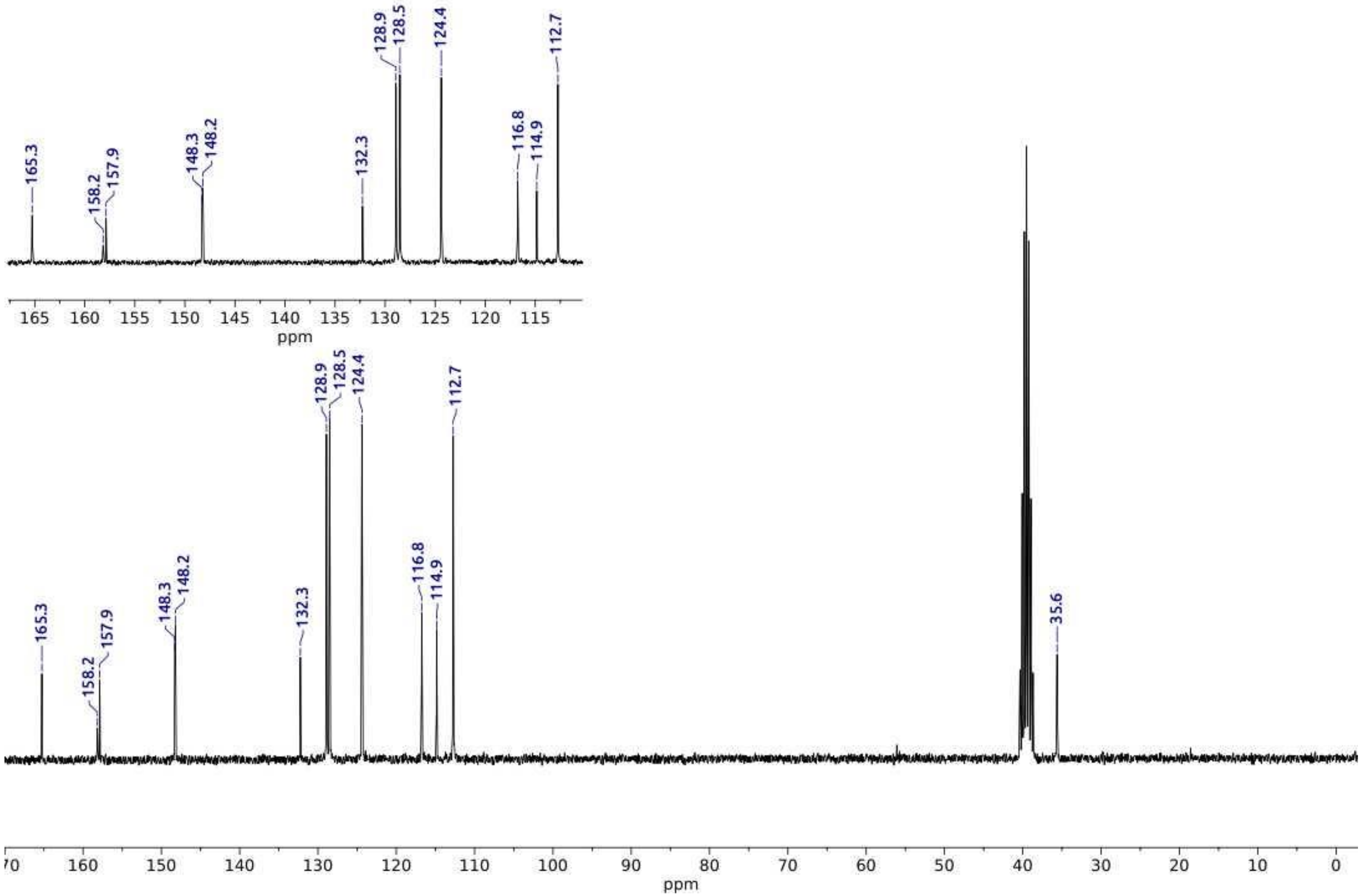

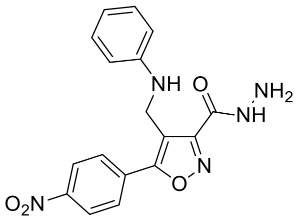

Figure S26 – ^{13}C NMR spectrum of compound **3aa** in DMSO-d_6 at 75.45 MHz.

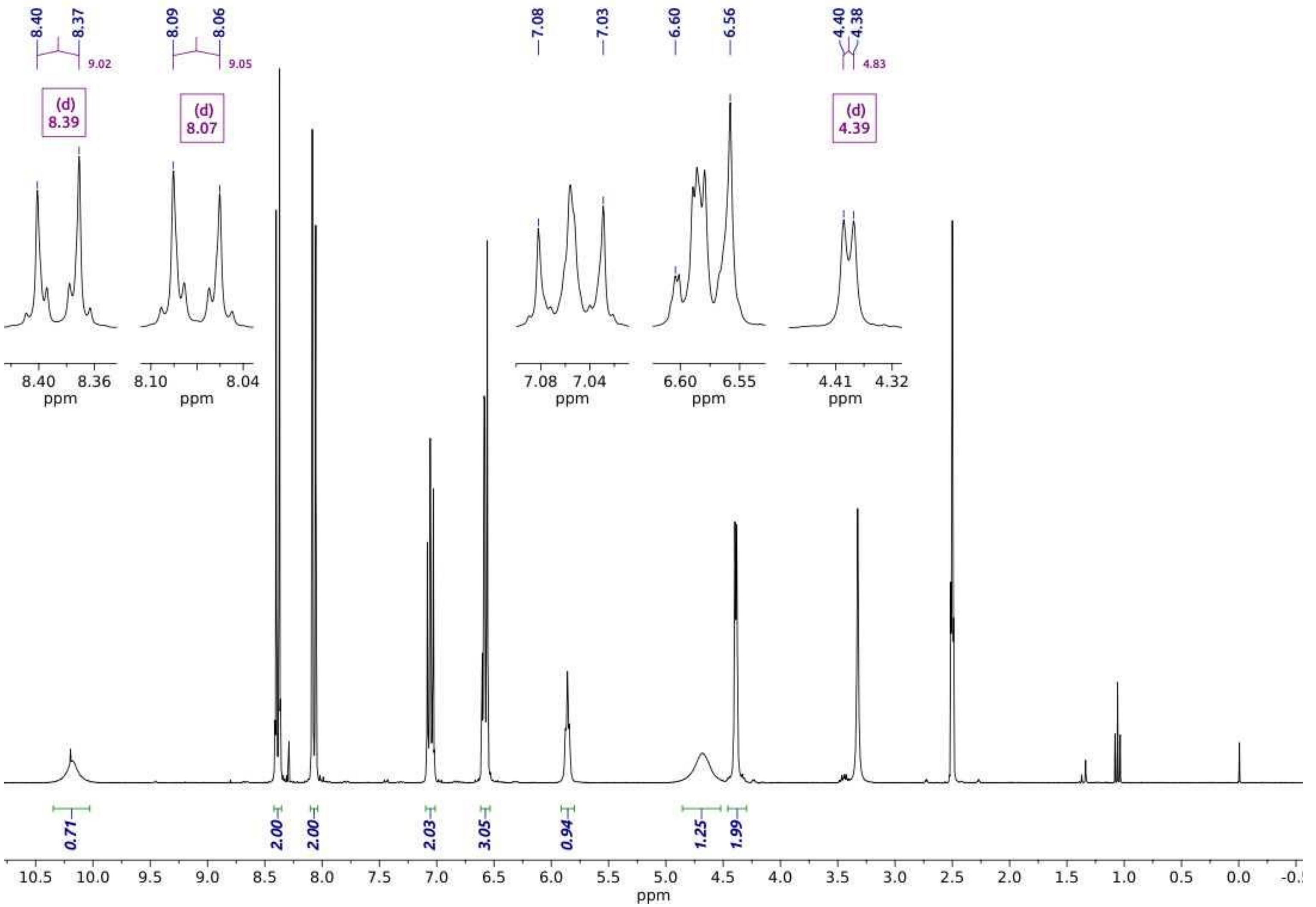

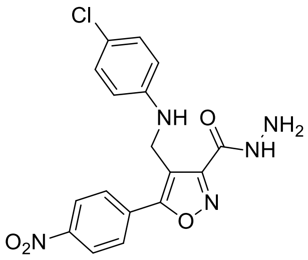

Figure S27 – ^{1}H NMR spectrum of compound **3ab** in DMSO-d_6 at 300.06 MHz.

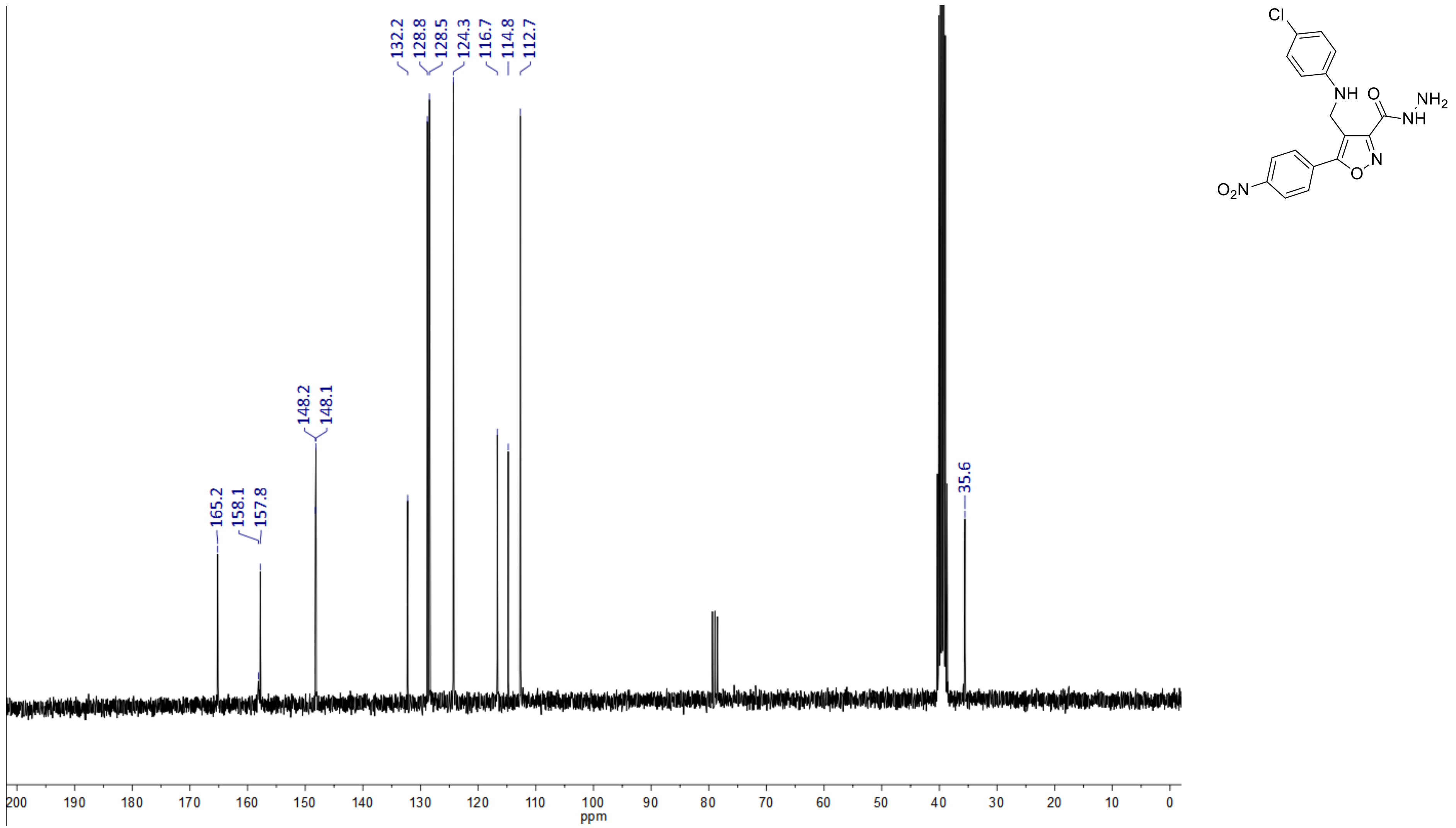

Figure S28 – ^{13}C NMR spectrum of compound **3ab** in DMSO-d_6 at 75.45 MHz.

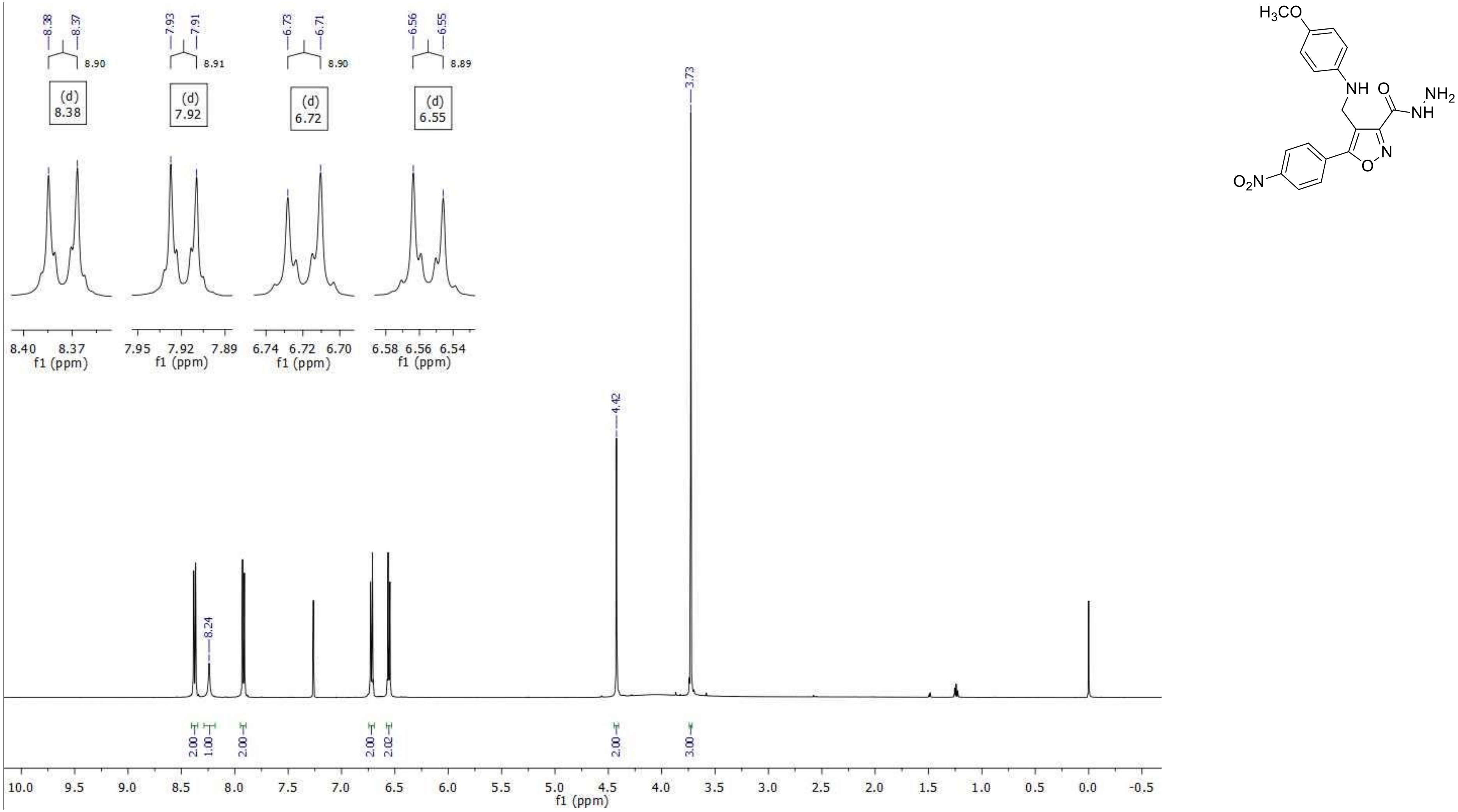

Figure S29 – ^{1}H NMR spectrum of compound **3ac** in $CDCl_3$ at 300.06 MHz.

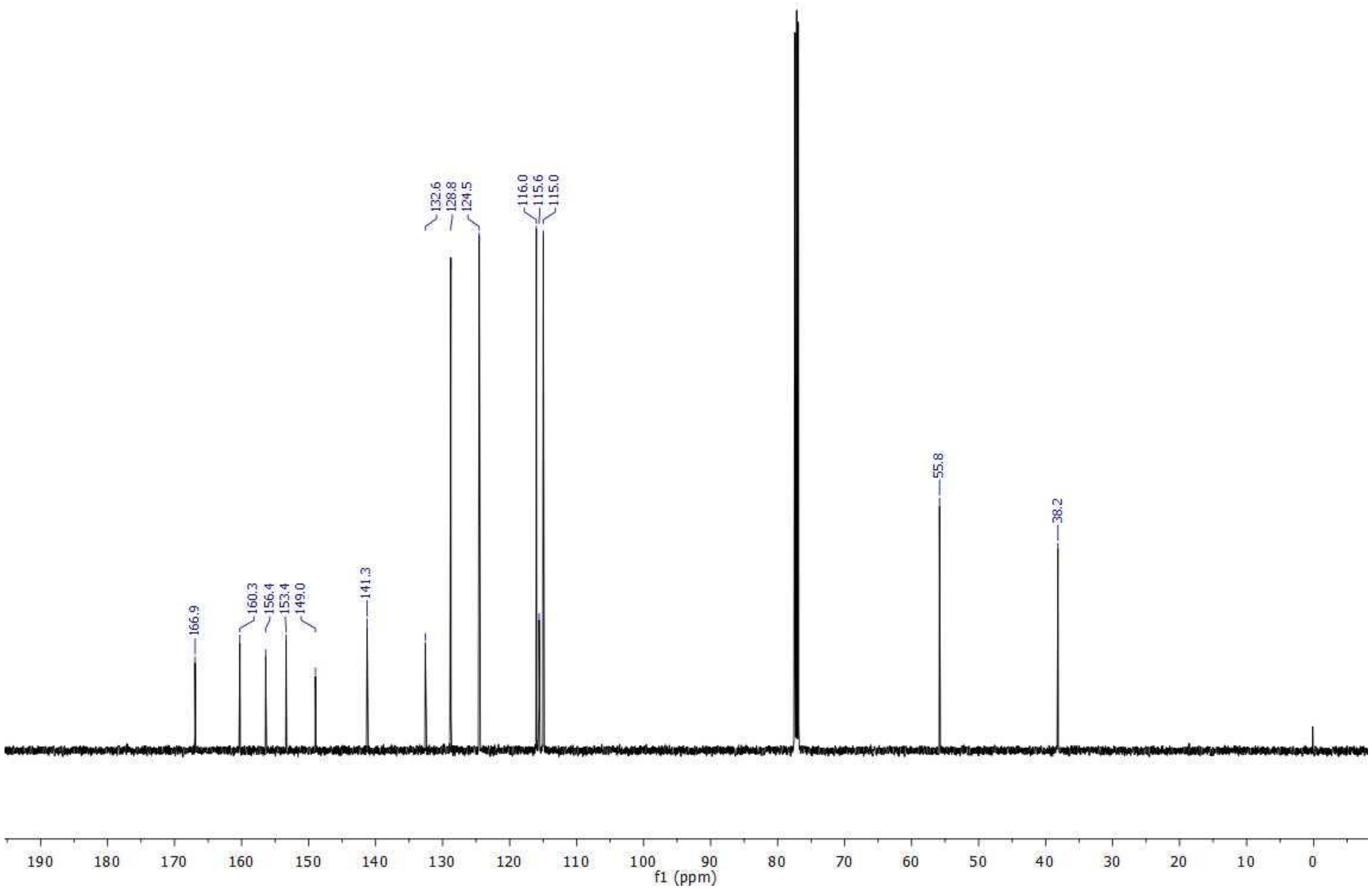

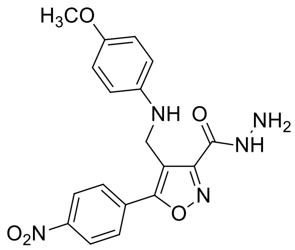

Figure S30 – ^{13}C NMR spectrum of compound **3ac** in $CDCl_3$ at 75.45 MHz.

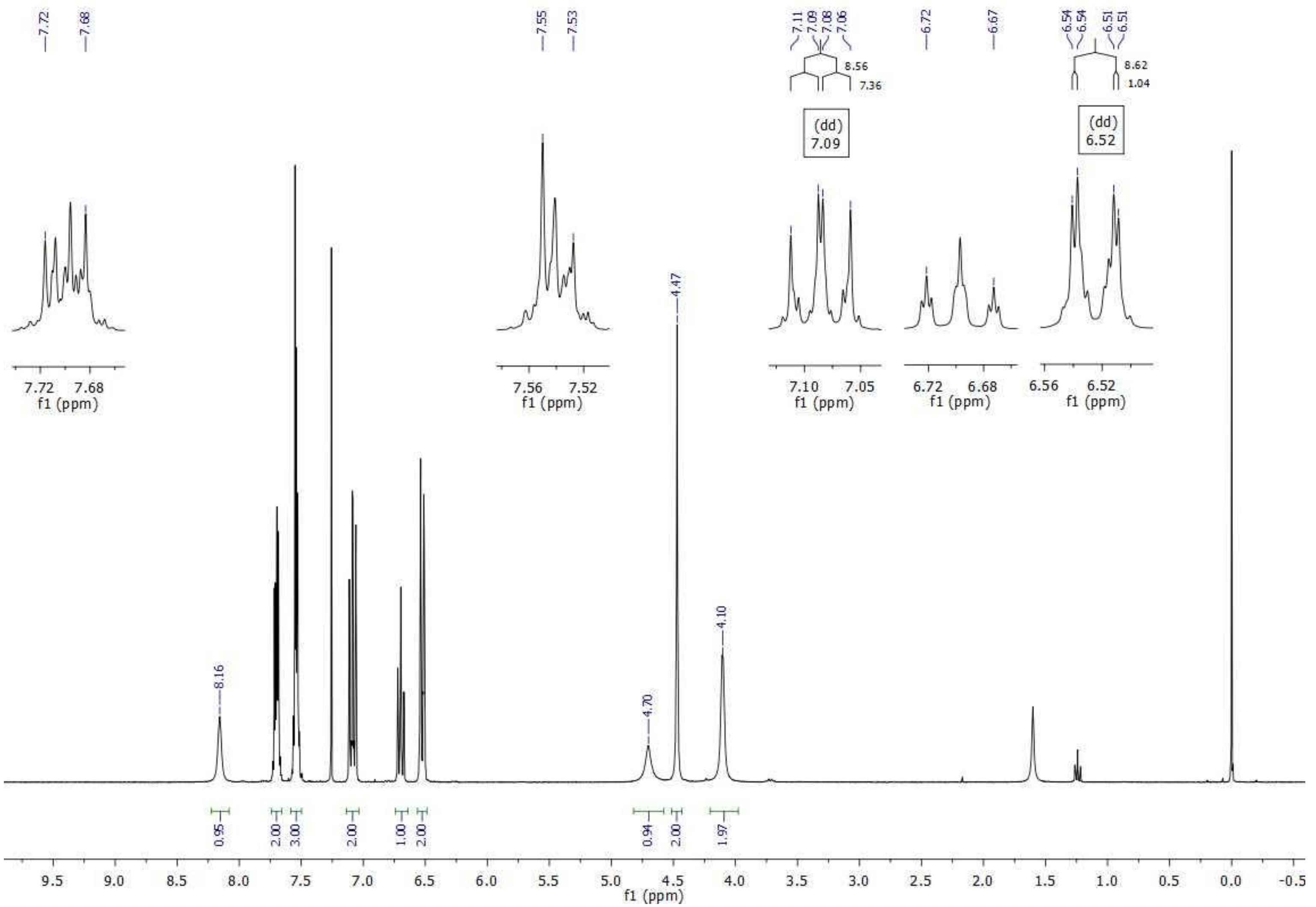

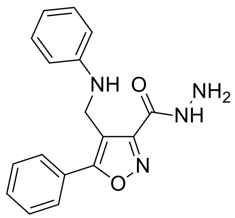

Figure S31 – ^{1}H NMR spectrum of compound **3ba** in $CDCl_3$ at 300.06 MHz.

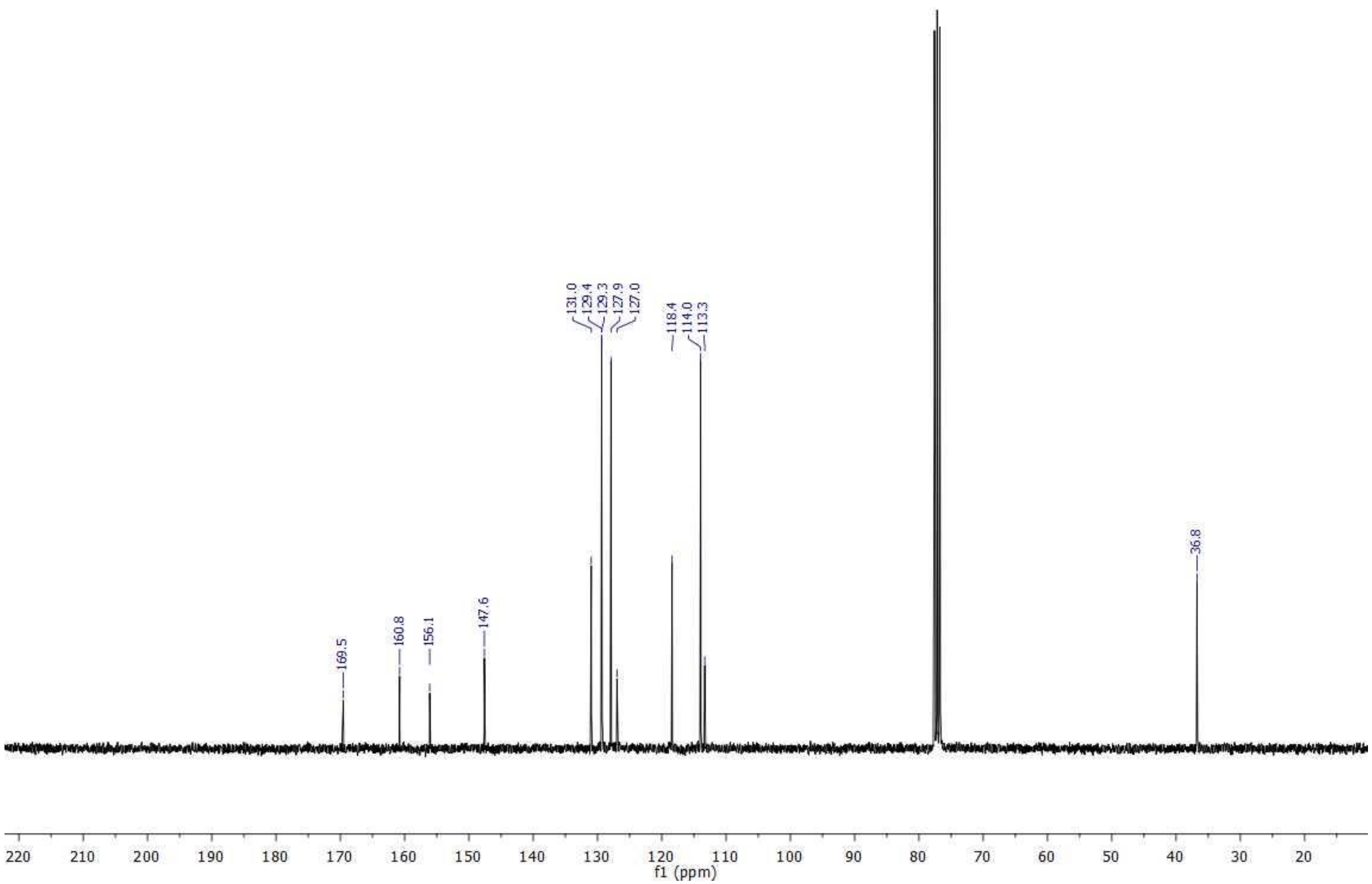

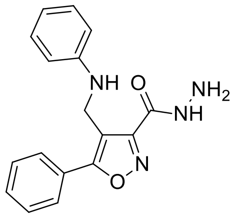

Figure S32 – ^{13}C NMR spectrum of compound **3ba** in $CDCl_3$ at 75.45 MHz.

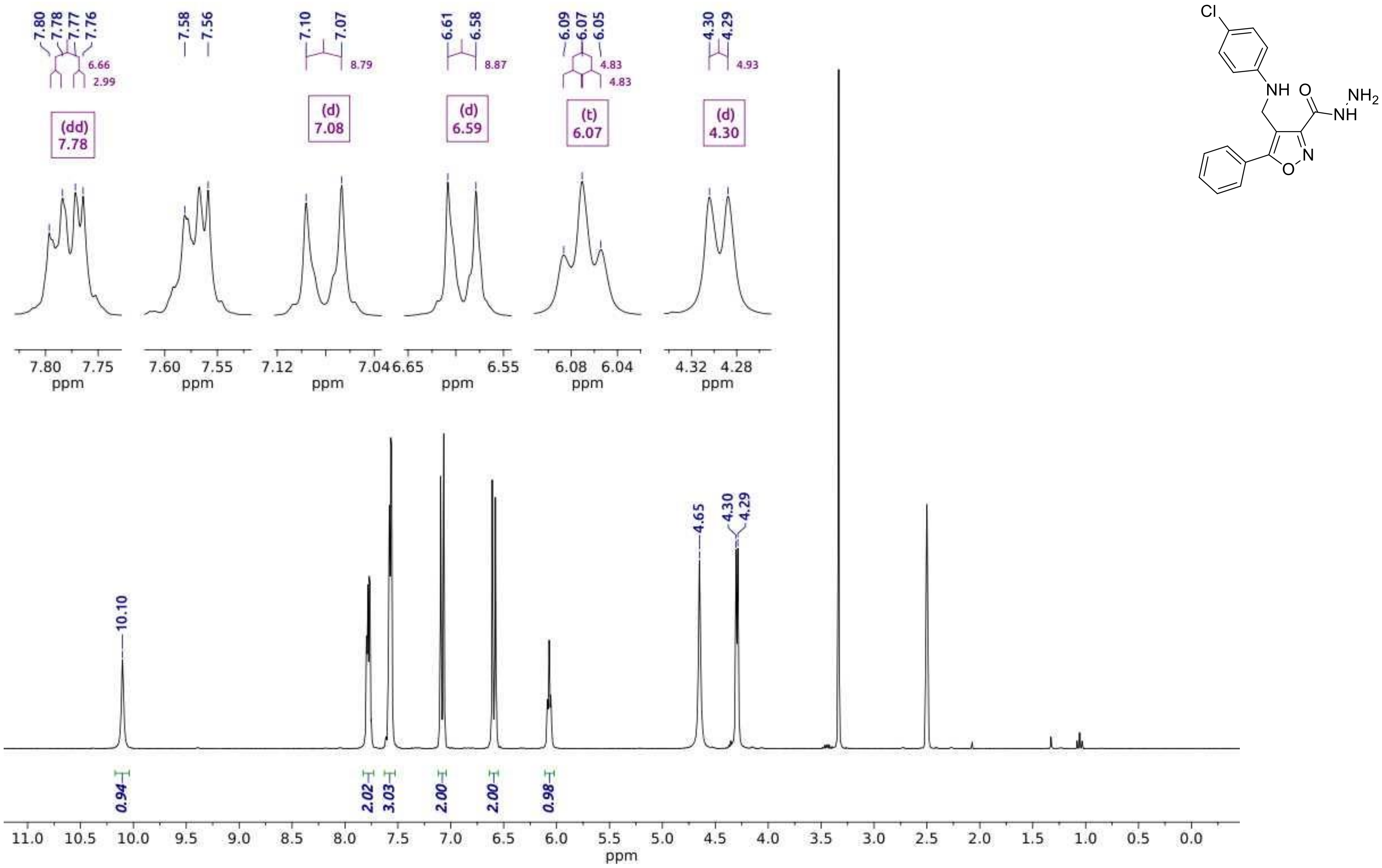

Figure S33 – ^{1}H NMR spectrum of compound **3bb** in DMSO-d_6 at 300.06 MHz.

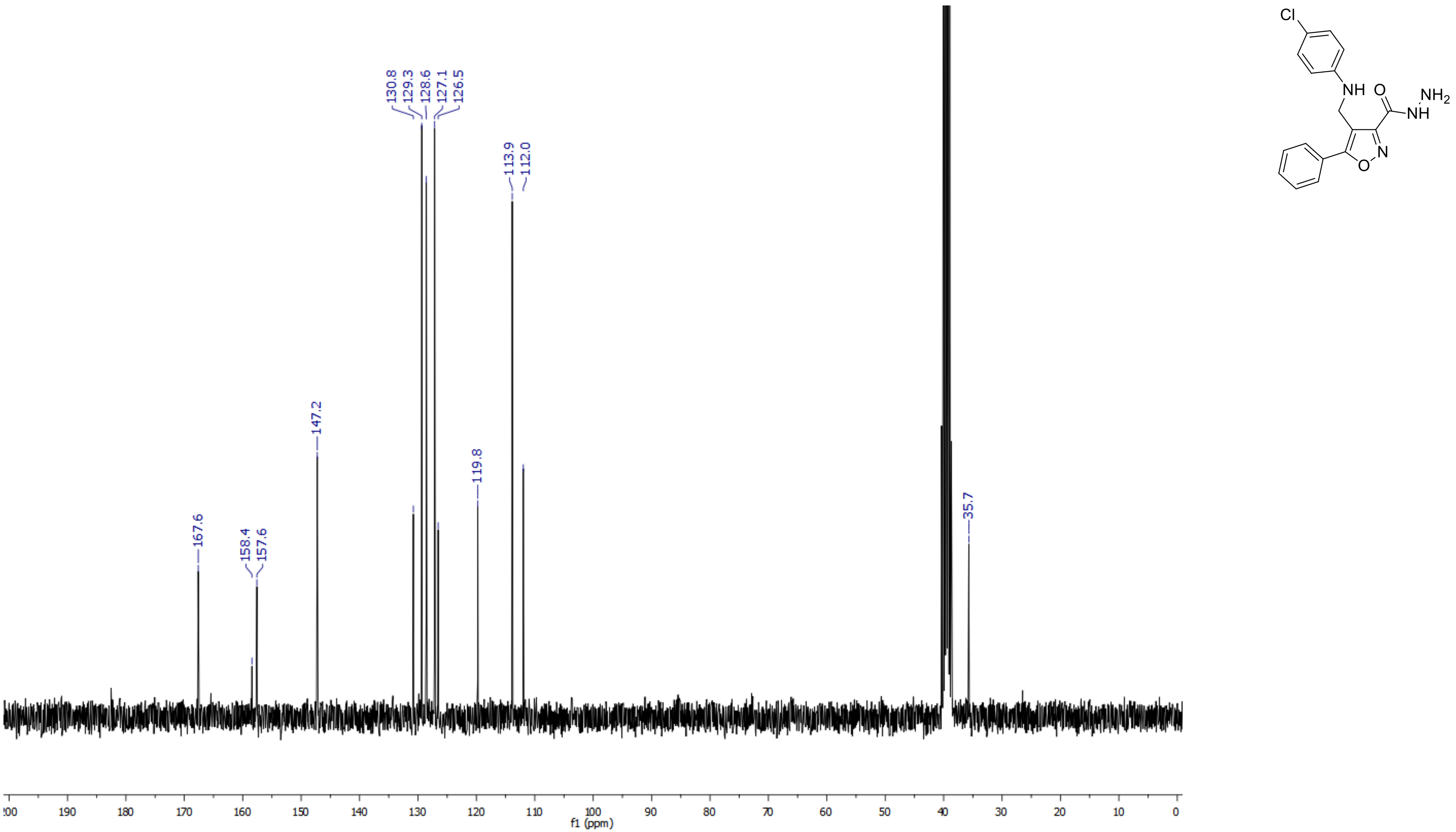

Figure S34 – ^{13}C NMR spectrum of compound **3bb** in DMSO-d_6 at 75.45 MHz.

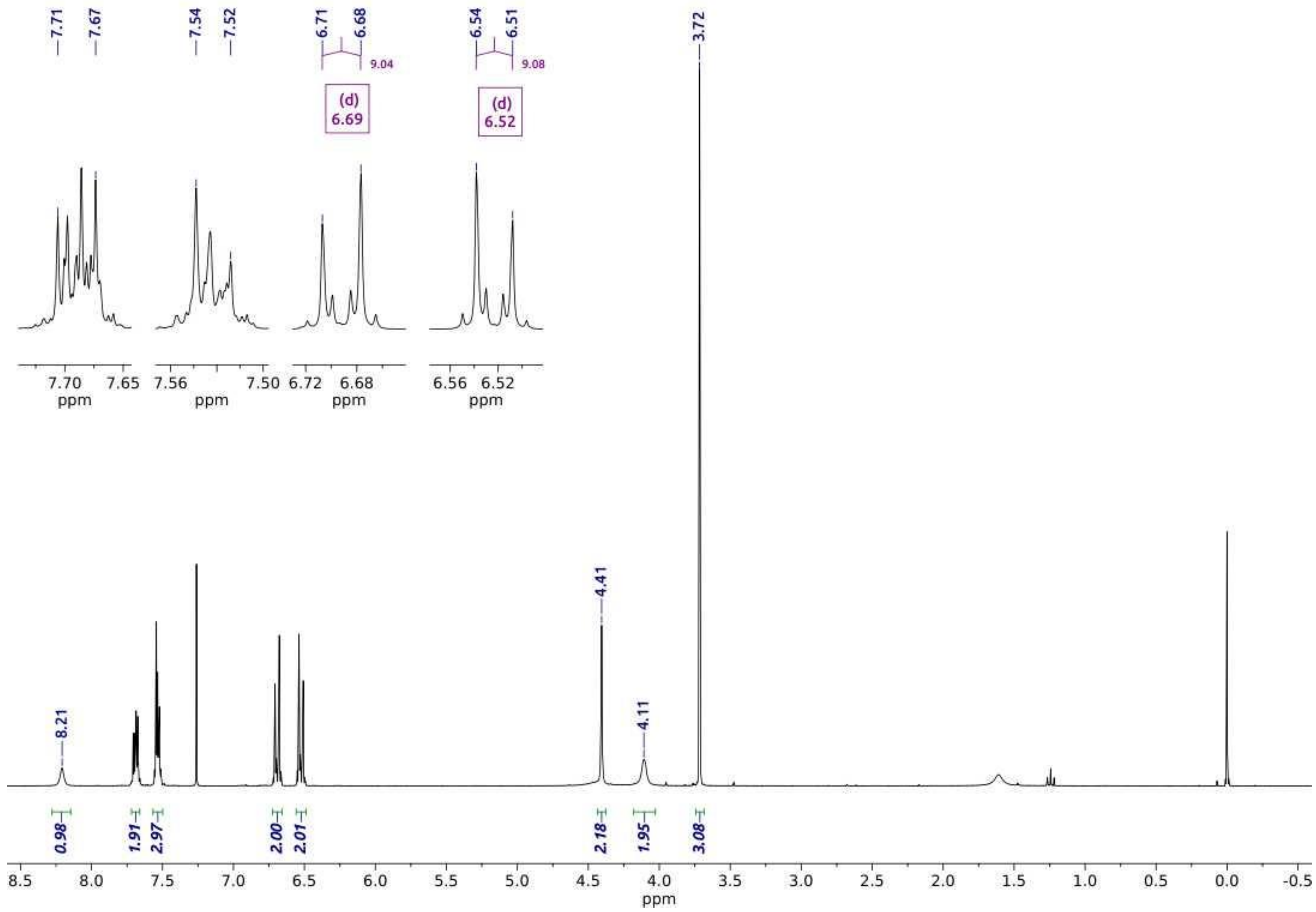

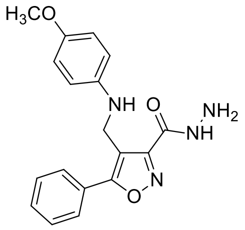

Figure S35 – ^{1}H NMR spectrum of compound **3bc** in $CDCl_3$ at 300.06 MHz.

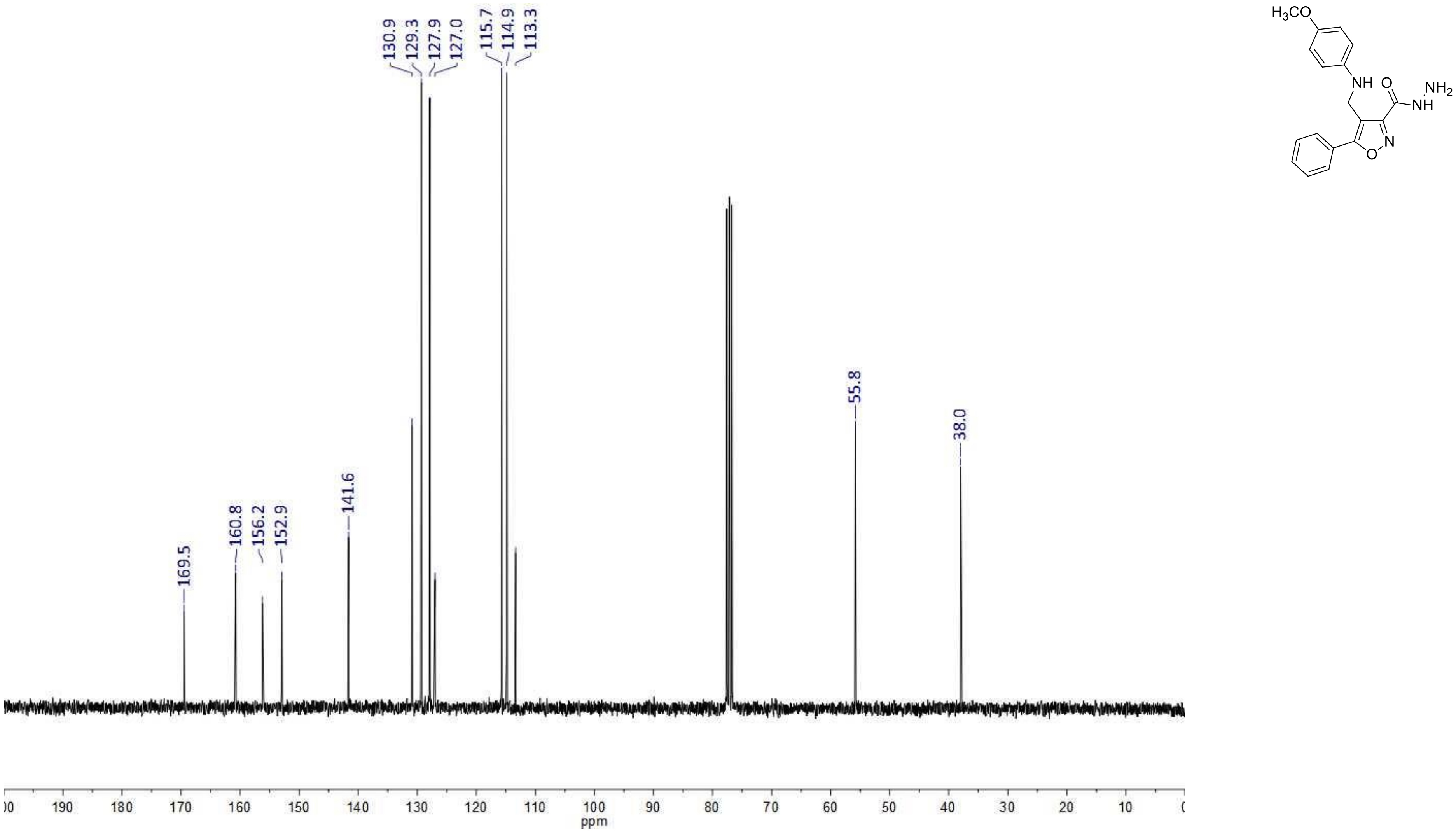

Figure S36 – ^{13}C NMR spectrum of compound **3bc** in $CDCl_3$ at 75.45 MHz.

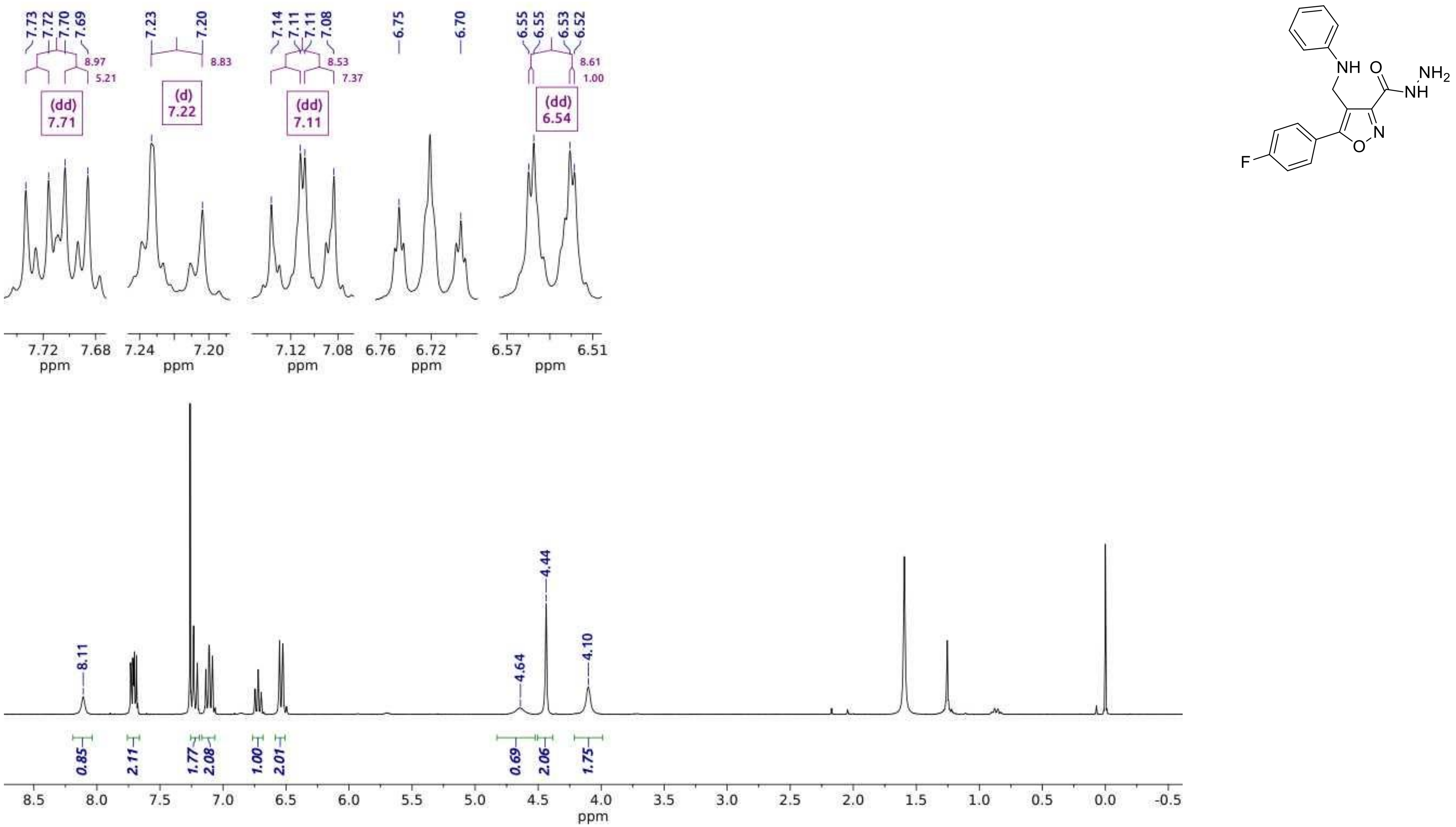

Figure S37 – ^{1}H NMR spectrum of compound **3ca** in $CDCl_3$ at 300.06 MHz.

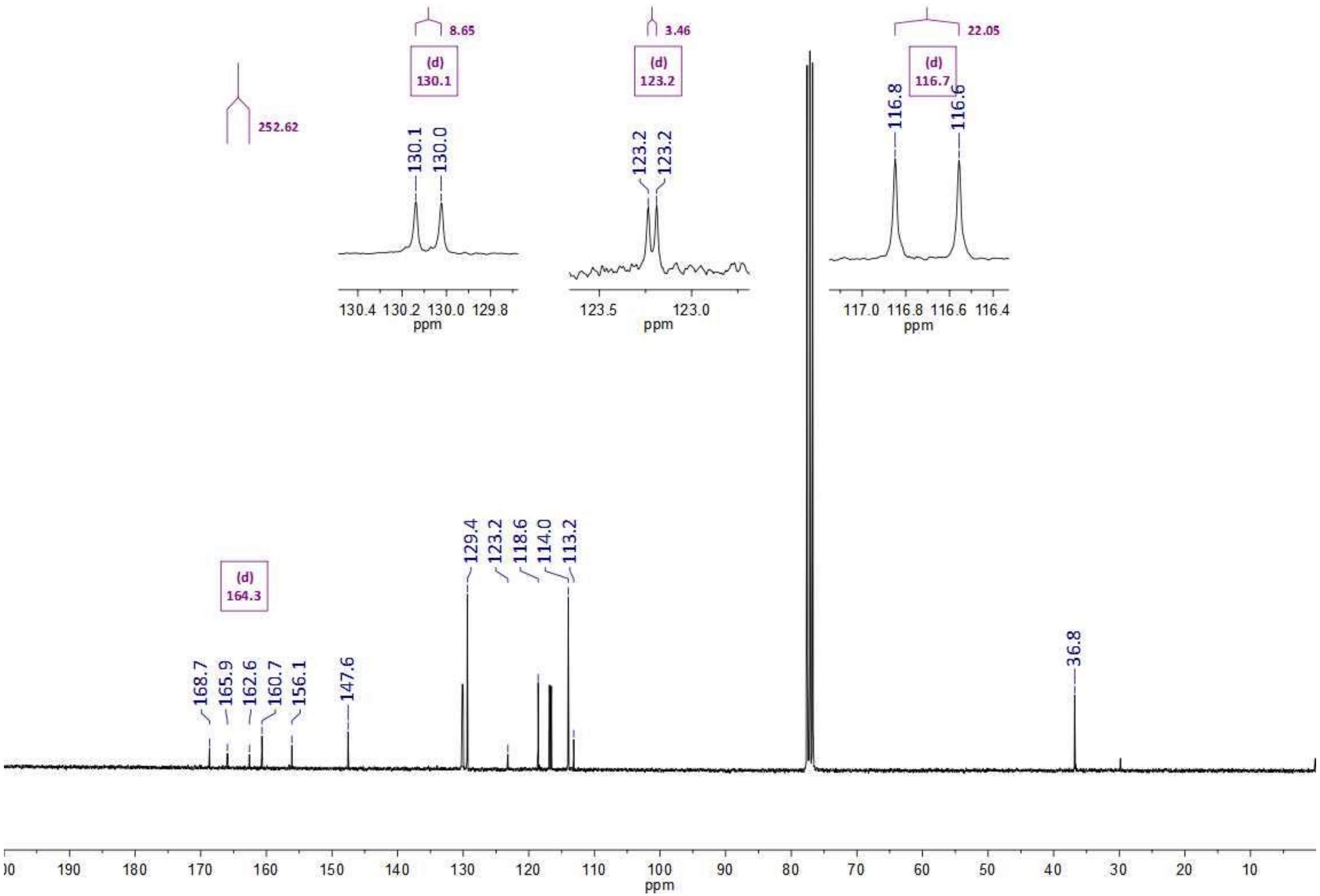

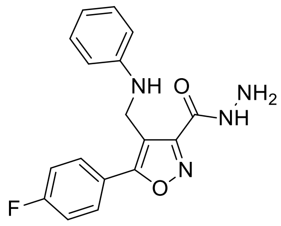

Figure S38 – ^{13}C NMR spectrum of compound **3ca** in $CDCl_3$ at 75.45 MHz.

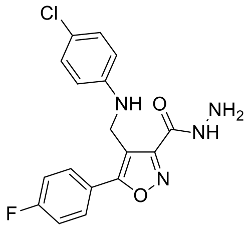

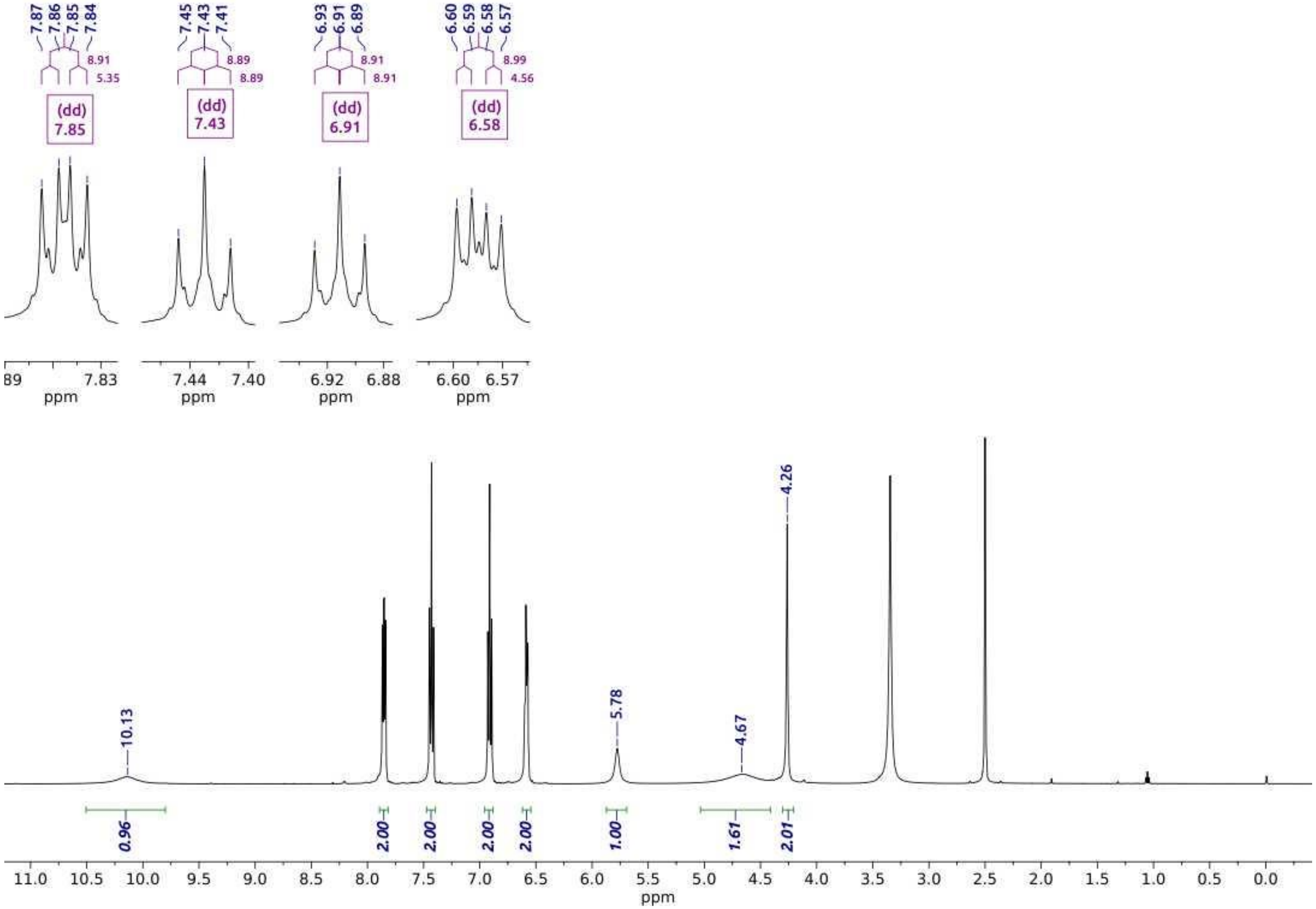

Figure S39 – ^{1}H NMR spectrum of compound **3cb** in DMSO-d_6 at 300.06 MHz.

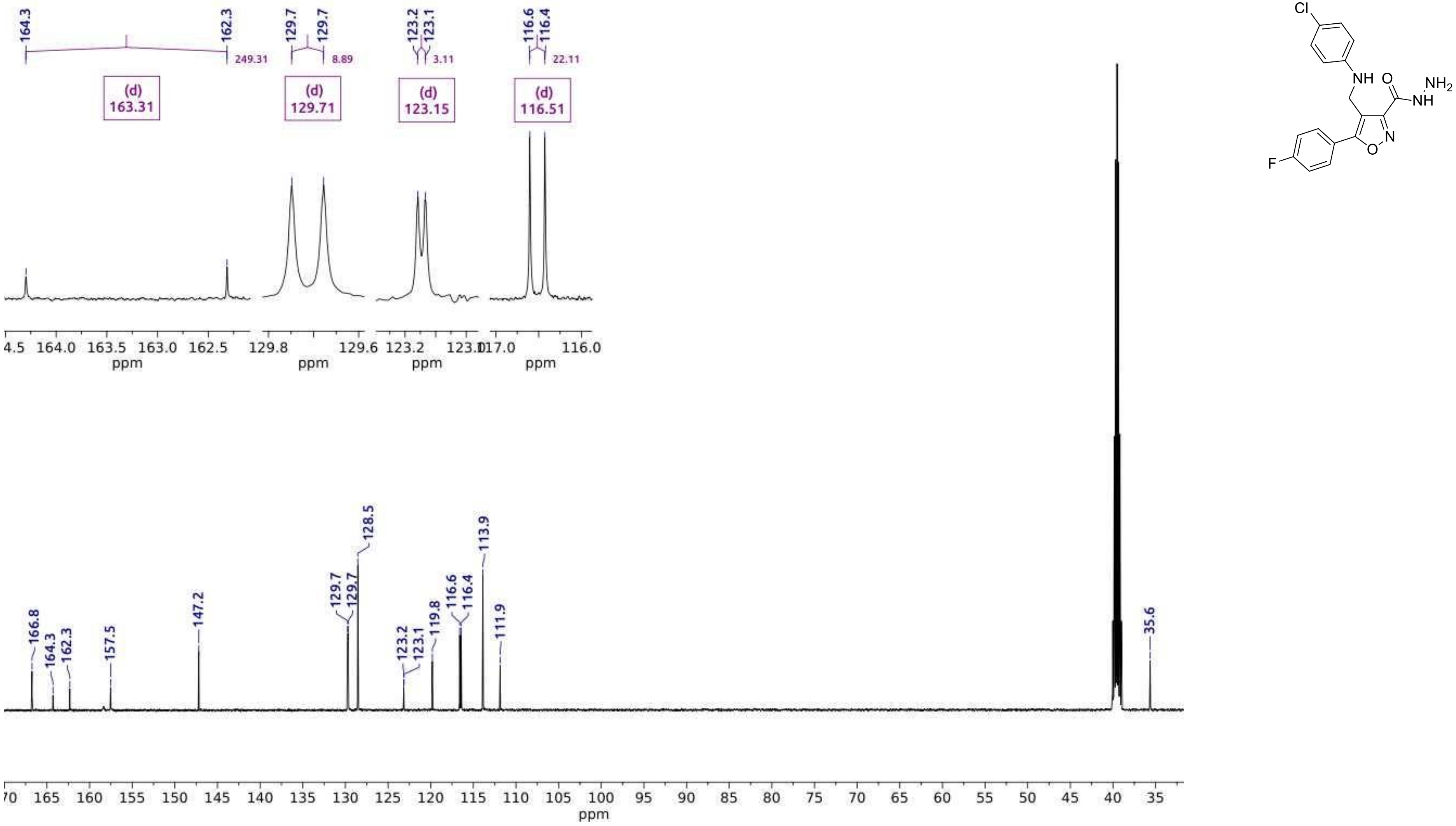

Figure S40 – ^{13}C NMR spectrum of compound **3cb** in DMSO-d_6 at 75.45 MHz.

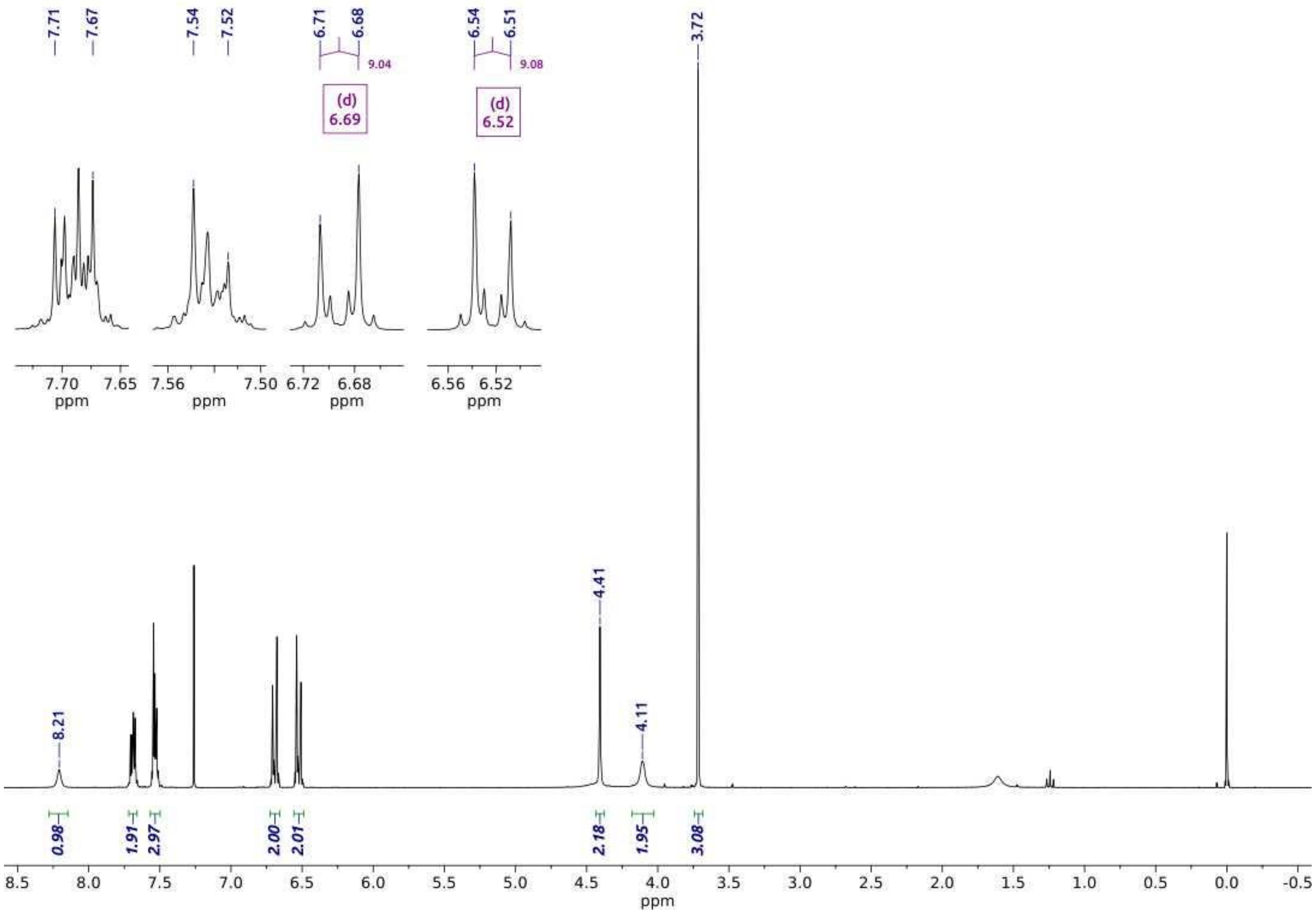

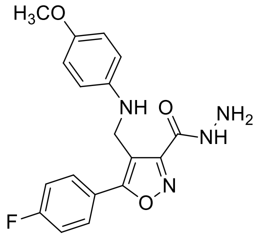

Figure S41 – ^{1}H NMR spectrum of compound **3cc** in $CDCl_3$ at 300.06 MHz.

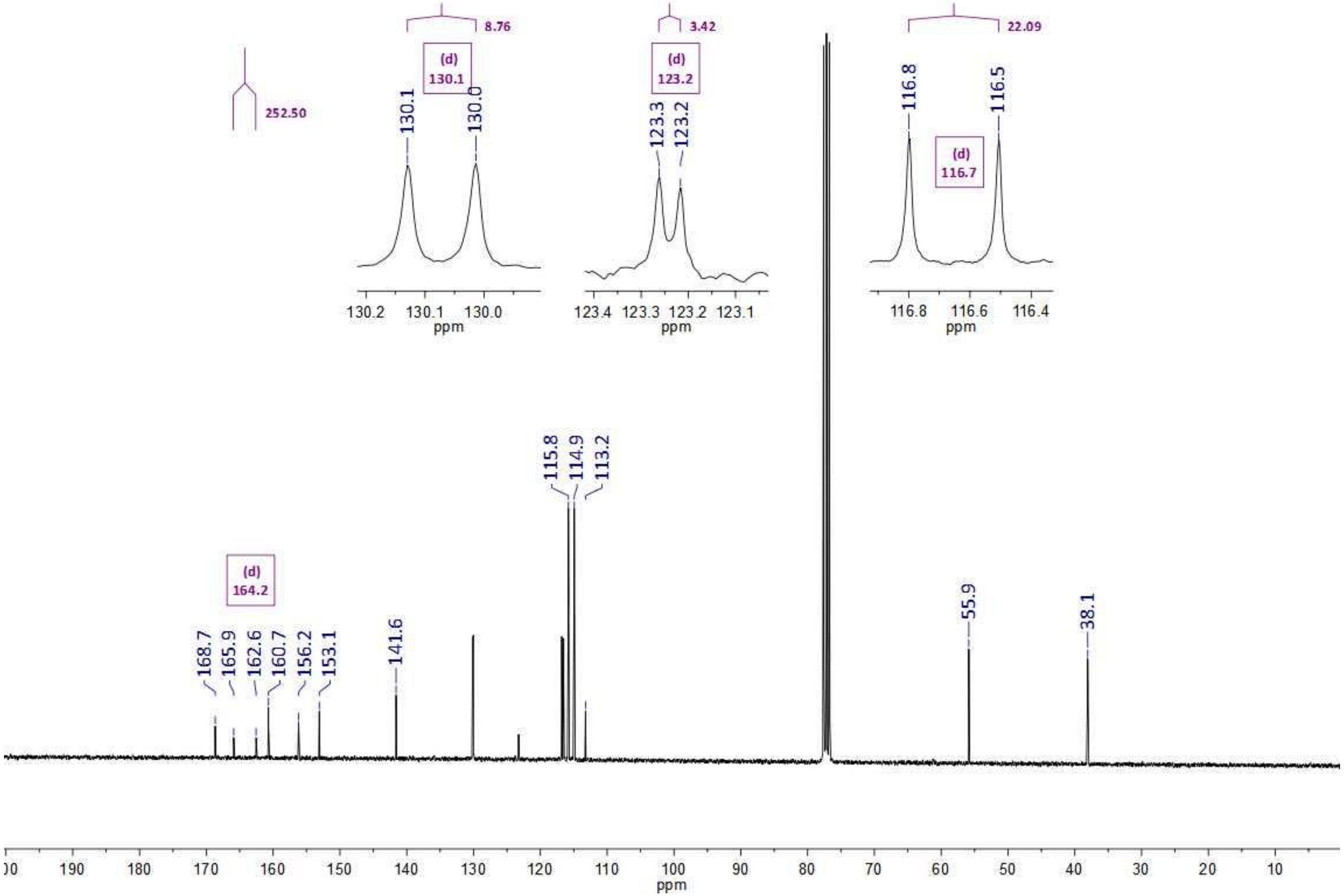

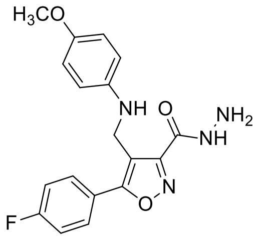

Figure S42 – ^{13}C NMR spectrum of compound **3cc** in $CDCl_3$ at 75.45 MHz.

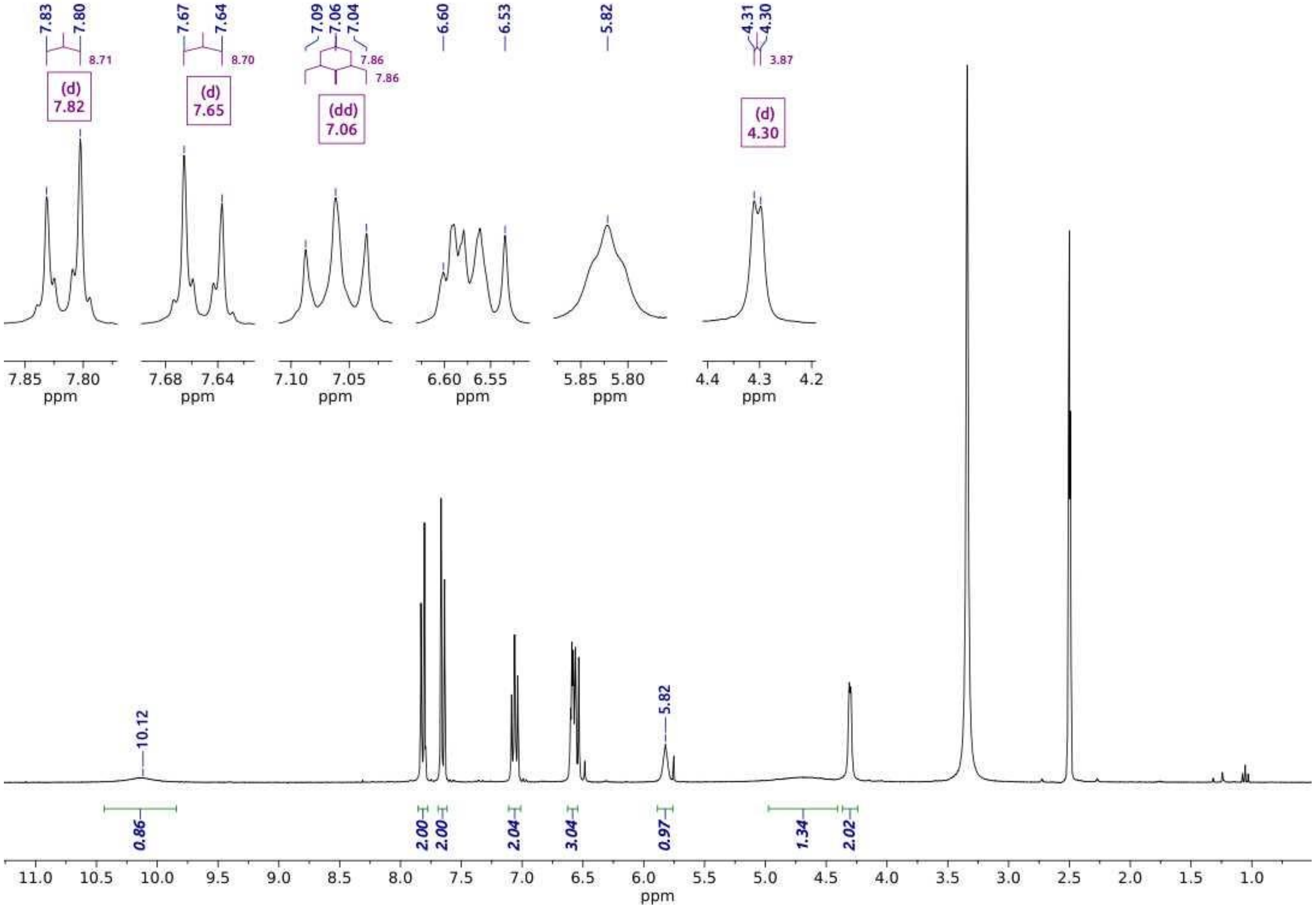

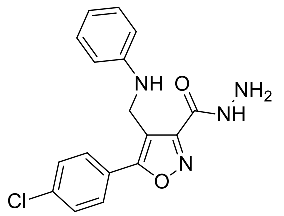

Figure S43 – ^{1}H NMR spectrum of compound **3da** in DMSO-d_6 at 300.06 MHz.

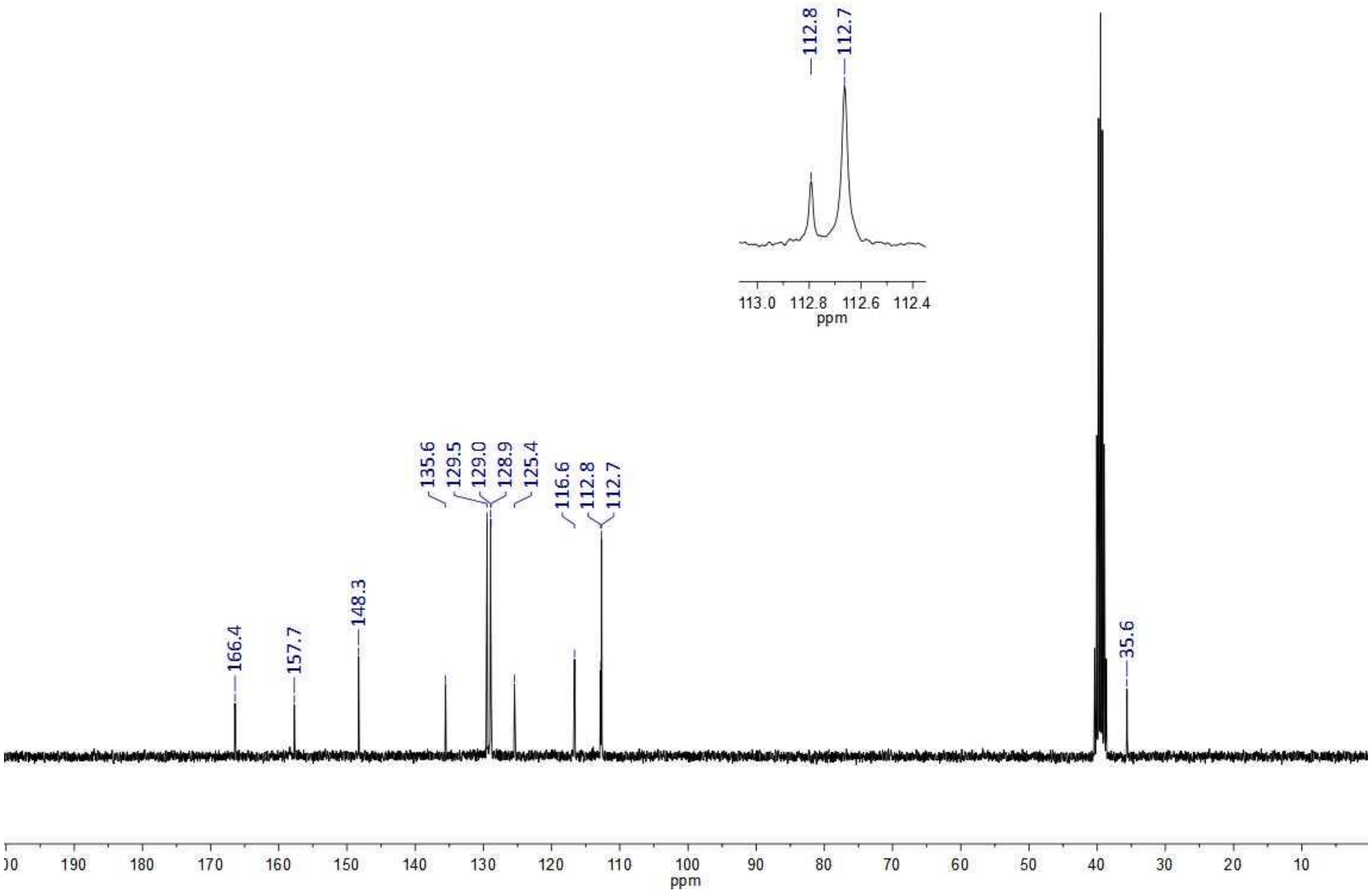

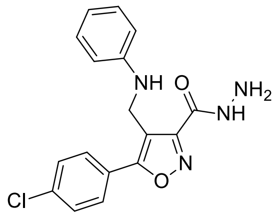

Figure S44 – ^{13}C NMR spectrum of compound **3da** in DMSO-d_6 at 75.45 MHz.

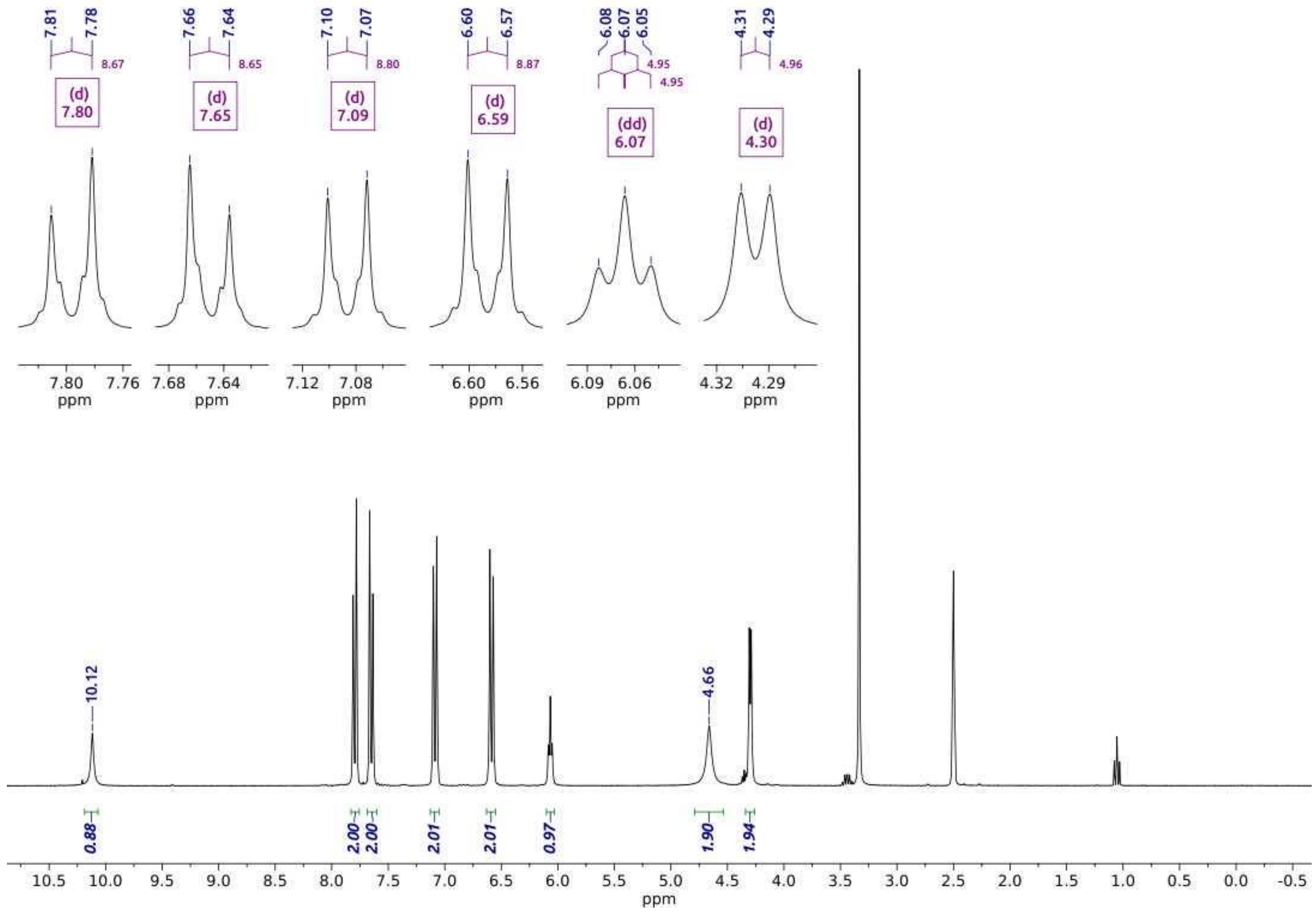

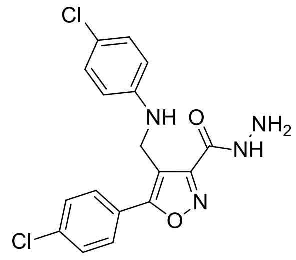

Figure S45 – ^{1}H NMR spectrum of compound **3db** in DMSO-d_6 at 300.06 MHz.

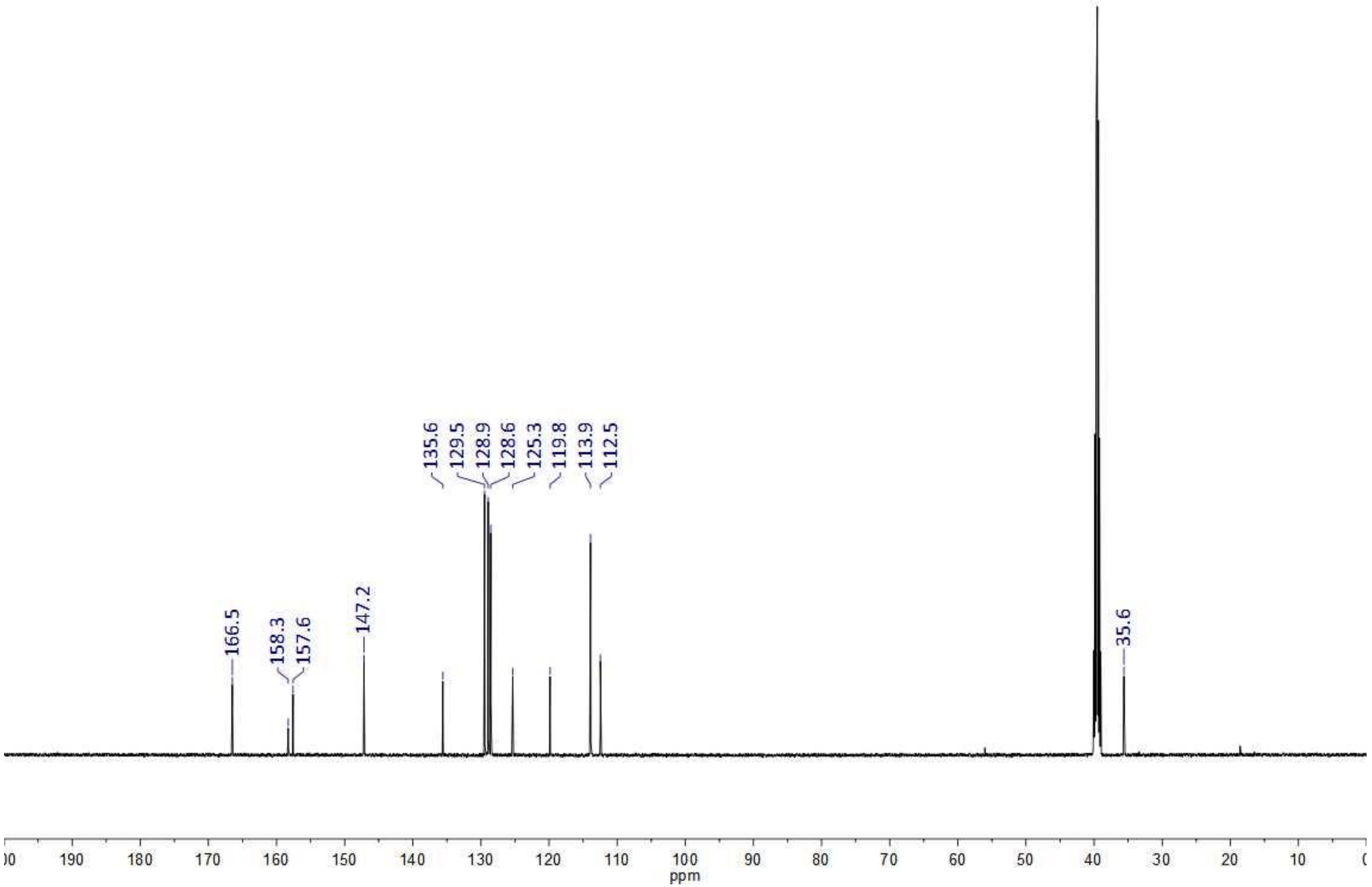

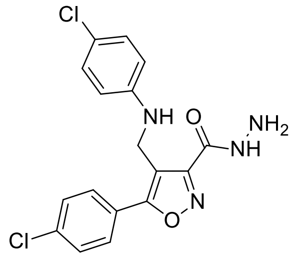

Figure S46 – ^{13}C NMR spectrum of compound **3db** in DMSO-d_6 at 75.45 MHz.

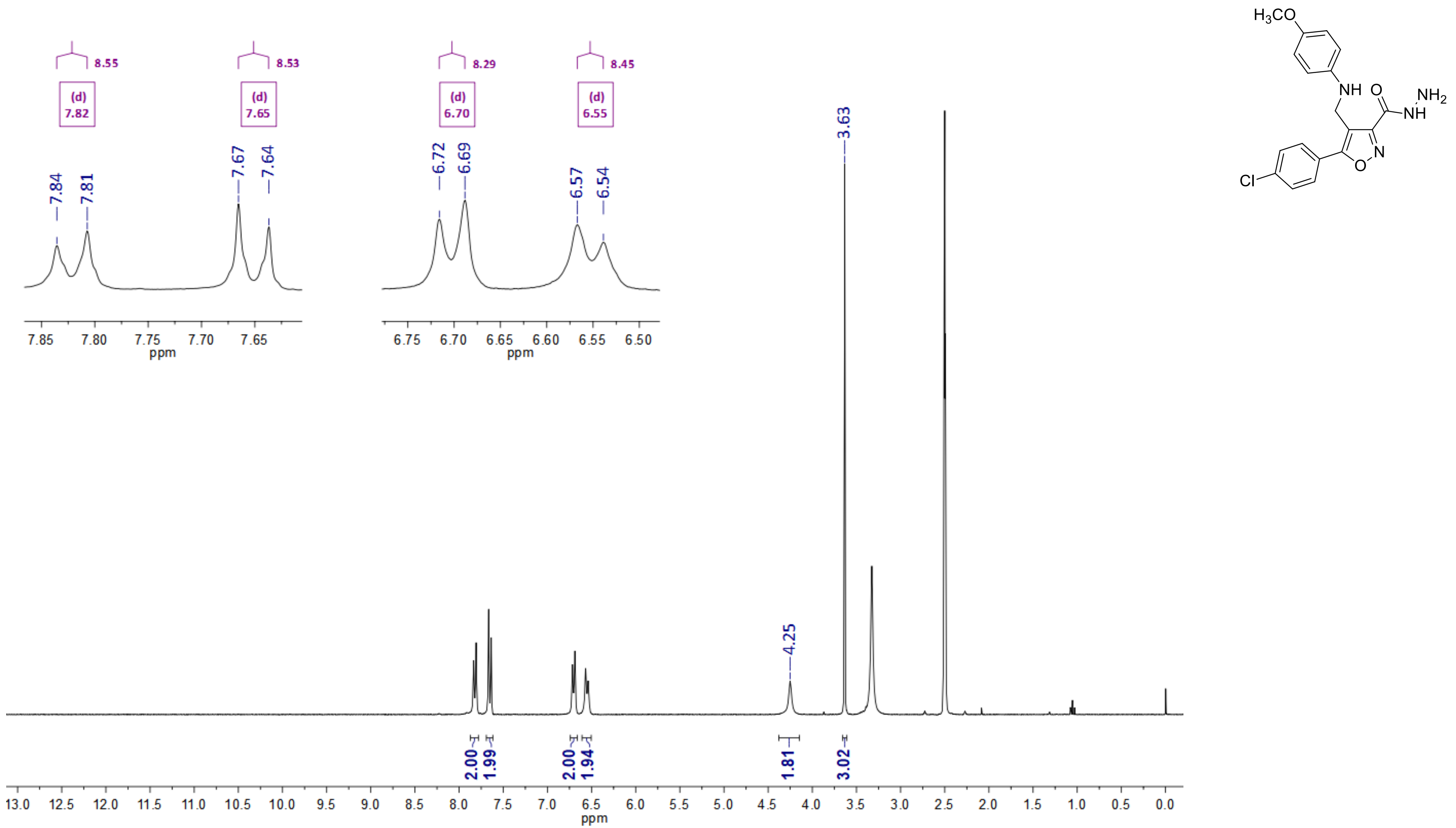

Figure S47 – ^{1}H NMR spectrum of compound **3dc** in DMSO-d_6 at 300.06 MHz.

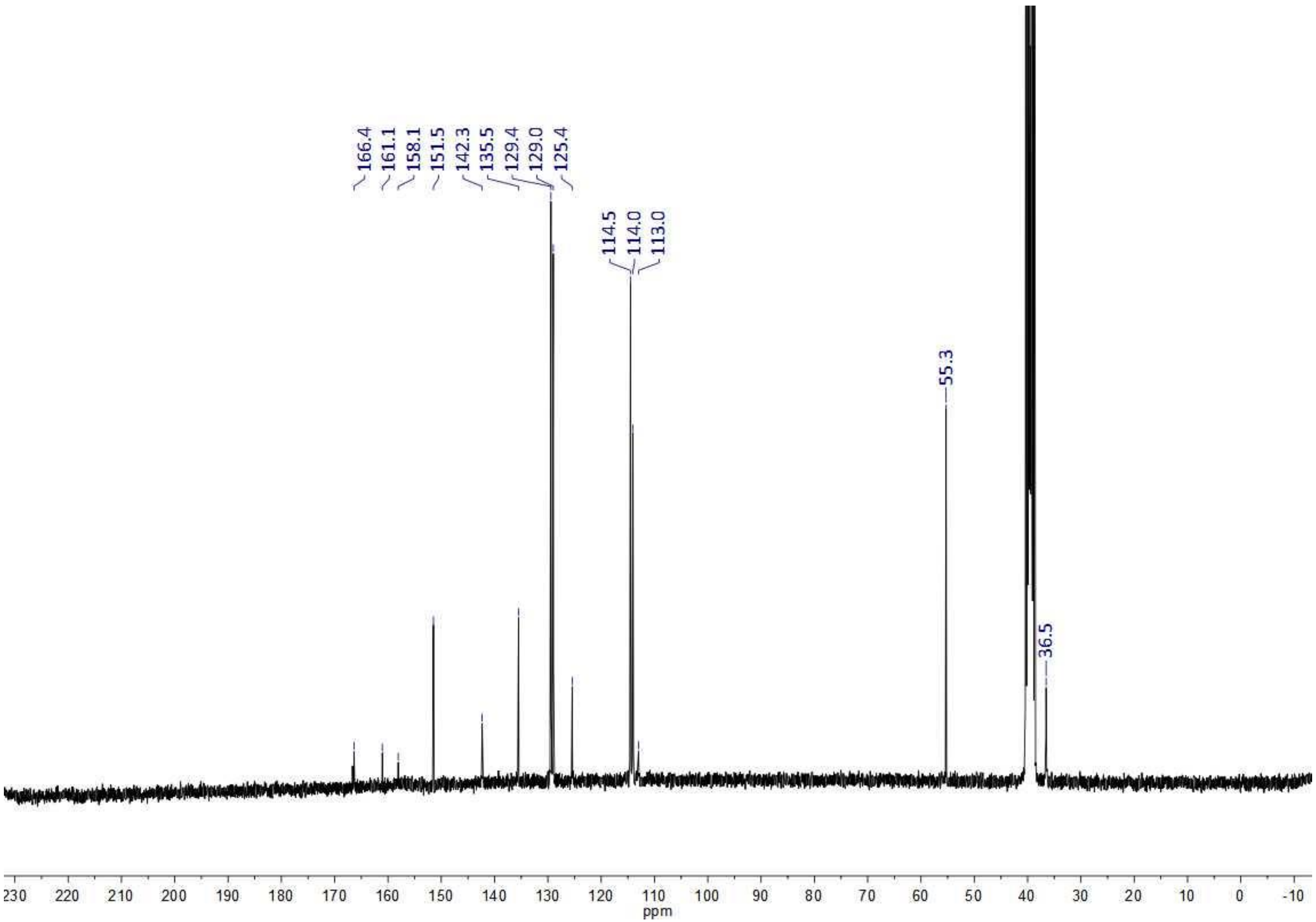

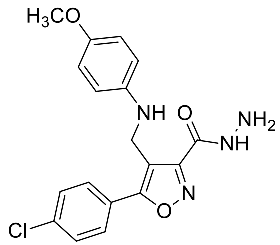

Figure S48 – ^{13}C NMR spectrum of compound **3dc** in DMSO-d_6 at 75.45 MHz.

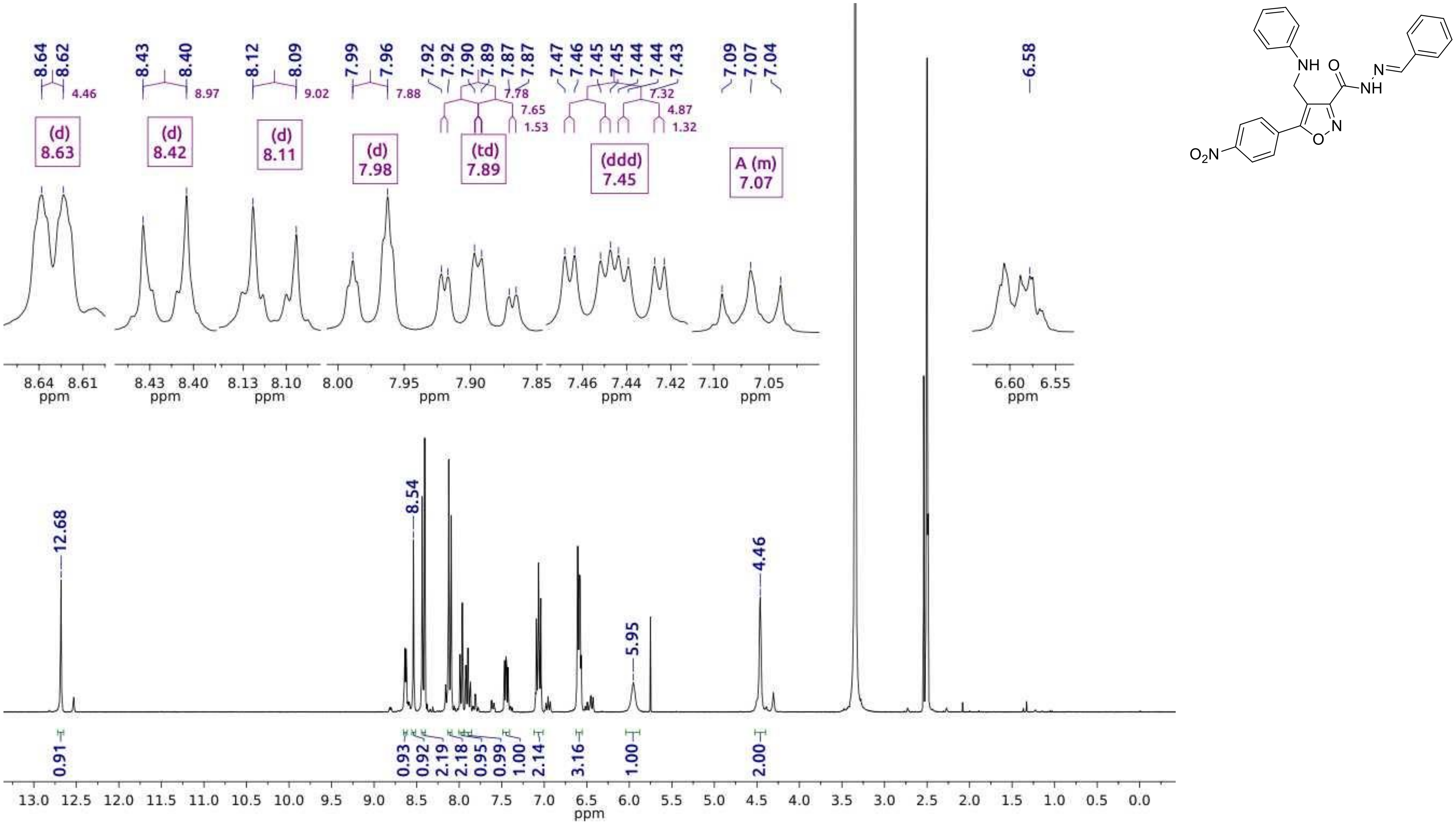

Figure S49 – ^{1}H NMR spectrum of compound **4aa** in DMSO-d_6 at 300.06 MHz.

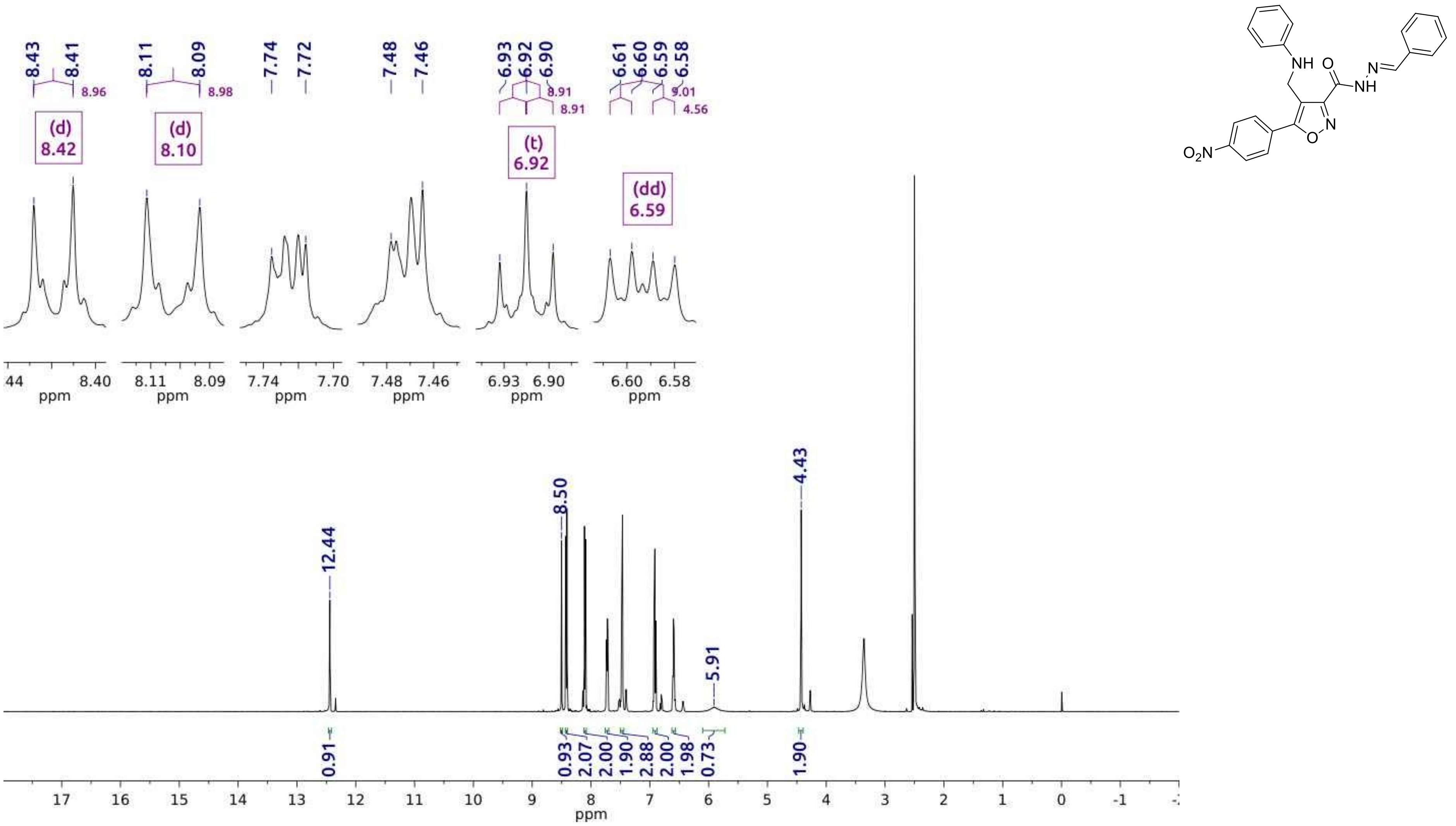

Figure S50 – ^{13}C NMR spectrum of compound **4aa** in DMSO-d_6 at 75.45 MHz.

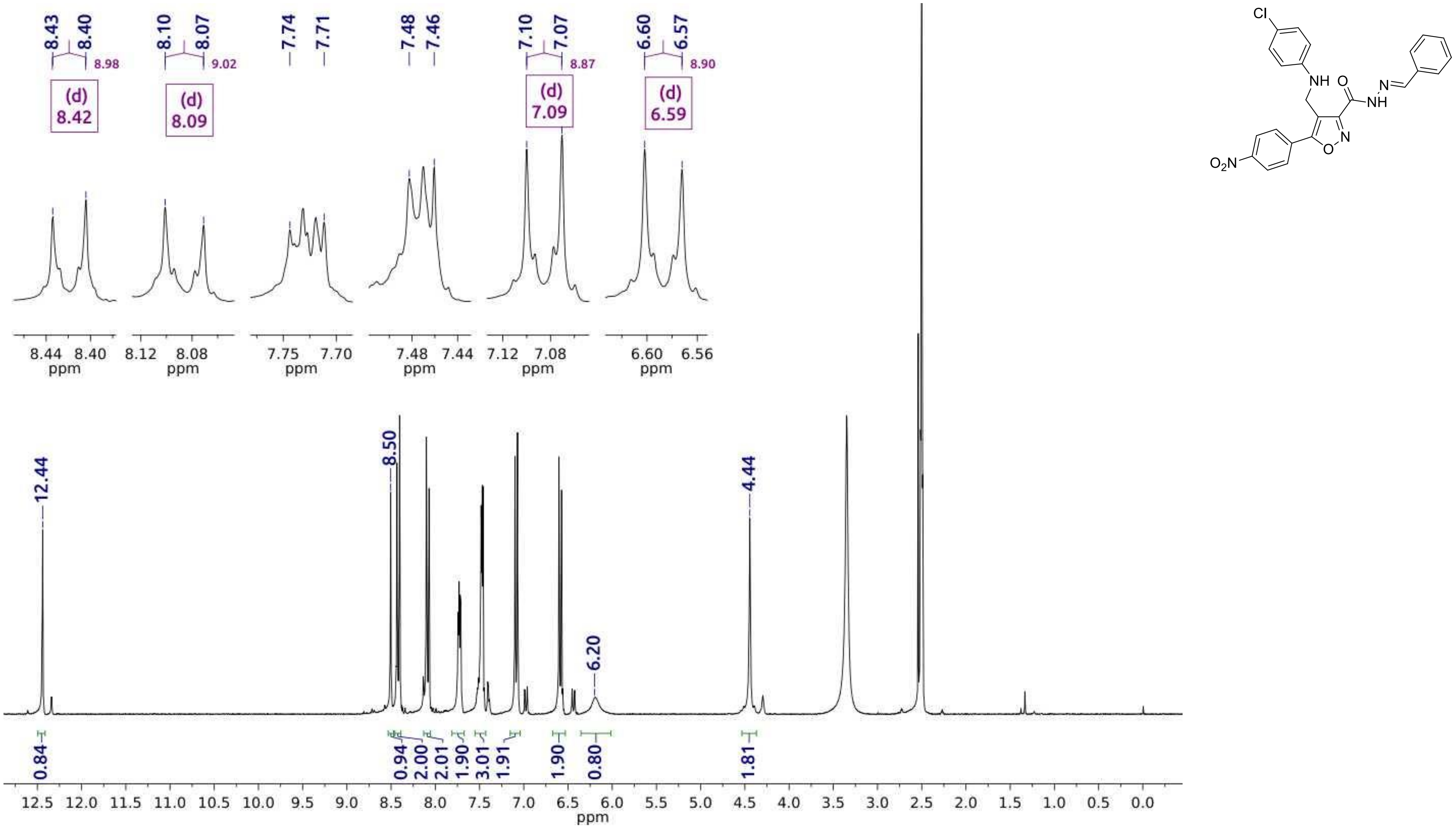

Figure S51 – ^{1}H NMR spectrum of compound **4ab** in DMSO-d_6 at 300.06 MHz.

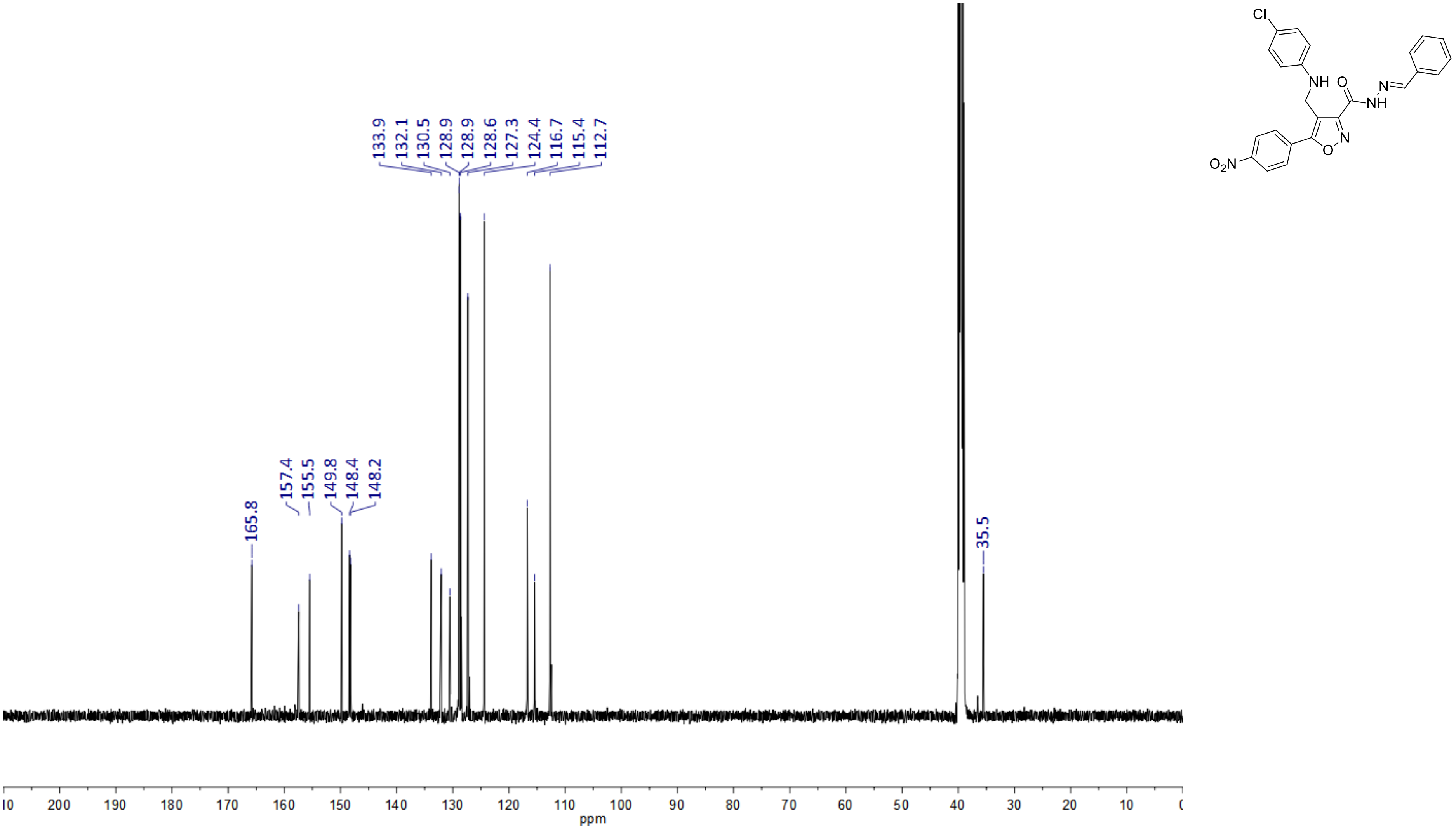

Figure S52 – ^{13}C NMR spectrum of compound **4ab** in DMSO-d_6 at 75.45 MHz.

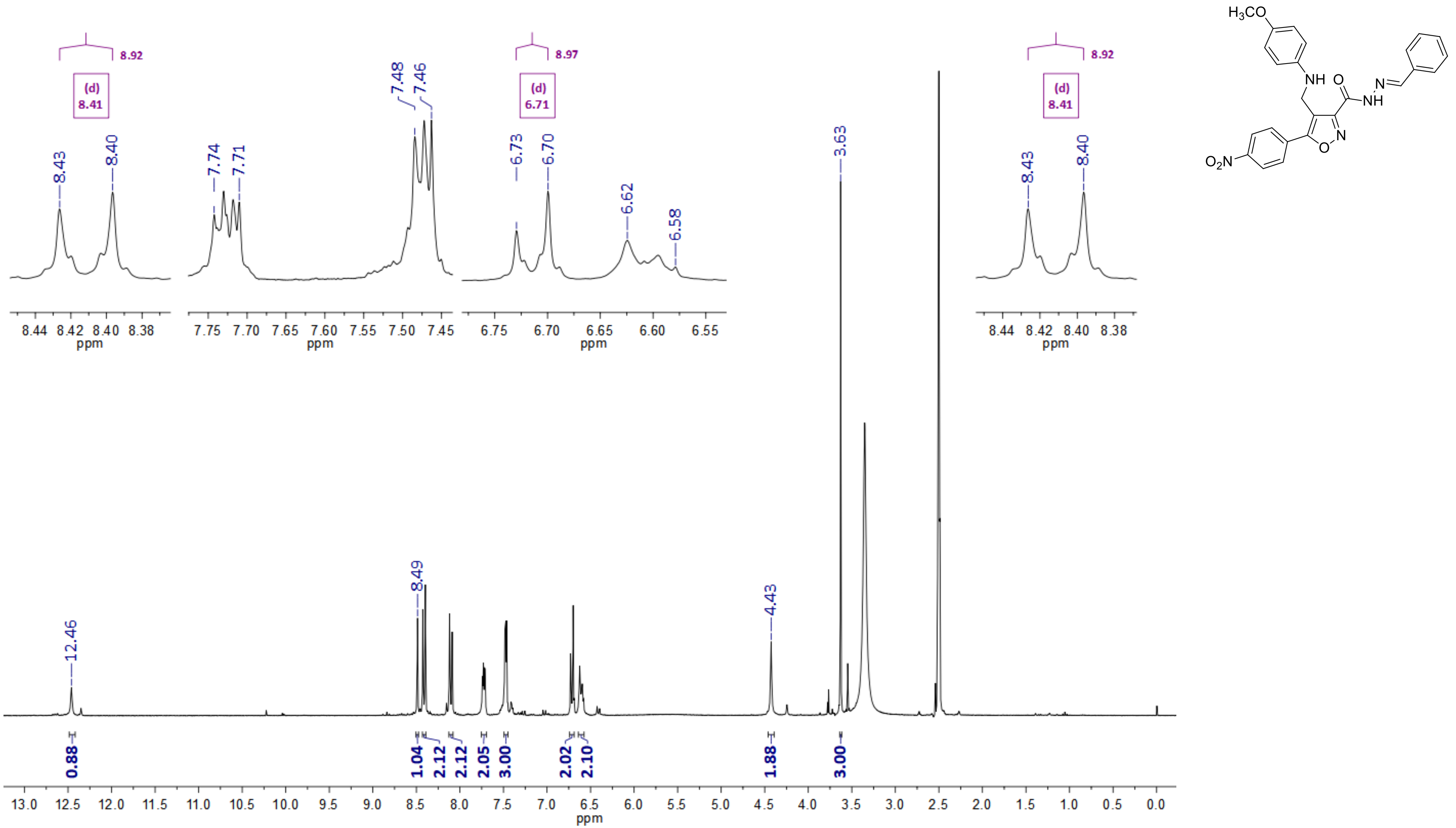

Figure S53 – ^{1}H NMR spectrum of compound **4ac** in DMSO-d_6 at 300.06 MHz.

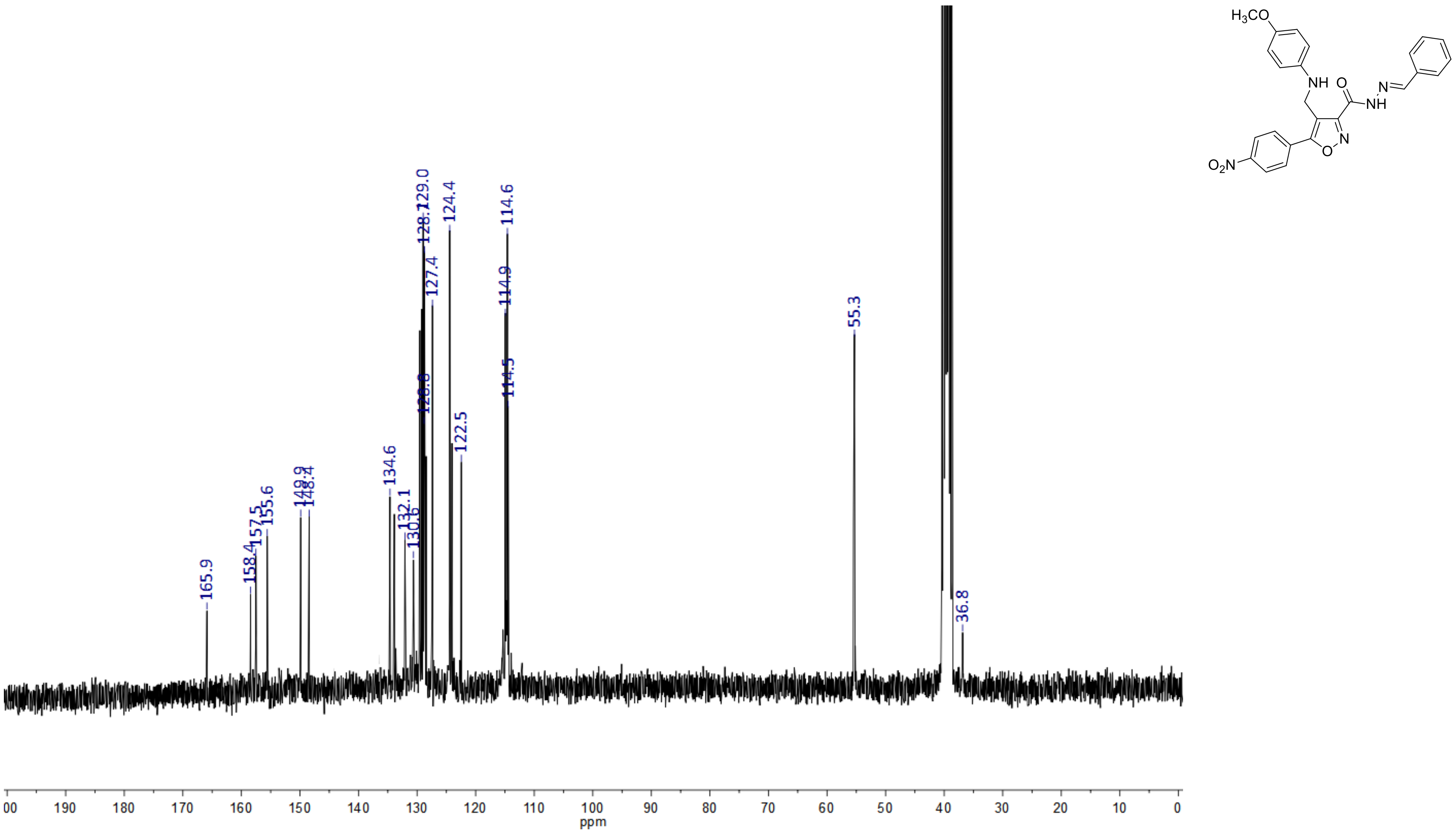

Figure S54 – ^{13}C NMR spectrum of compound **4ac** in DMSO-d_6 at 75.45 MHz.

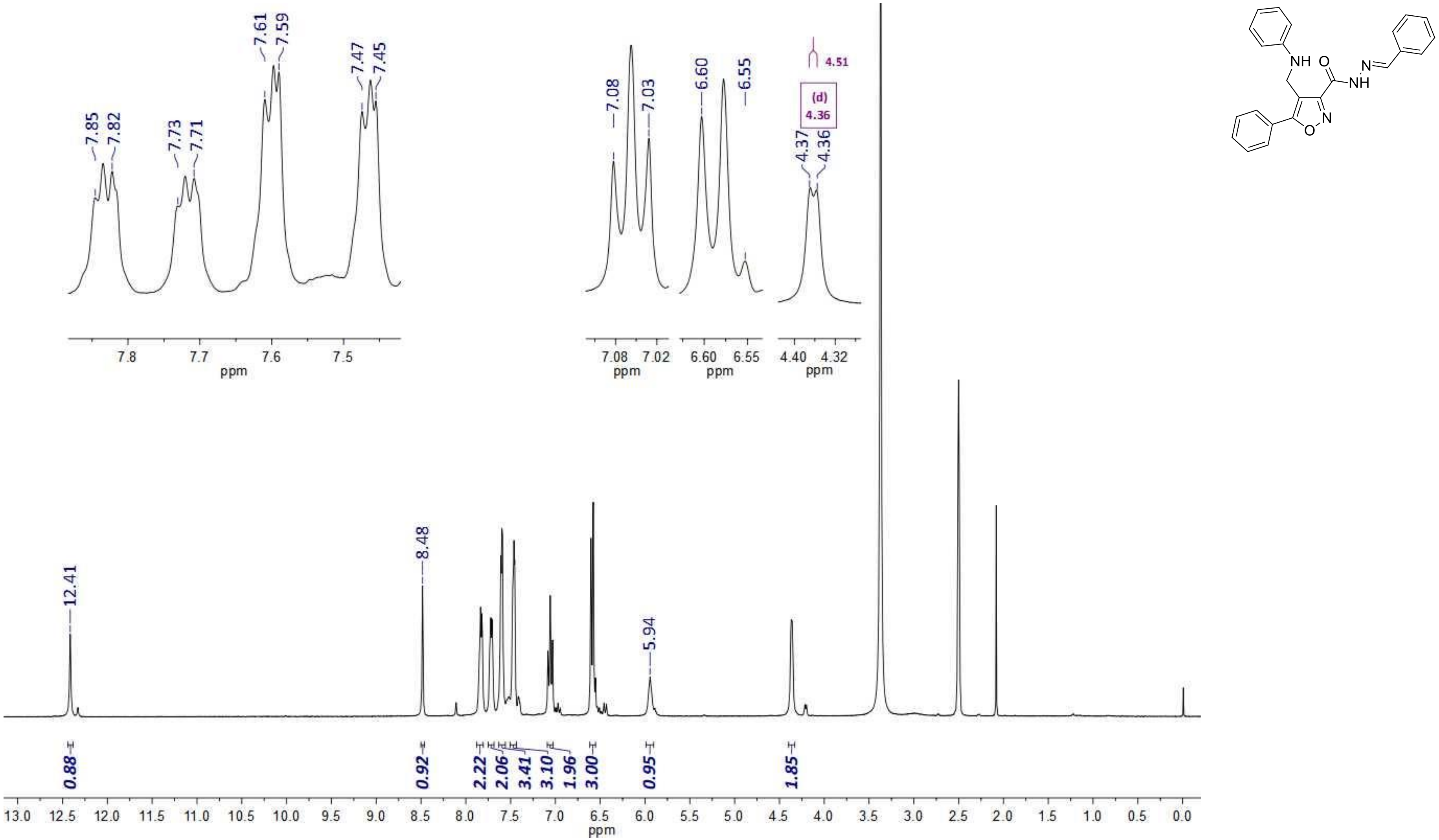

Figure S55 – ^{1}H NMR spectrum of compound **4ba** in DMSO-d_6 at 300.06 MHz.

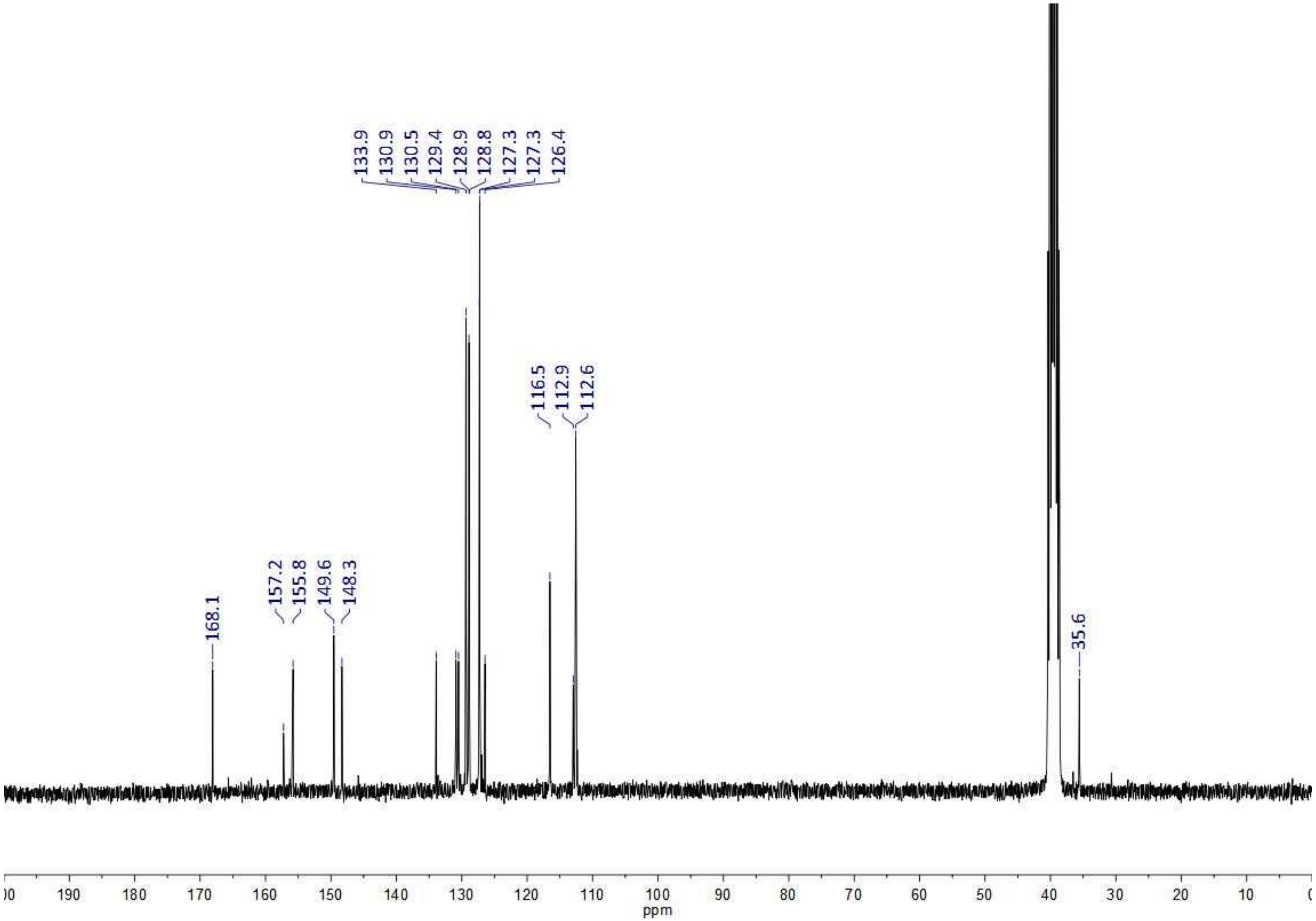

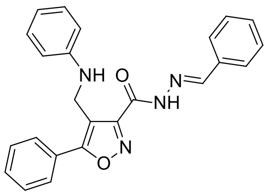

Figure S56 – ^{13}C NMR spectrum of compound **4ba** in DMSO-d_6 at 75.45 MHz.

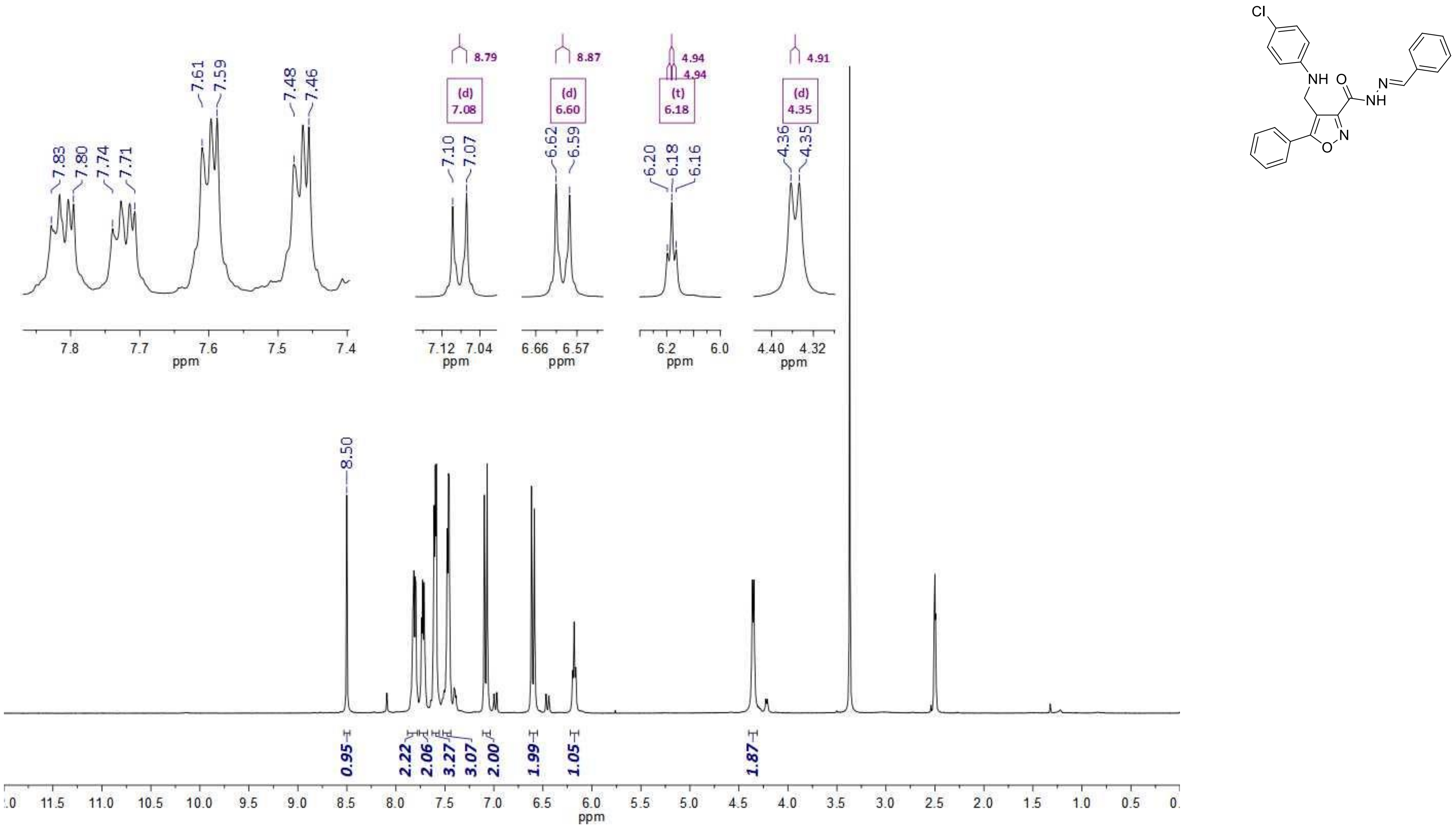

Figure S57 – ^{1}H NMR spectrum of compound **4bb** in DMSO-d_6 at 300.06 MHz.

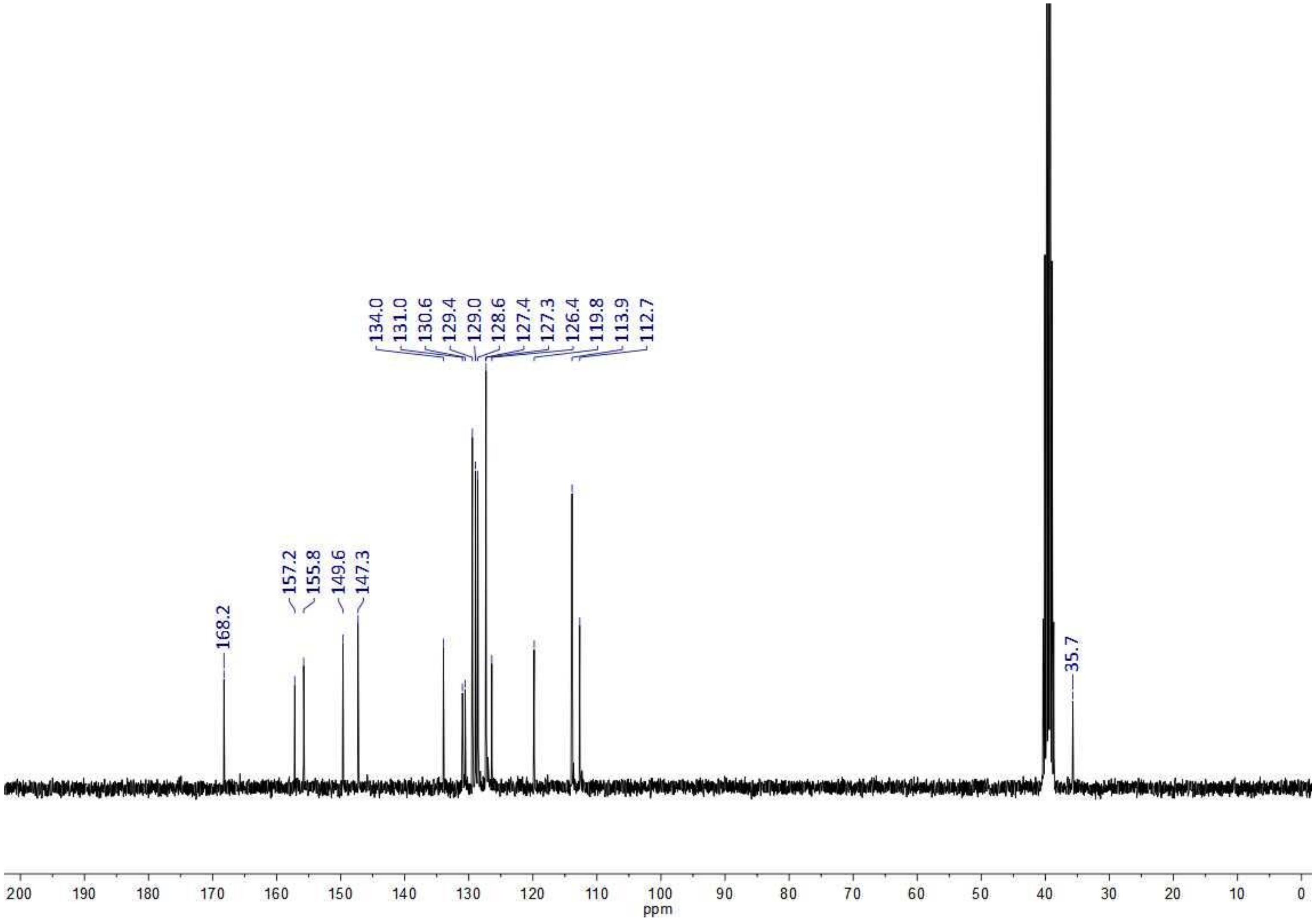

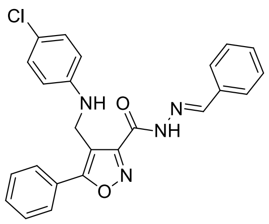

Figure S58 – ^{13}C NMR spectrum of compound **4bb** in DMSO-d_6 at 75.45 MHz.

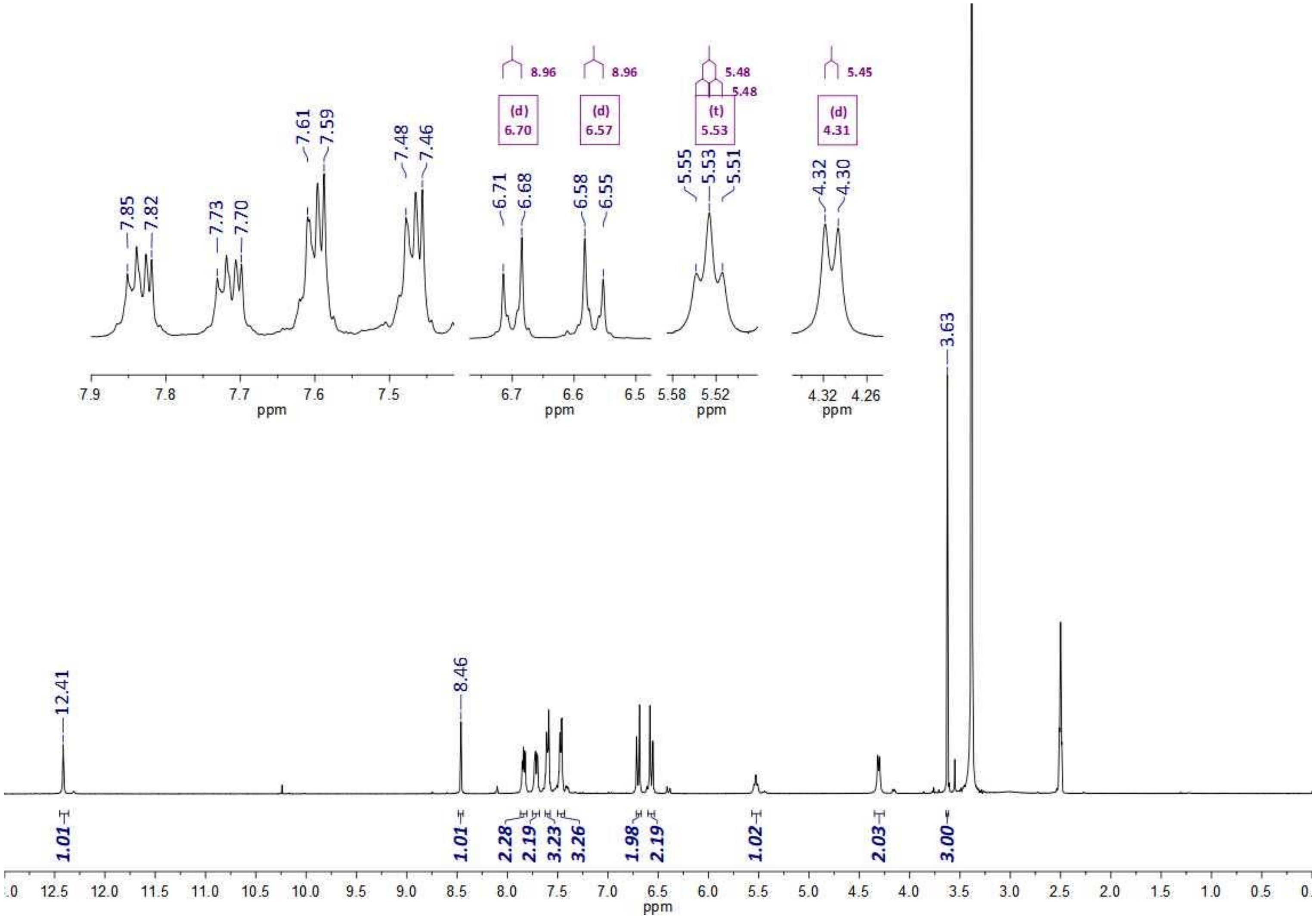

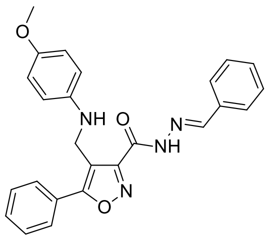

Figure S59 – ^{1}H NMR spectrum of compound **4bc** in DMSO-d_6 at 300.06 MHz.

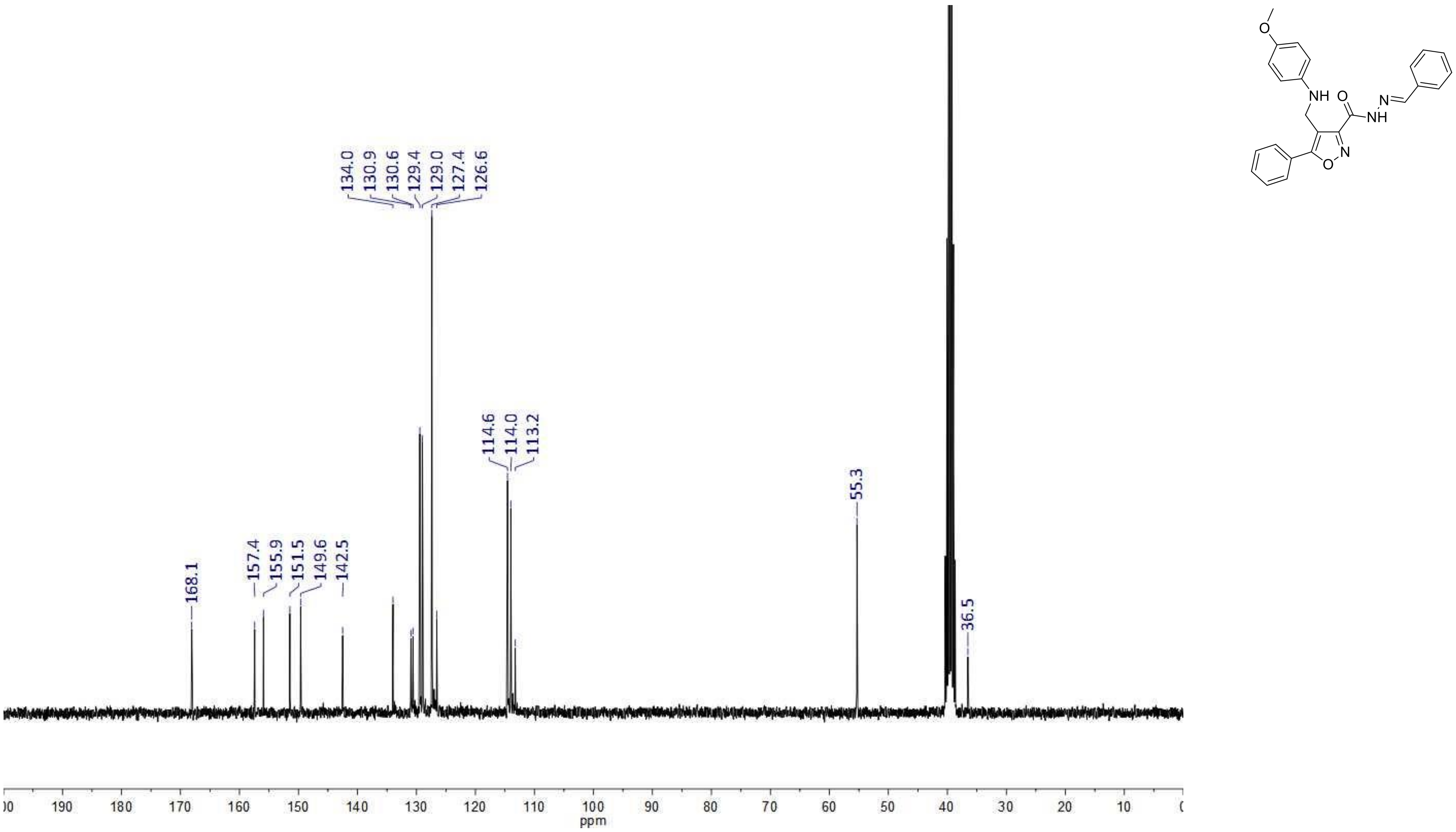

Figure S60 – ^{13}C NMR spectrum of compound **4bc** in DMSO-d_6 at 75.45 MHz.

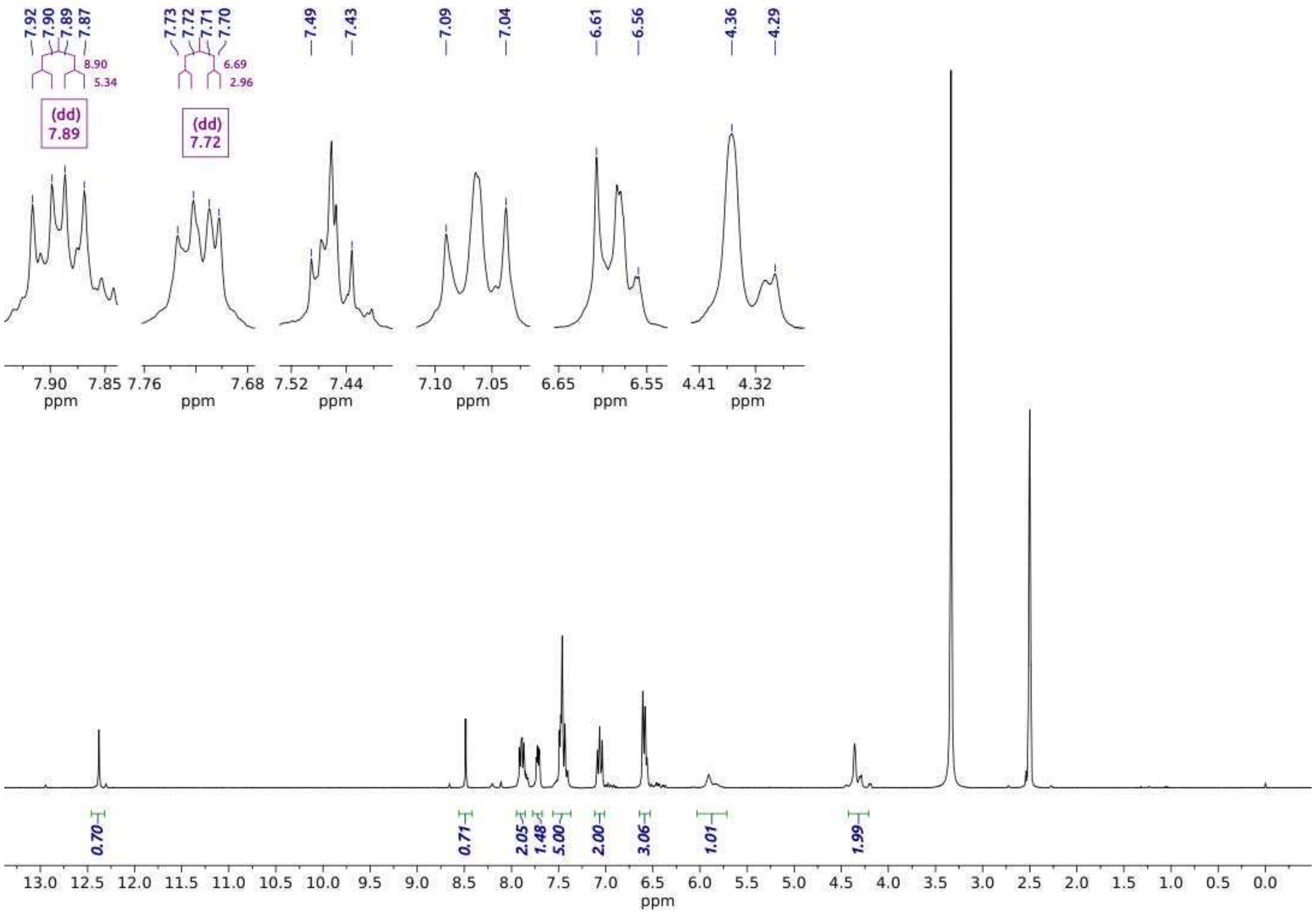

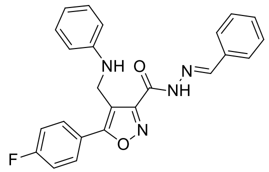

Figure S61 – ^{1}H NMR spectrum of compound **4ca** in DMSO-d_6 at 300.06 MHz.

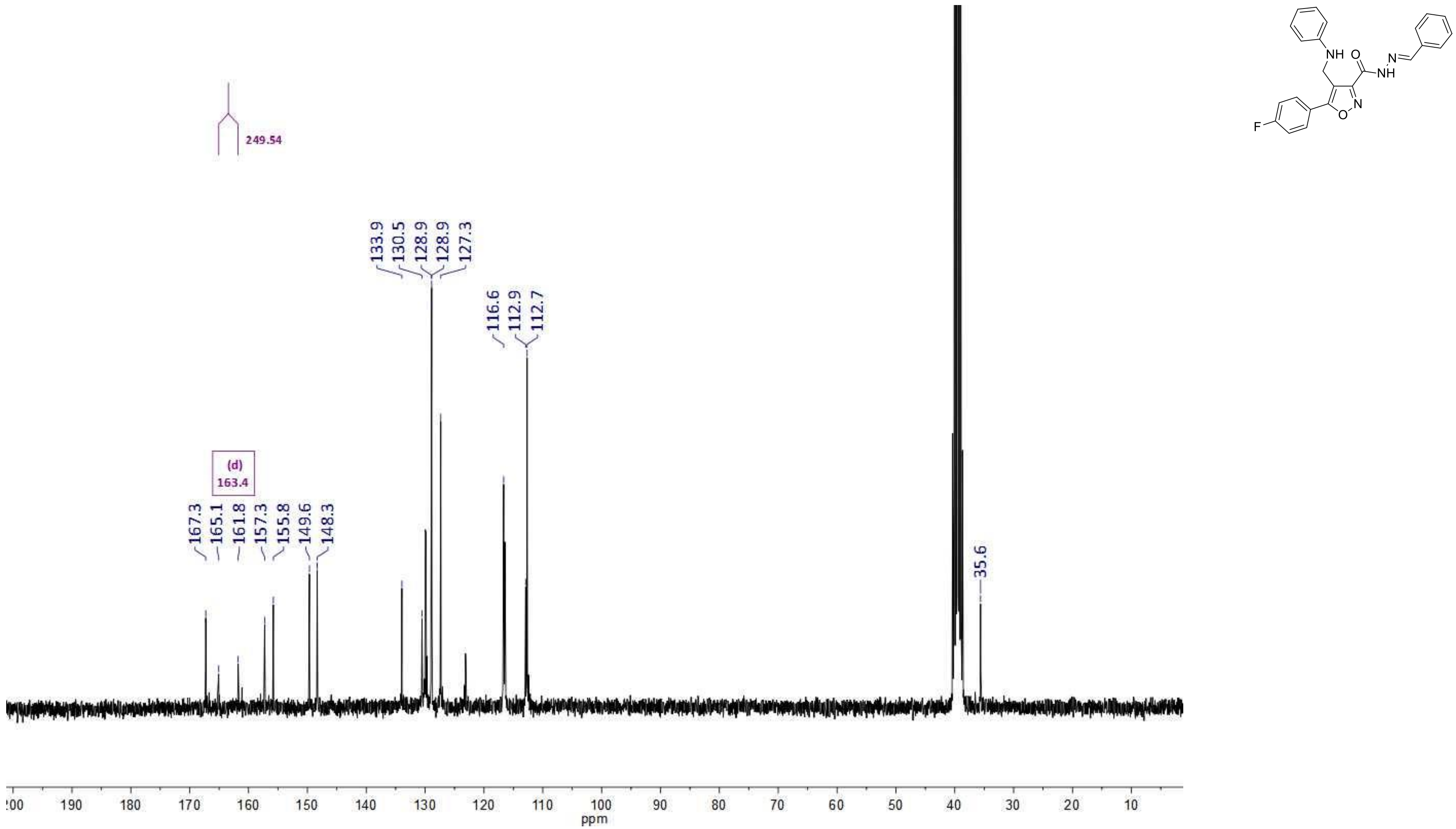

Figure S62 – ^{13}C NMR spectrum of compound **4ca** in DMSO-d_6 at 75.45 MHz.

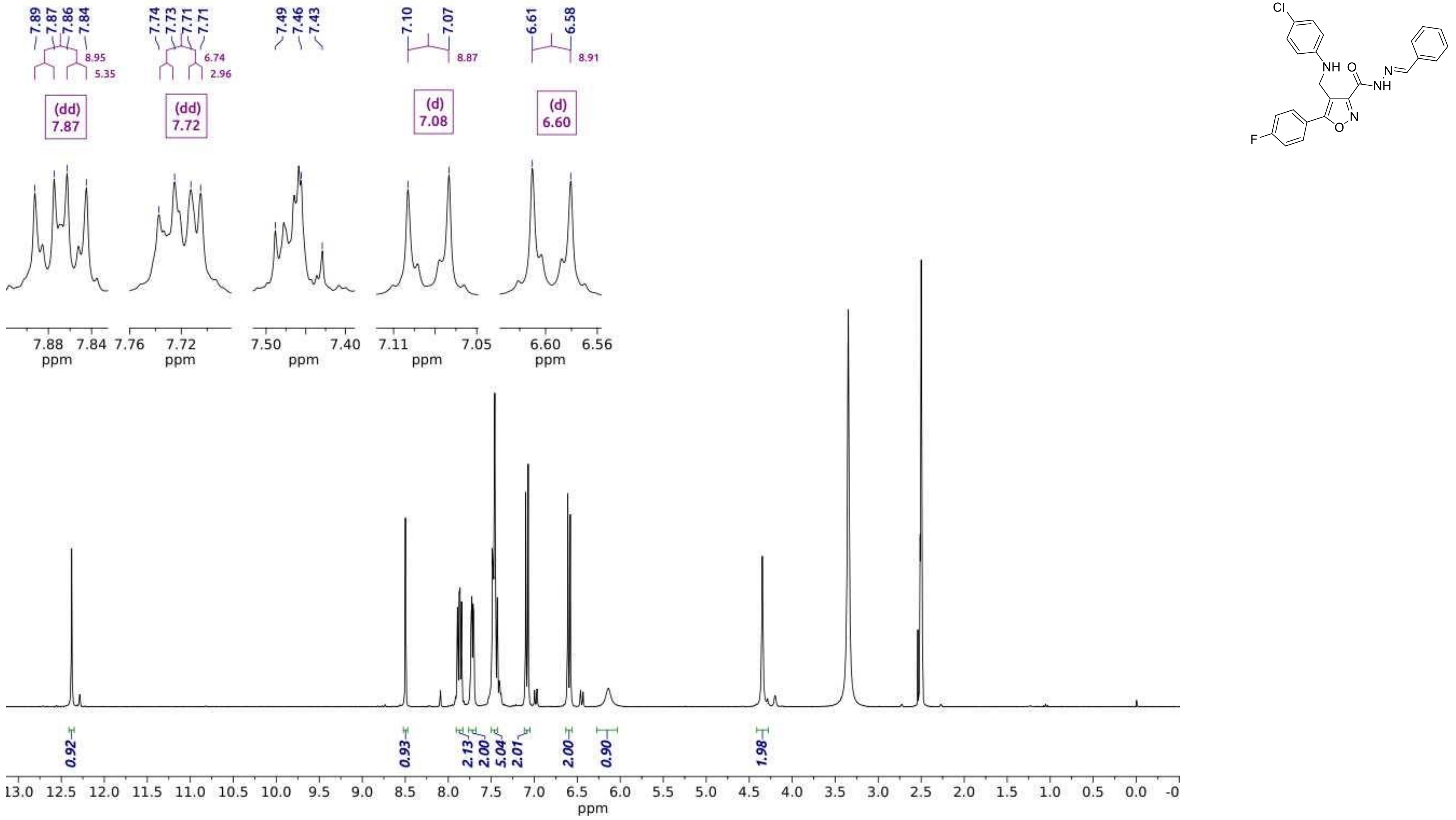

Figure S63 – ^{1}H NMR spectrum of compound **4cb** in DMSO-d_6 at 300.06 MHz.

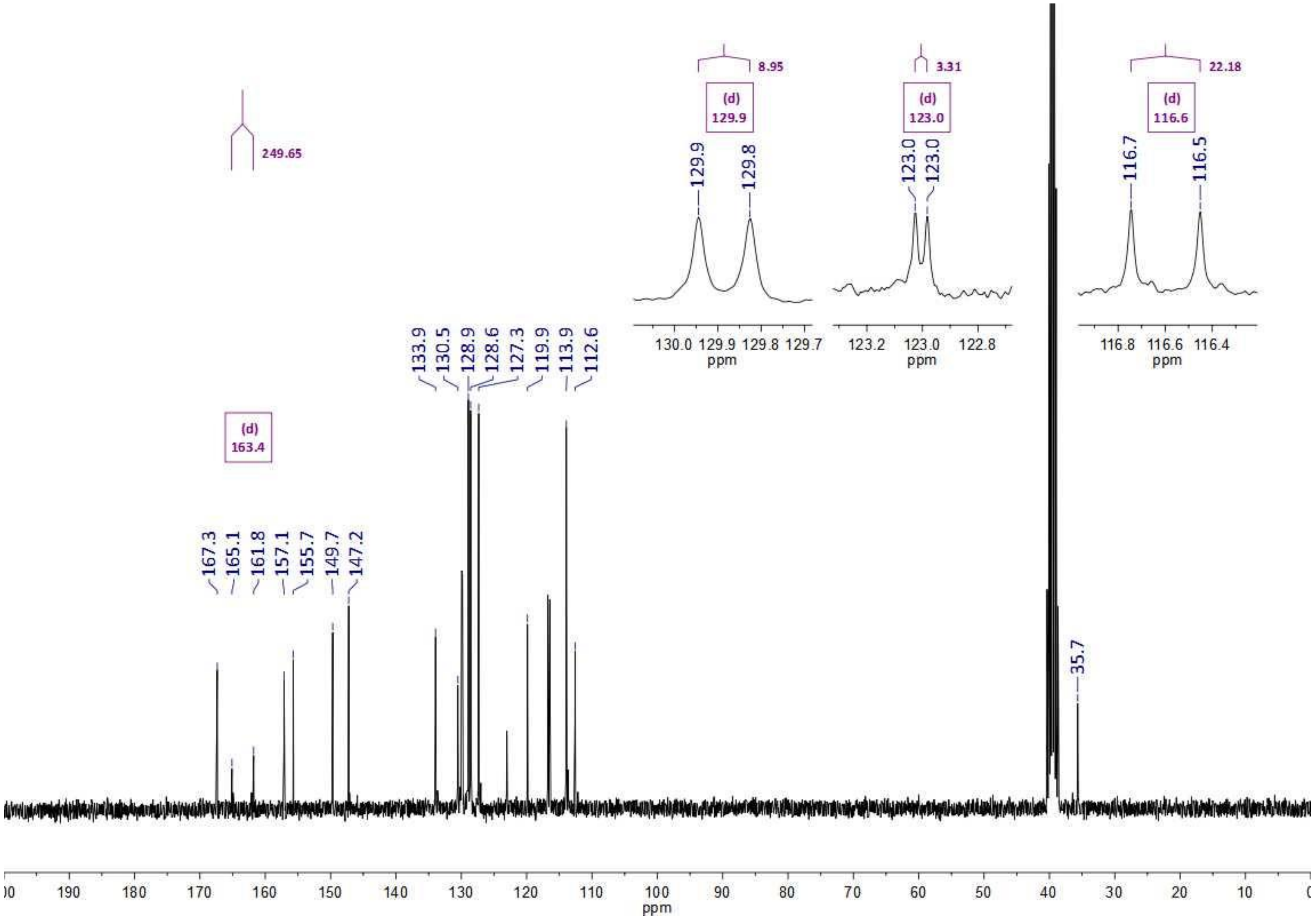

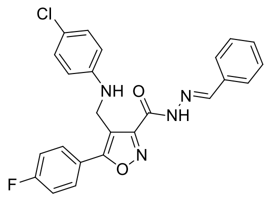

Figure S64 – ^{13}C NMR spectrum of compound **4cb** in DMSO-d_6 at 75.45 MHz.

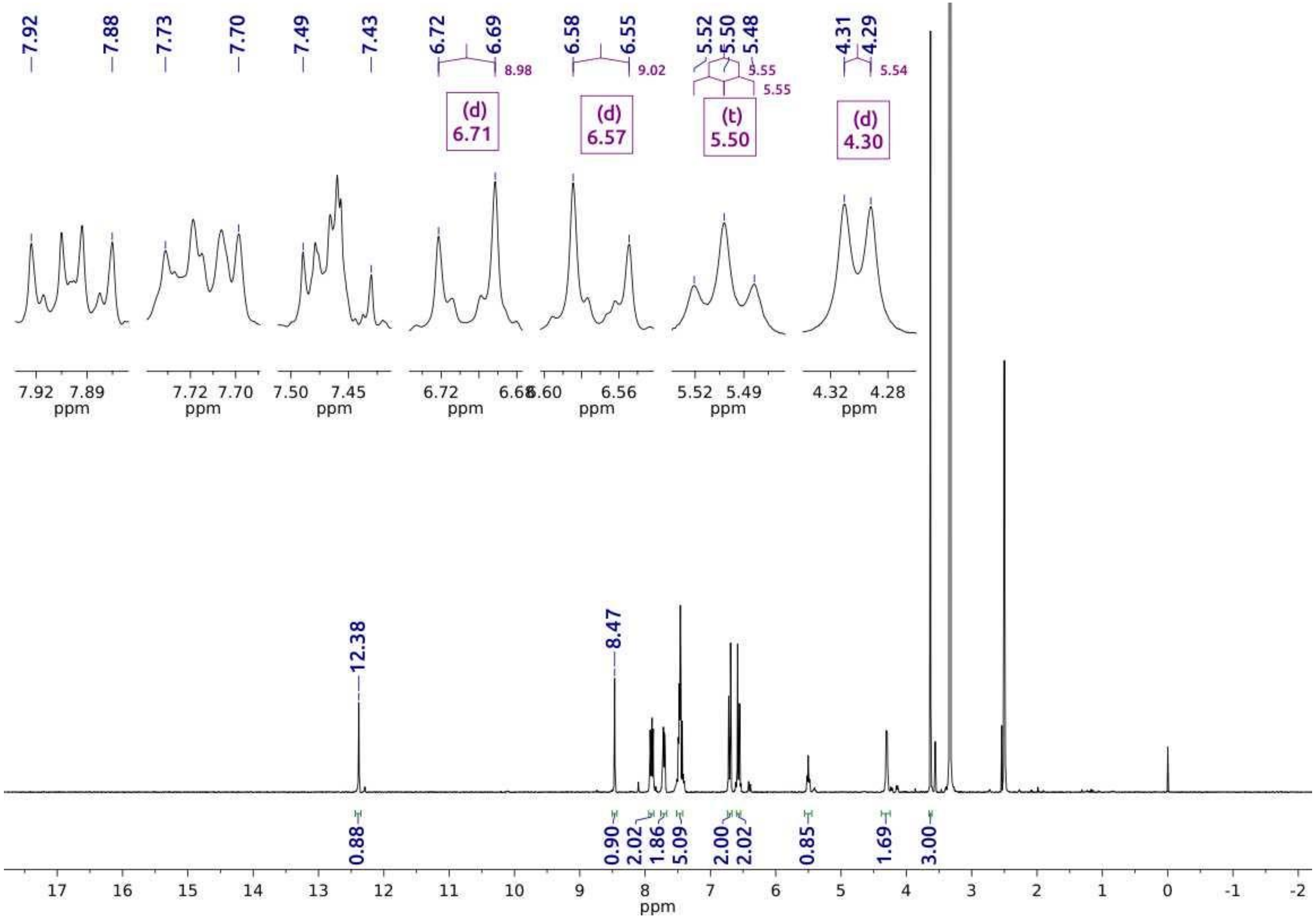

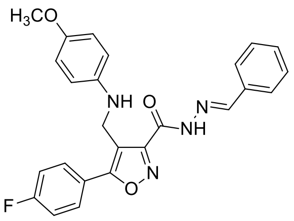

Figure S65 – ^{1}H NMR spectrum of compound **4cc** in DMSO-d_6 at 300.06 MHz.

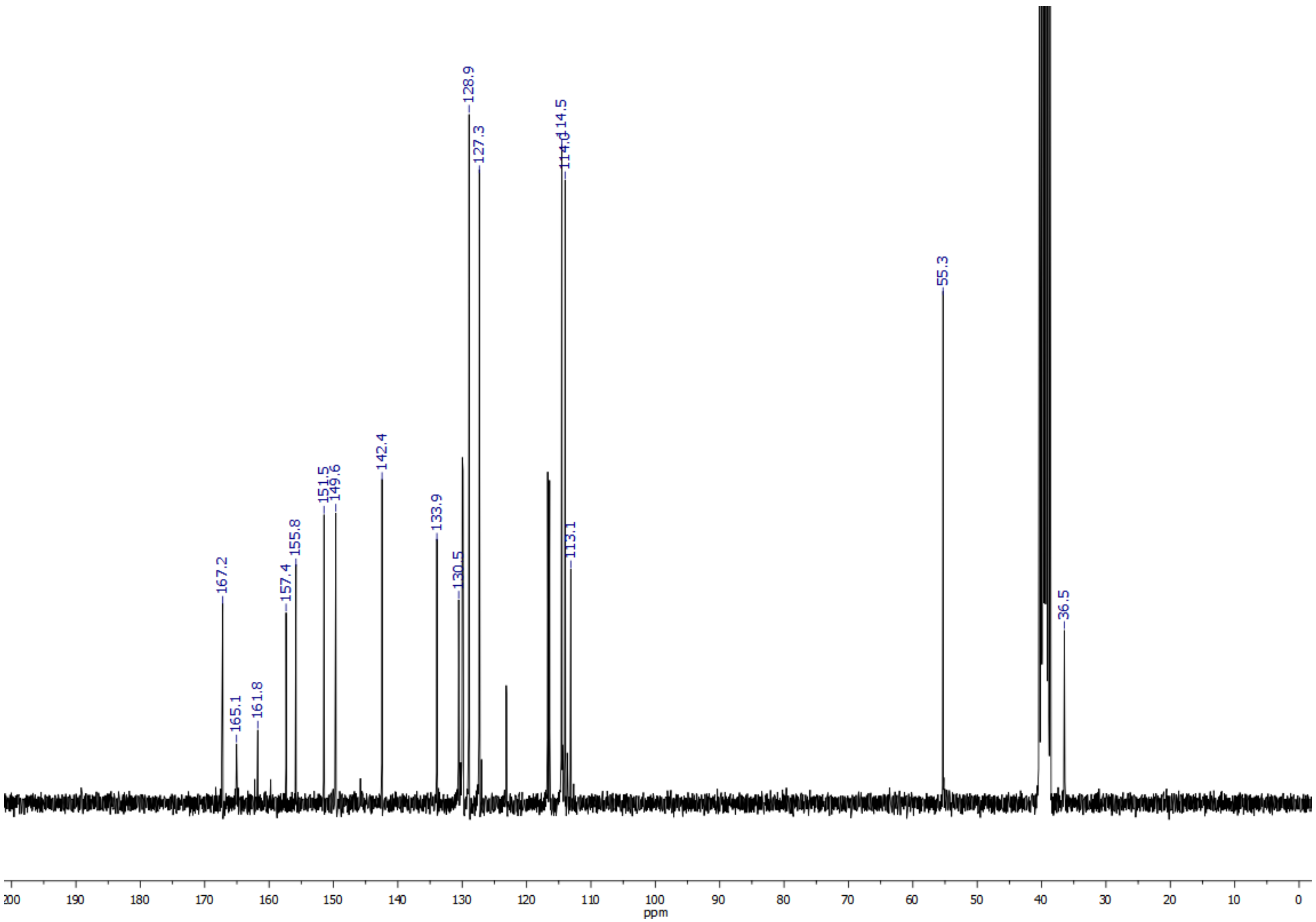

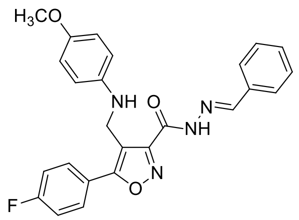

Figure S66 – ^{13}C NMR spectrum of compound **4cc** in DMSO-d_6 at 75.45 MHz.

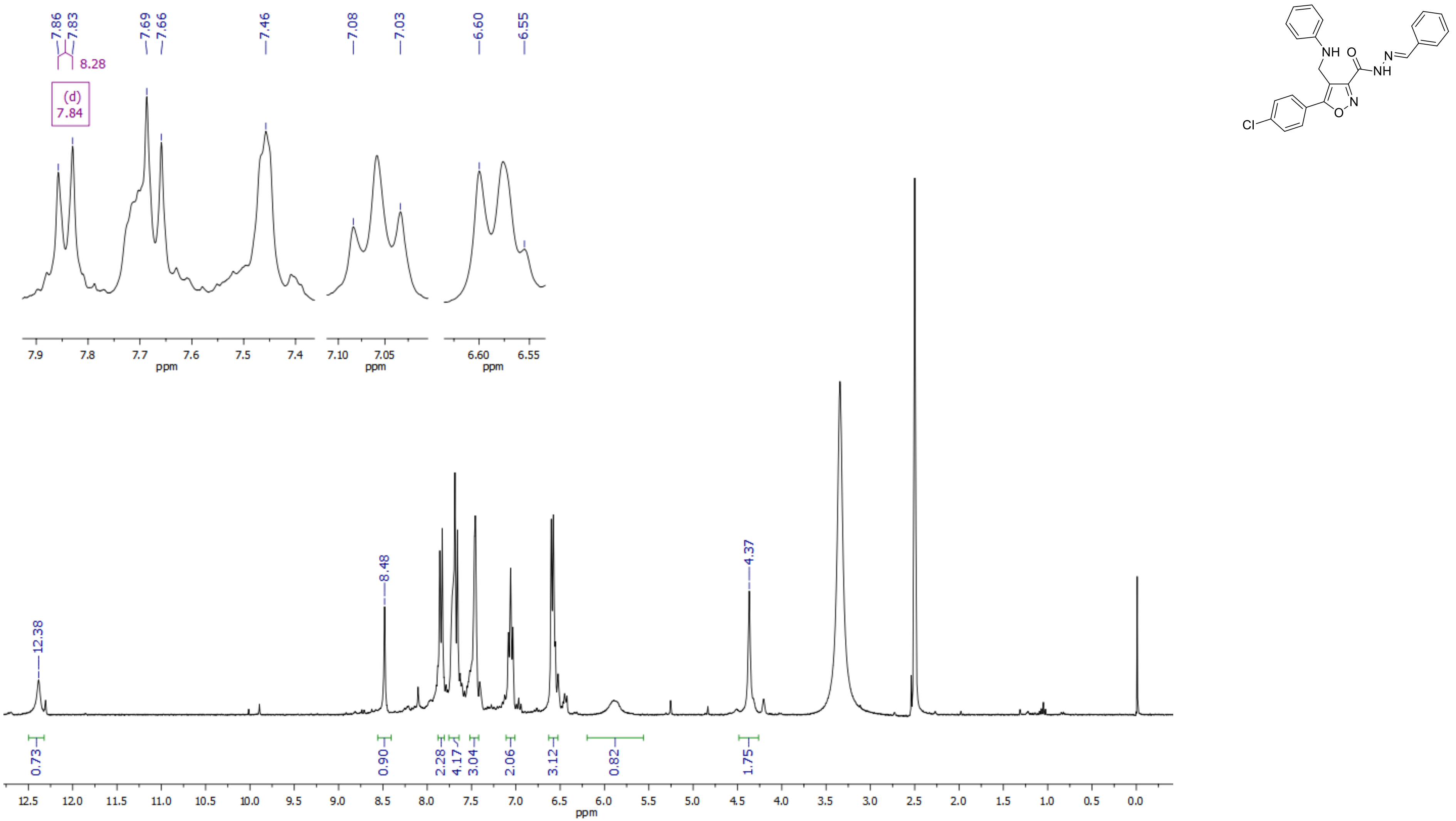

Figure S67 – ^{1}H NMR spectrum of compound **4da** in DMSO-d_6 at 300.06 MHz.

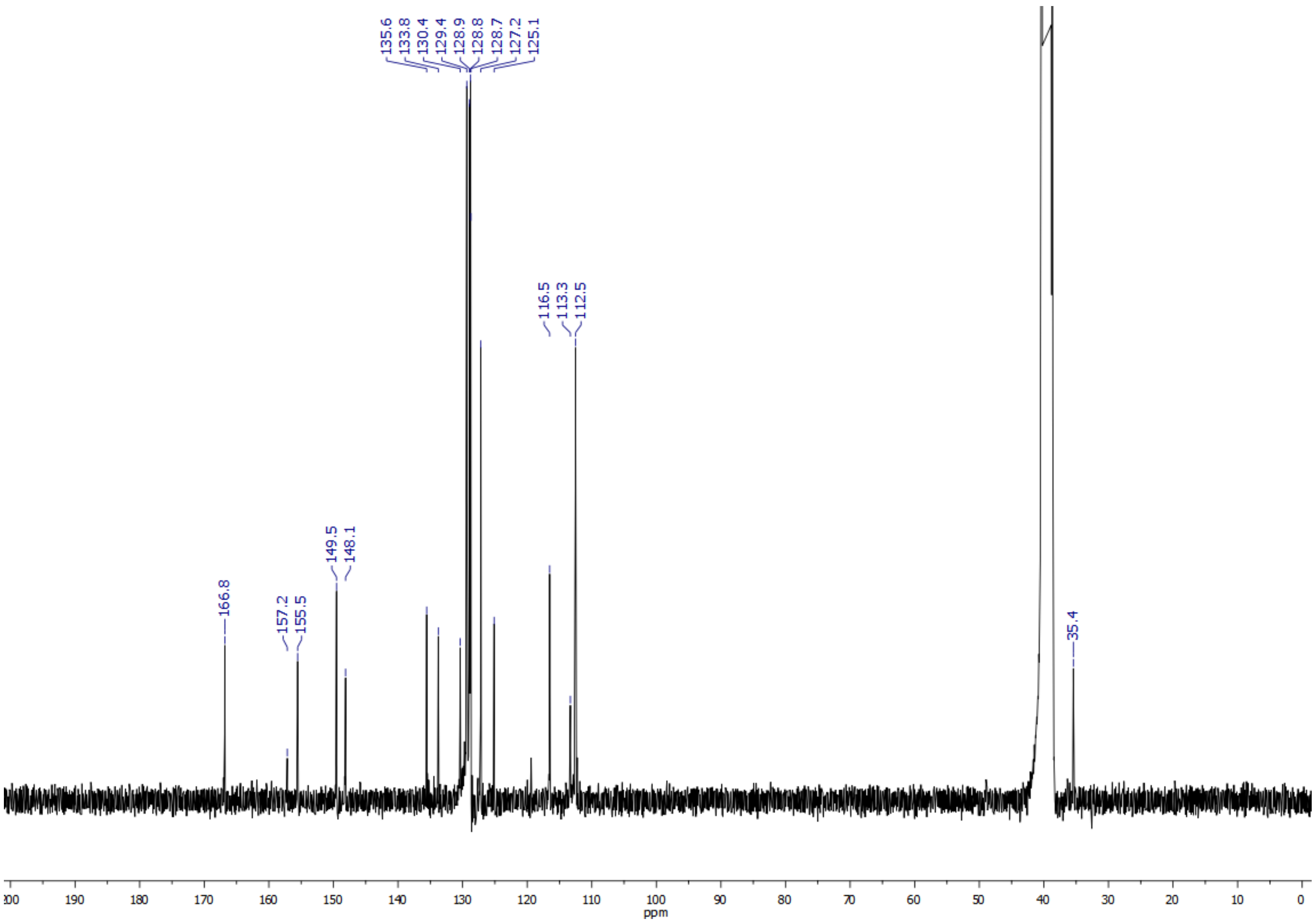

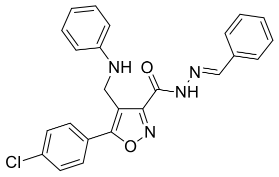

Figure S68 – ^{13}C NMR spectrum of compound **4da** in DMSO-d_6 at 75.45 MHz.

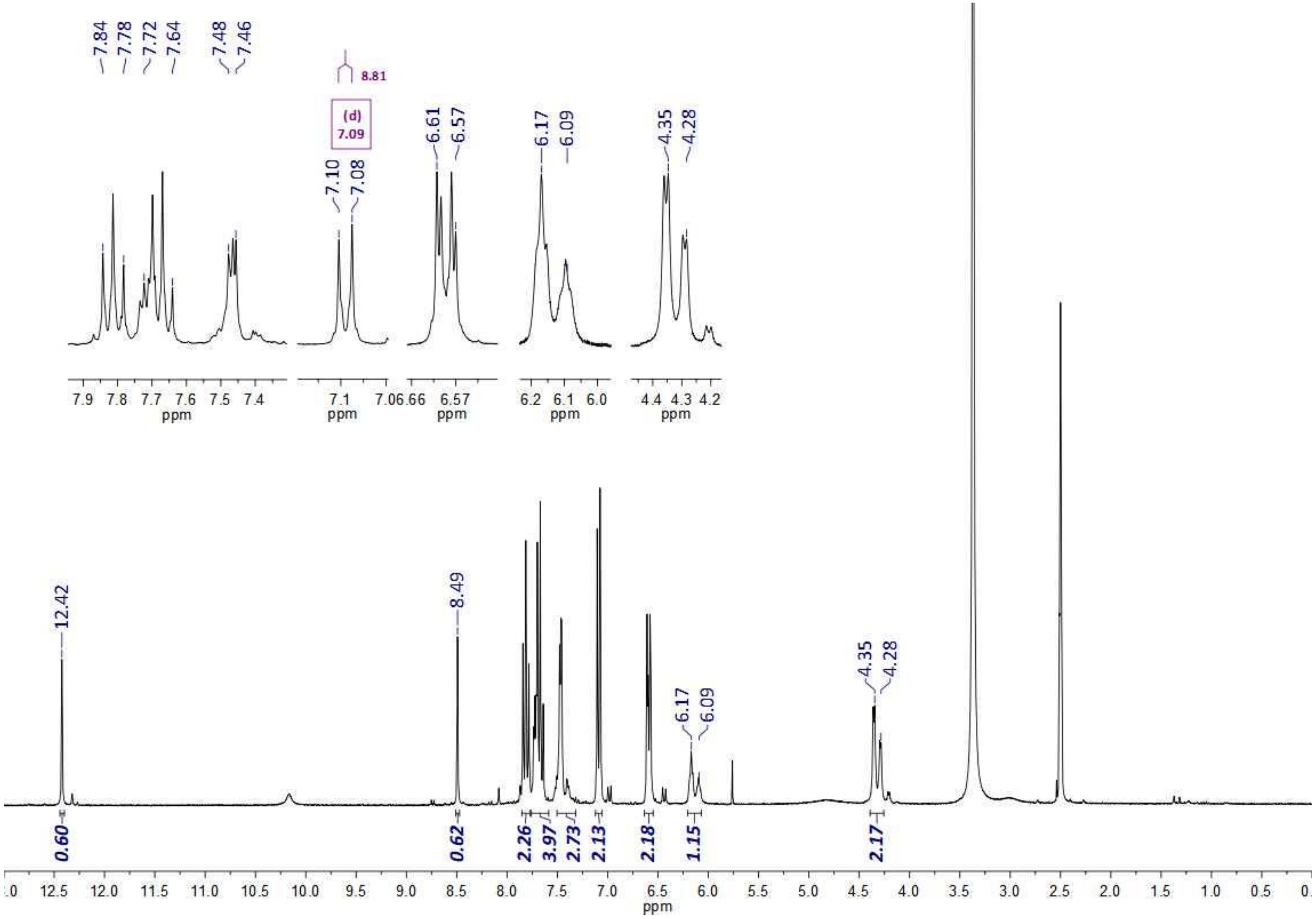

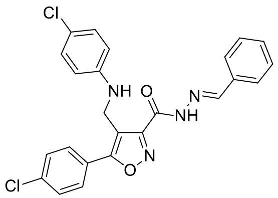

Figure S69 – ^{1}H NMR spectrum of compound **4db** in DMSO-d_6 at 300.06 MHz.

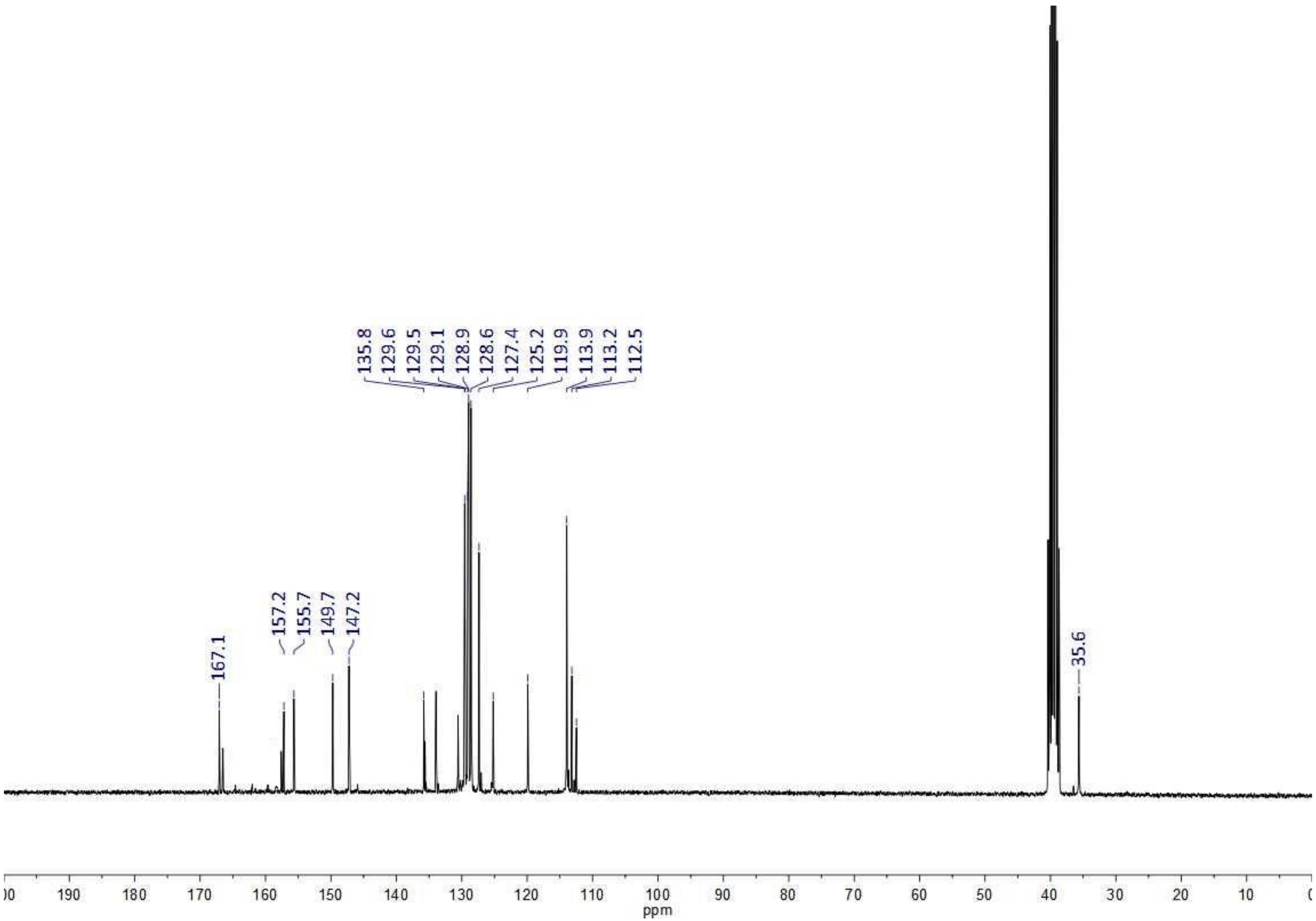

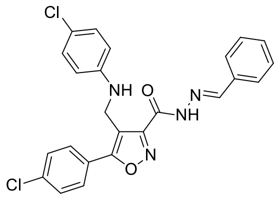

Figure S70 – ^{13}C NMR spectrum of compound **4db** in DMSO-d_6 at 75.45 MHz.

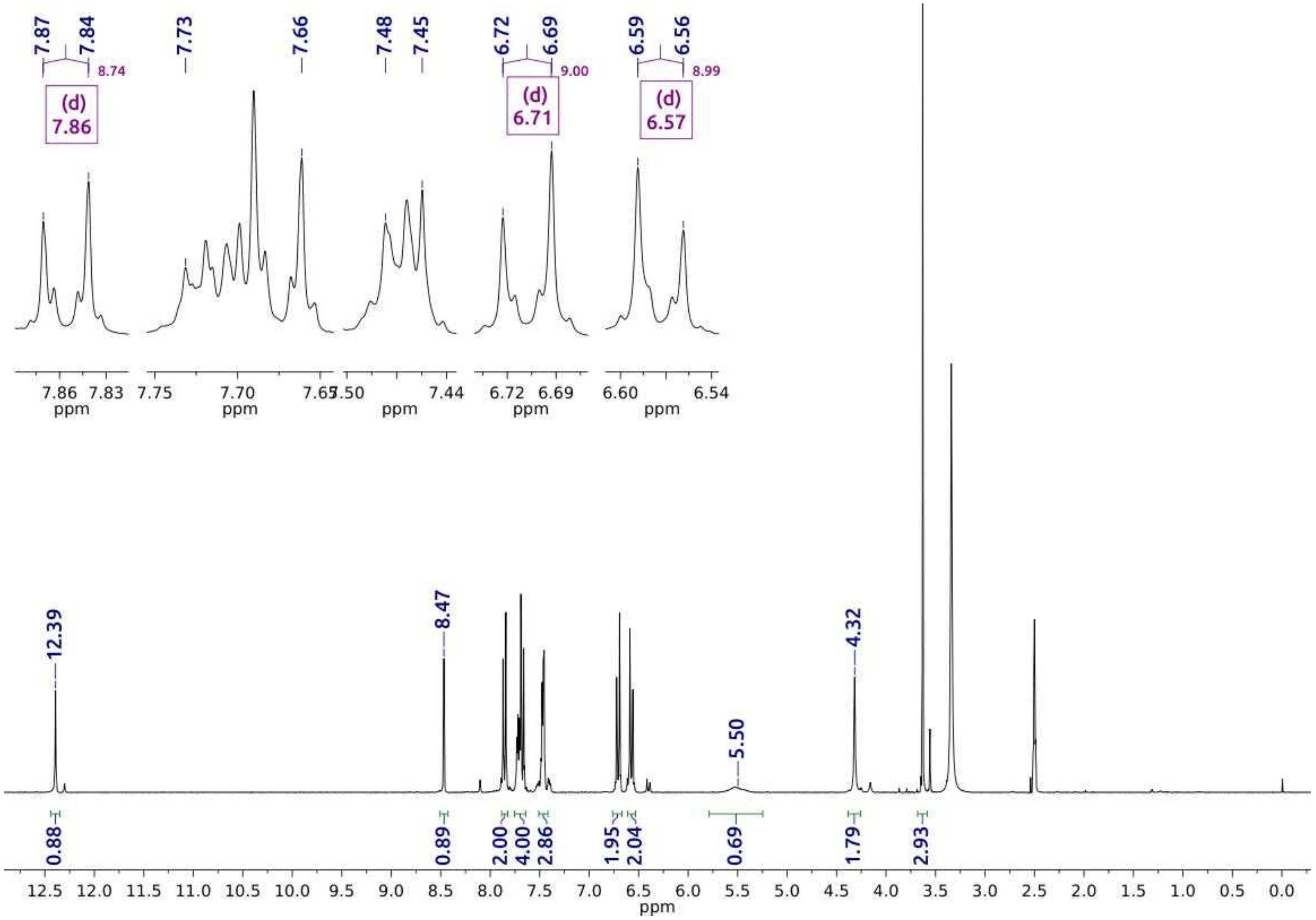

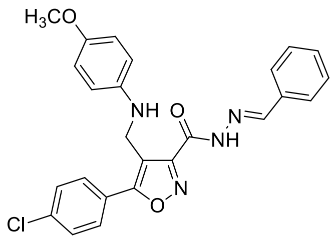

Figure S71 – ^{1}H NMR spectrum of compound **4dc** in DMSO-d_6 at 300.06 MHz.

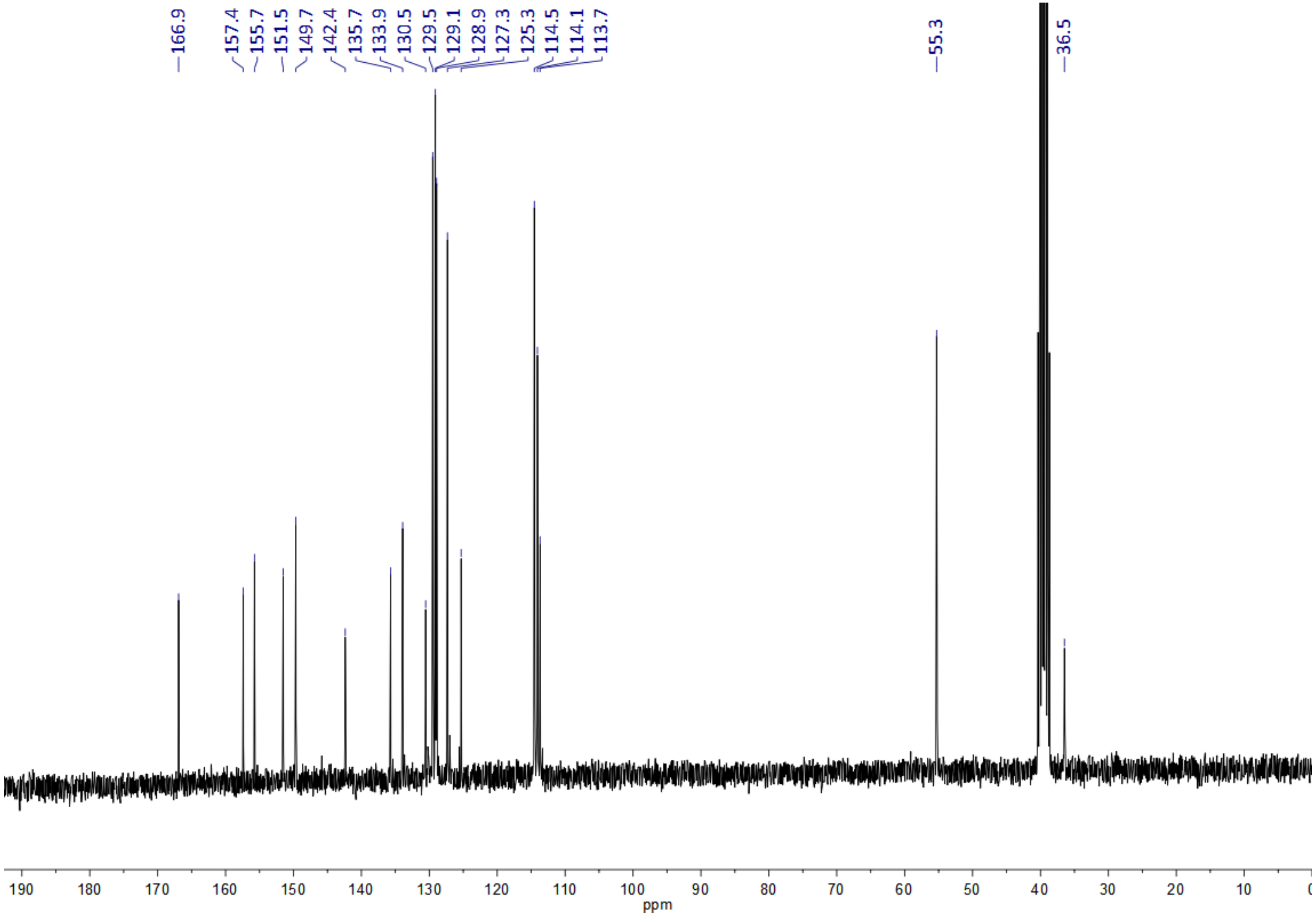

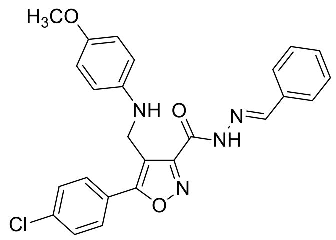

Figure S72 – ^{13}C NMR spectrum of compound **4dc** in DMSO-d_6 at 75.45 MHz.

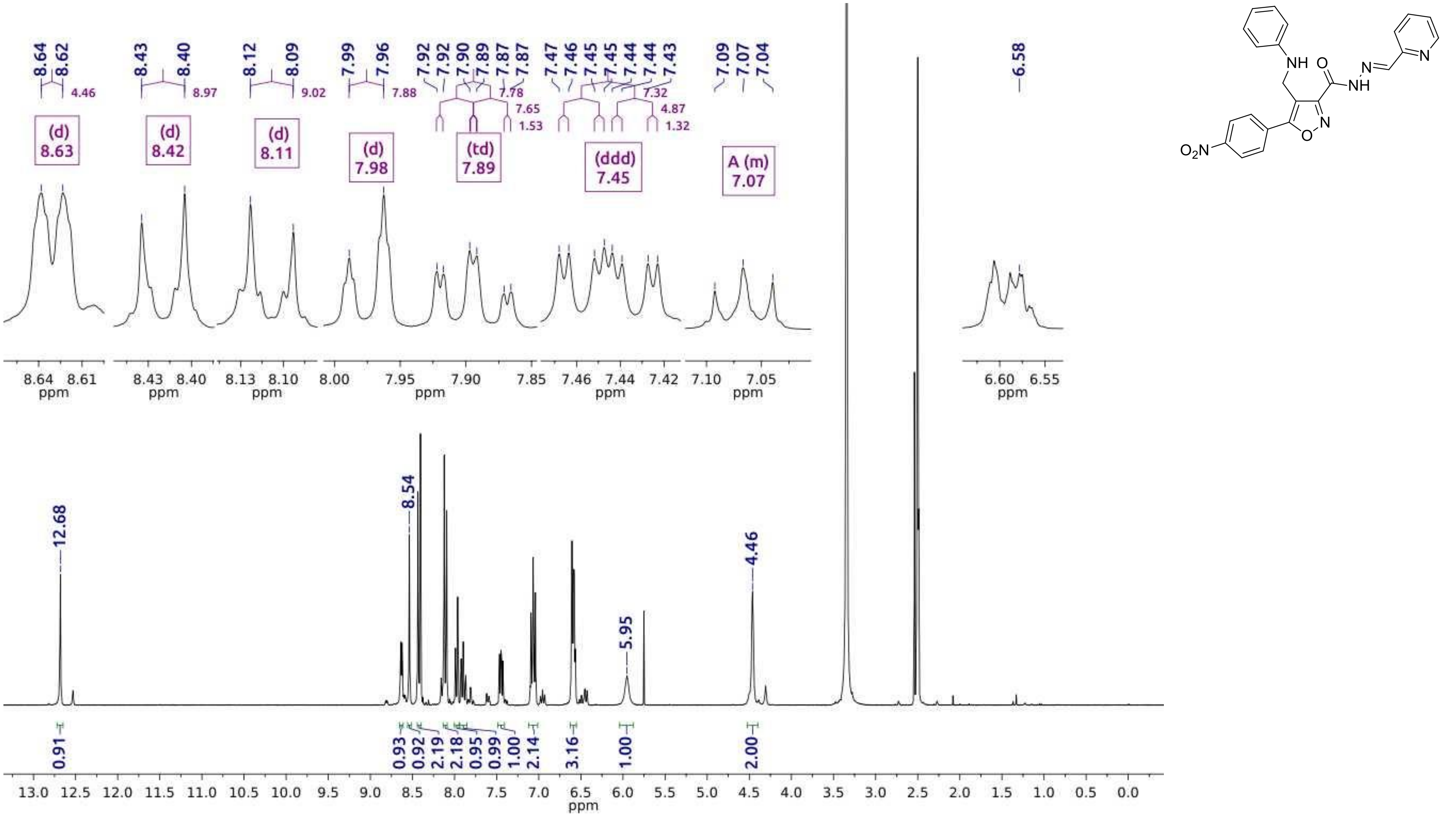

Figure S73 – ^{1}H NMR spectrum of compound **5aa** in DMSO-d_6 at 300.06 MHz.

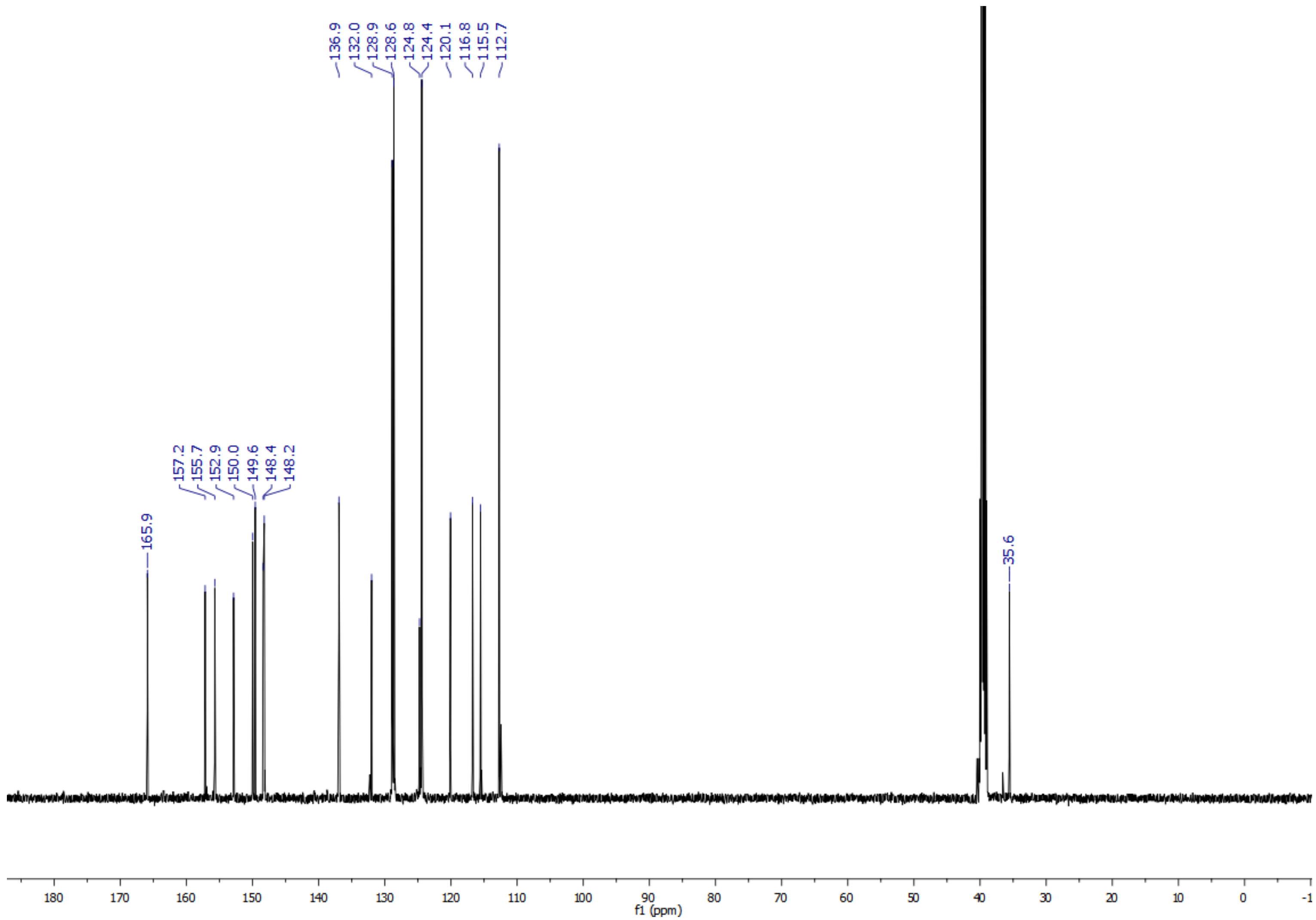

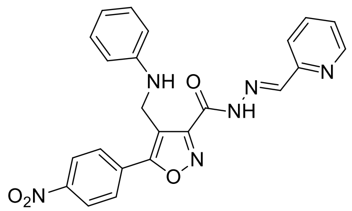

Figure S74 – ^{13}C NMR spectrum of compound **5aa** in DMSO-d_6 at 75.45 MHz.

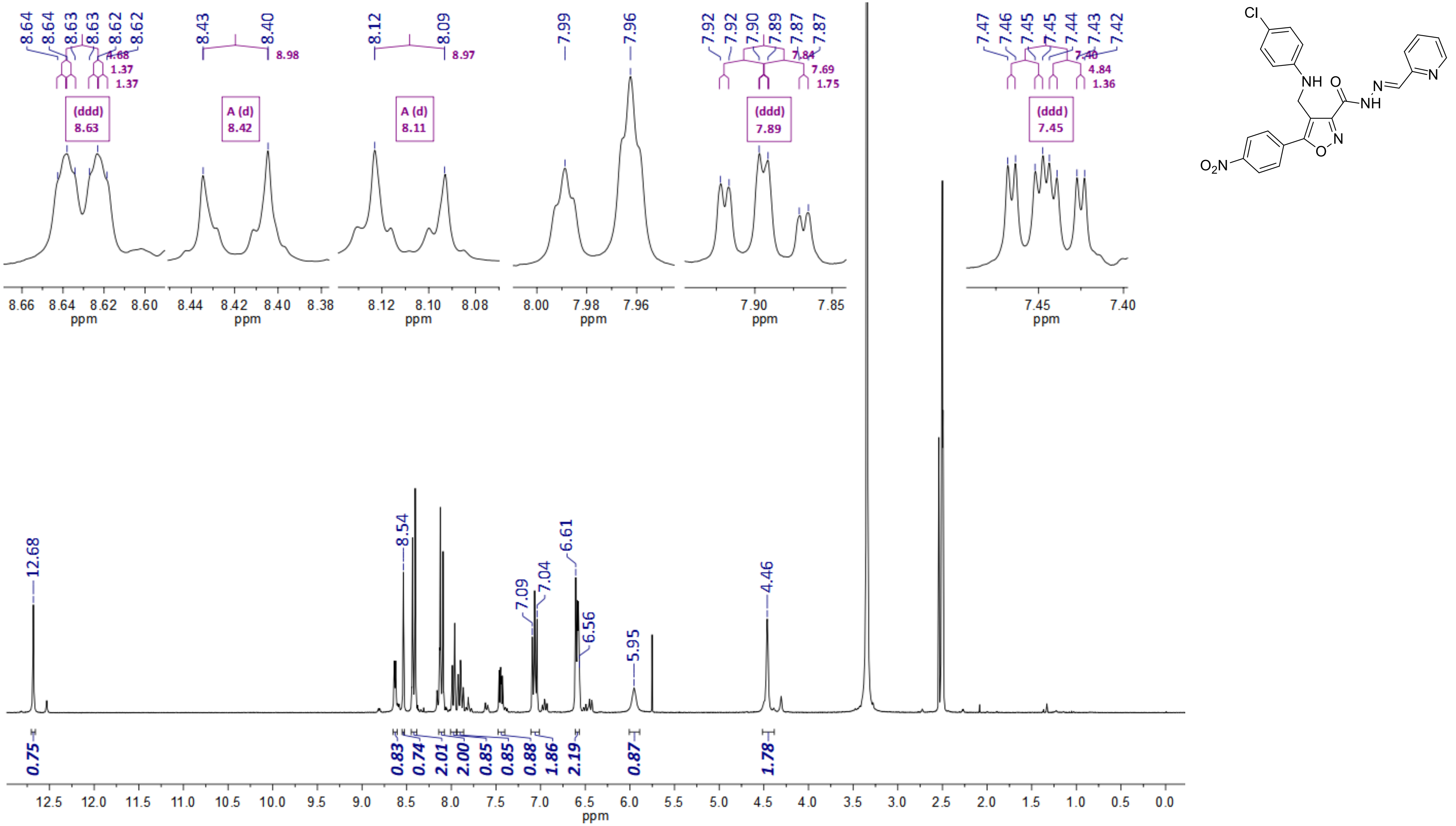

Figure S75 – ^{1}H NMR spectrum of compound **5ab** in DMSO-d_6 at 300.06 MHz.

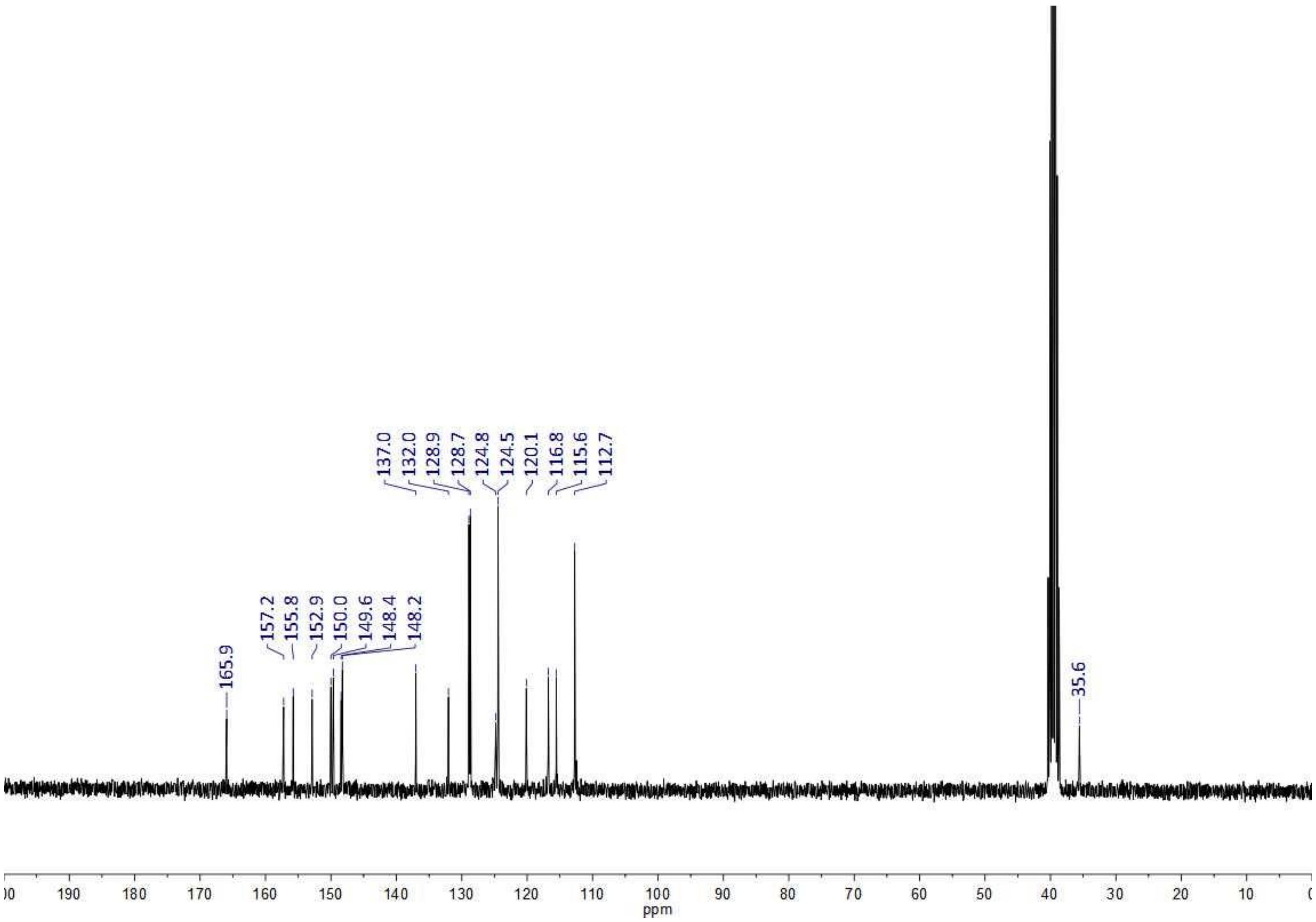

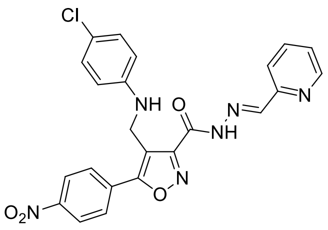

Figure S76 – ^{13}C NMR spectrum of compound **5ab** in DMSO-d_6 at 75.45 MHz.

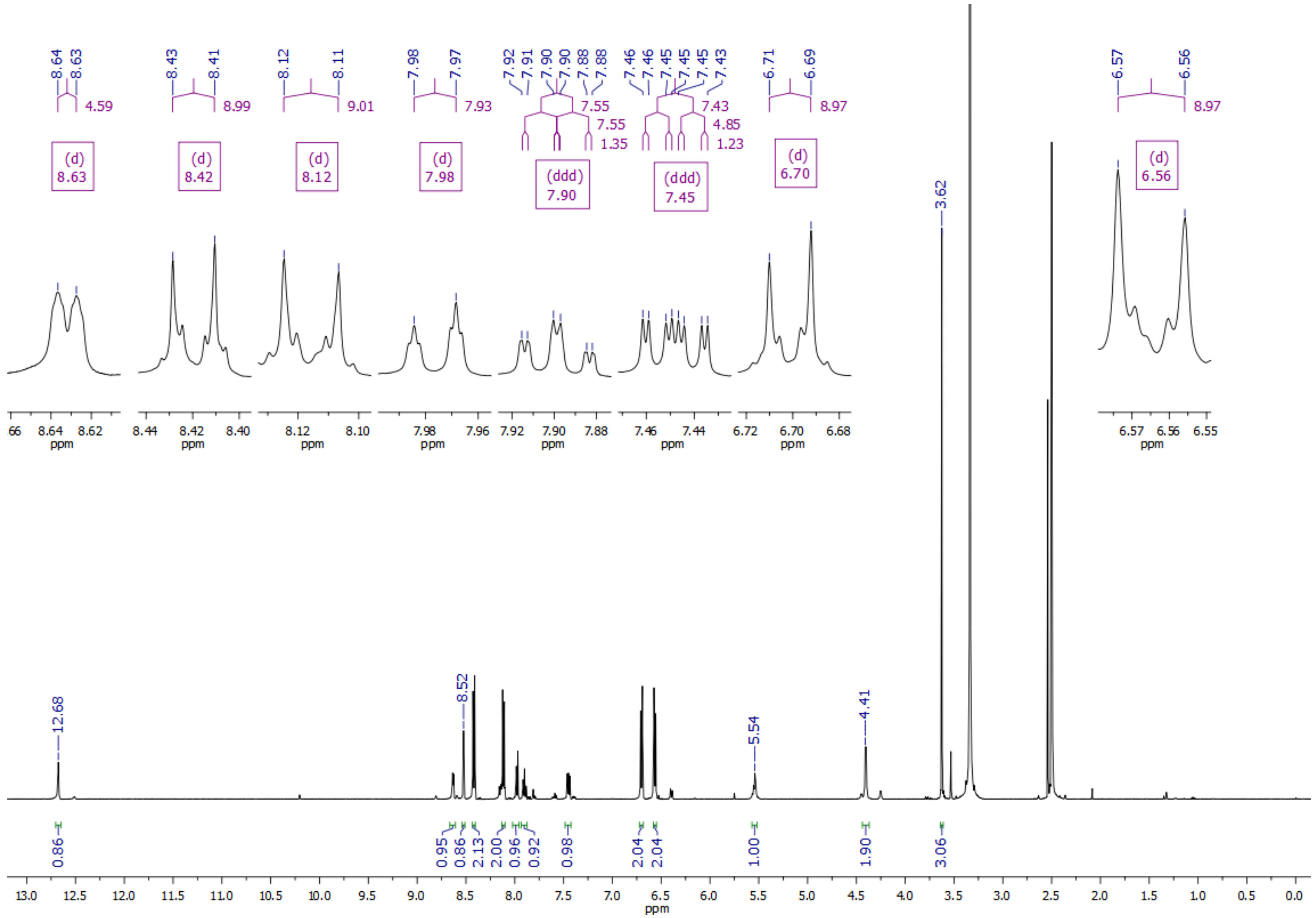

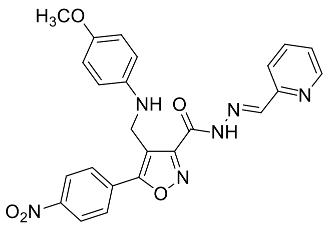

Figure S77 – ^{1}H NMR spectrum of compound **5ac** in DMSO-d_6 at 300.06 MHz.

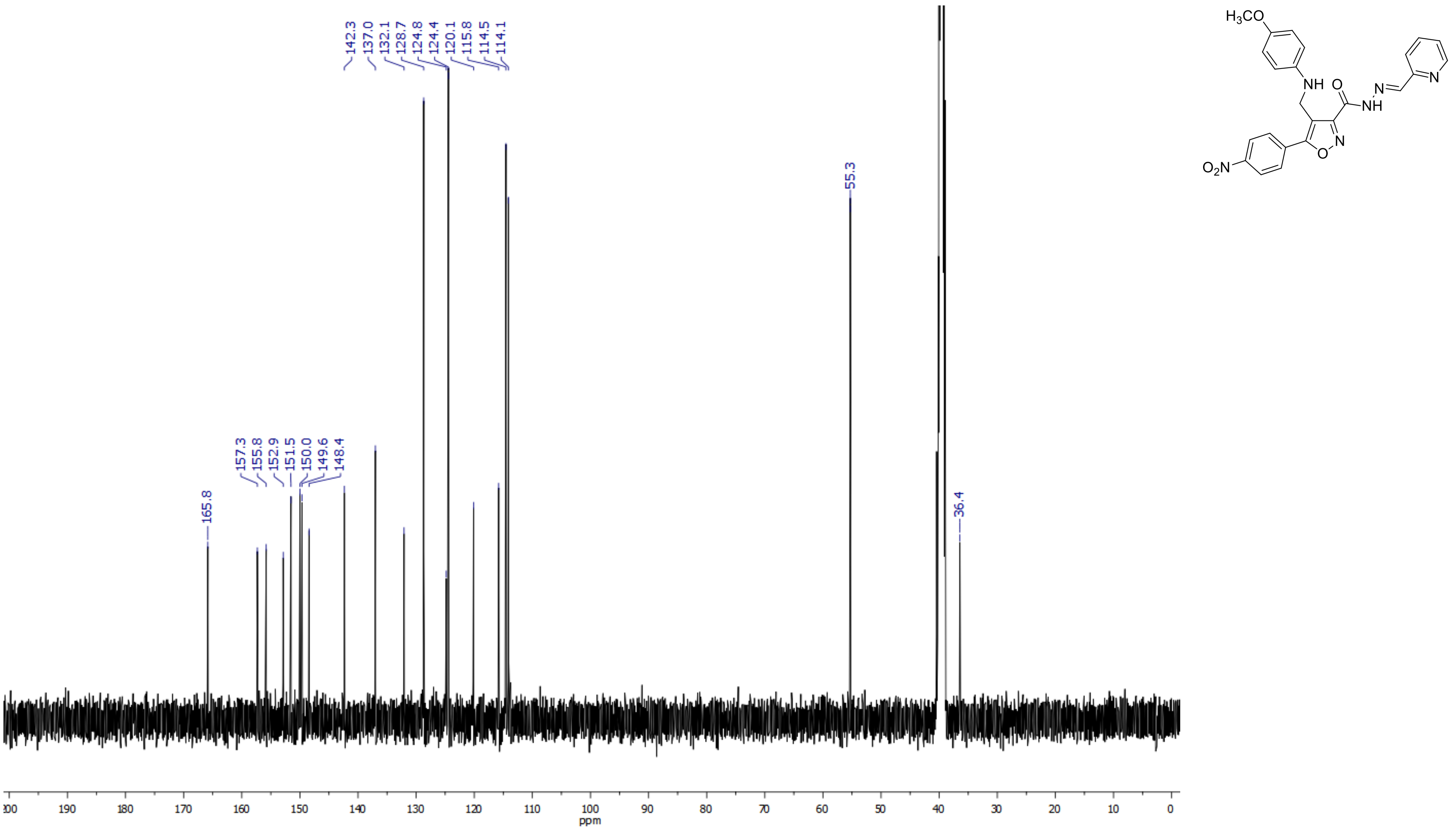

Figure S78 – ^{13}C NMR spectrum of compound **5ac** in DMSO-d_6 at 75.45 MHz.

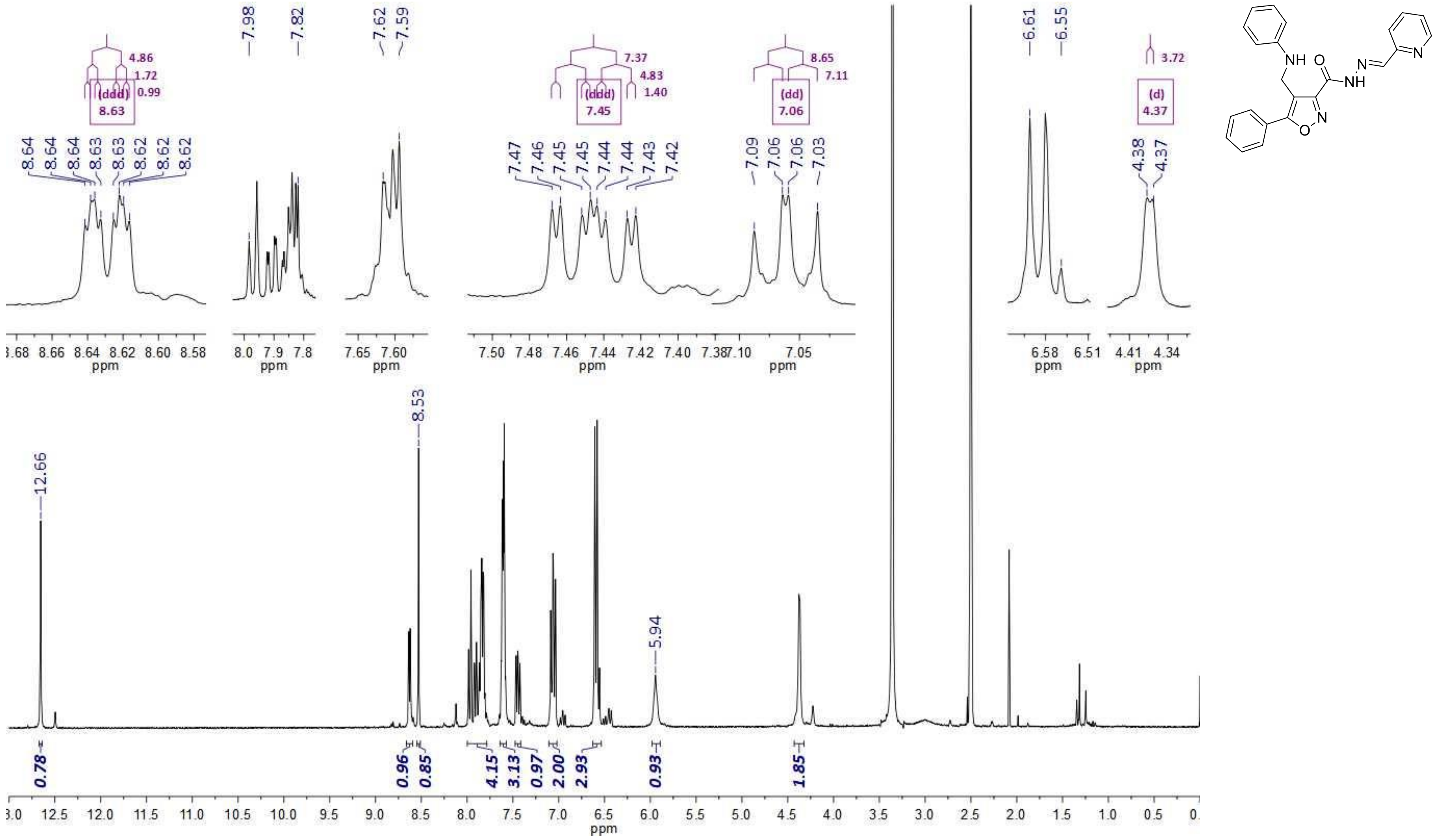

Figure S79 – ^{1}H NMR spectrum of compound **5ba** in DMSO-d_6 at 300.06 MHz.

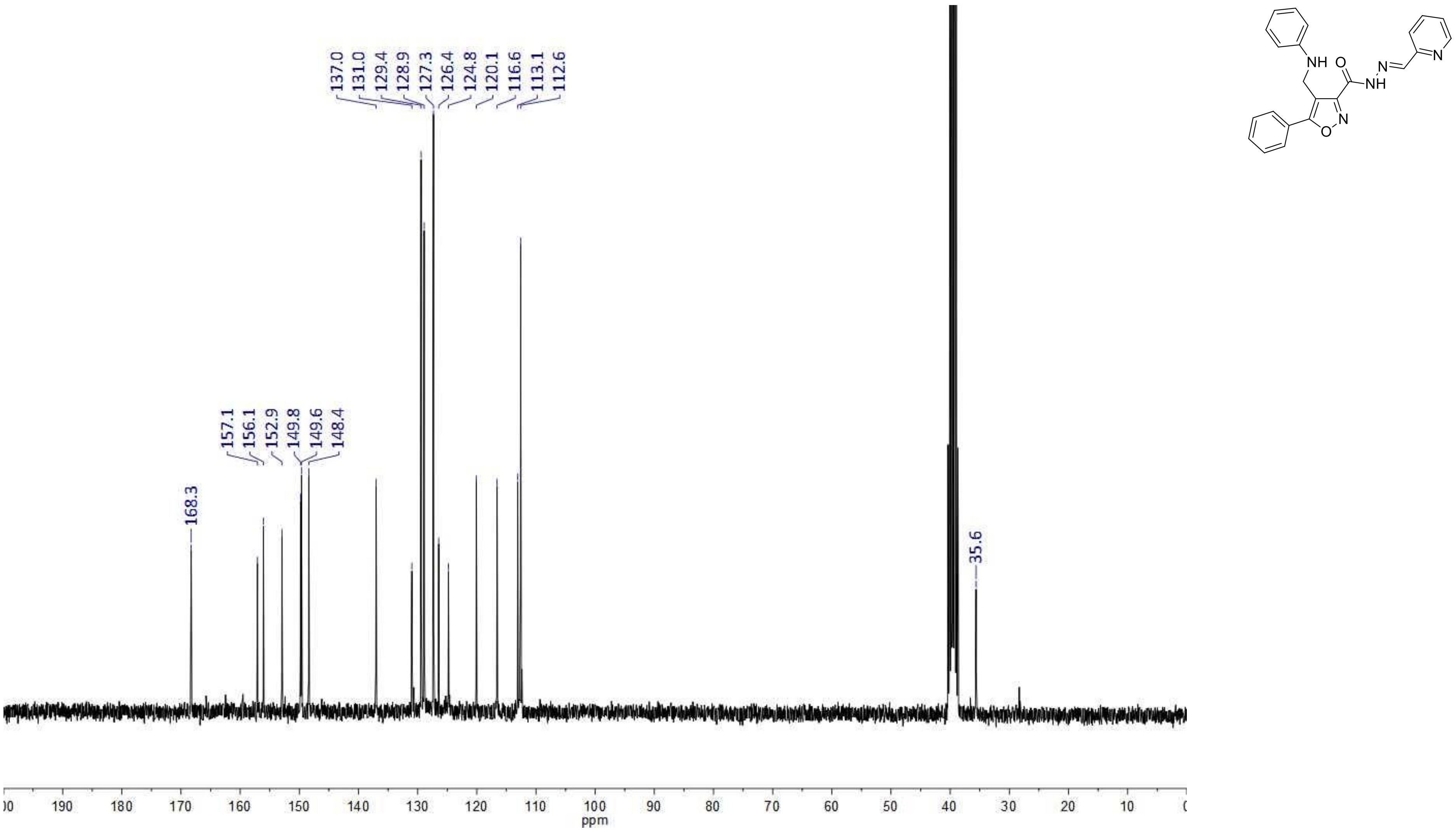

Figure S80 – ^{13}C NMR spectrum of compound **5ba** in DMSO-d_6 at 75.45 MHz.

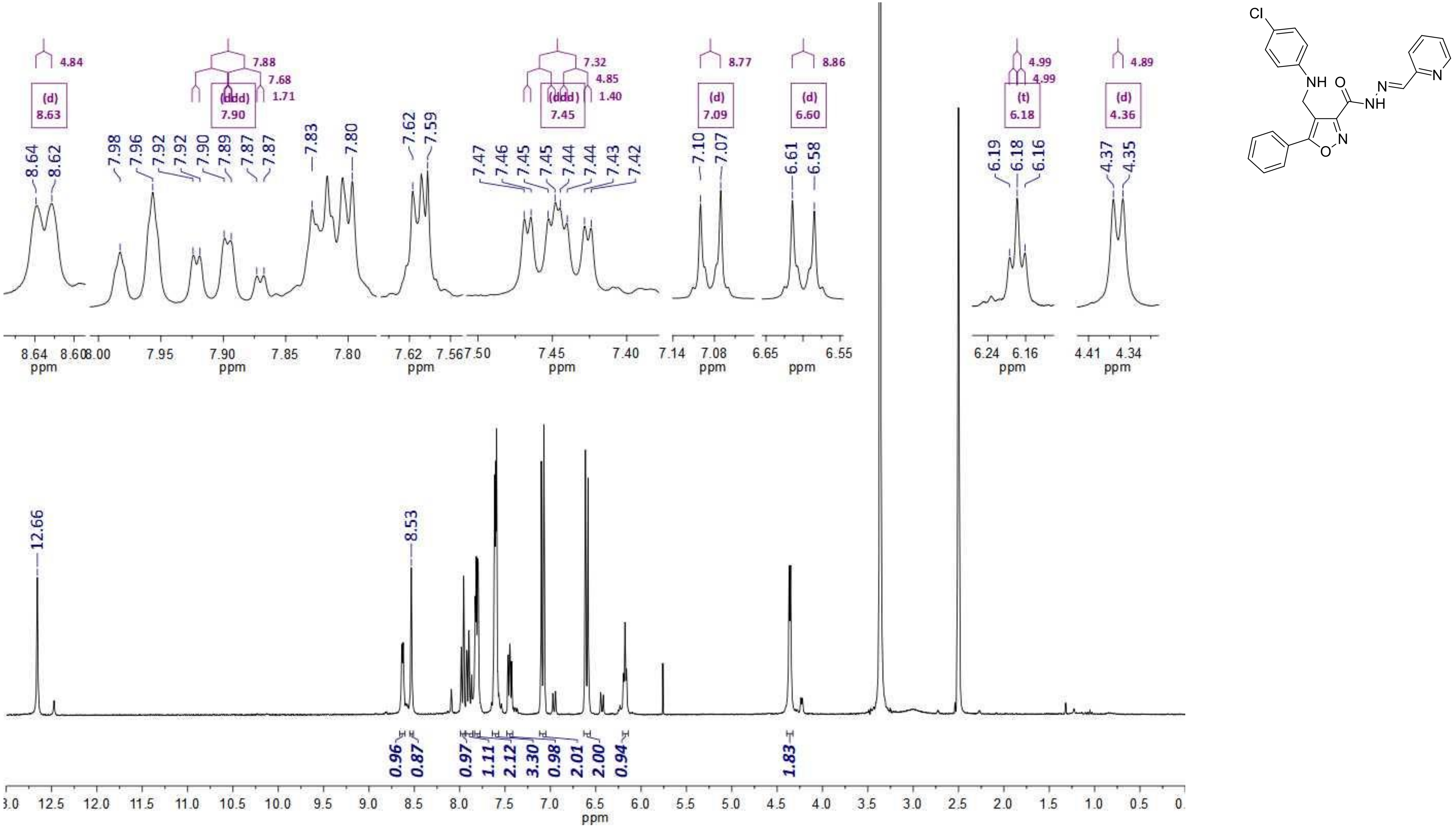

Figure S81 – ^{1}H NMR spectrum of compound **5bb** in DMSO-d_6 at 300.06 MHz.

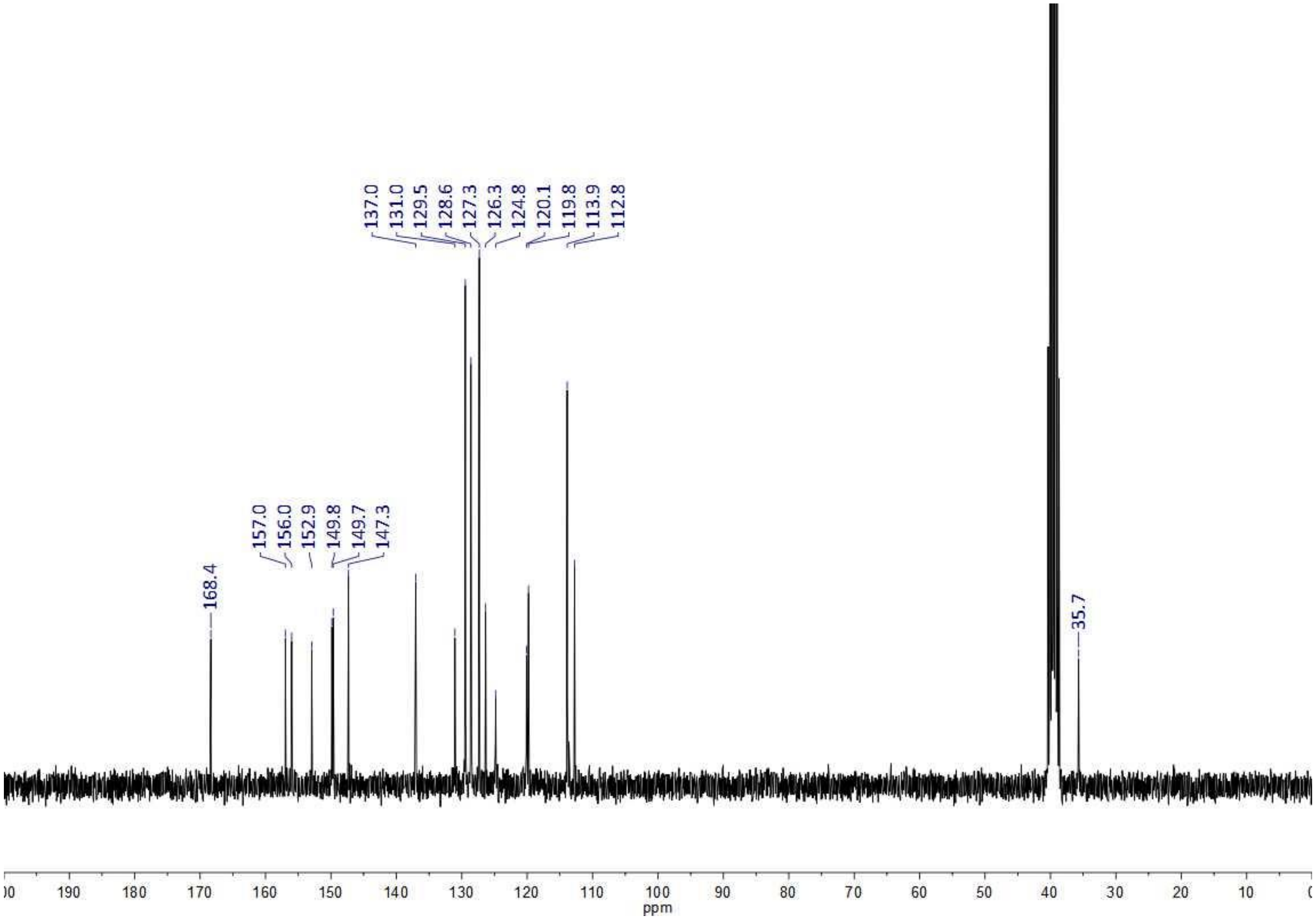

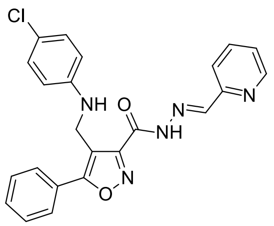

Figure S82 – ^{13}C NMR spectrum of compound **5bb** in DMSO-d_6 at 75.45 MHz.

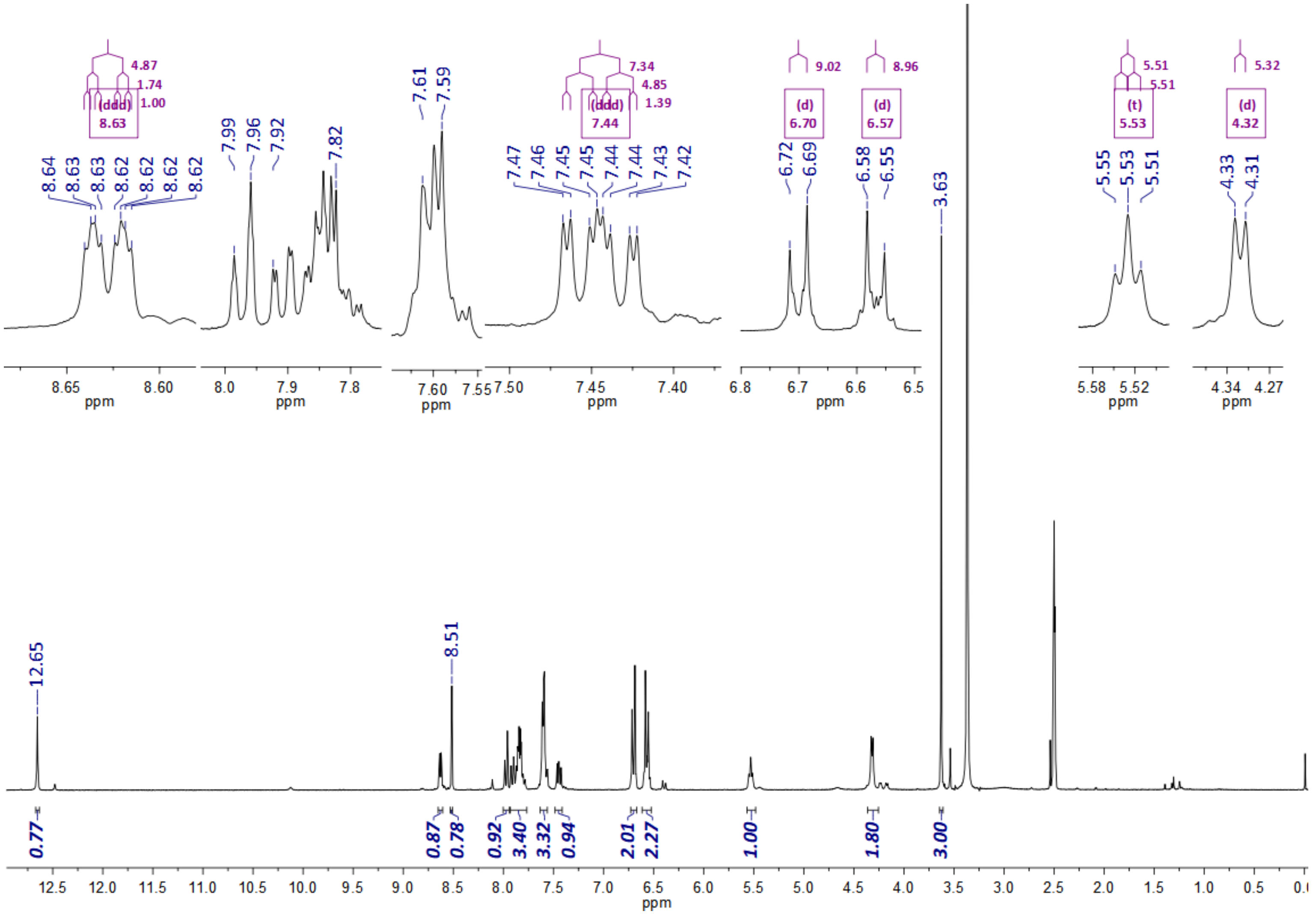

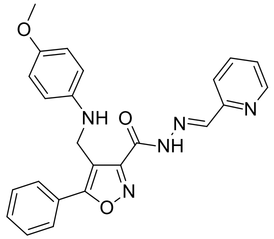

Figure S83 – ^{1}H NMR spectrum of compound **5bc** in DMSO-d_6 at 300.06 MHz.

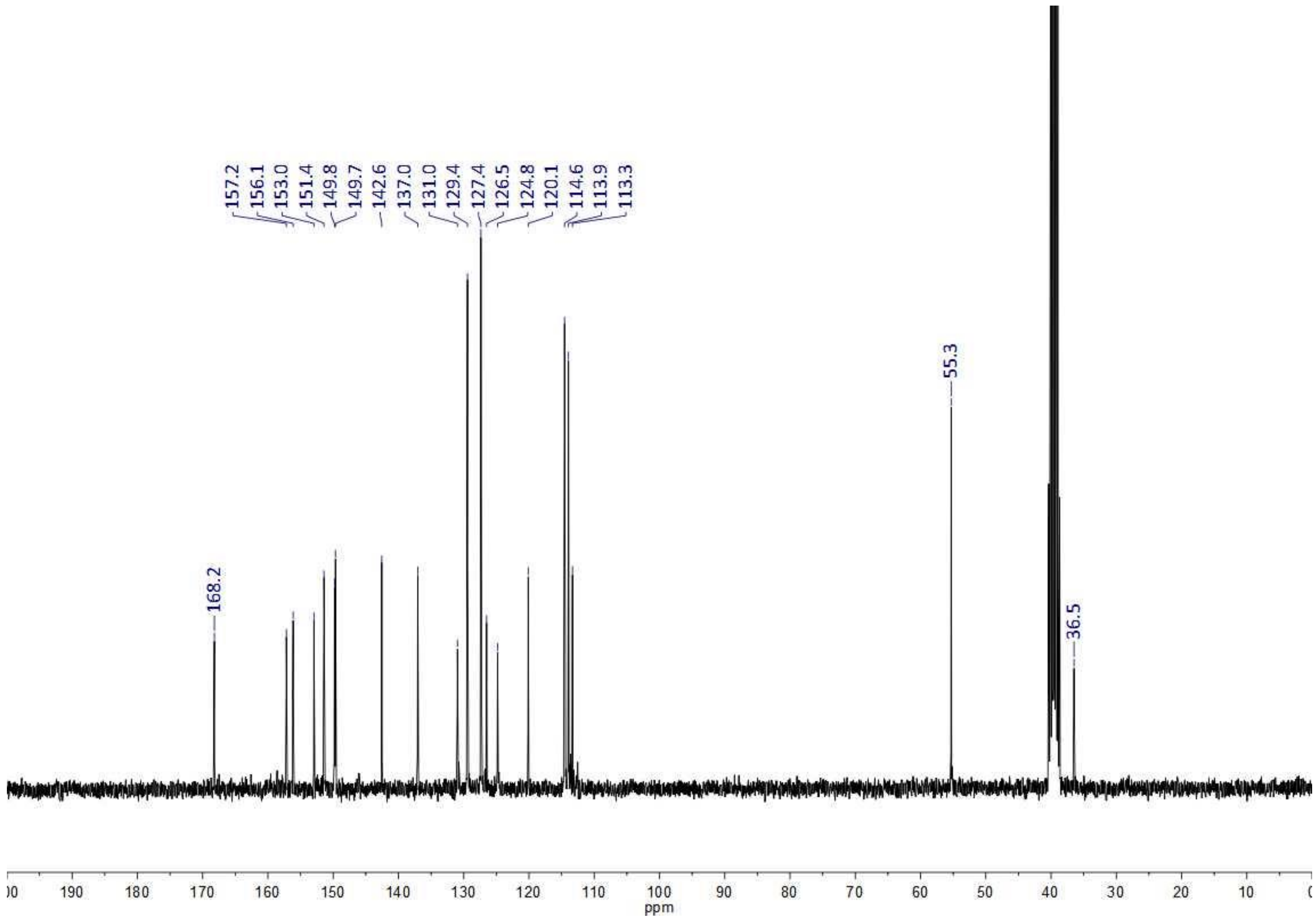

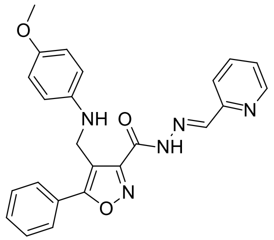

Figure S84 – ^{13}C NMR spectrum of compound **5bc** in DMSO-d_6 at 75.45 MHz.

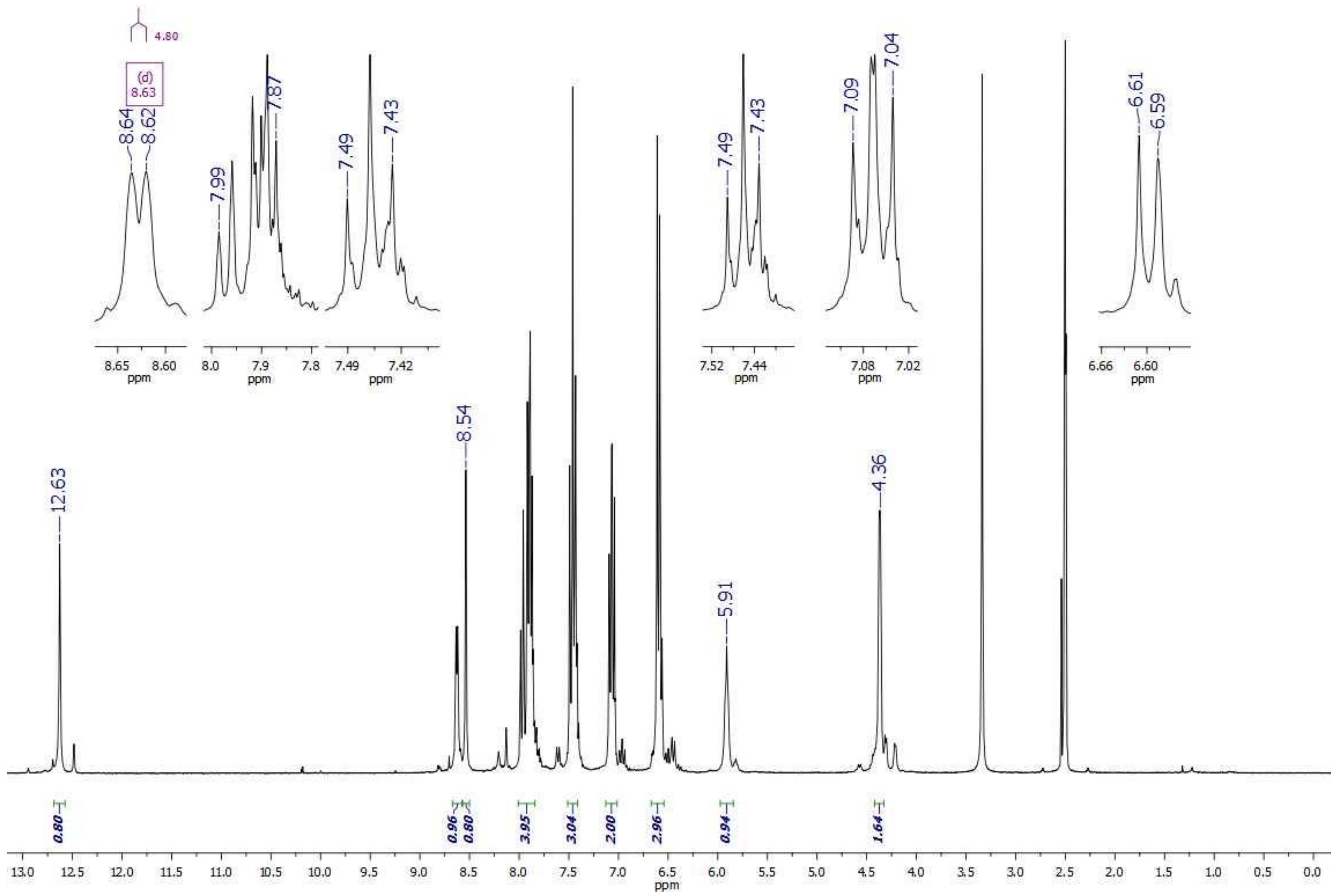

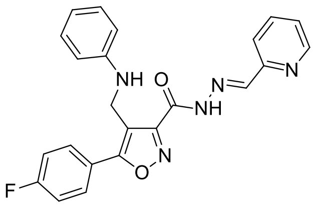

Figure S85 – ^{1}H NMR spectrum of compound **5ca** in DMSO-d_6 at 300.06 MHz.

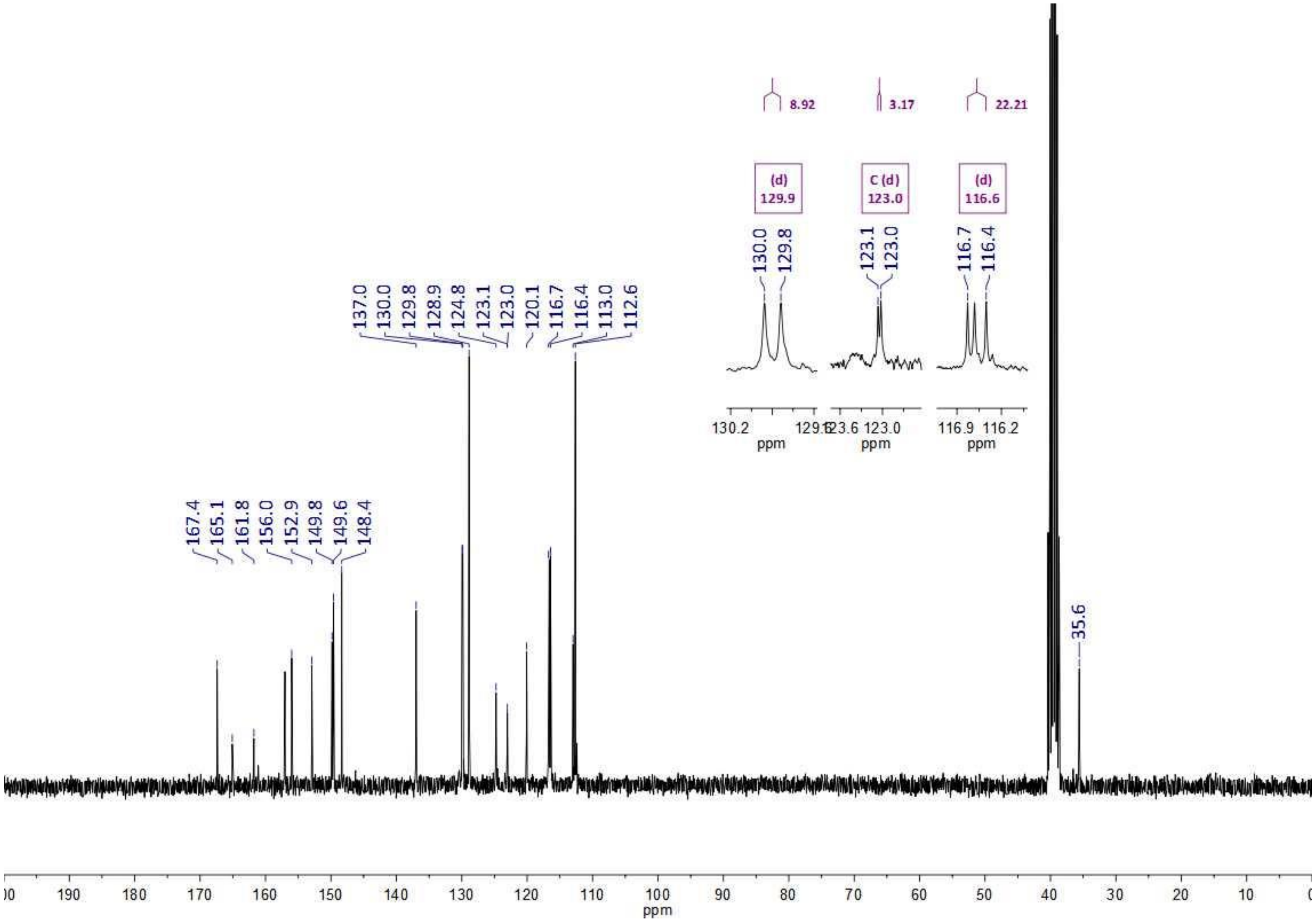

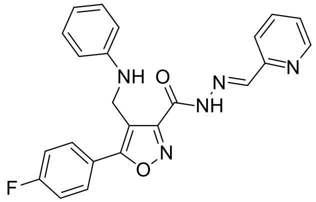

Figure S86 – ^{13}C NMR spectrum of compound **5ca** in DMSO-d_6 at 75.45 MHz.

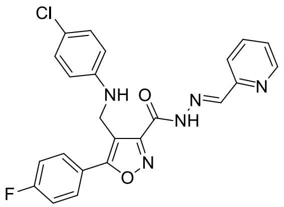

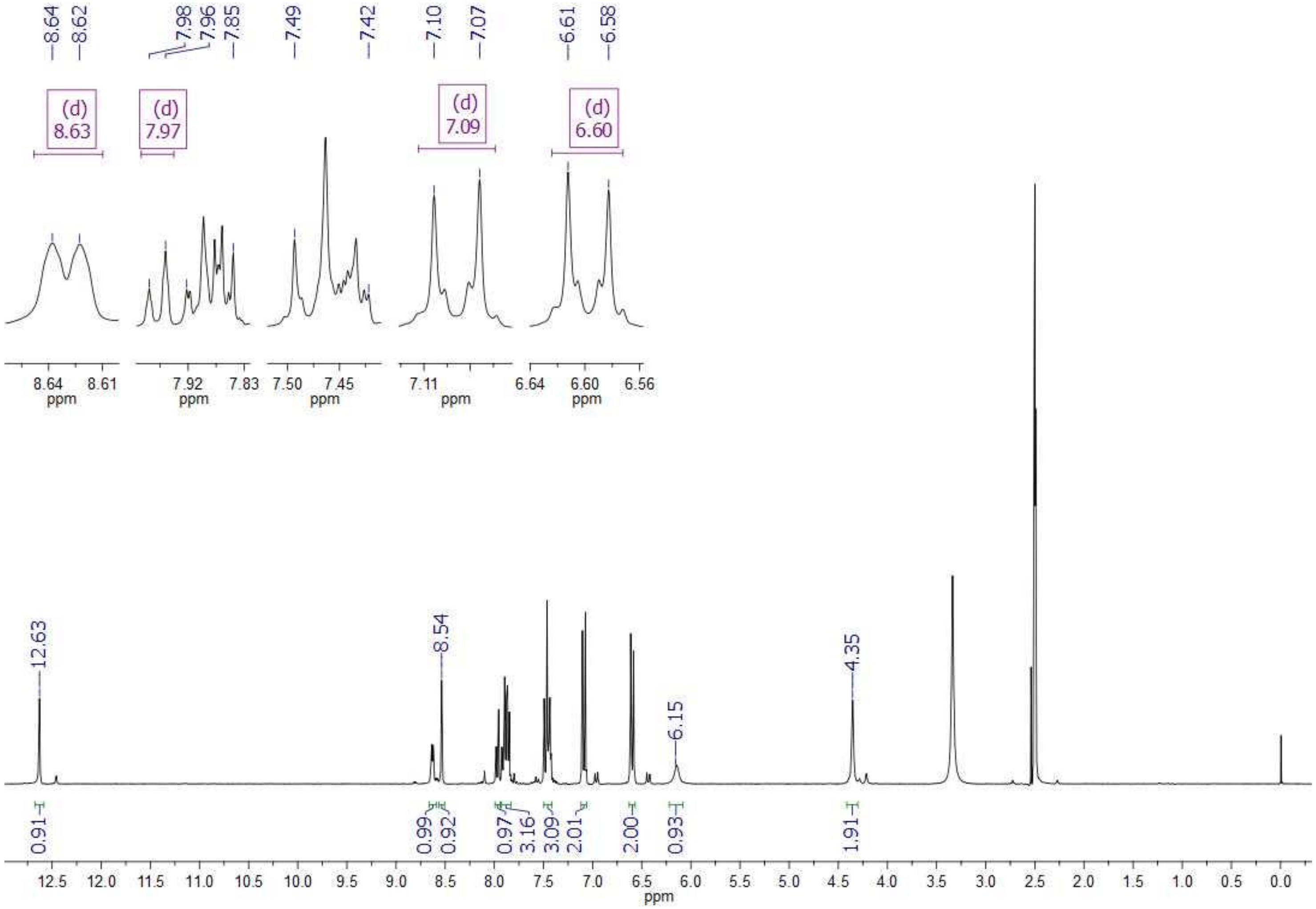

Figure S87 – ^{1}H NMR spectrum of compound **5cb** in DMSO-d_6 at 300.06 MHz.

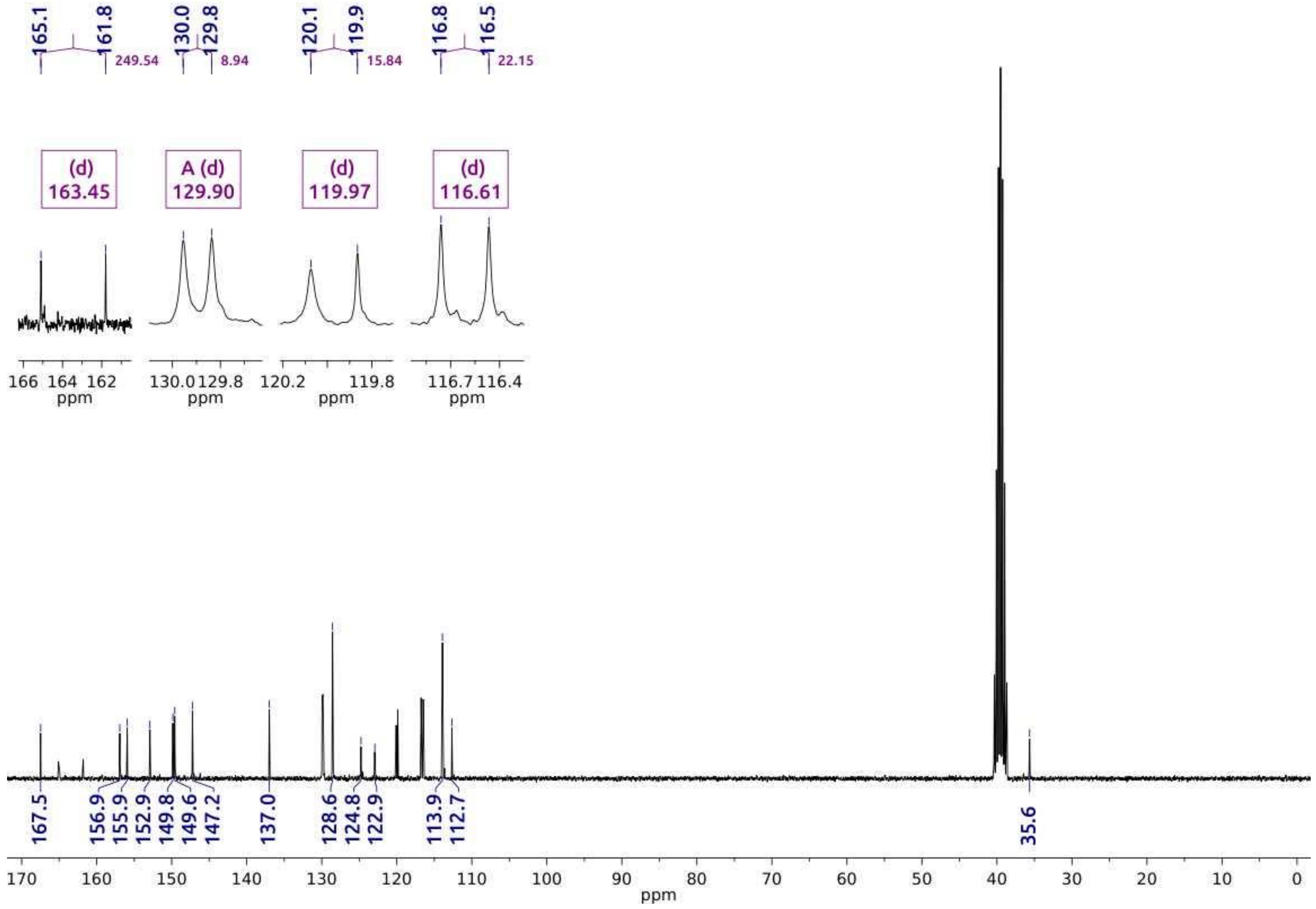

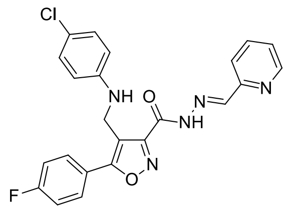

Figure S88 – ^{13}C NMR spectrum of compound **5cb** in DMSO-d_6 at 75.45 MHz.

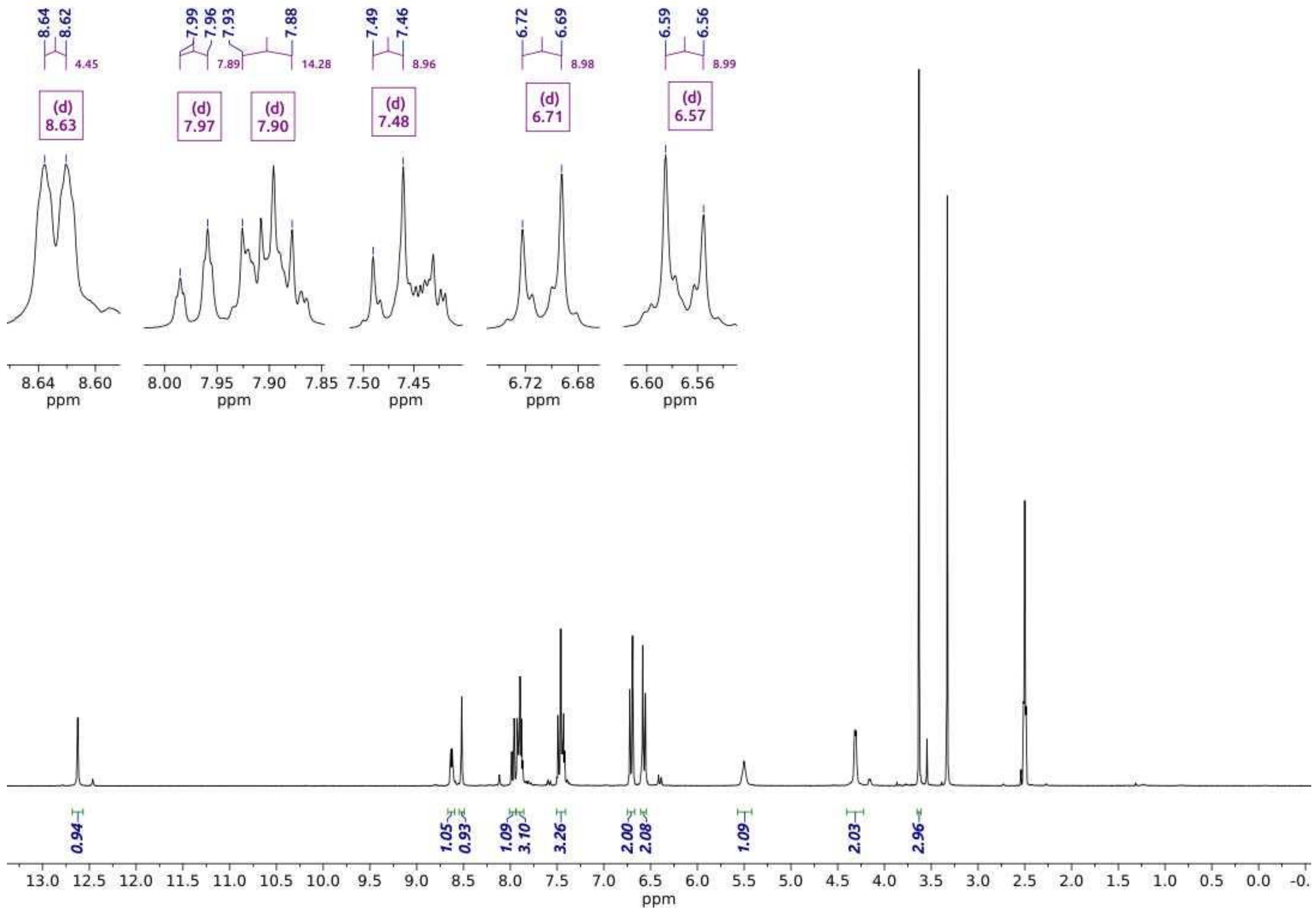

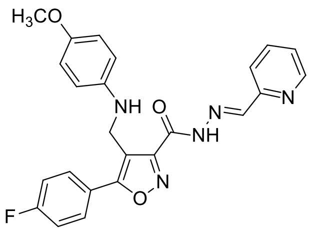

Figure S89 – ^{1}H NMR spectrum of compound **5cc** in DMSO-d_6 at 300.06 MHz.

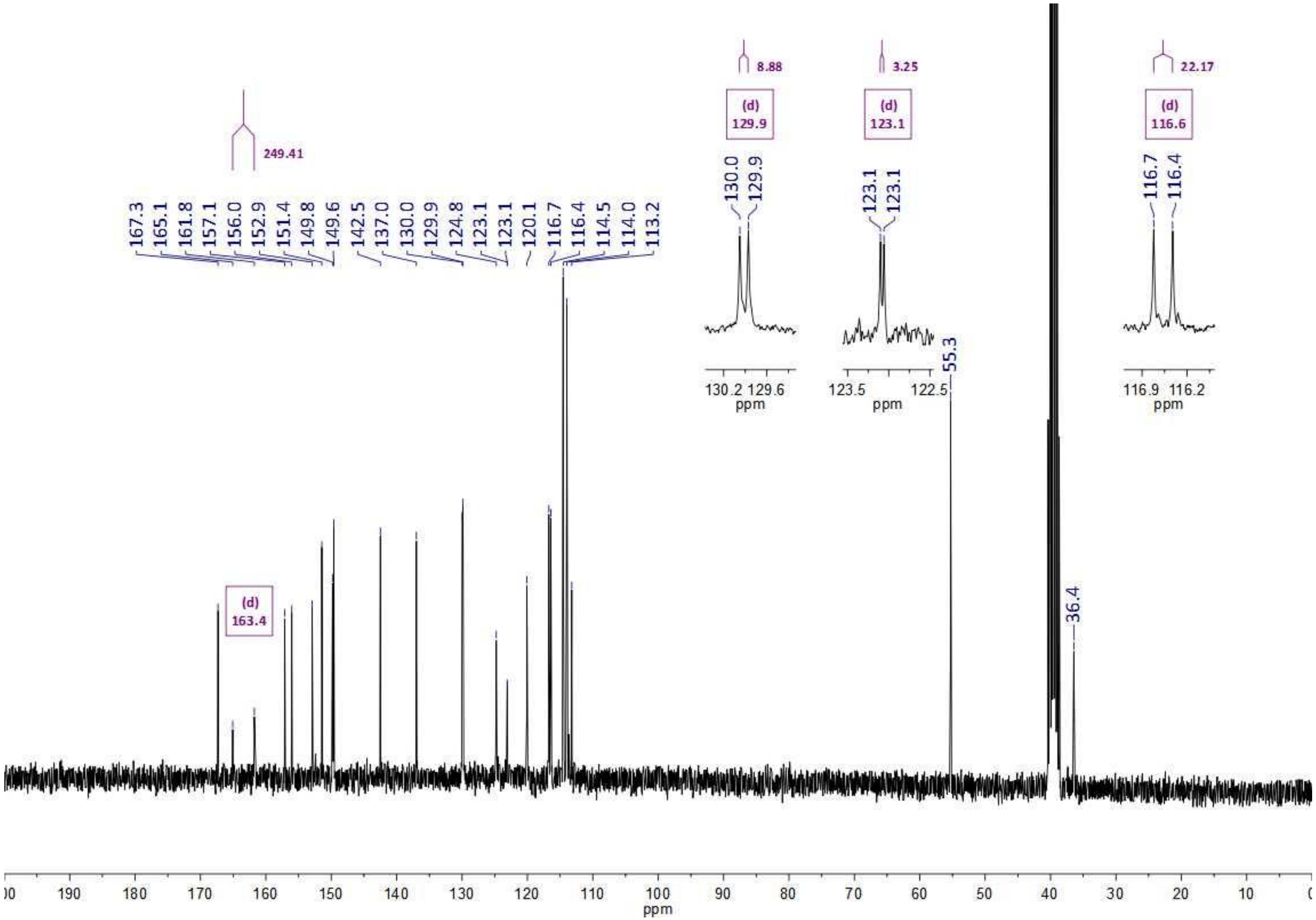

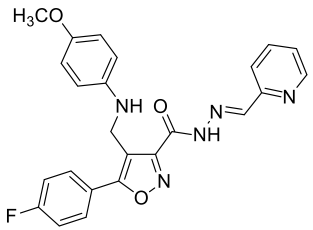

Figure S90 – ^{13}C NMR spectrum of compound **5cc** in DMSO-d_6 at 75.45 MHz.

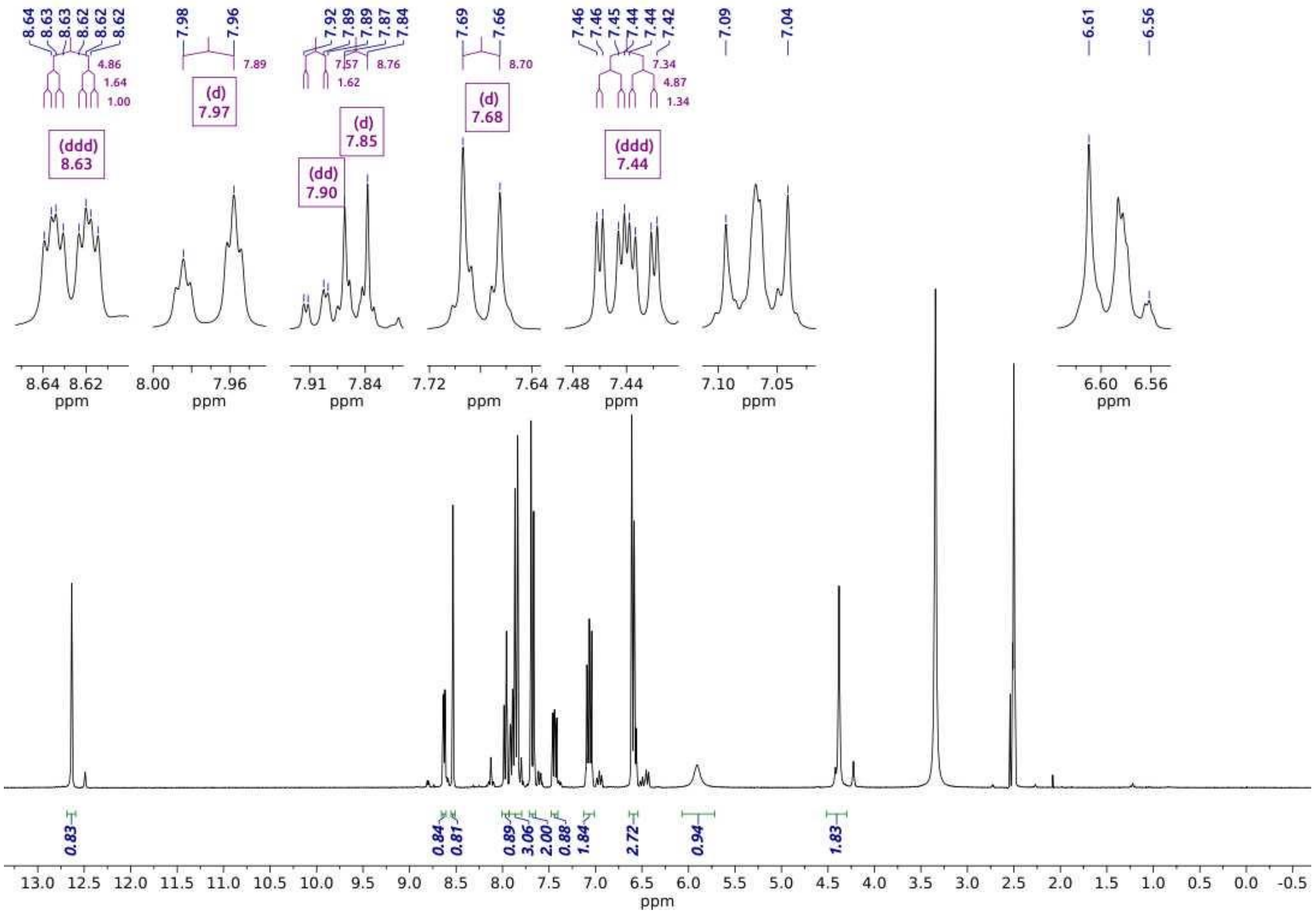

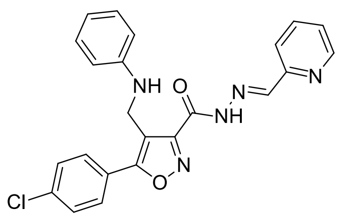

Figure S91 – ^{1}H NMR spectrum of compound **5da** in DMSO-d_6 at 300.06 MHz.

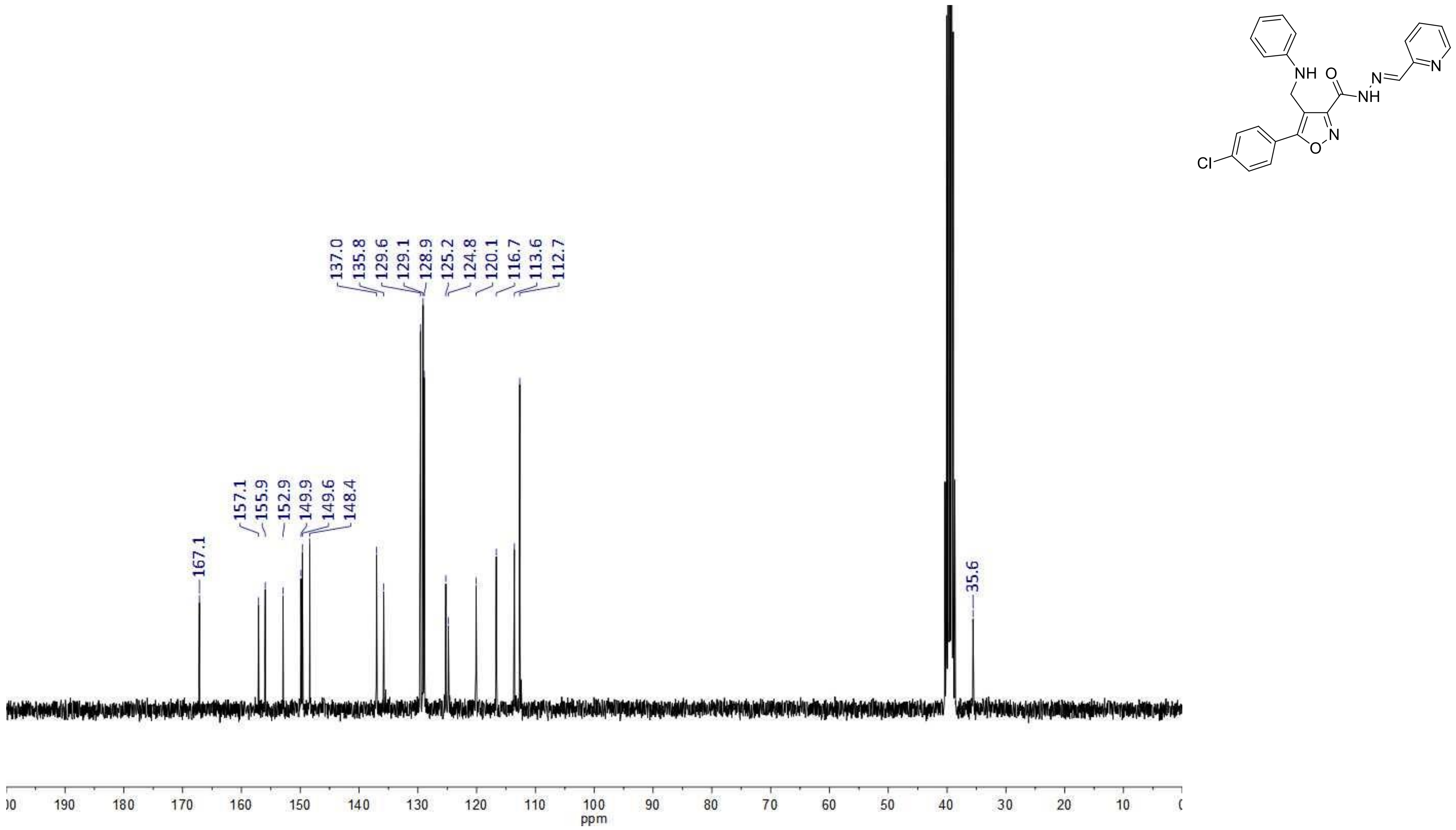

Figure S92 – ^{13}C NMR spectrum of compound **5da** in DMSO-d_6 at 75.45 MHz.

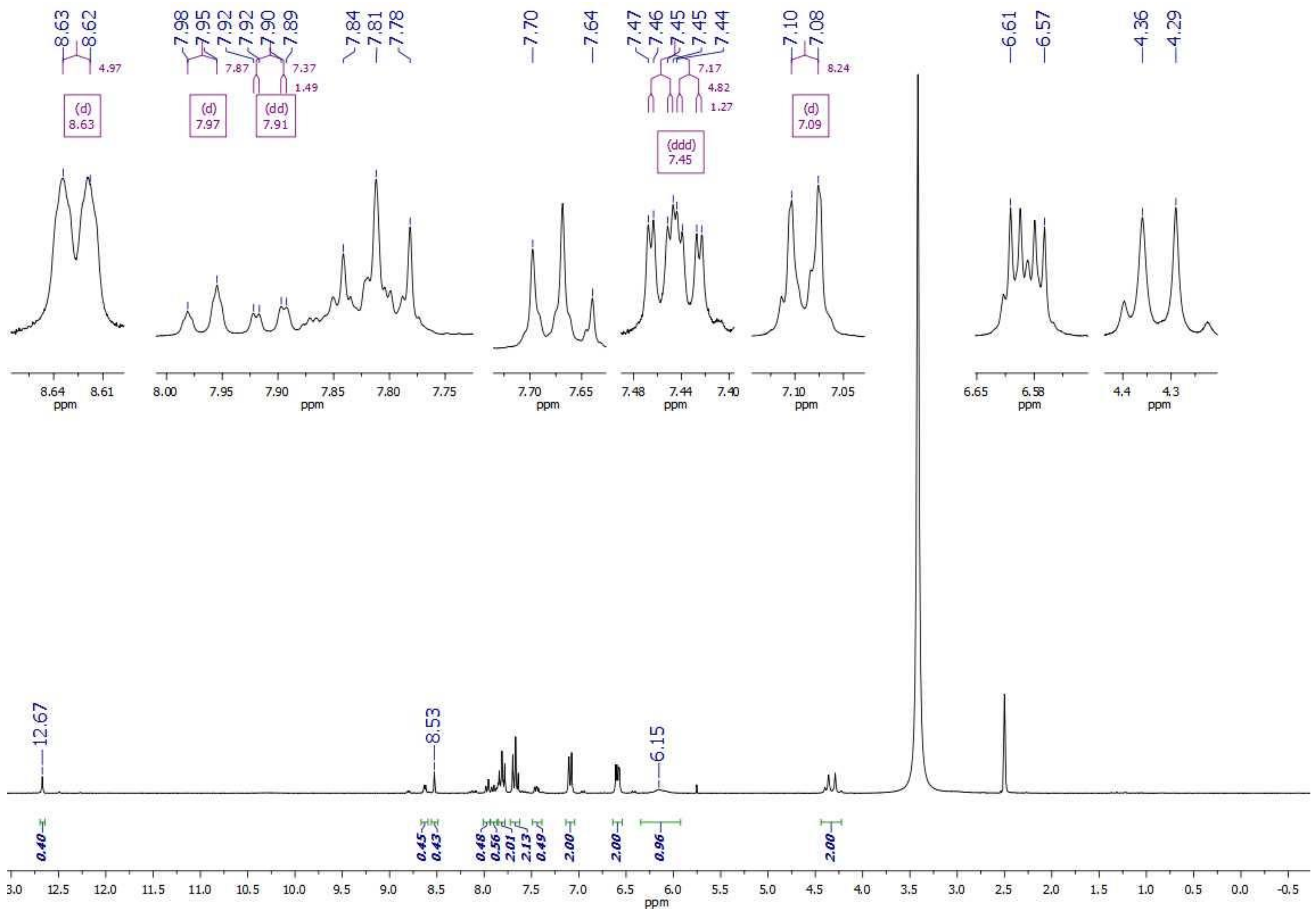

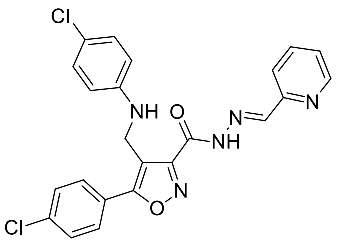

Figure S93 – ^{1}H NMR spectrum of compound **5db** in DMSO-d_6 at 300.06 MHz.

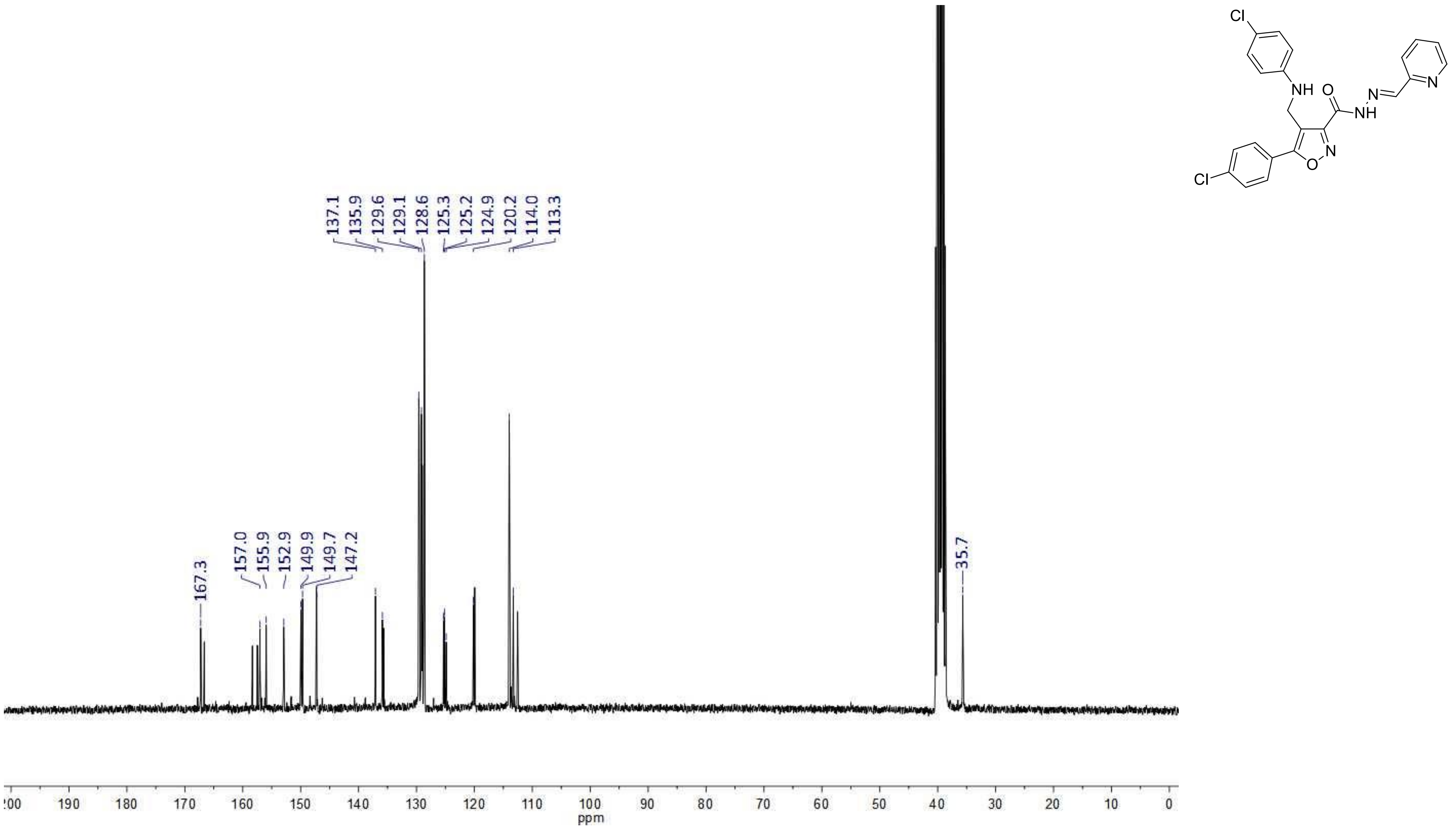

Figure S94 – ^{13}C NMR spectrum of compound **5db** in DMSO-d_6 at 75.45 MHz.

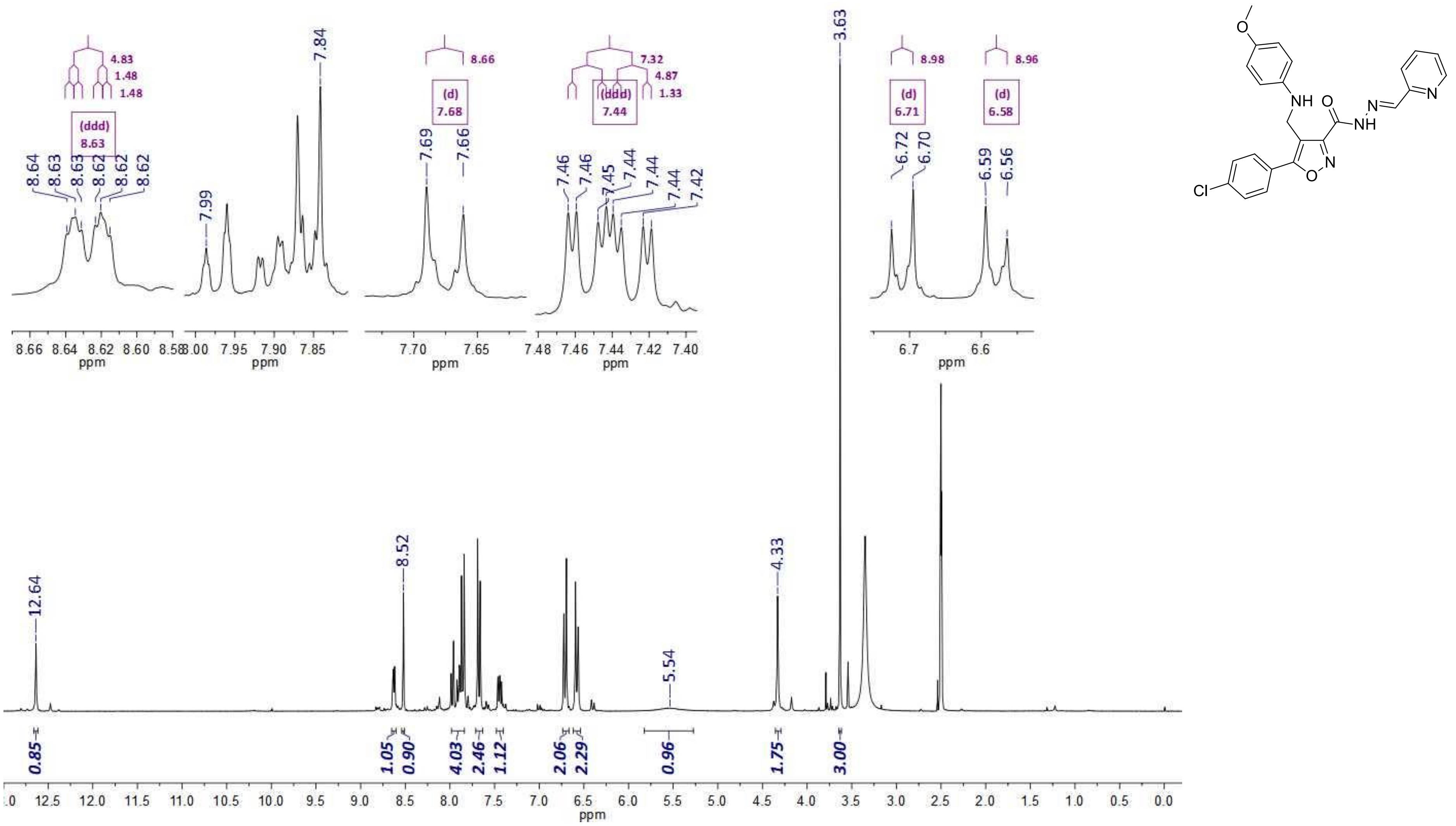

Figure S95 – ^{1}H NMR spectrum of compound **5dc** in DMSO-d_6 at 300.06 MHz.

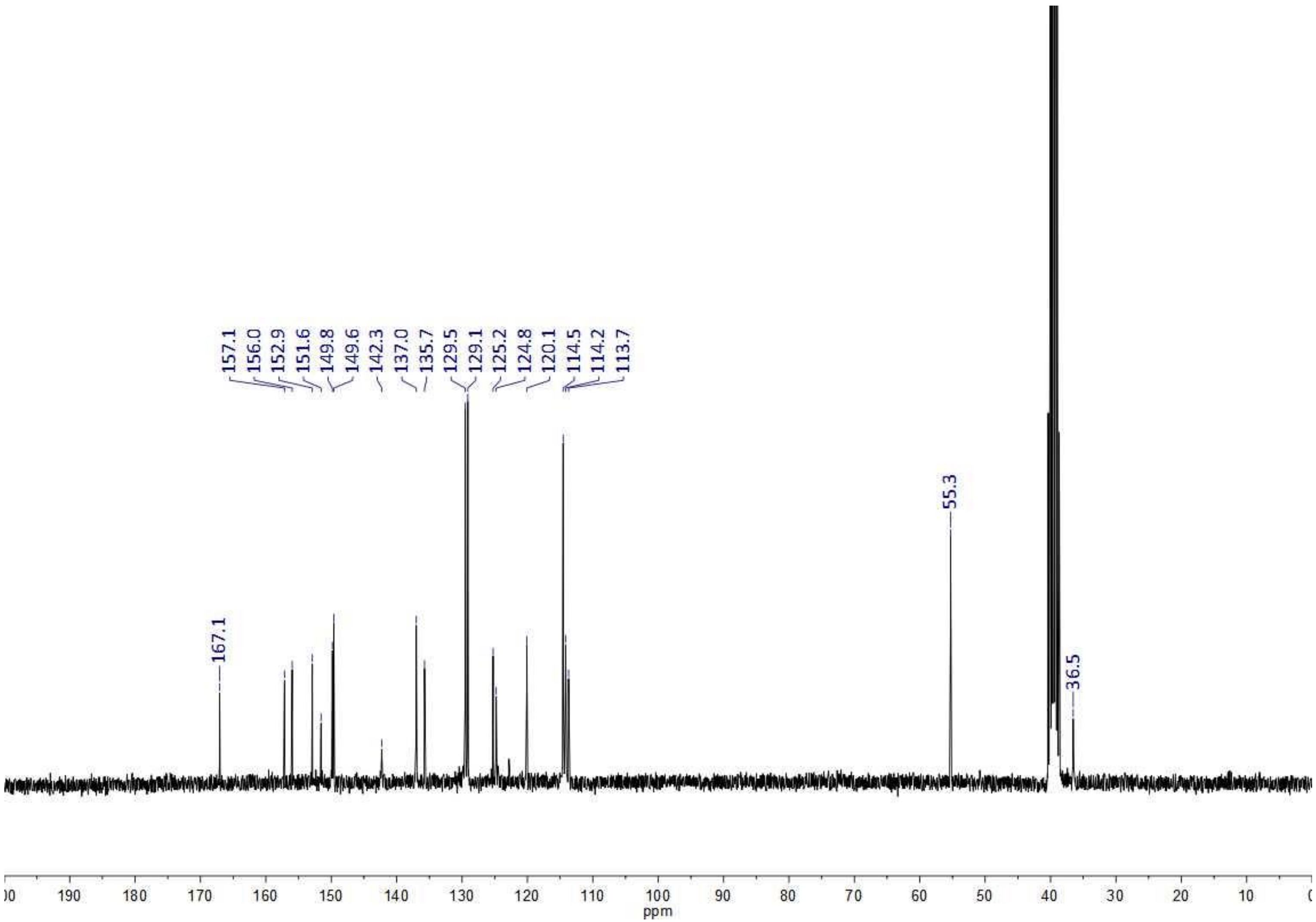

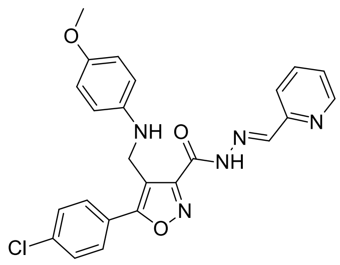

Figure S96 – ^{13}C NMR spectrum of compound **5dc** in DMSO-d_6 at 75.45 MHz.

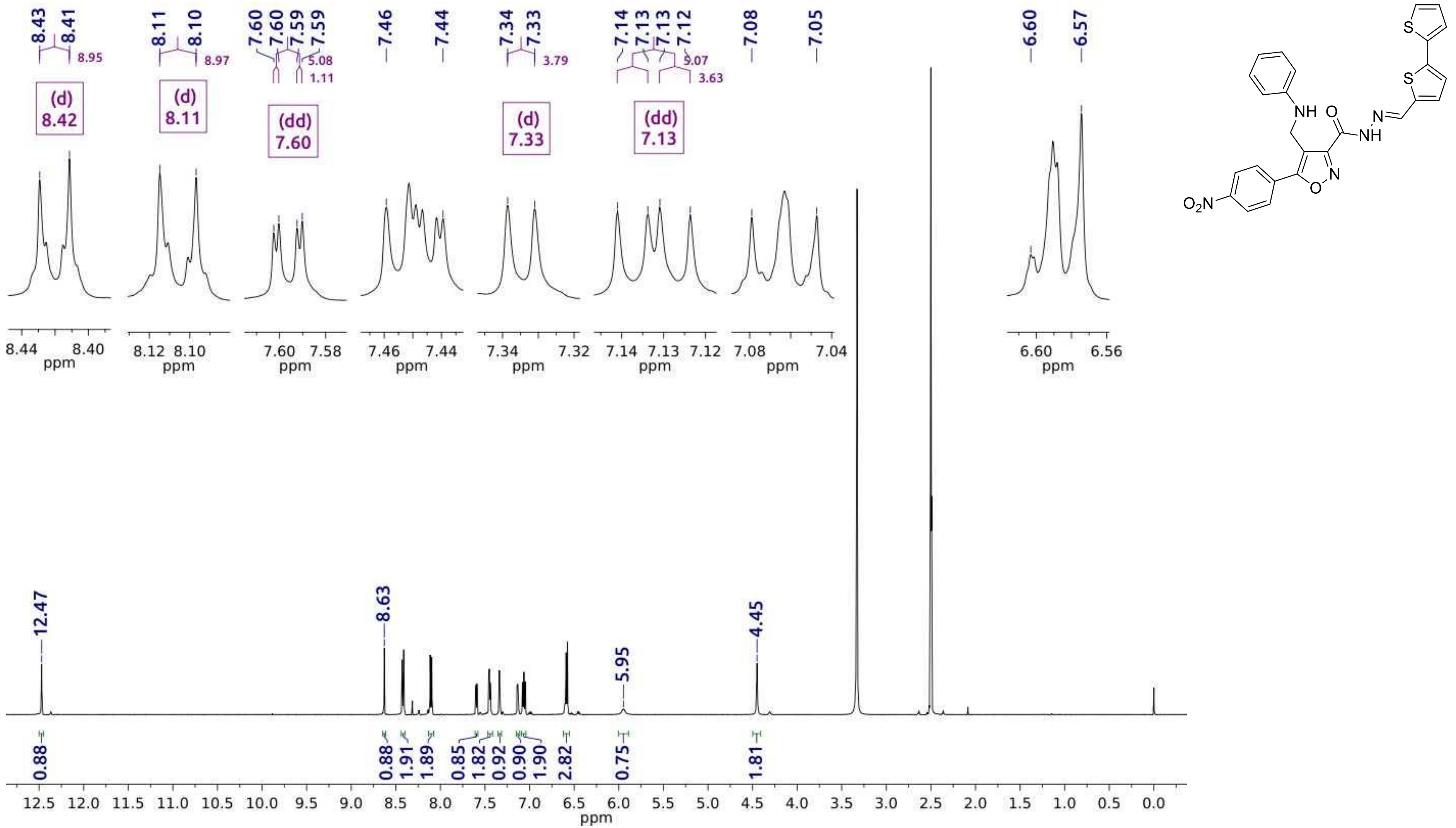

Figure S97 – ^{1}H NMR spectrum of compound **6aa** in DMSO-d_6 at 300.06 MHz.

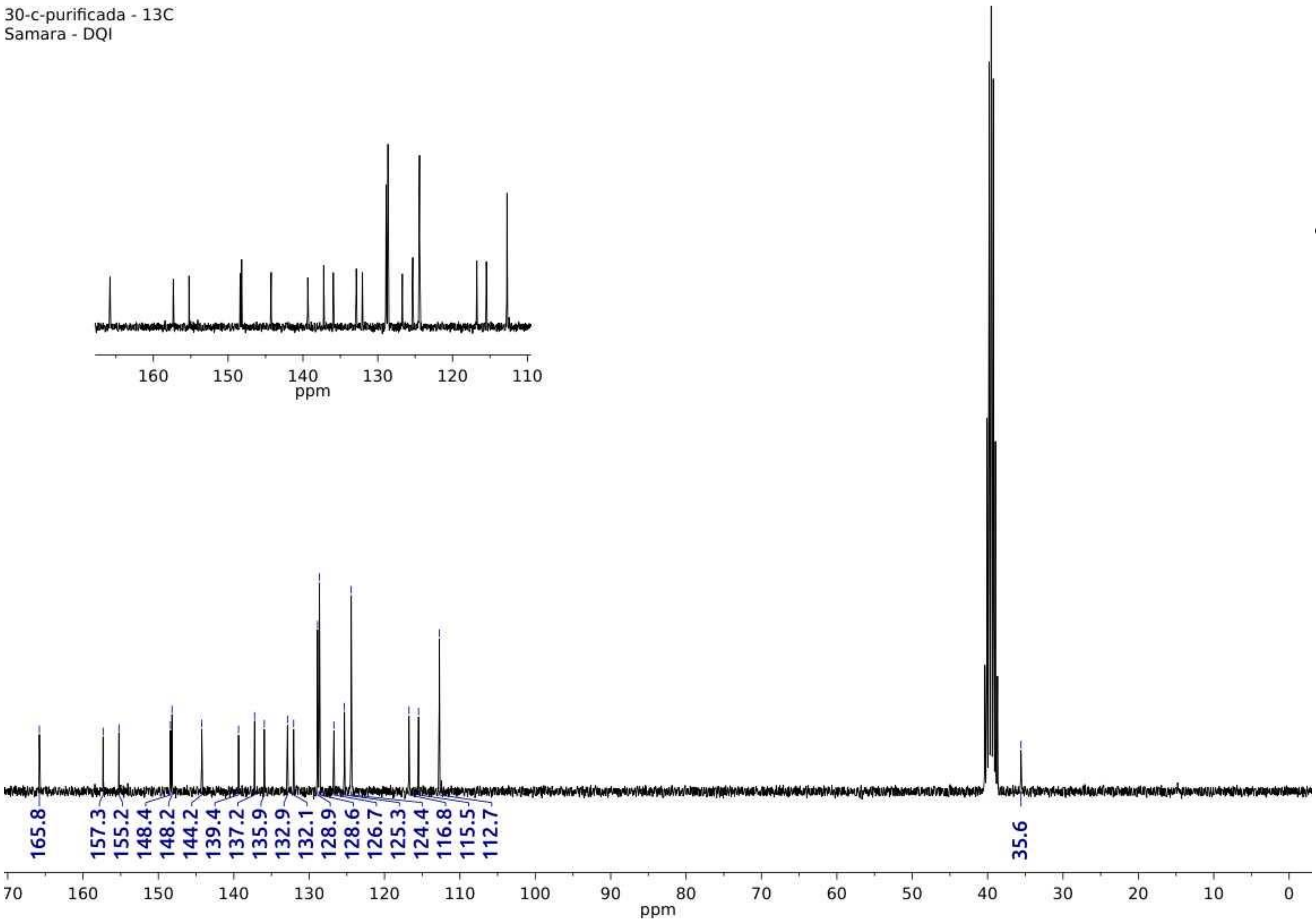

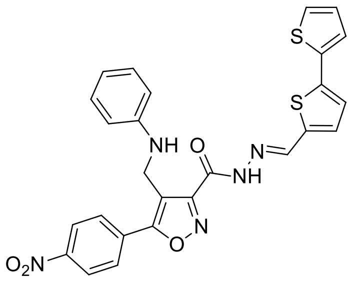

Figure S98 – ^{13}C NMR spectrum of compound **6aa** in DMSO-d_6 at 75.45 MHz.

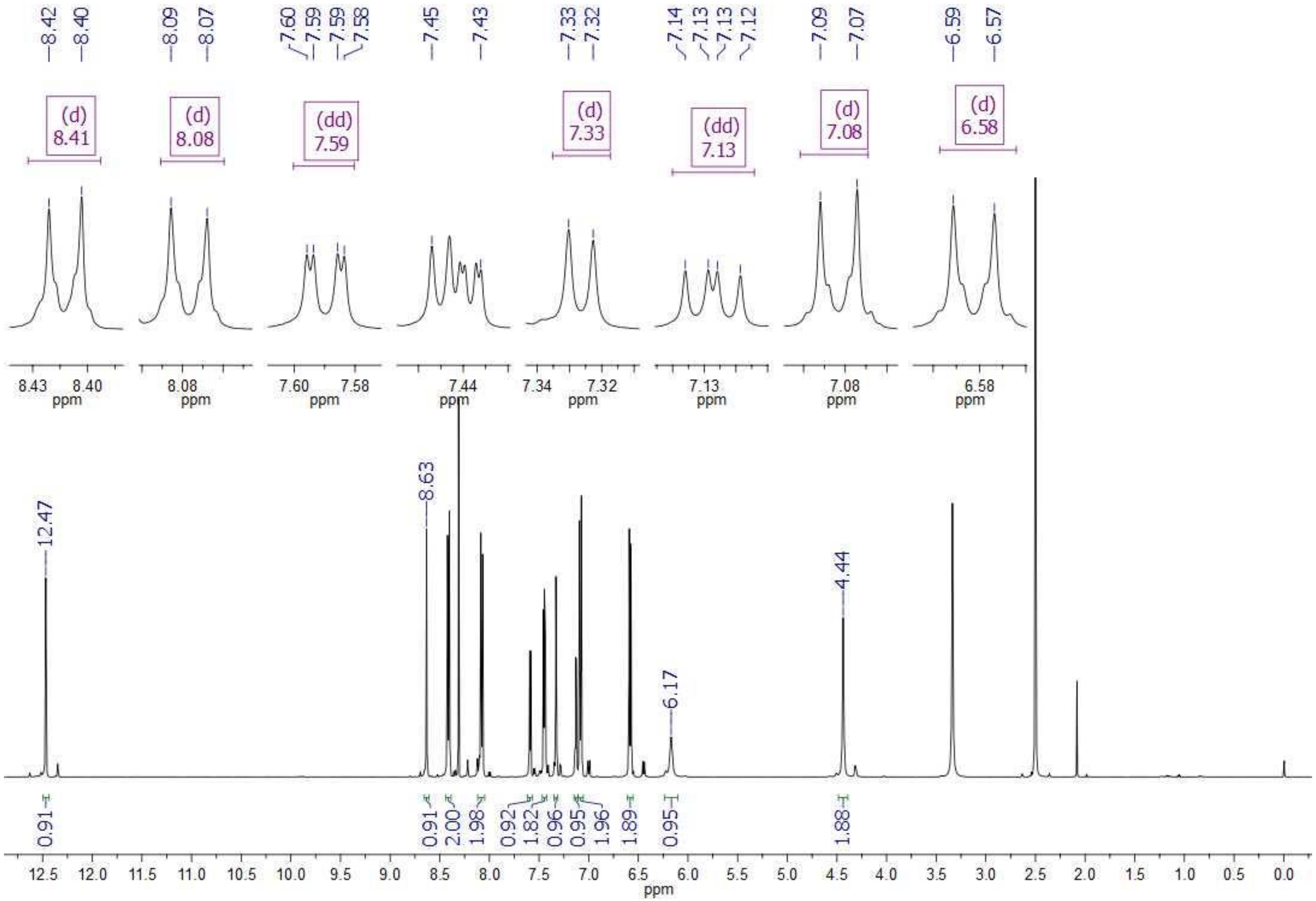

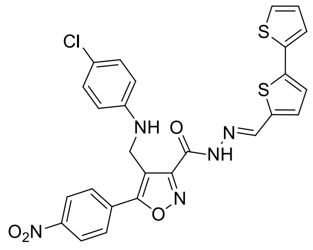

Figure S99 – ^{1}H NMR spectrum of compound **6ab** in DMSO-d_6 at 300.06 MHz.

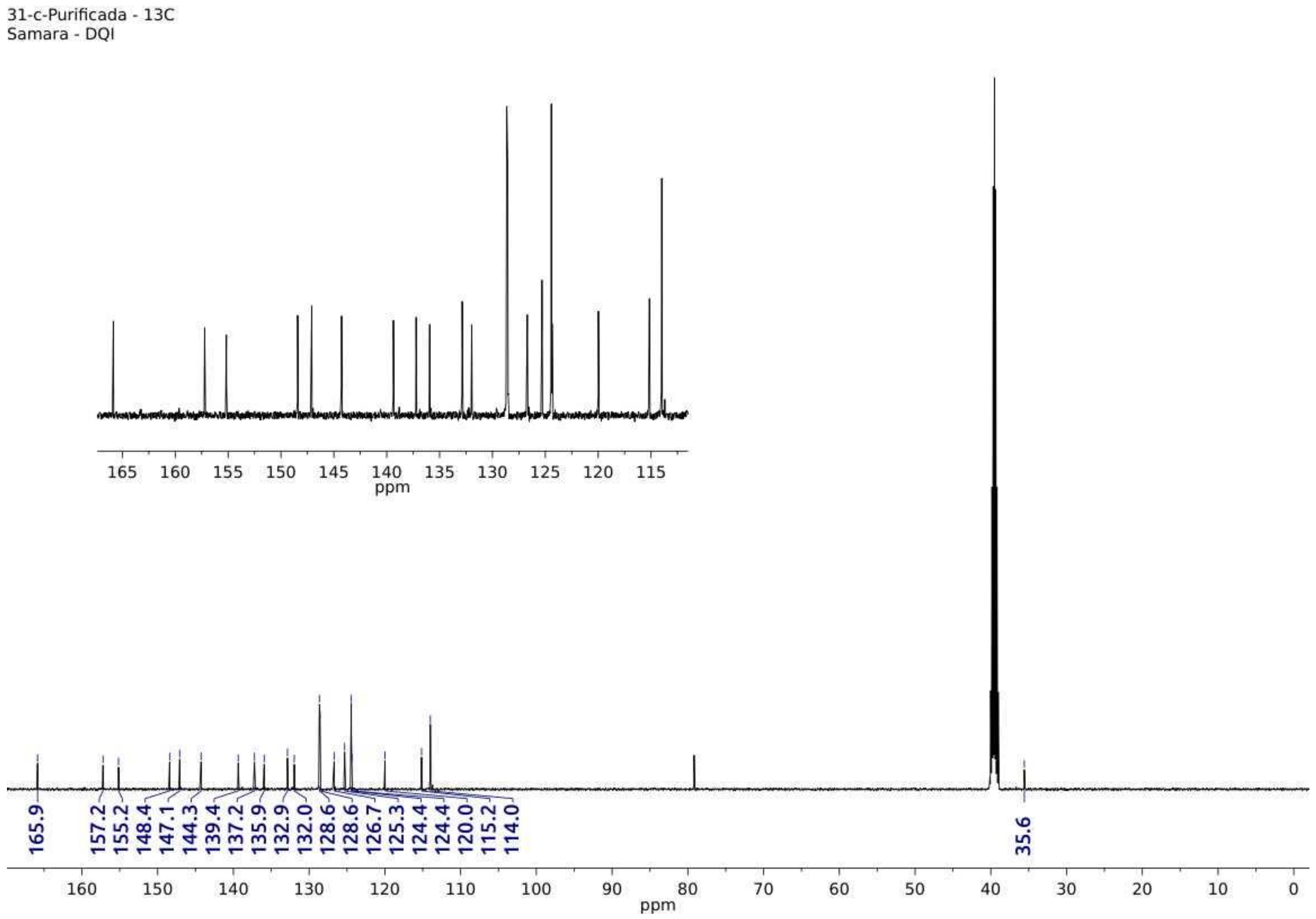

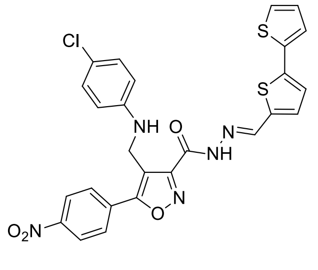

Figure S100 – ^{13}C NMR spectrum of compound **6ab** in DMSO-d_6 at 75.45 MHz.

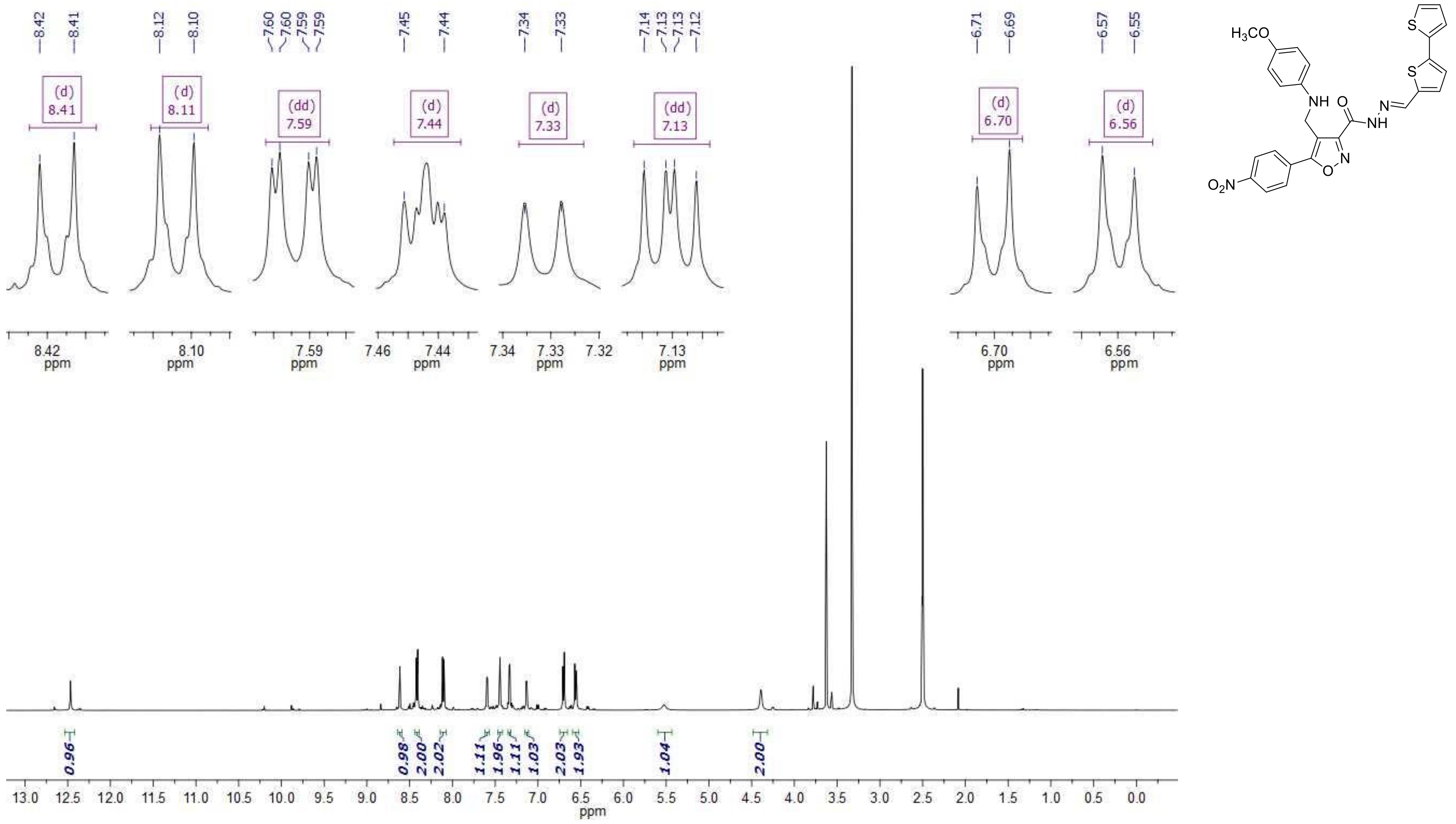

Figure S101 – ^{1}H NMR spectrum of compound **6ac** in DMSO-d_6 at 300.06 MHz.

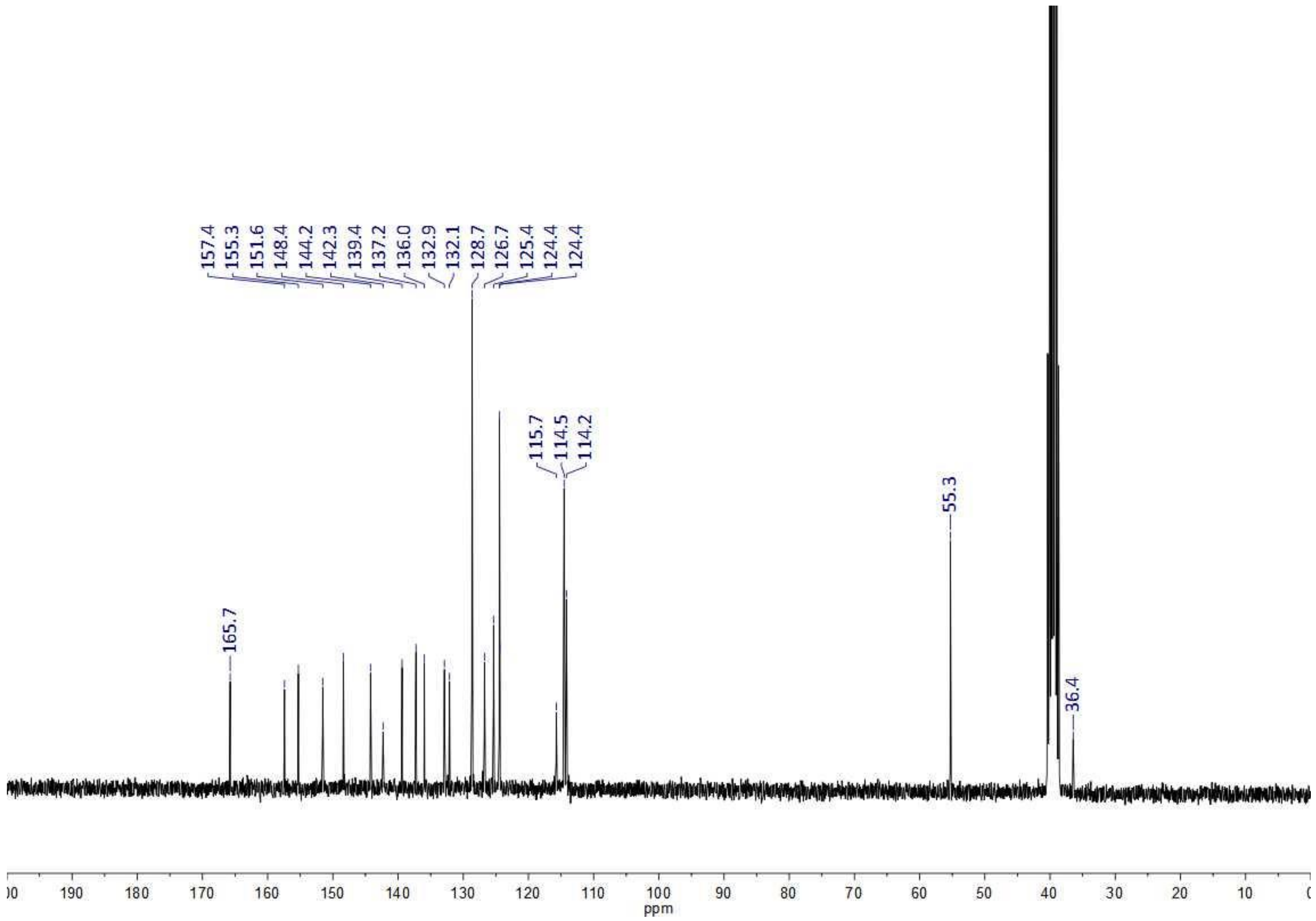

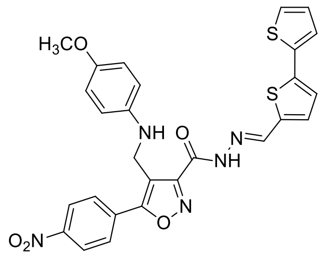

Figure S102 – ^{13}C NMR spectrum of compound **6ac** in DMSO-d_6 at 75.45 MHz.

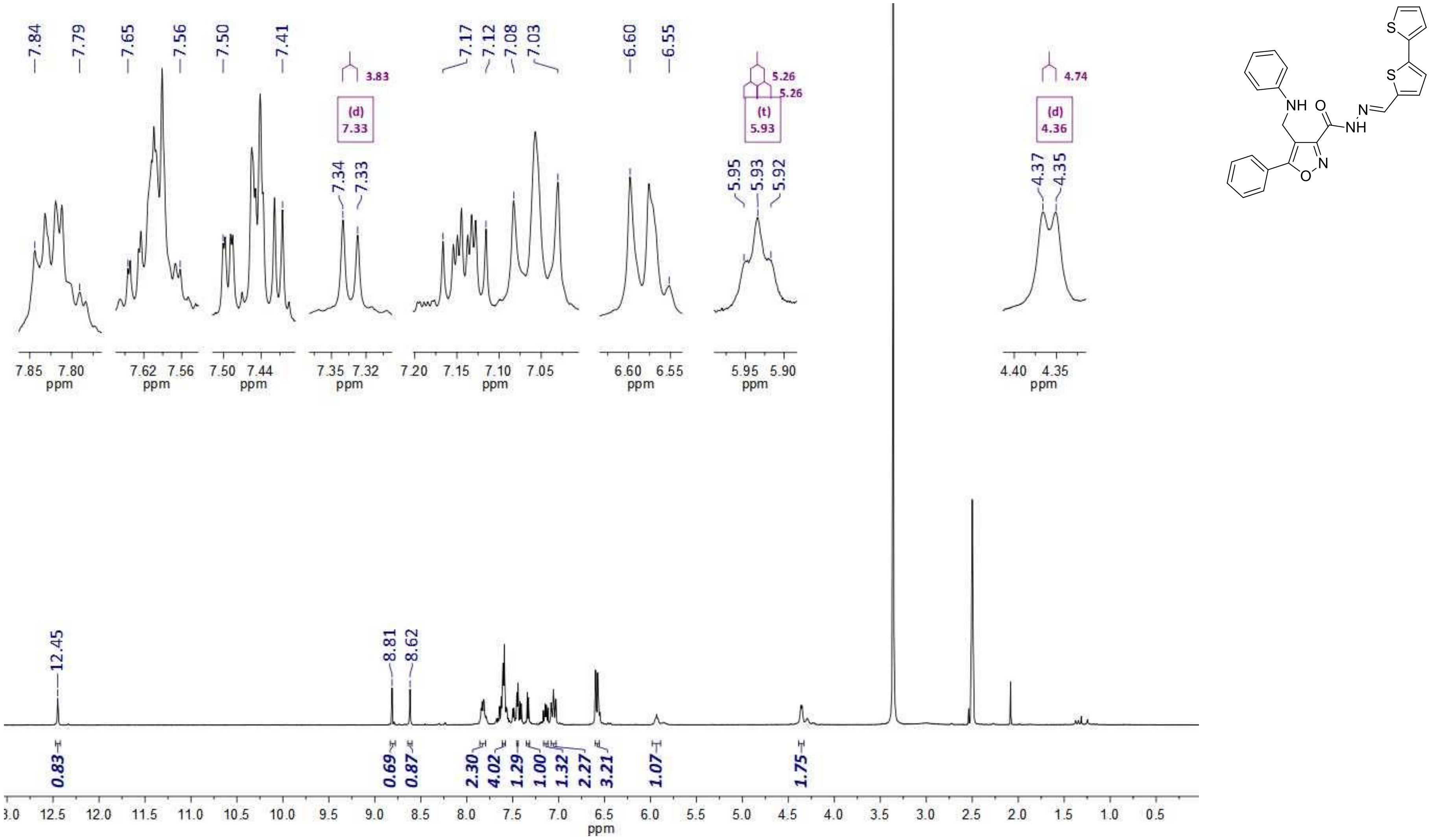

Figure S103 – ^{1}H NMR spectrum of compound **6ba** in DMSO-d_6 at 300.06 MHz.

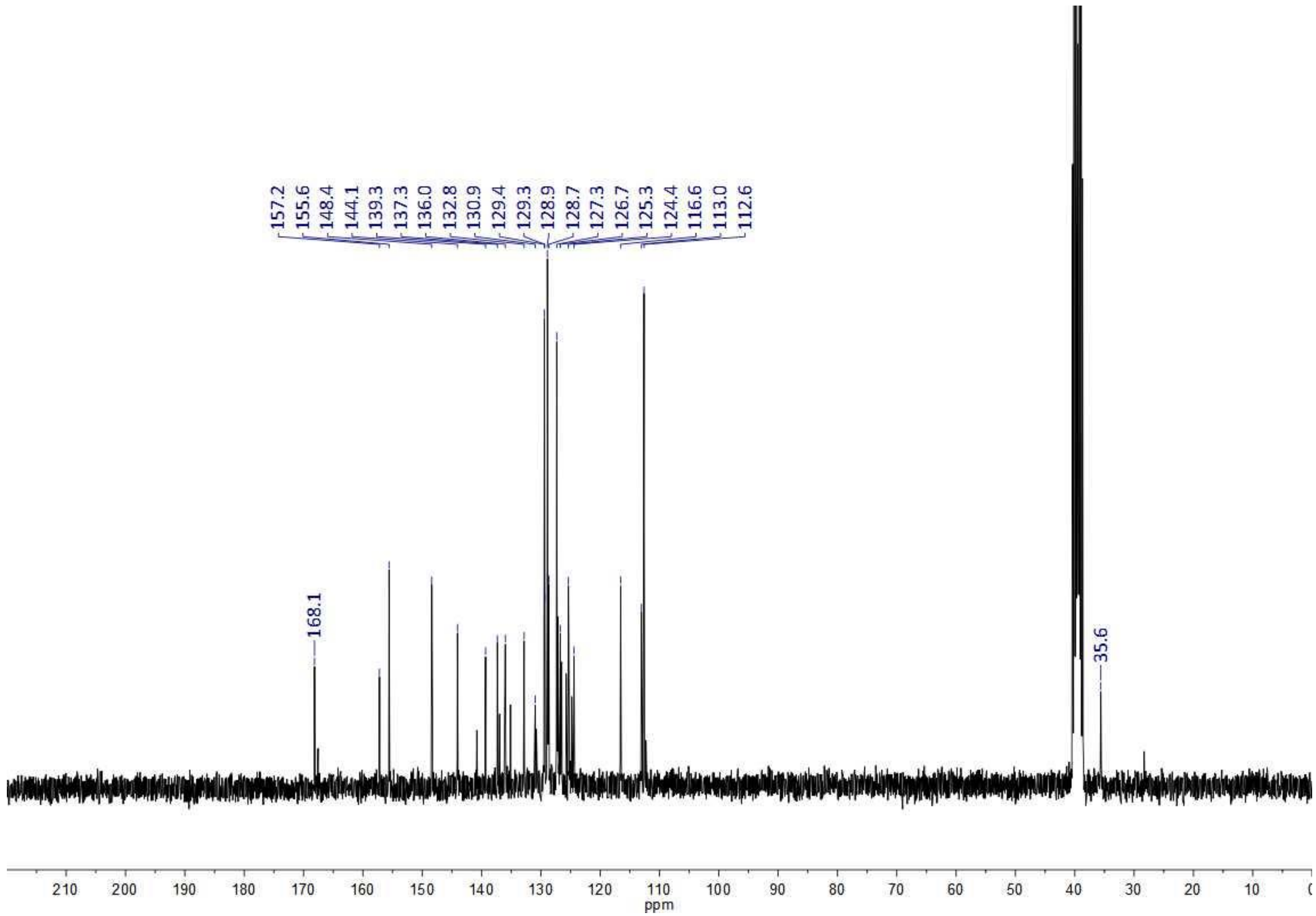

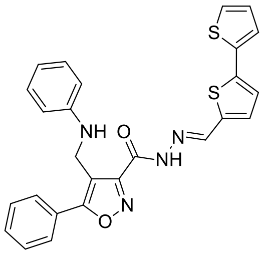

Figure S104 – ^{13}C NMR spectrum of compound **6ba** in DMSO-d_6 at 75.45 MHz.

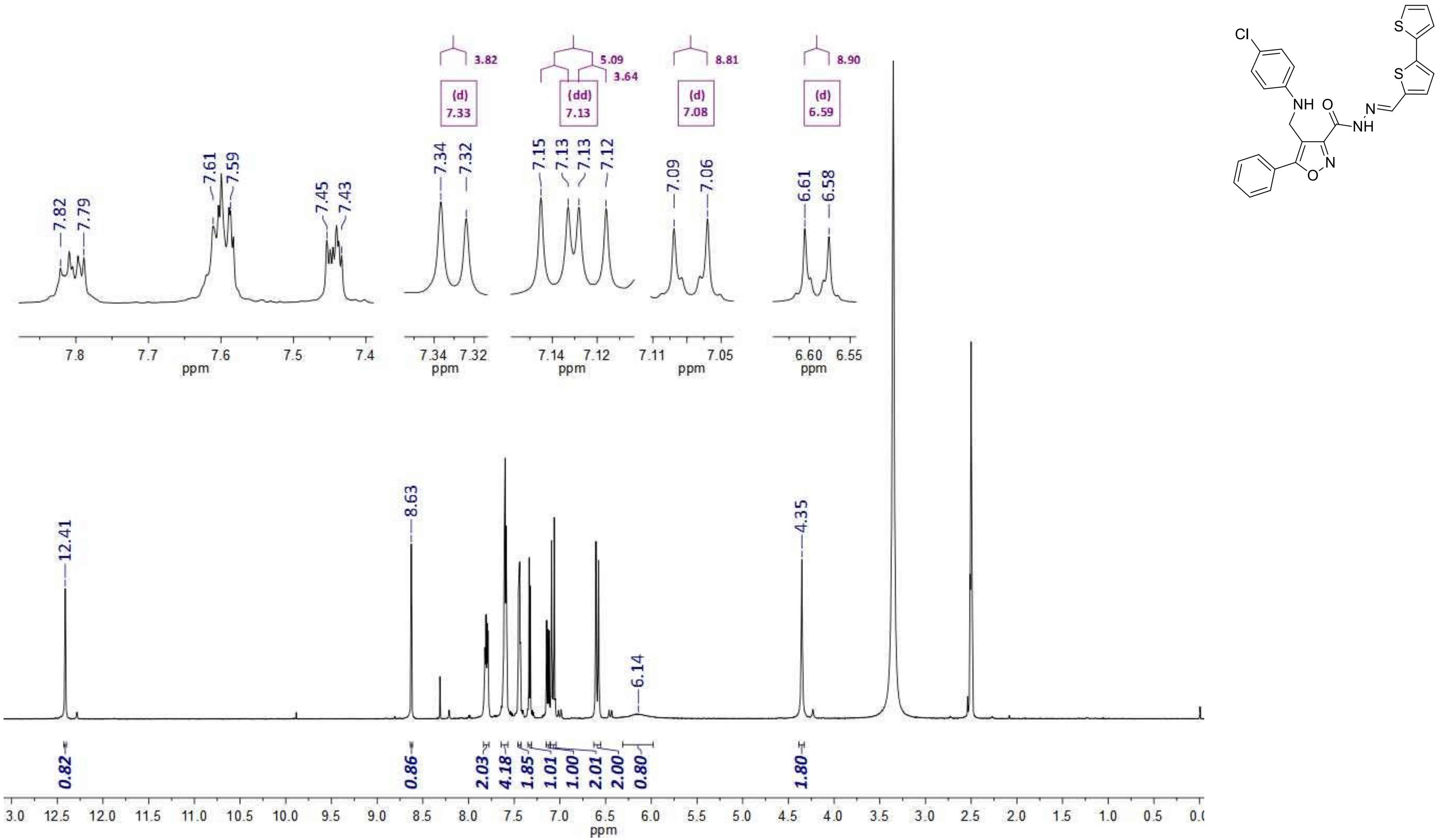

Figure S105 – ^{1}H NMR spectrum of compound **6bb** in DMSO-d_6 at 300.06 MHz.

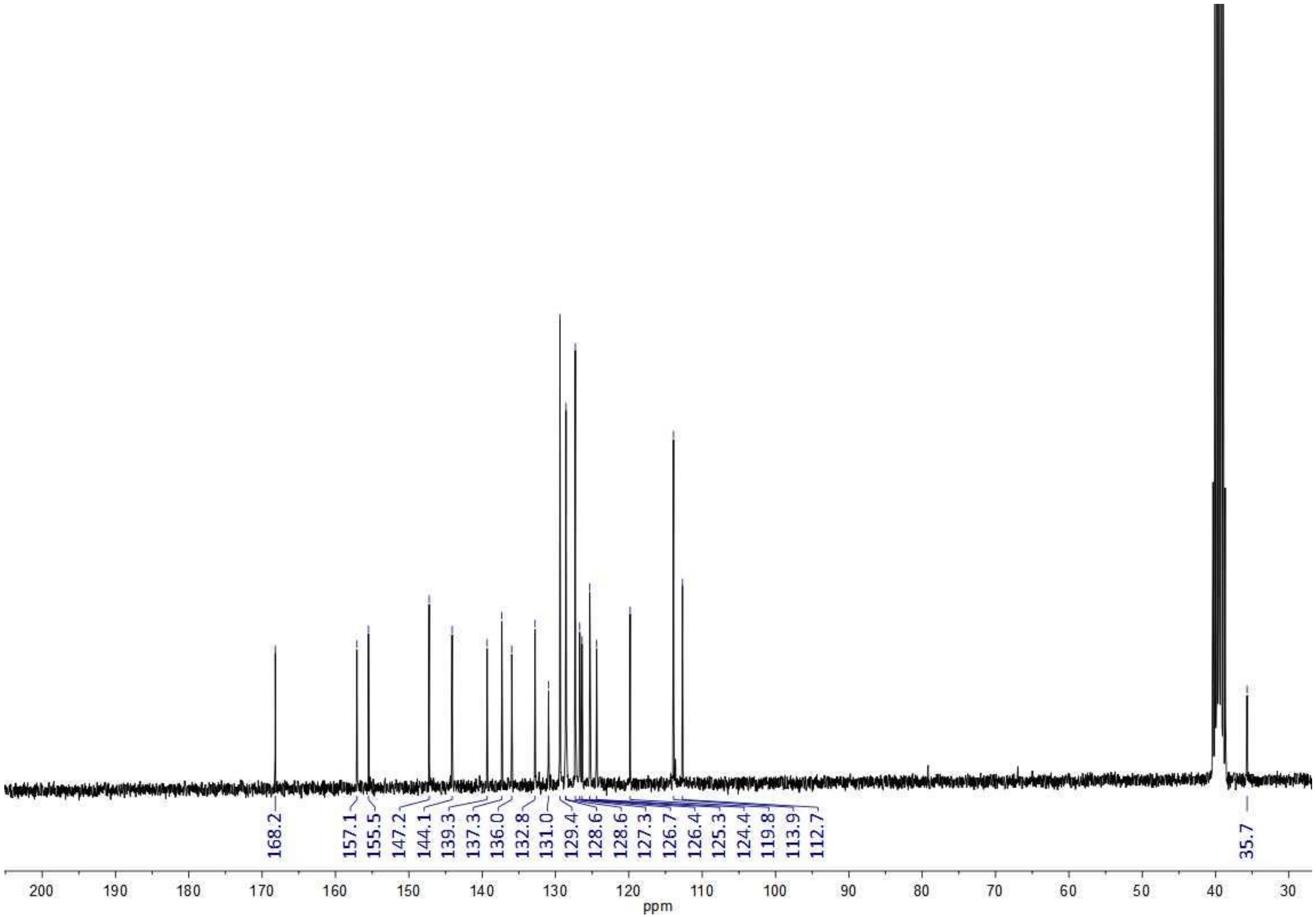

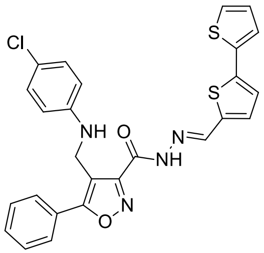

Figure S106 – ^{13}C NMR spectrum of compound **6bb** in DMSO-d_6 at 75.45 MHz.

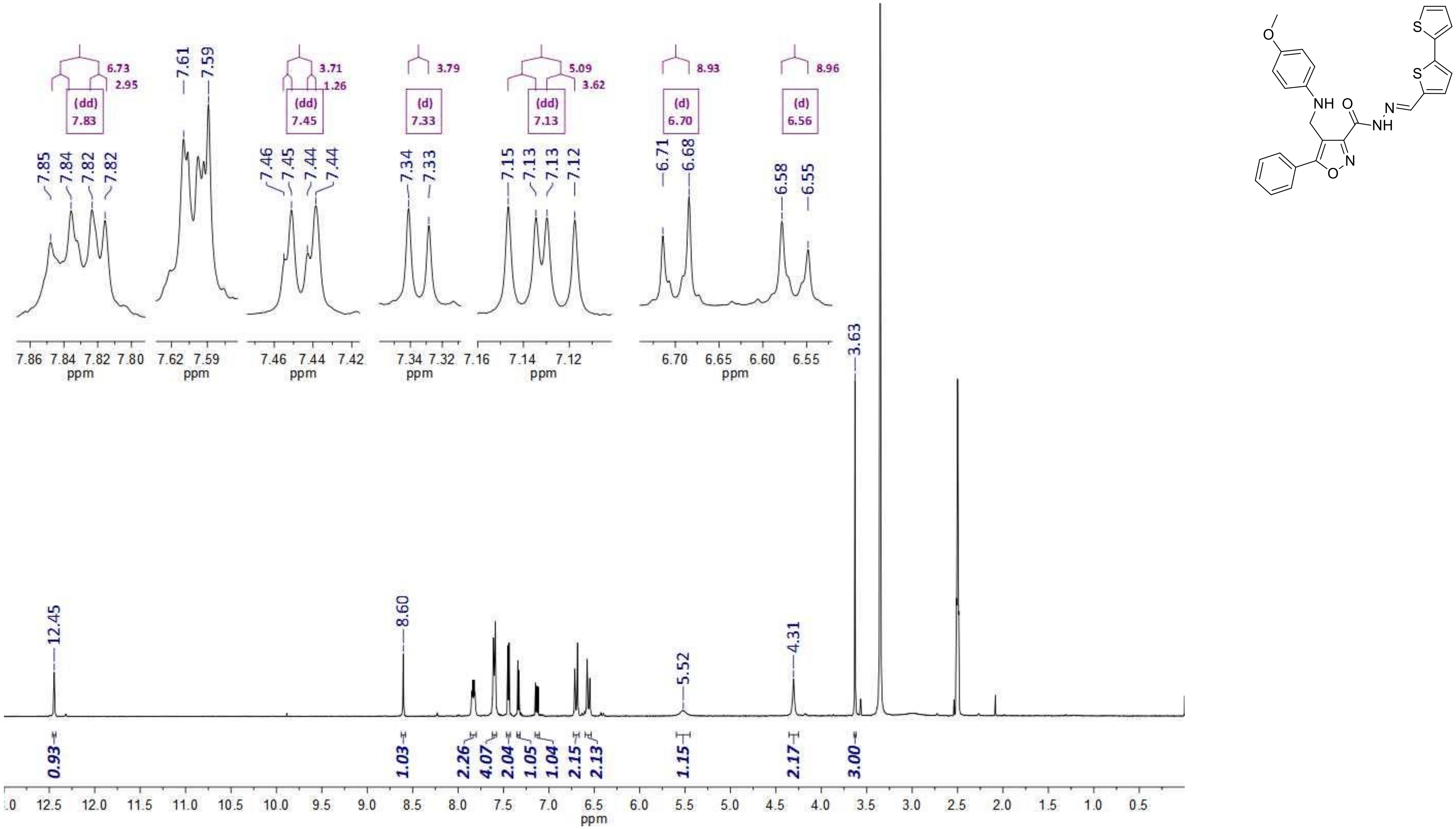

Figure S107 – ^{1}H NMR spectrum of compound **6bc** in DMSO-d_6 at 300.06 MHz.

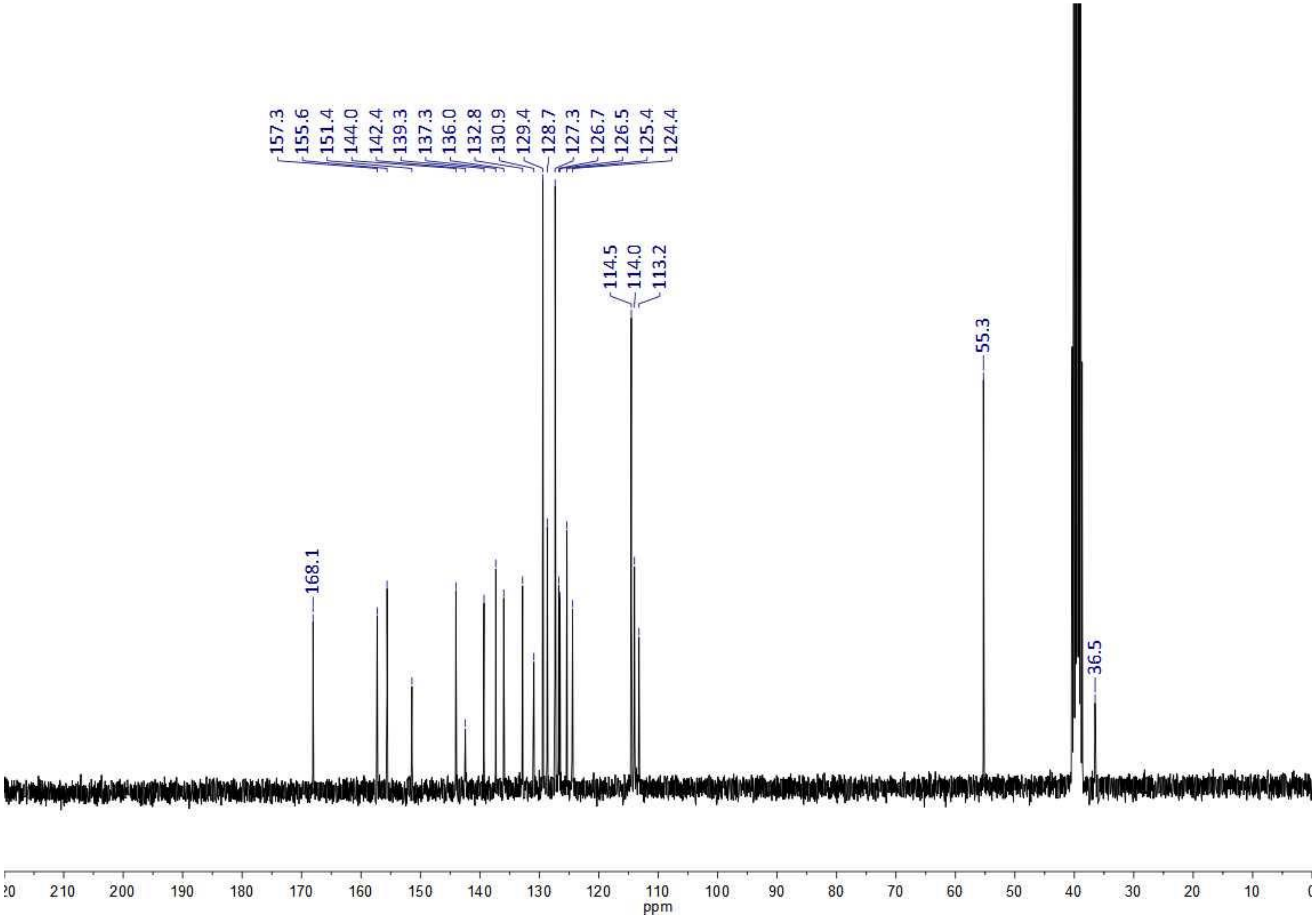

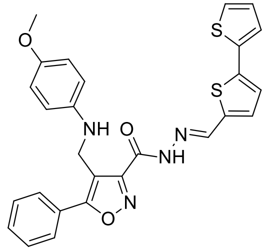

Figure S108 – ^{13}C NMR spectrum of compound **6bc** in DMSO-d_6 at 75.45 MHz.

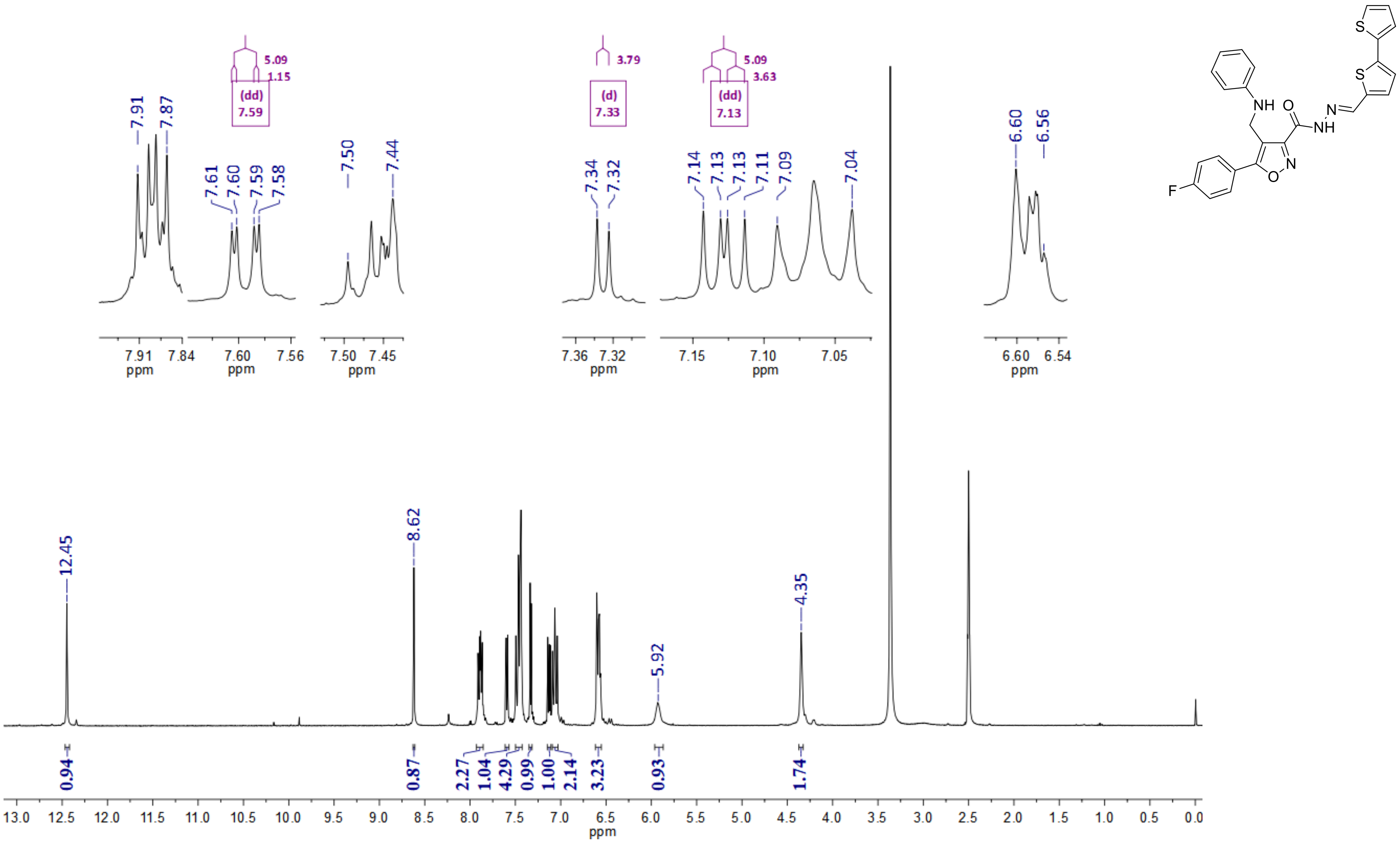

Figure S109 – ^{1}H NMR spectrum of compound **6ca** in DMSO-d_6 at 300.06 MHz.

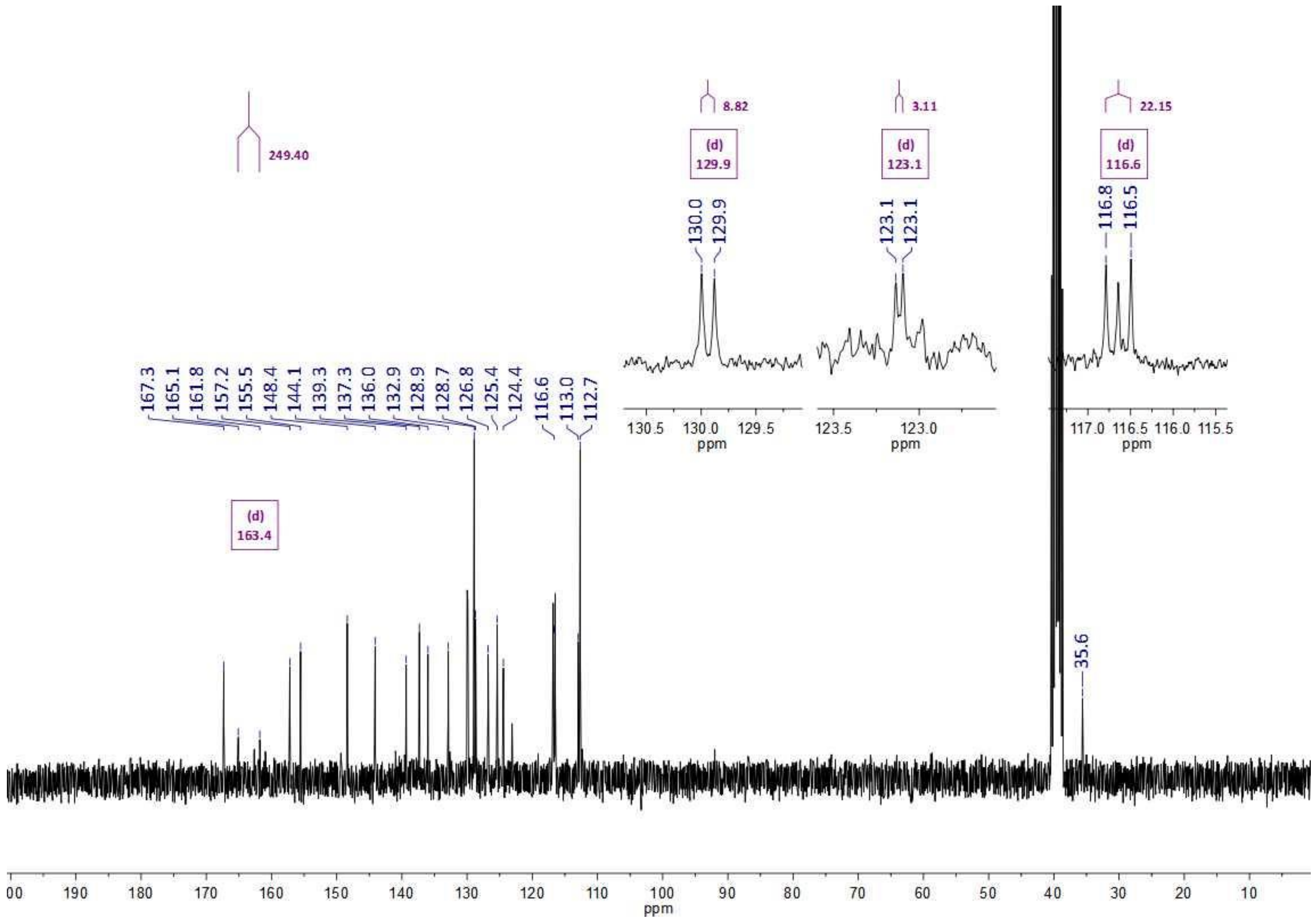

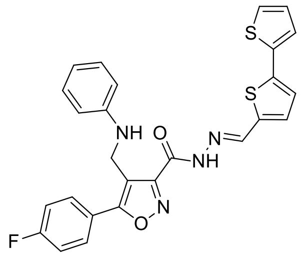

Figure S110 – ^{13}C NMR spectrum of compound **6ca** in DMSO-d_6 at 75.45 MHz.

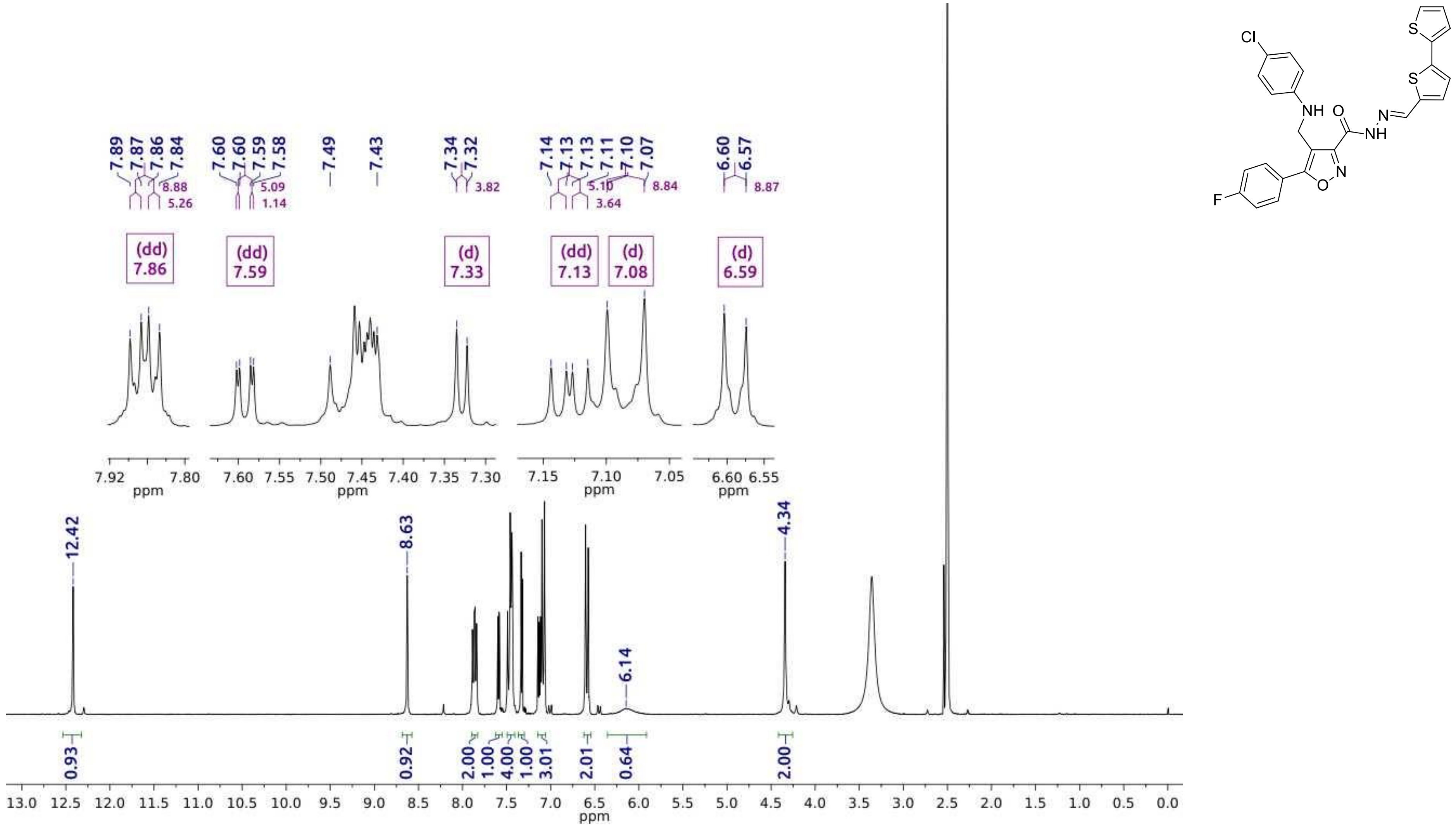

Figure S111 – ^{1}H NMR spectrum of compound **6cb** in DMSO-d_6 at 300.06 MHz.

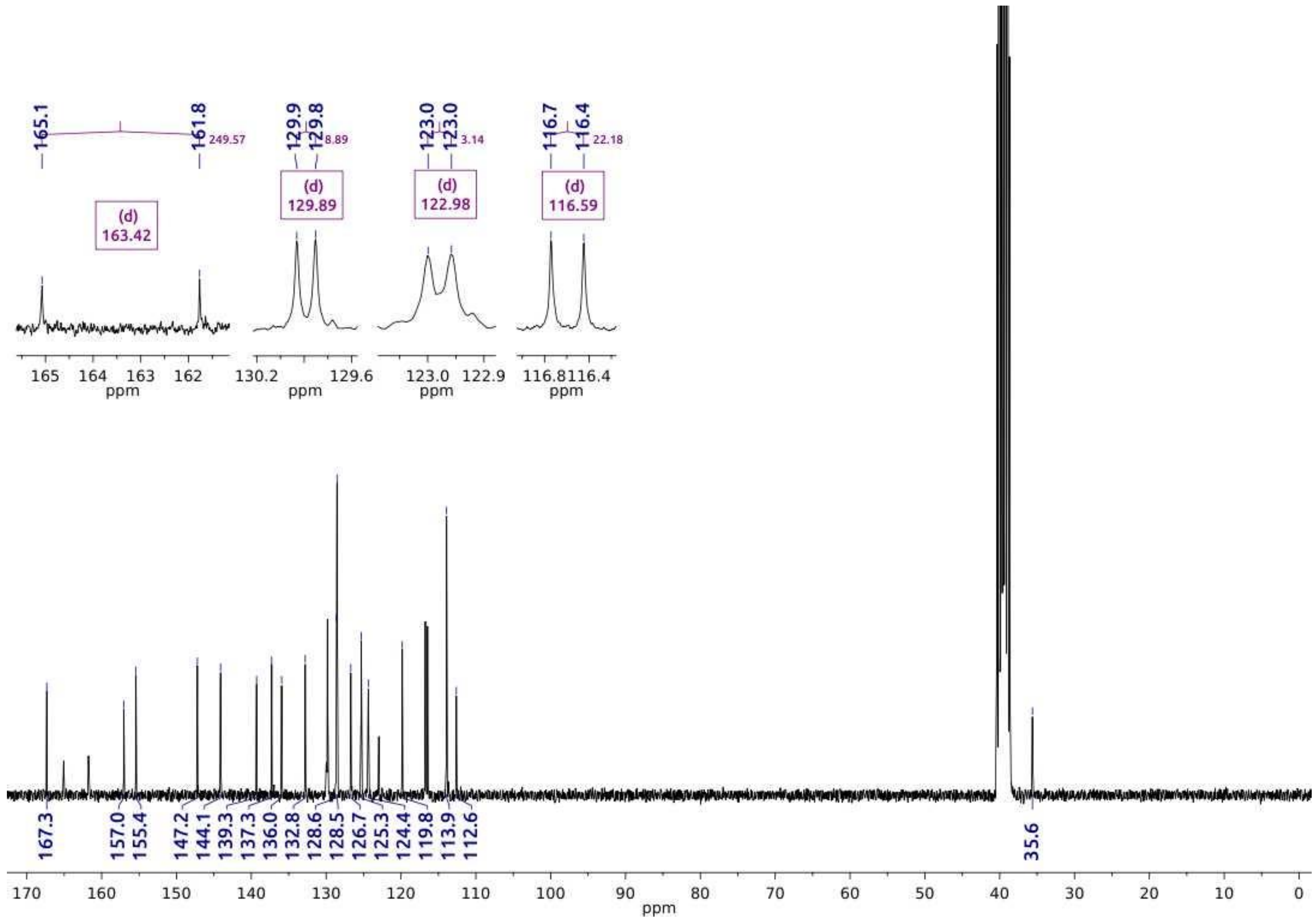

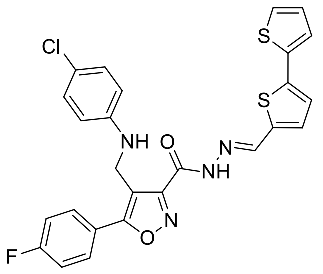

Figure S112 – ^{13}C NMR spectrum of compound **6cb** in DMSO-d_6 at 75.45 MHz.

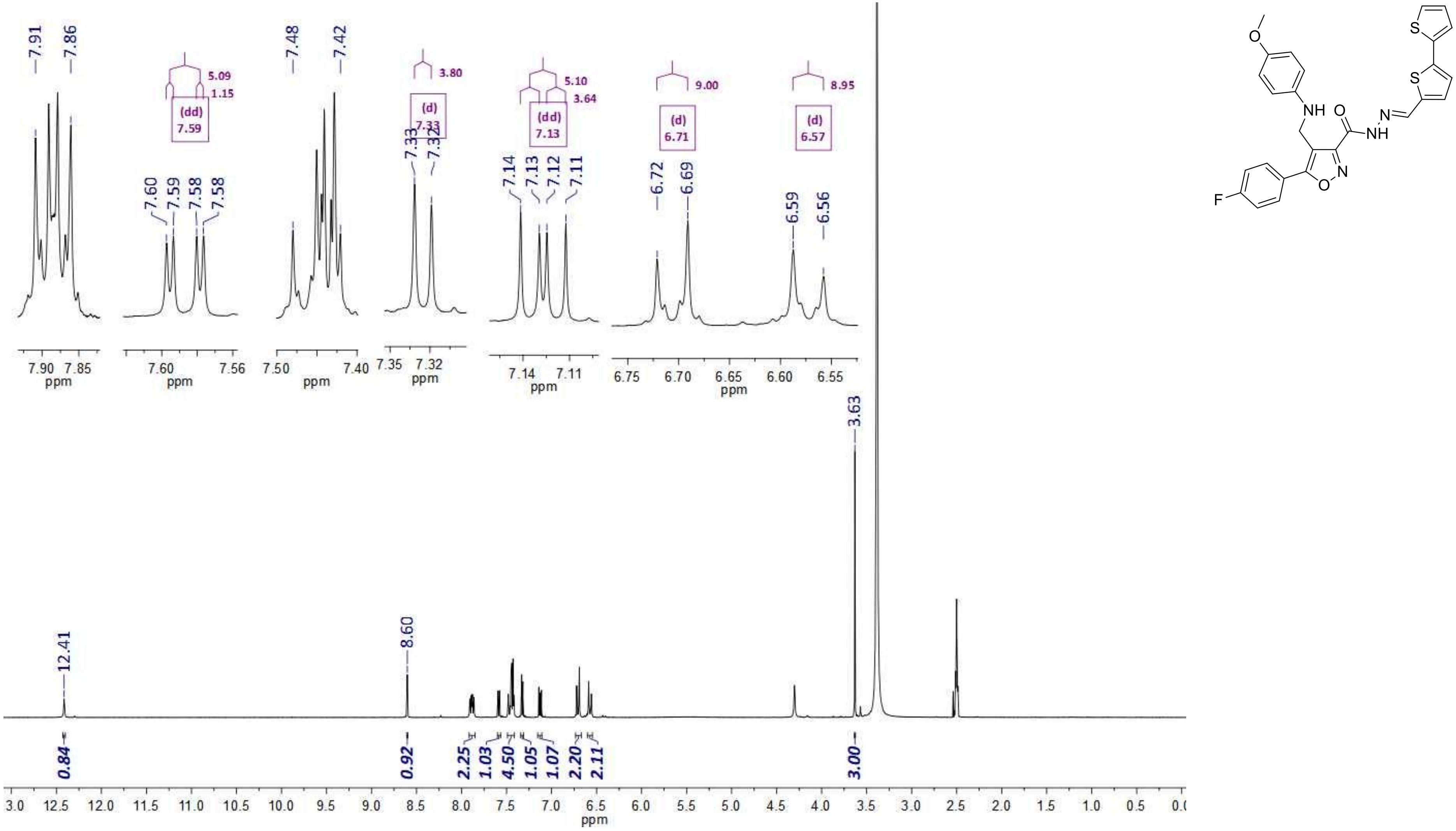

Figure S113 – ^{1}H NMR spectrum of compound **6cc** in DMSO-d_6 at 300.06 MHz.

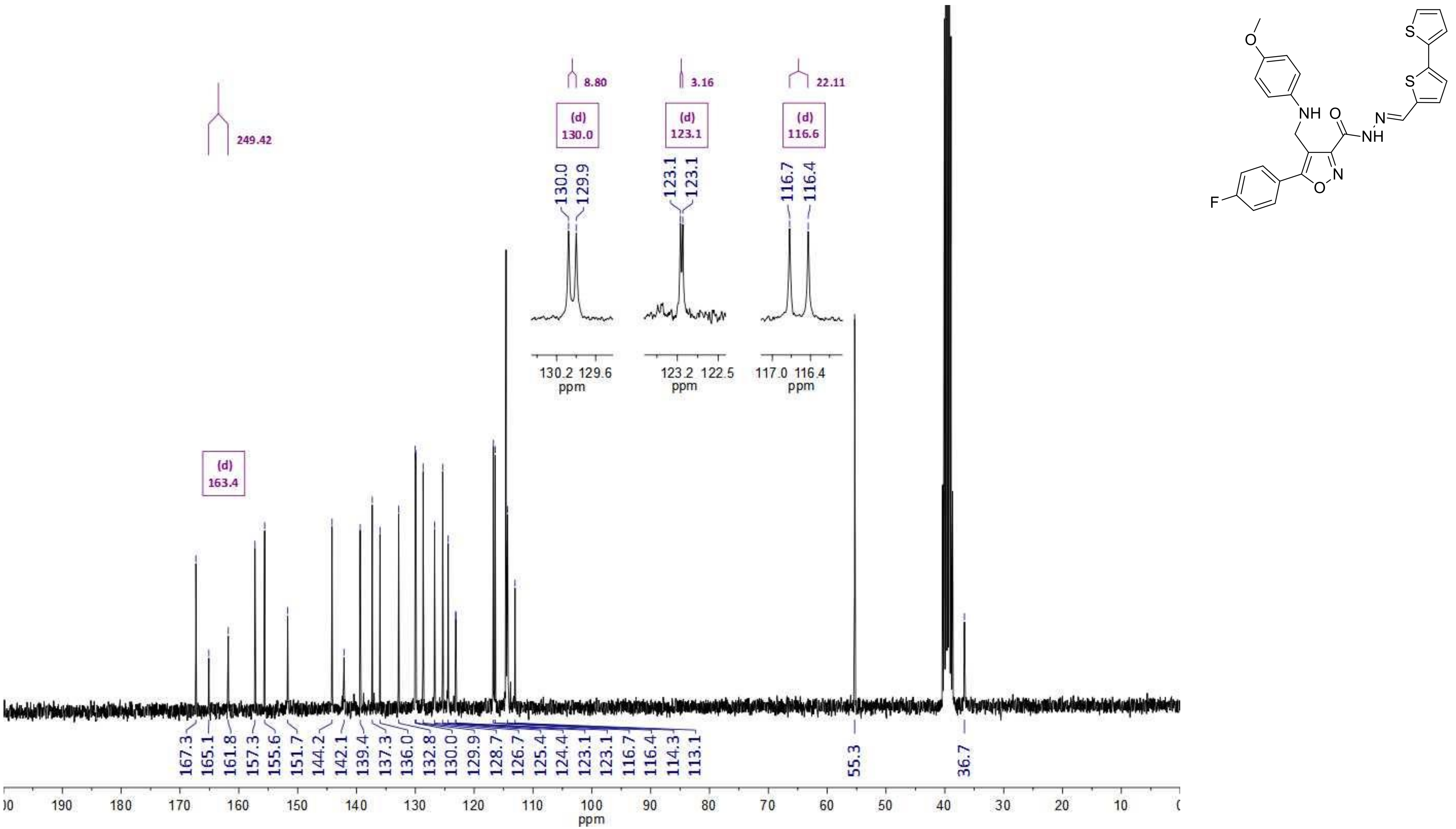

Figure S114 – ^{13}C NMR spectrum of compound **6cc** in DMSO-d_6 at 75.45 MHz.

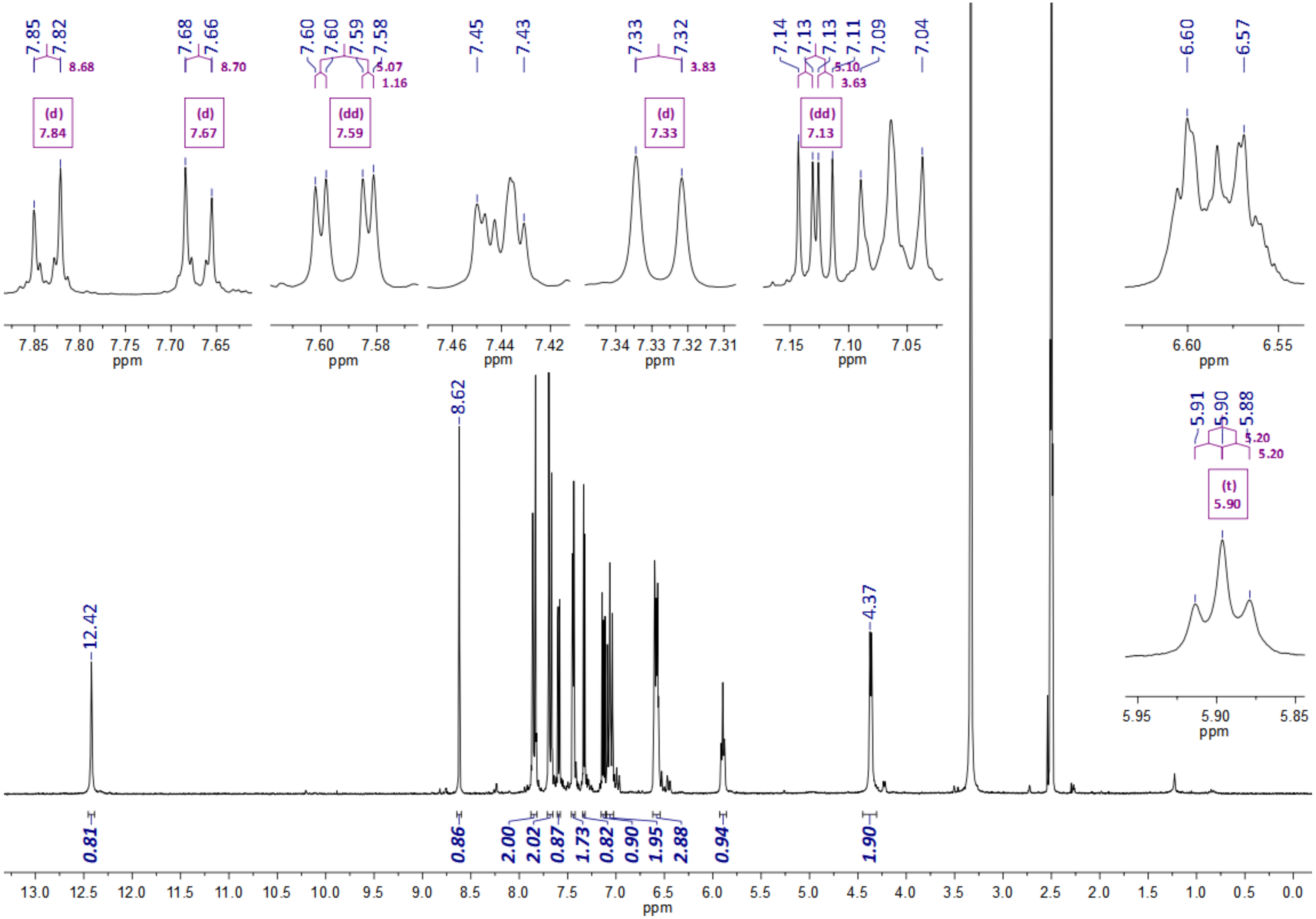

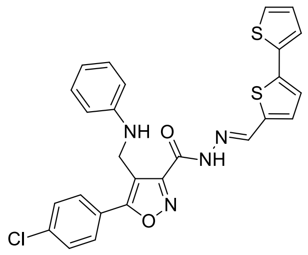

Figure S115 – ^{1}H NMR spectrum of compound **6da** in DMSO-d_6 at 300.06 MHz.

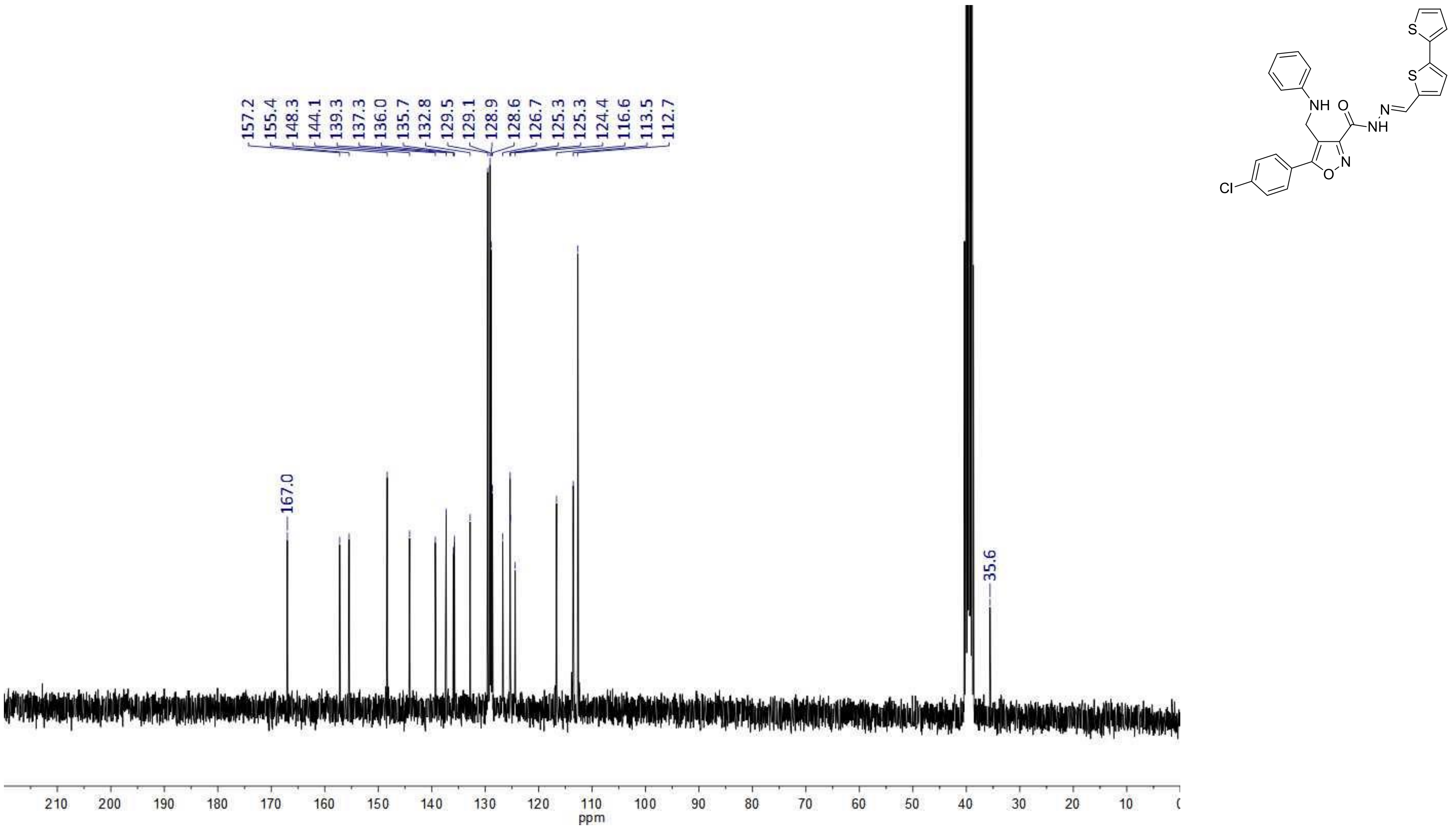

Figure S116 – ^{13}C NMR spectrum of compound **6da** in DMSO-d_6 at 75.45 MHz.

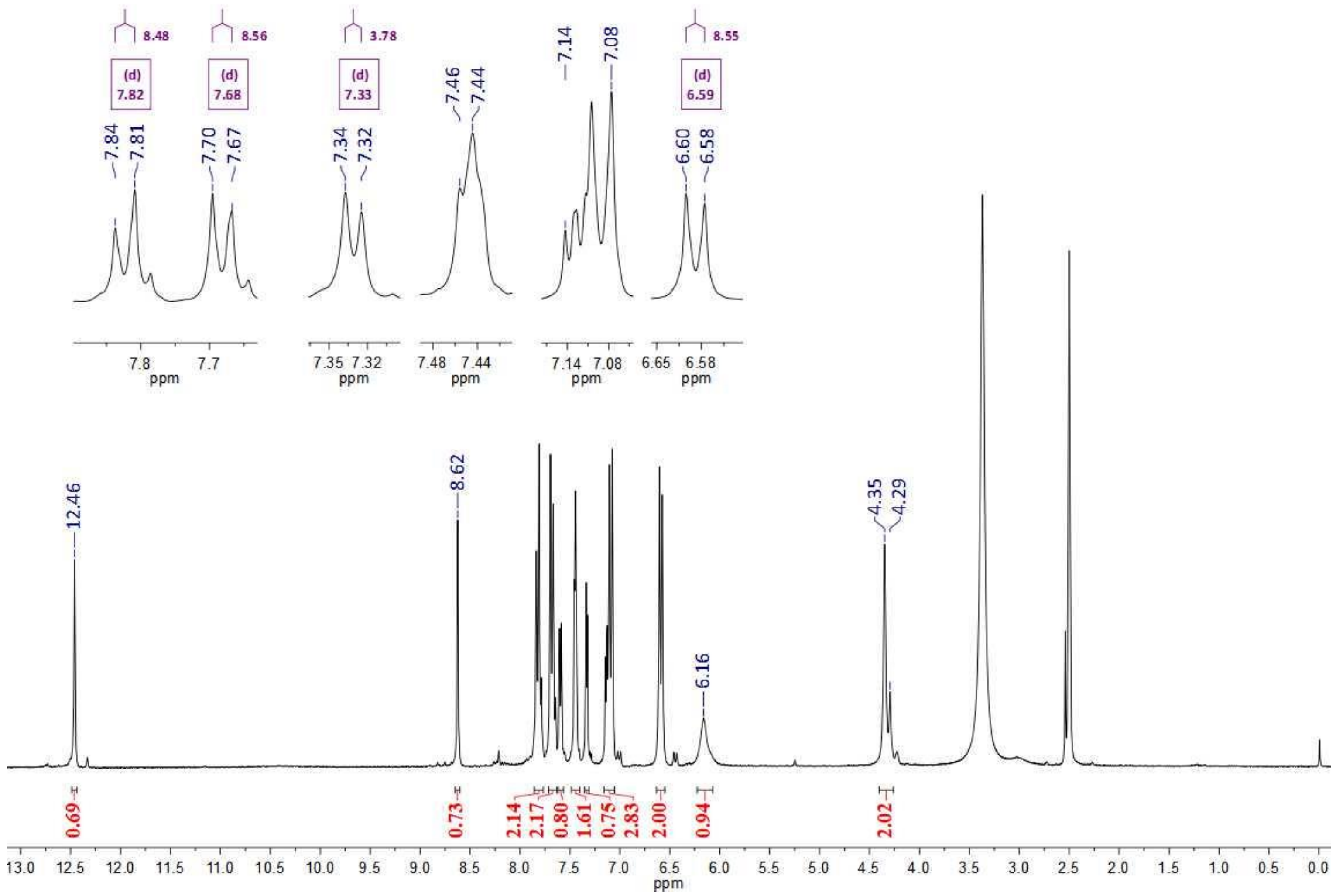

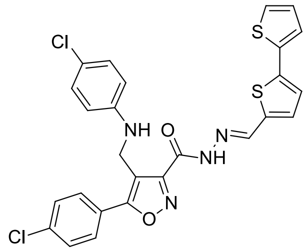

Figure S117 – ^{1}H NMR spectrum of compound **6db** in DMSO-d_6 at 300.06 MHz.

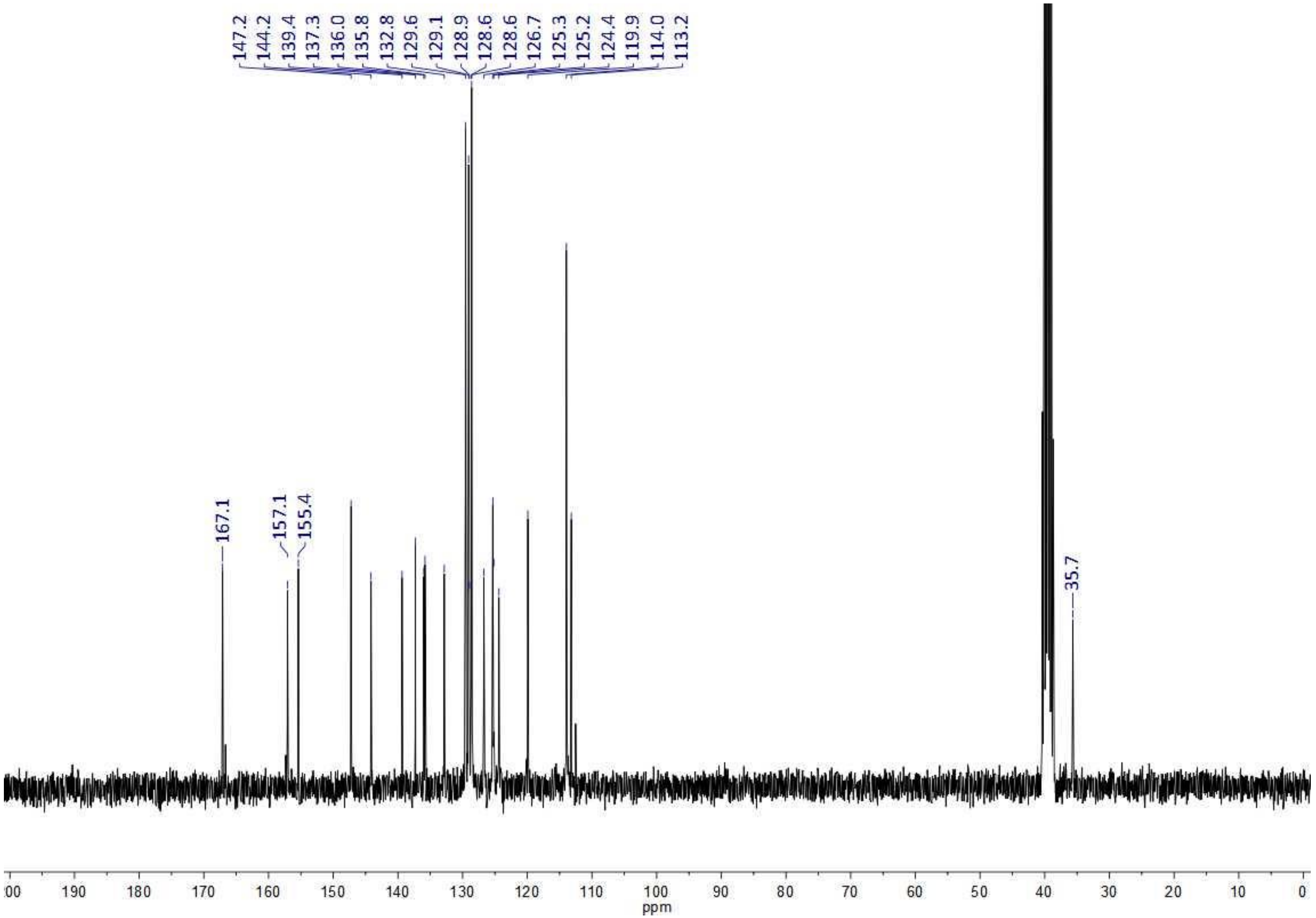

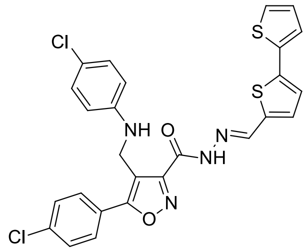

Figure S118 – ^{13}C NMR spectrum of compound **6db** in DMSO-d_6 at 75.45 MHz.

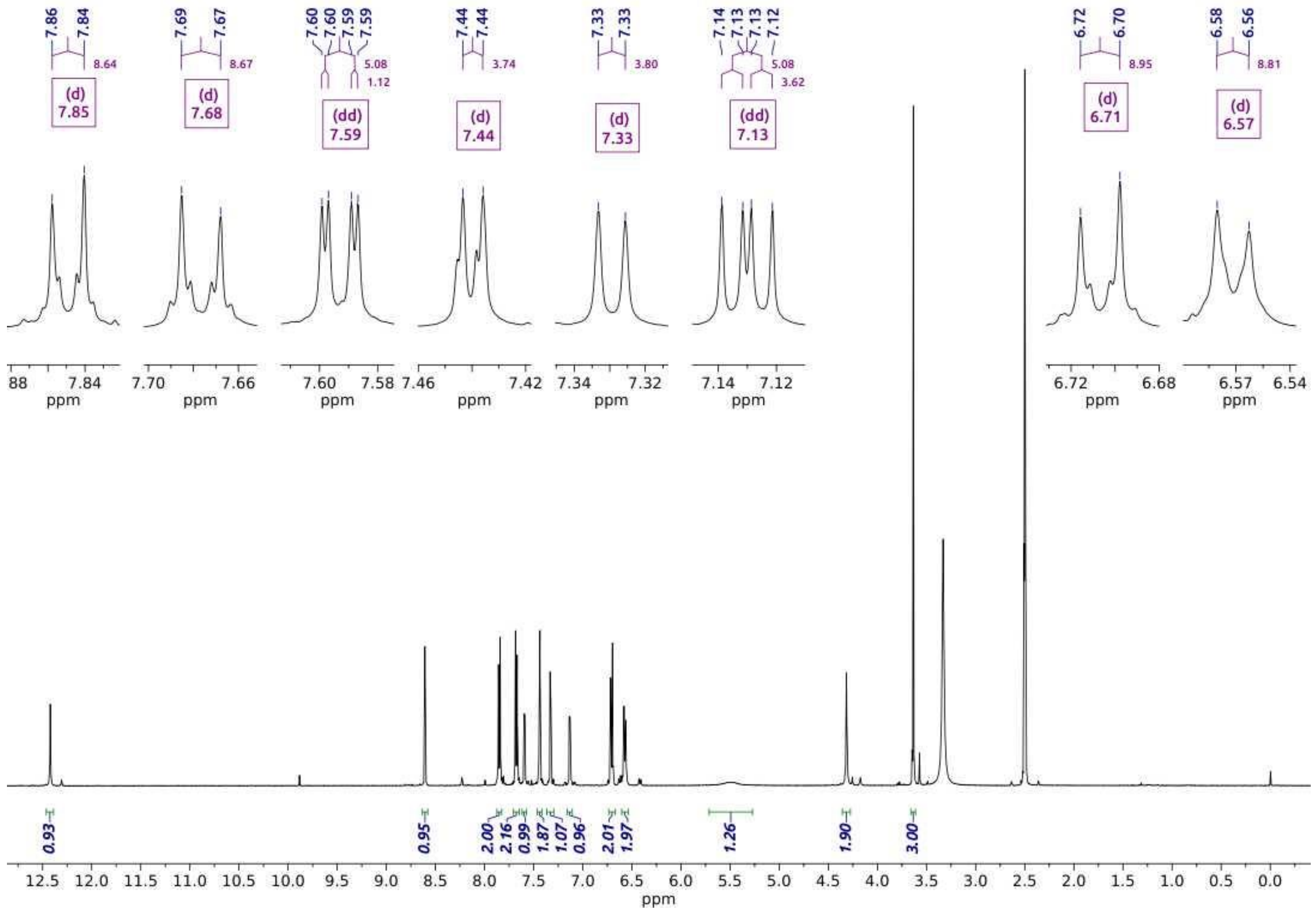

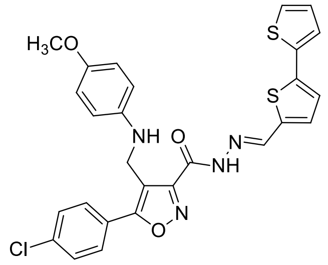

Figure S119 – ^{1}H NMR spectrum of compound **6dc** in DMSO-d_6 at 300.06 MHz.

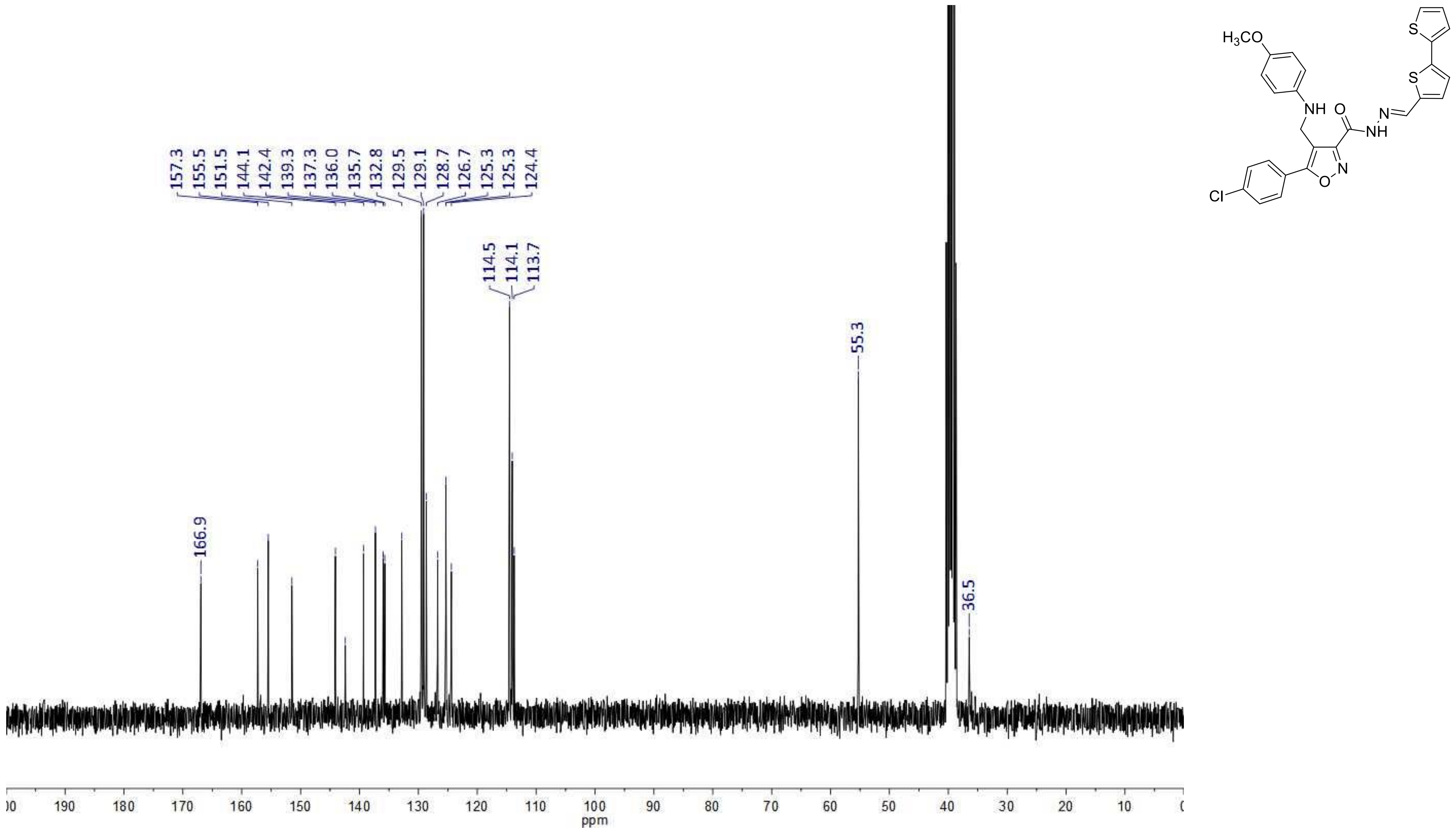

Figure S120 – ^{13}C NMR spectrum of compound **6dc** in DMSO-d_6 at 75.45 MHz.

References

[1] D. D. Perrin, L. F. Armarego, in Purification of Laboratory Chemicals, Pergamon Press, New York, 3rd ed, 1996.

[2] F. A. Rosa, P. Machado, M. Rossatto, P. S. Vargas, H. G. Bonacorso, N. Zanatta, M. A. P. Martins, *Synlett* 2007, 3165–3171
